# Supplementary material for: p-nitrobenzyloxycarbonyl protective group as key to automated glycan assembly of neutral human milk oligosaccharides
Source: Nat Commun. 2025 Dec 5;16:10941. doi: 10.1038/s41467-025-66557-3 (PMC12686065; doi:10.1038/s41467-025-66557-3)
Supplement: Supplementary file 1 — Supplementary Information [file 41467_2025_66557_MOESM1_ESM.pdf]

Supplementary information

p-Nitrobenzyloxycarbonyl Protective Group as Key to Automated Glycan Assembly of Neutral Human Milk Oligosaccharides

Mei-Huei Lin[1,2‡], Yan-Ting Kuo[1,2,3‡], Kim Le Mai Hoang[3], and Peter H. Seeberger[1,2*]

[1]Biomolecular Systems Department, Max Planck Institute of Colloids and Interfaces, 14476, Potsdam, Germany.

[2]Institute of Chemistry and Biochemistry, Freie Universität Berlin, 14195, Berlin, Germany.

[3]GlycoUniverse GmbH &Co. KGaA, 14476, Potsdam, Germany.

‡These authors contributed equally to this work.

*corresponding author: Peter.Seeberger@mpikg.mpg.de

Table of Contents

1. General Information 1

2. Optimization of N-*p*NZ Protected Glucosamine in AGA 2

2.1 Temperature 2

2.2 Pyridine washing 2

2.3 Comparison of *p*NZ-protected, TCA-protected and Cbz-protected glucosamine building blocks 3

3. Synthesis of Building Blocks 4

4. Materials and Conditions for Automated Synthesis 24

4.1 Materials and Measurements 24

4.2 Preparation of Stock Solutions 25

4.3 Modules for Automated Synthesis 26

4.4 Post-synthesizer Manipulation 27

4.4.1 Cleavage from Solid Support: Protected Oligosaccharides 27

4.4.2 General Deprotection Procedure: 27

4.5 Purification 28

4.5.1 Analytical NP-HPLC 28

4.5.2 Preparative NP-HPLC 28

4.5.3 Analytical RP-HPLC 28

4.5.4 Preparative RP-HPLC 29

5. Automated Glycan Assembly of Oligo-LacNAc and Human Milk Oligosaccharides 30

5.1 Protected Oligo-LacNAc: Tetramer (**10**) 30

5.2 Protected Oligo-LacNAc: Hexamer (**18**) 32

5.3 Protected Oligo-LacNAc: Octamer (**19**) 34

5.4 Protected Lacto-*N*-neotetraose (**20**) 36

5.5 Protected *para*-Lacto-*N*-neohexaose (**21**) 38

5.6 Protected Lacto-*N*-tetraose (**22**) 41

5.7 Protected Lacto-*N*-fucopentaose III (**23**) 44

5.8 Protected Lacto-*N*-fucopentaose II (**24**) 47

5.9.1 *p*NZ-protected Lacto-*N*-neohexaose (**25a**) 50

5.9.2 TCA-protected Lacto-*N*-neohexaose (**25b**) 53
5.9.3 Cbz-protected Lacto-*N*-neohexaose (**25c**) 56
5.10 Protected Difucosyllacto-*N*-neohexaose (**26**) 59
5.11 Protected *iso*-Lacto-*N*-decaose (**27**) 62
5.12 Protected Fucosyllacto-*N*-neohexaose II (**28**) 65
5.13 Protected Lacto-*N*-difuco-hexoase II (**29**) 68
5.14 Protected Lacto-*N*-fucopentaose I (**30**) 71
5.15 Protected Lacto-*N*-difuco-hexoase I (**31**) 74
6. Optimization of *p*NZ group deprotection 77
6.1 Oligo-LacNAc: Tetramer **32** 85
6.2 Oligo-LacNAc: Hexamer **33** 88
6.3 Oligo-LacNAc: Octamer **34** 91
6.4 Lacto-N-neotetraose **LNnT 35** 94
6.5 *para*-Lacto-*N*-neohexaose ***p*LNnH 36** 97
6.6 Lacto-*N*-tetraose **LNT 37** 100
6.7 Lacto-*N*-fucopentaose III **LNFP III 38** 103
6.8 Lacto-*N*-fucopentaose II **LNFP II 39** 106
6.9 Lacto-*N*-neohexaose **LNnH 40** 109
6.10 Difucosyllacto-*N*-neohexaose **DF-LNnH 41** 112
6.11 *iso*-Lacto-*N*-decaose ***i*LND 42** 115
6.12 Fucosyllacto-*N*-neohexaose II **FLNnH II 43** 118
6.13 Lacto-*N*-difuco-hexoase II **LNDFH II 44** 121
6.14 Lacto-*N*-fucopentaose I **LNFP I 45** 124
6.15 Lacto-*N*-difuco-hexoase I **LNDFH I 46** 127
7. References 129

1. General Information

All chemicals were reagent grade and used as supplied unless otherwise noted. All solvents for chemical reactions were commercially purchased in p.a. quality. If stated, residual water was first removed by passing through a solvent dispensing system (J.C. Meyer) equipped with adsorption columns, and was further dried with 4 Å molecular sieves granules following the described protocol (**Section 4.2**). For HPLC and MS spectrometry, solvents with corresponding quality grades were used. Thioglycosyl donors were purchased from GlycoUniverse GmbH & Co. KGaA or synthesized if stated. All solutions were freshly prepared and kept under argon during the automation process. All reagent lines involved were washed and primed before dispensing.

Reaction progress and identity of all compounds were determined by analytical thin-layer chromatography (TLC) for synthesized building blocks and intermediates. TLC was performed on Merck silica gel 60 F254 plates (0.25 mm). Compounds were visualized by UV irradiation (254 nm) or stained (Hanessian's Stain: 235 mL of distilled water, 12 g of ammonium molybdate, 0.5 g of ceric ammonium molybdate, and 15 mL sulfuric acid). Flash column chromatography was performed on Kieselgel 60 with 230-400 mesh (Sigma-Aldrich, St. Louis, USA). ^{1}H, ^{13}C-NMR spectra were recorded on a Bruker Ascend-400 (400 MHz), Varian 600-MR (600 MHz) and Bruker Ascend-700 (700 MHz) spectrometer. Chemical shifts (δ) are reported in parts per million (ppm) relative to the respective residual solvent peaks ($CDCl_3$: δ 7.26 in ^{1}H and 77.00 in ^{13}C; DMSO: δ 2.50 in ^{1}H and 39.52 in ^{13}C; D_2O: δ 4.79 in ^{1}H). Bidimensional and non-decoupled experiments were performed to assign identities of peaks showing relevant structural features. The following abbreviations are used to indicate peak multiplicities: br. (broad), s (singlet), d (doublet), dd (doublet of doublets), ddd (doublet of doublet of doublets), t (triplet), dt (doublet of triplets), td (triplet of doublets), m (multiplet). Coupling constants (*J*) are reported in Hertz (Hz). NMR spectra were processed using MestreNova 14.1 (MestreLab Research). Assignments were supported by COSY, HSQC and HMBC experiments. Mass spectra were obtained using a Xevo G2-XS QTof mass spectrometer (HRMS, Waters).

2. Optimization of N-*p*NZ Protected Glucosamine in AGA

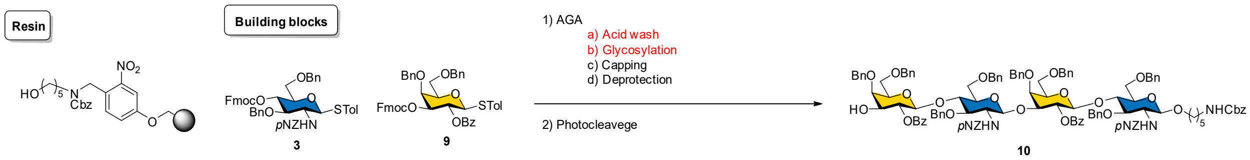

Analytical NP-HPLC of crude tetrasaccharide **10** (ELSD trace, t_R = 31.3 min)

2.1 Temperature

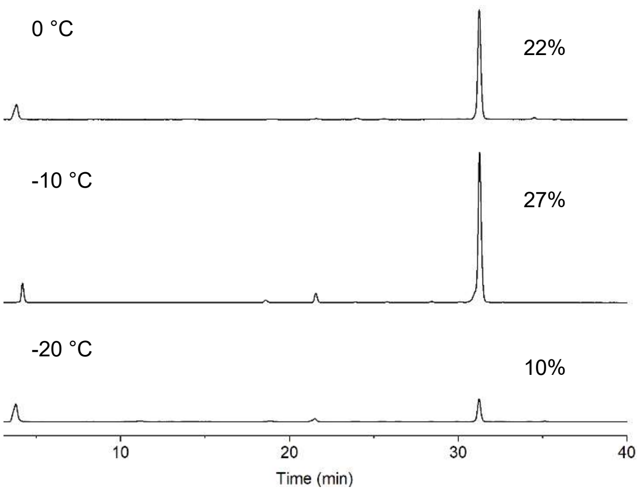

Supplementary Fig. 1 Crude HPLC spectrum under different temperatures

2.2 Pyridine washing

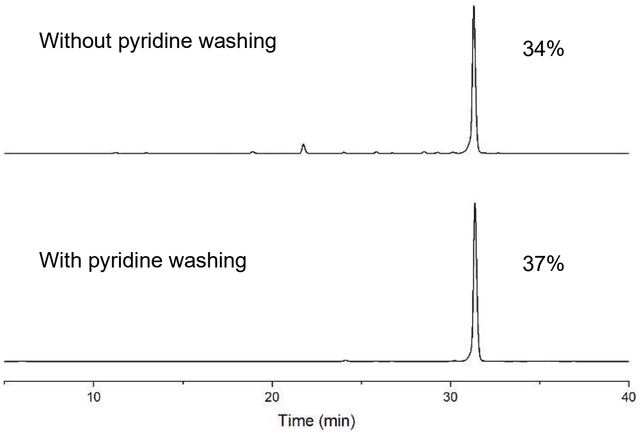

Supplementary Fig. 2 Crude HPLC spectrum with and without pyridine washing module

2.3 Comparison of *p*NZ-protected, TCA-protected and Cbz-protected glucosamine building blocks

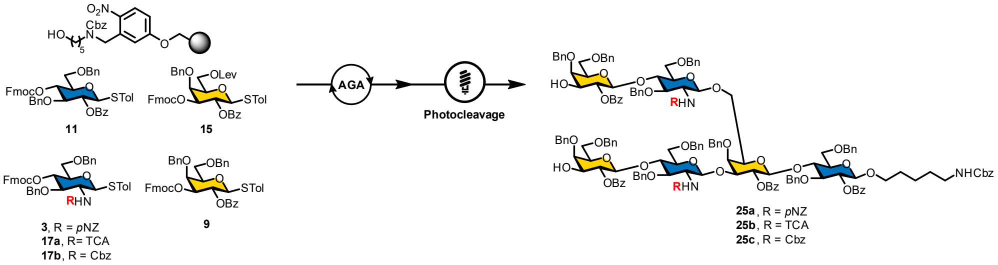

Analytical NP-HPLC of crude **25a** (ELSD trace, t_R = 27.4 min)**;** Analytical NP-HPLC of crude **25b** (ELSD trace, t_R = 30.1 min)**;** Analytical NP-HPLC of crude **25c** (ELSD trace, t_R = 37.1 min)

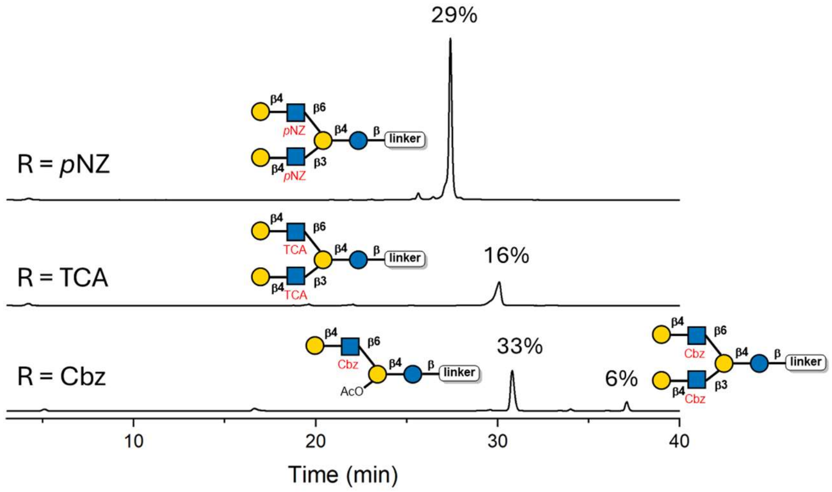

Supplementary Fig. 3 Comparison of *p*NZ-protected glucosamine **3**, TCA-protected glucosamine **17a** and Cbz-protected glucosamine **17b**.

3. Synthesis of Building Blocks

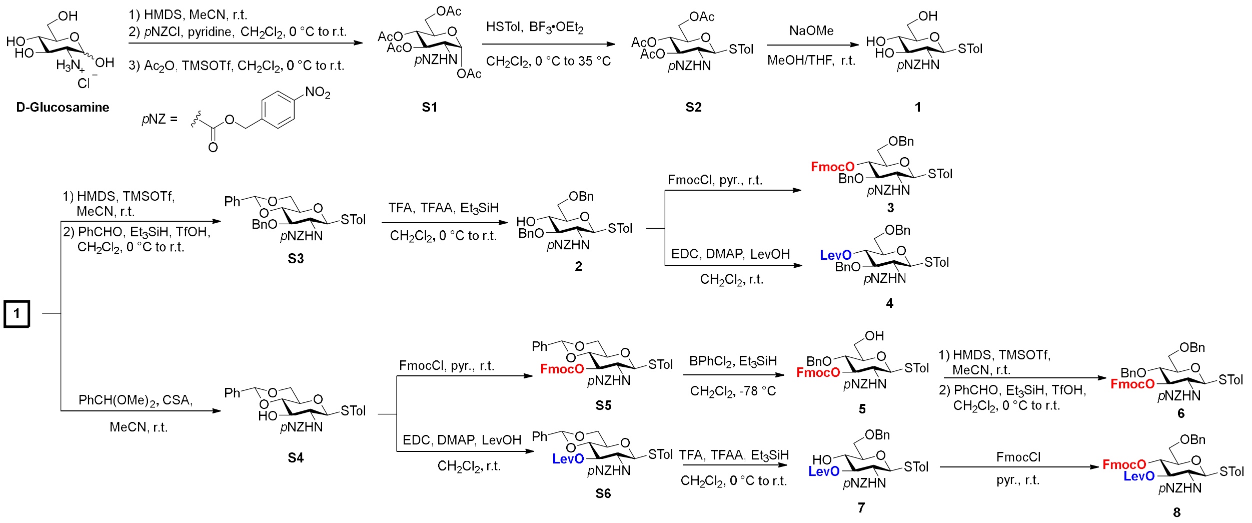

1,3,4,6-Tetra-*O*-acetyl-2-deoxy-2-(4-nitrobenzyloxycarbonylamino)-α-D-glucopyranoside (S1)[1]

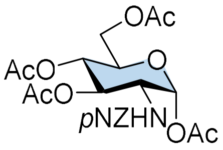

To a suspension of D-(+)-glucosamine hydrochloride (10.0 g, 46.3 mmol, 1.0 equiv.) in MeCN (200 mL), hexamethyldisilazane (HMDS, 25.1 mL, 115.9 mmol, 2.5 equiv.) was added, and the suspension was stirred at room temperature for 2 h, and monitored by TLC. After reaction completion, the mixture was filtered and washed with CH_2Cl_2. The filtered solution was concentrated under reduced pressure, and the crude product was used in the next step without further purification. The crude product was dissolved in CH_2Cl_2 (200 mL), and pyridine (5.7 mL, 69.5 mmol, 1.5 equiv.) was added at 0 °C. Then, 4-nitrobenzyl chloroformate (*p*NZCl, 1.3 g, 55.6 mmol, 1.2 equiv.) was slowly added to the stirred solution at 0 °C. The reaction was stirred at 0 °C and gradually allowed to reach room temperature overnight. The solution was concentrated under reduced pressure and filtered to remove the salt with CH_2Cl_2. The mixture was extracted with water (200 mL) and CH_2Cl_2, and the combined organic phase was dried over $MgSO_4$, filtered, and concentrated. The 1,3,4,6-tetra-*O*-trimethylsily-2-*N*-(4-nitrobenzyloxycarbonylamino)-D-glucopyranoside (quantitative) was obtained as a yellow syrup after purification by short column chromatography (Hex/EtOAc 5:1). 1,3,4,6-Tetra-*O*-trimethylsily-2-*N*-(4-nitrobenzyloxycarbonyl-amino)-D-glucopyranoside was dissolved in anhydrous CH_2Cl_2 (300 mL), and acetic anhydride

(Ac_2O, 21.4 mL, 222.2 mmol, 4.8 equiv.) and TMSOTf (1.7 mL, 9.3 mmol, 0.2 equiv.) were slowly added at 0 °C. The reaction was stirred at 0 °C and was allowed to gradually come to room temperature over 12 h while monitoring by TLC. Upon the reaction completion, aq. sat. $NaHCO_3$ was added to the stirred solution at 0 °C. The mixture was extracted with CH_2Cl_2 and water, and the combined organic phase was dried over $MgSO_4$, filtered, and concentrated.

^{1}H NMR (600 MHz, $CDCl_3$) δ 8.21 (d, *J* = 8.5 Hz, 2H), 7.47 (d, *J* = 8.5 Hz, 2H), 6.23 (d, *J* = 3.5 Hz, 1H, **H_1**), 5.32 – 5.10 (m, 4H), 4.95 (d, *J* = 9.3 Hz, 1H, N*H*), 4.26 (dd, *J* = 12.5, 4.0 Hz, 1H), 4.18 (td, *J* = 10.2, 3.5 Hz, 1H), 4.12 – 3.98 (m, 2H), 2.19 (s, 3H), 2.08 (s, 3H), 2.04 (s, 3H), 1.98 (s, 3H).
^{13}C NMR (151 MHz, $CDCl_3$) δ 171.3, 170.6, 169.1, 168.6, 155.1, 147.8, 143.2, 128.2, 123.8, 90.6 (**C_1**), 77.2, 77.0, 76.8, 70.7, 69.8, 67.5, 65.7, 61.5, 53.2, 20.9, 20.6, 20.6, 20.5.
HRMS (QToF): Calcd for $C_{22}H_{26}N_2O_{13}Na$ $[M + Na]^+$ 549.1327; found 549.1335.

^{1}H NMR (600 MHz, $CDCl_3$) of **S1**:

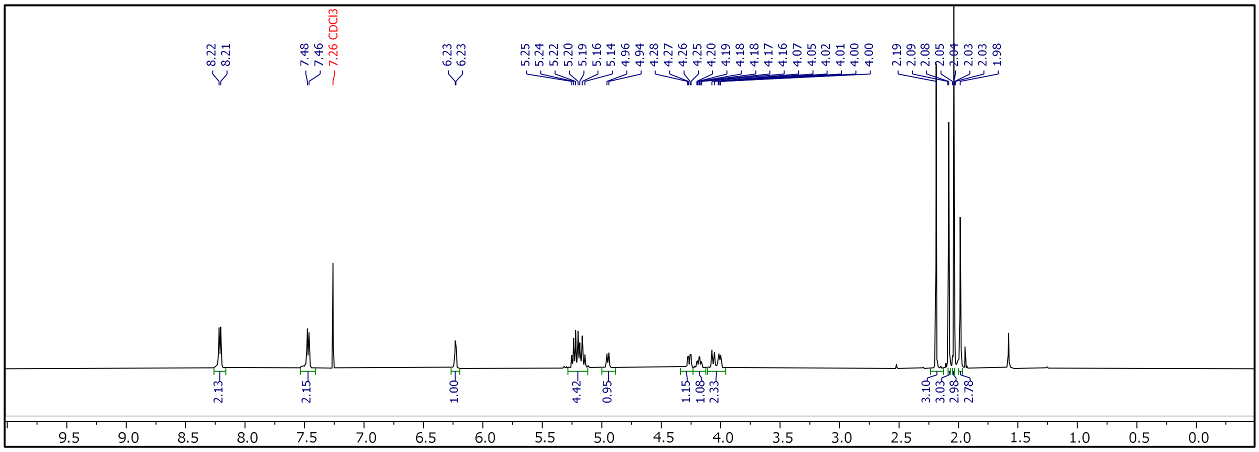

^{13}C NMR (151 MHz, $CDCl_3$) of **S1**:

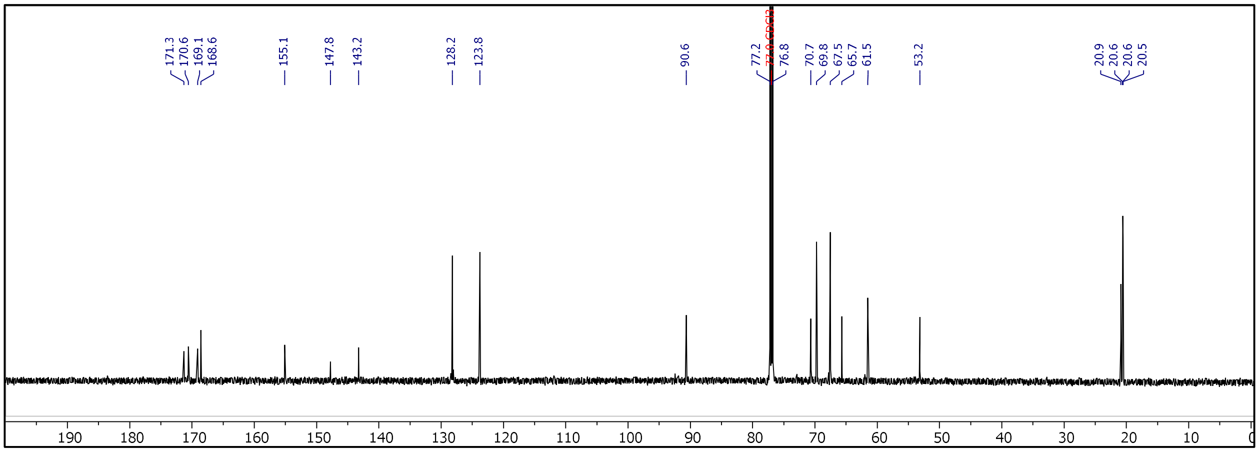

***p*-Methylphenyl 3,4,6-Tri-*O*-acetyl-2-deoxy-2-(4-nitrobenzyloxycarbonylamino)-1-thio-β-D-glucopyranoside (S2)**

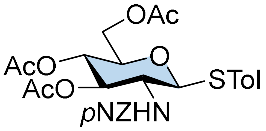

1,3,4,6-Tetra-*O*-acetyl-2-*N*-(4-nitrobenzyloxycarbonylamino)-D-glucopyranoside (crude, *ca.* 46.3 mmol, 1.0 equiv.) was used in the next step without further purification. To a mixture of the crude product and 4-methoxybenzenethiol (TolSH, 11.7 g, 92.6 mmol, 2.0 equiv.) in CH_2Cl_2 (400 mL) was added $BF_3{\cdot}OEt_2$ (17.5 mL, 138.9 mmol, 3.0 equiv.) under an argon atmosphere at 0 °C. The temperature was raised gradually to 35 °C over 12 h. After the reaction completion, as judged by TLC, the mixture was quenched with aq. sat. $NaHCO_3$ and was was extracted with CH_2Cl_2 and washed with brine. The organic phase was dried by $MgSO_4$, filtered, and concentrated. Compound **S2** (23.23 g, 39.36 mmol, 85%) was obtained as a white solid after washing with hot EtOH.

^{1}H NMR (600 MHz, $CDCl_3$) δ 8.21 (d, *J* = 8.8 Hz, 2H), 7.51 (d, *J* = 8.8 Hz, 2H), 7.37 (d, *J* = 8.1 Hz, 2H), 7.10 (d, *J* = 7.9 Hz, 2H), 5.29 – 5.18 (m, 3H), 5.01 (t, *J* = 9.7 Hz, 2H, N*H*), 4.84 (d, *J* = 9.3 Hz, 1H, **H_1**), 4.22 (dd, *J* = 12.2, 5.4 Hz, 1H), 4.15 (dd, *J* = 12.2, 2.3 Hz, 1H), 3.78 – 3.64 (m, 1H), 3.65 – 3.52 (m, 1H), 2.34 (s, 3H), 2.07 (s, 3H), 2.01 (s, 3H), 1.96 (s, 3H).
^{13}C NMR (151 MHz, $CDCl_3$) δ 170.5, 169.4, 155.0, 147.7, 138.7, 133.4, 129.8, 128.0, 123.7, 86.6 (**C_1**), 77.2, 77.0, 76.8, 75.8, 73.3, 68.5, 65.4, 62.3, 55.2, 21.1, 20.7, 20.6, 20.6.
HRMS (QToF): Calcd for $C_{27}H_{30}N_2O_{11}SNa$ $[M + Na]^+$ 613.1462; found 613.1485.

^{1}H NMR (600 MHz, $CDCl_3$) of **S2**:

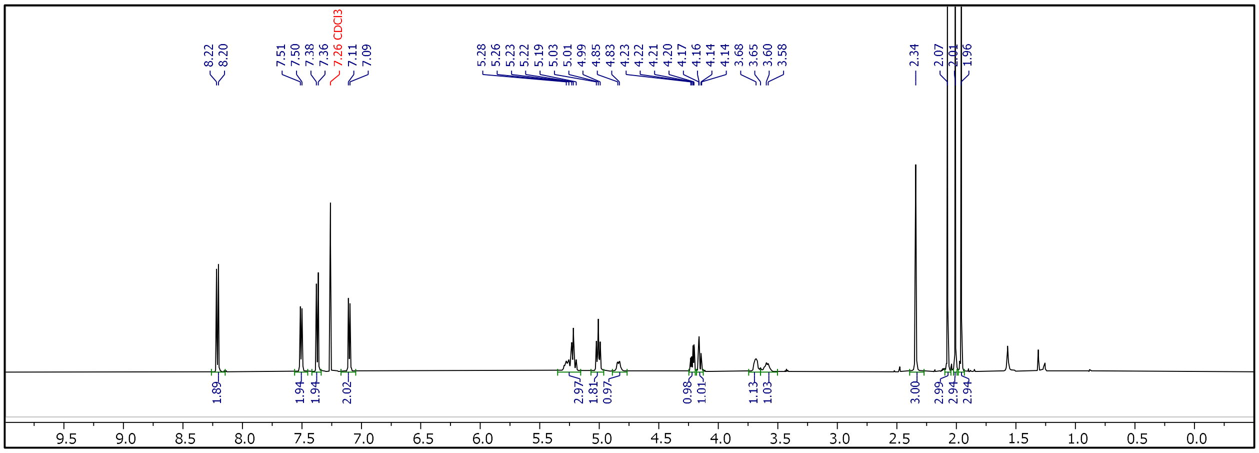

^{13}C NMR (151 MHz, $CDCl_3$) of **S2**:

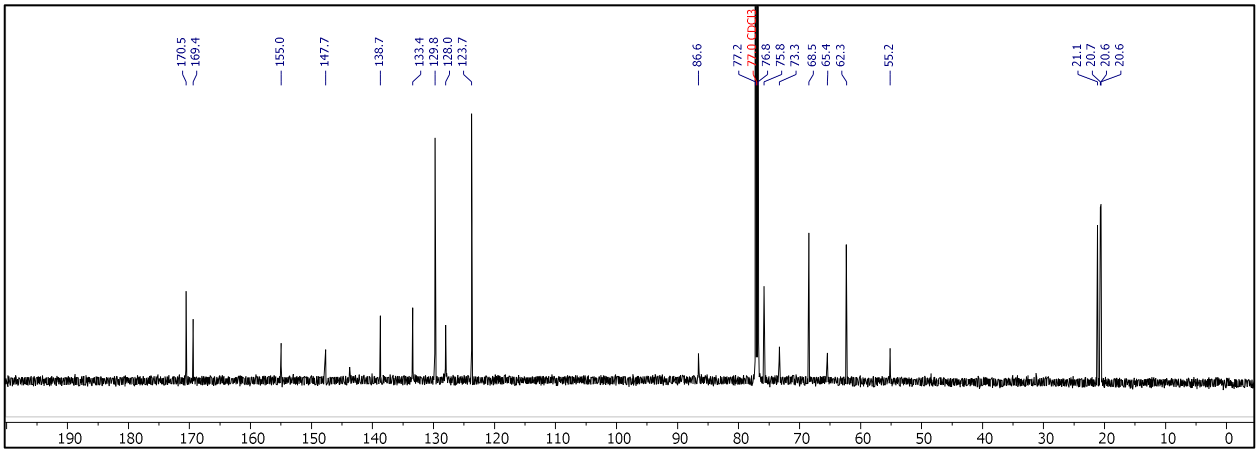

***p*-Methylphenyl 3-*O*-benzyl-4,6-*O*-benzylidene-2-deoxy-2-(4-nitrobenzyloxycarbonylamino)-1-thio-β-D-glucopyranoside (S3)**

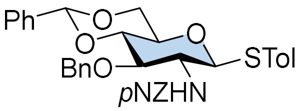

Sodium methoxide (280 mg, 5.08 mmol, 0.2 equiv.) was added at room temperature to a solution of compound **S2** (15 g, 25.40 mmol, 1.0 equiv.) in methanol (MeOH, 150 mL) and tetrahydrofuran (THF, 100 mL). The solution was stirred for 3 h and neutralized with acidic Amberlite resin IRC-120H. The resin was filtered off and washed with MeOH and THF. The filtrate was concentrated *in vacuo* to afford compound **1** (quantitative). TMSOTf (0.71 mL, 3.81 mmol, 0.15 equiv.) was added at room temperature under N_2 to a suspension of the compound **1** (11.80 g, 25.40 mmol, 1.0 equiv.) and HMDS (13.8 mL, 63.50 mmol, 2.5 equiv.) in acetonitrile (200 mL). The suspension disappeared in minutes, and the reaction was monitored by TLC. Upon reaction completion, the mixture was concentrated under reduced pressure, and the crude product was used in the next step without further purification. A solution of the crude product and benzaldehyde (PhCHO, 10.5 mL, 101.6 mmol, 4.0 equiv.) in CH_2Cl_2 was stirred at room temperature under an N_2 atmosphere for 1 h. The mixture was cooled to 0 °C, and Et_3SiH (8.28 mL, 50.8 mmol, 2.0 equiv.) and TfOH (0.34 mL, 3.81 mmol, 0.15 equiv.) were added successively. After 1 h at 0 °C, the reaction was monitored by TLC. Upon reaction completion, $NaHCO_3$ and MeOH were added successively. The crude solution was extracted with CH_2Cl_2, and the combined organic phase was dried over $MgSO_4$, filtered, and concentrated. Compound **S3** (14.2 g, 22.10 mmol, 87%) was obtained as a white solid after washing with hot EtOH.

^{1}H NMR (600 MHz, DMSO, 50 °C) δ 8.14 (d, *J* = 8.4 Hz, 2H), 7.74 (d, *J* = 9.1 Hz, 1H, N*H*), 7.59 (d, *J* = 8.2 Hz, 2H), 7.47 – 7.35 (m, 5H), 7.33 (d, *J* = 8.1 Hz, 2H), 7.22 (s, 5H), 7.15 (d, *J* = 7.7 Hz, 2H), 5.70 (s, 1H), 5.28 – 5.19 (m, 2H), 4.93 (d, *J* = 10.3 Hz, 1H, $\mathbf{H_1}$), 4.73 (d, *J* = 11.8 Hz, 1H), 4.61 (d, *J* = 11.8 Hz, 1H), 4.24 (dd, *J* = 10.1, 4.9 Hz, 1H), 3.87 – 3.69 (m, 3H), 3.58 (q, *J* = 9.5 Hz, 1H), 3.48 (dt, *J* = 9.5, 4.9 Hz, 1H), 2.29 (s, 3H).

^{13}C NMR (151 MHz, DMSO, 50 °C) δ 155.4, 146.8, 145.0, 138.4, 137.4, 136.8, 131.0, 129.5, 128.6, 127.9, 127.8, 127.0, 125.8, 124.7, 123.2, 100.0, 86.8 ($\mathbf{C_1}$), 80.4, 79.7, 73.3, 69.7, 67.5, 64.1, 55.7, 39.5, 30.3, 20.4.

HRMS (QToF): Calcd for $C_{35}H_{34}N_2O_8SNa$ $[M + Na]^+$ 665.1928; found 665.1951.

^{1}H NMR (600 MHz, DMSO) of **S3**:

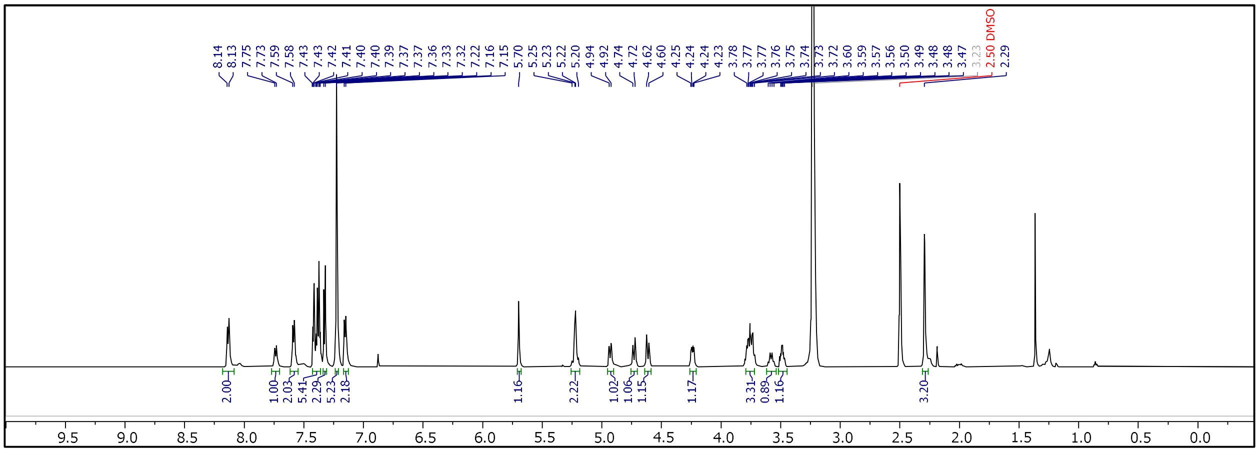

^{13}C NMR (151 MHz, DMSO) of **S3**:

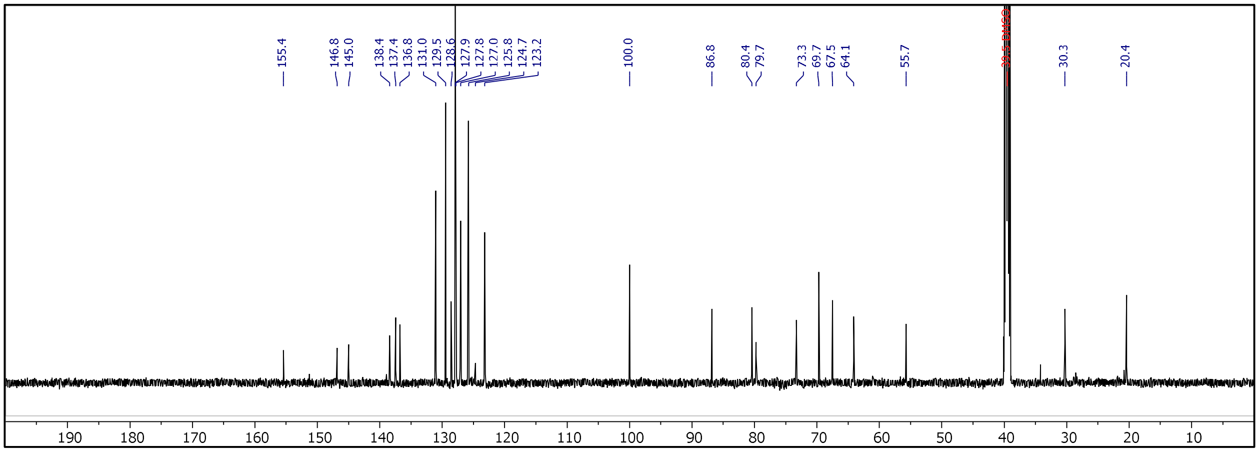

***p*-Methylphenyl 3,6-di-*O*-benzyl-2-deoxy-2-(4-nitrobenzyloxycarbonylamino)-1-thio-β-D-glucopyranoside (2)**

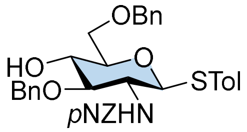

To compound **S3** (10 g, 15.56 mmol, 1.0 equiv.) in anhydrous CH_2Cl_2 (200 mL), triethylsilane (TES, 15.22 mL, 93.36 mmol, 6.0 equiv.) and trifluoroacetic anhydride (TFAA, 2.21 mL, 15.56 mmol, 1.0 equiv.) were added at 0 °C. Trifluoroacetic acid (TFA, 7.29 mL, 93.36 mmol, 6.0 equiv.) was added dropwise. The mixture was allowed to warm to room temperature and stirred for 2 h. The solution was diluted with CH_2Cl_2 and quenched with aq. sat. $NaHCO_3$. The aqueous phase was extracted twice with CH_2Cl_2, and the combined organic phase was dried over $MgSO_4$, filtered, and concentrated. Compound **2** (8.42 g, 13.07 mmol, 84%) was obtained as a white solid after washing with hot EtOH.

^{1}H NMR (600 MHz, DMSO, 50°C) δ 8.12 (d, *J* = 8.6 Hz, 2H), 7.66 (d, *J* = 8.9 Hz, 1H, N*H*), 7.57 (d, *J* = 8.6 Hz, 2H), 7.40-7.33 (m, 5H), 7.32 – 7.28 (m, 3H), 7.27 – 7.24 (m, 4H), 7.05 (d, *J* = 7.9 Hz, 2H), 5.39 (d, *J* = 6.5 Hz, 1H), 5.21 (d, *J* = 14.2 Hz, 1H), 5.20 (d, *J* = 14.2 Hz, 1H), 4.81 (d, *J* = 11.4 Hz, 1H), 4.77 (d, *J* = 9.8 Hz, 1H, **H_1**), 4.61 (d, *J* = 11.4 Hz, 1H), 4.51 (d, *J* = 12.1 Hz, 1H), 4.48 (d, *J* = 12.1 Hz, 1H), 3.80 (dd, *J* = 11.1, 1.5 Hz, 1H), 3.58 (dd, *J* = 11.1, 6.5 Hz, 1H), 3.54 – 3.46 (m, 2H), 3.45 – 3.39 (m, 1H), 3.39 – 3.33 (m, 1H), 2.25 (s, 3H).

^{13}C NMR (151 MHz, DMSO, 50°C) δ 145.0, 138.8, 138.5, 136.3, 130.6, 129.3, 127.9, 127.8, 127.7, 127.1, 127.0, 127.0, 126.9, 123.2, 86.3 (**C_1**), 79.6, 73.6, 72.2, 70.0, 69.5, 64.0, 55.4, 39.5, 20.3.
HRMS (QToF): Calcd for $C_{35}H_{36}N_2O_8SNa$ $[M + Na]^+$ 667.2084; found 667.2109.

^{1}H NMR (600 MHz, DMSO) of **2**:

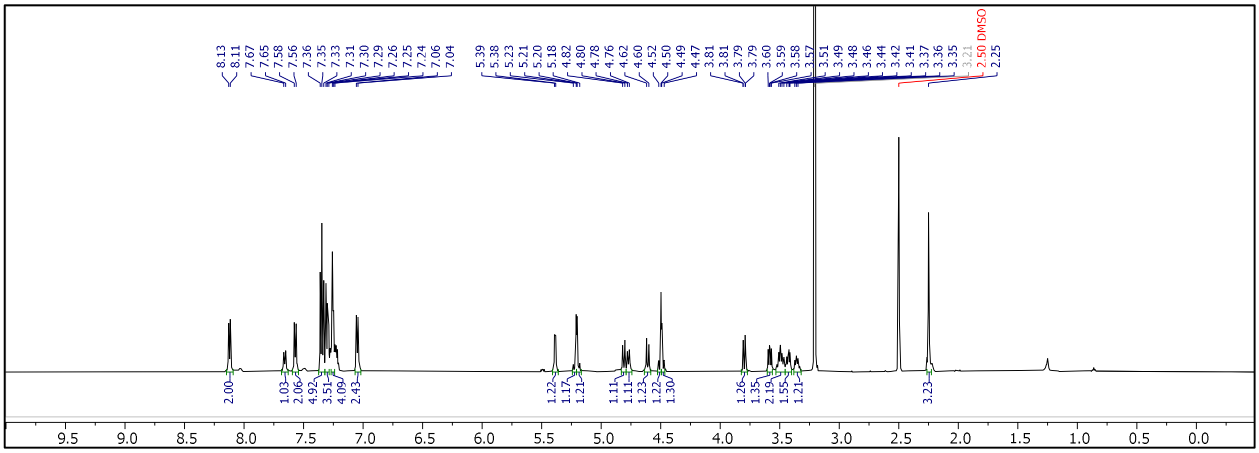

^{13}C NMR (151 MHz, DMSO) of **2**:

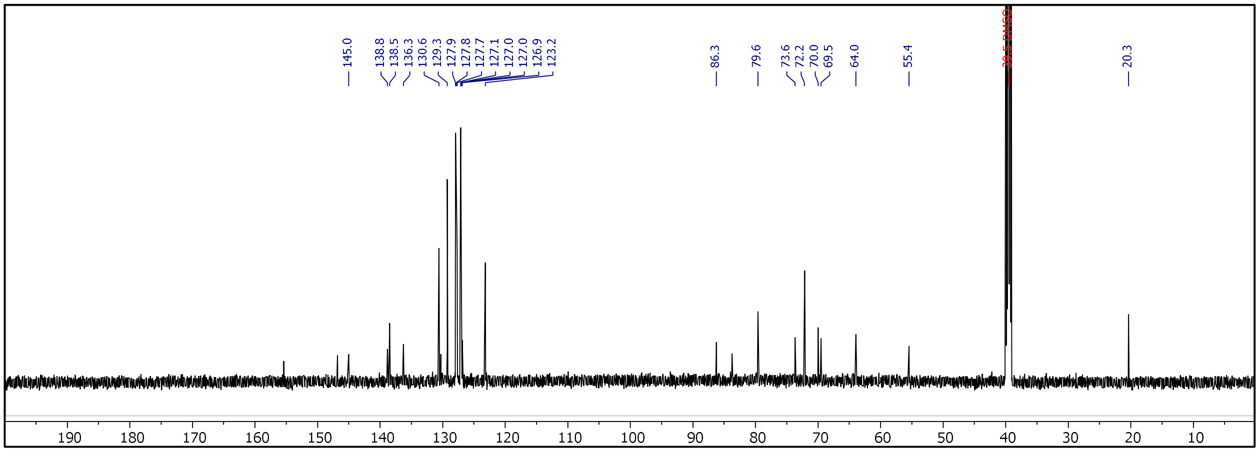

***p*-Methylphenyl 3,6-di-*O*-benzyl-2-deoxy-2-(4-nitrobenzyloxycarbonylamino)-4-*O*-fluorenylmethoxycarbonyl-1-thio-β-D-glucopyranoside (3)**

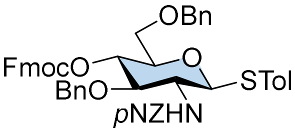

Compound **2** (8 g, 12.41 mmol, 1.0 equiv.) was dissolved in anhydrous CH_2Cl_2 (100 mL), and anhydrous pyridine (1.53 mL, 18.62 mmol, 1.5 equiv.) was added, followed by fluorenylmethyloxycarbonyl chloride (FmocCl, 4.92 g, 18.62 mmol, 1.5 equiv.) at 0 °C. The reaction mixture was stirred for 1 h at 0 °C. Sat. aq. citric acid solution (5 mL) was added, and the mixture was warmed to room temperature. The aqueous phase was extracted with CH_2Cl_2, and

the combined organic phase was dried over $MgSO_4$, filtered, and concentrated. Compound **3** (9.25 g, 10.67 mmol, 86%) was obtained as a white solid after washing with hot EtOH.

^{1}H NMR (600 MHz, DMSO, 50°C) δ 8.14 (d, *J* = 8.6 Hz, 2H), 7.87 (dd, *J* = 7.6, 3.3 Hz, 2H), 7.77 (d, *J* = 9.4 Hz, 1H, N*H*), 7.64 – 7.54 (m, 4H), 7.39 (q, *J* = 7.2 Hz, 2H), 7.35 – 7.30 (m, 4H), 7.29 – 7.24 (m, 5H), 7.21 – 7.17 (m, 3H), 7.06 (d, *J* = 7.6 Hz, 4H), 5.23 (d, *J* = 14.3 Hz, 1H), 5.20 (d, *J* = 14.3 Hz, 1H), 4.83 (d, *J* = 10.3 Hz, 1H, **H_1**), 4.64 (t, *J* = 9.6 Hz, 1H), 4.58 – 4.50 (m, 2H), 4.49 – 4.42 (m, 3H), 4.41 – 4.36 (m, 1H), 4.22 (t, *J* = 6.0 Hz, 1H), 3.76 (t, *J* = 9.6 Hz, 1H), 3.74 – 3.69 (m, 1H), 3.55 (q, *J* = 9.6 Hz, 1H), 3.50 – 3.40 (m, 2H), 2.26 (s, 3H).

^{13}C NMR (151 MHz, DMSO, 50°C) δ 153.6, 143.0, 140.6, 138.0, 136.6, 131.0, 129.3, 127.9, 127.8, 127.5, 127.2, 127.0, 126.9, 124.6, 124.5, 123.2, 119.9, 86.0, 80.6 (**C_1**), 76.0, 74.5, 72.3, 68.8, 64.1, 46.2, 39.5, 20.4.

HRMS (QToF): Calcd for $C_{50}H_{46}N_2O_{10}SNa$ $[M + Na]^+$ 889.2765; found 889.2791

^{1}H NMR (600 MHz, DMSO) of **3**:

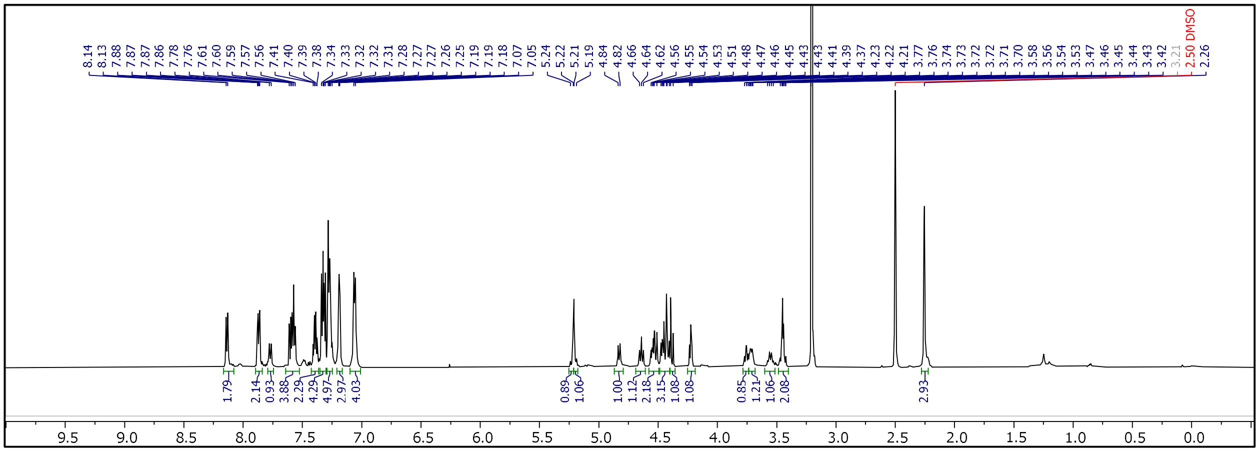

^{13}C NMR (151 MHz, DMSO) of **3**:

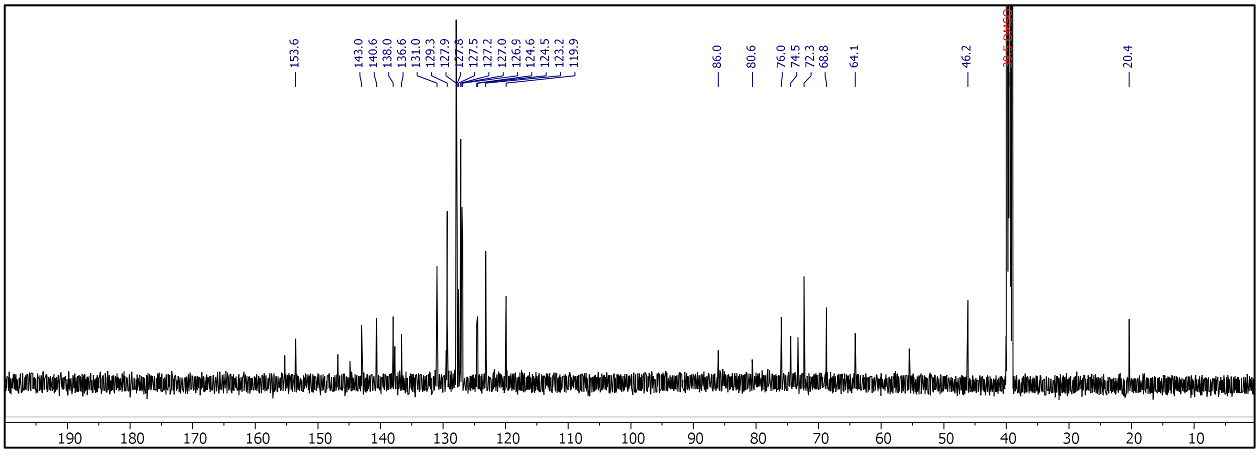

_p_-Methylphenyl 3,6-di-_O_-benzyl-2-deoxy-2-(4-nitrobenzyloxycarbonylamino)-4-_O_-levulinoyl-1-thio-β-D-glucopyranoside (4)

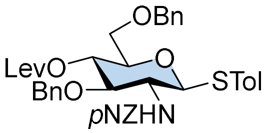

To a solution of **2** (7.0 g, 10.86 mmol, 1.0 equiv.) and levulinic acid (LevOH, 2.27 mL, 21.72 mmol, 2.0 equiv.) in anhydrous CH_2Cl_2 (100 mL) was added 1-Ethyl-3-(3-dimethylaminopropyl)carbodiimide (EDC, 5.16 g, 32.58 mmol, 3.0 equiv.) and DMAP (270 mg, 2.17 mmol, 0.2 equiv.) at 0 °C. The reaction was stirred at room temperature overnight. The reaction mixture was filtered through Celite, the filtrate was washed with aqueous $NaHCO_3$ and the aqueous phase was extracted with CH_2Cl_2. The combined organic phase was dried over $MgSO_4$, filtered and concentrated. Compound **4** (7.1 g, 9.56 mmol, 88%) was obtained as a white solid after washing with hot EtOH.

^{1}H NMR (600 MHz, DMSO, 50ºC) δ 8.15 (d, J = 8.6 Hz, 2H), 7.78 (d, J = 9.7 Hz, 1H, N*H*), 7.59 (d, J = 8.6 Hz, 2H), 7.40 – 7.32 (m, 4H), 7.32 – 7.22 (m, 6H), 7.19 – 7.15 (m, 2H), 7.06 (d, J = 7.9 Hz, 2H), 5.23 (s, 2H), 4.87 (d, J = 10.3 Hz, 1H, **H_1**), 4.82 (t, J = 9.7 Hz, 1H), 4.55 (s, 2H), 4.46 (d, J = 11.8 Hz, 1H), 4.42 (d, J = 11.8 Hz, 1H), 3.77 (t, J = 9.7 Hz, 1H), 3.68 (ddd, J = 9.7, 6.0, 2.1 Hz, 1H), 3.60 – 3.51 (m, 2H), 3.45 (dd, J = 11.2, 6.0 Hz, 1H), 2.65 (t, J = 6.5 Hz, 2H), 2.46 – 2.34 (m, 2H), 2.26 (s, 3H), 2.08 (s, 3H).

^{13}C NMR (151 MHz, DMSO, 50°C) δ 206.3, 171.1, 146.8, 144.9, 138.1, 138.0, 136.5, 130.8, 129.3, 127.9, 127.9, 127.8, 127.3, 127.2, 127.1, 123.2, 86.1(**C_1**), 76.6, 73.3, 72.3, 70.2, 68.7, 64.1, 55.6, 39.5, 37.1, 29.2, 27.5.

HRMS (QToF): Calcd for $C_{40}H_{42}N_2O_{10}SNa$ $[M + Na]^+$ 765.2452; found 765.2487.

^{1}H NMR (600 MHz, DMSO) of **4**:

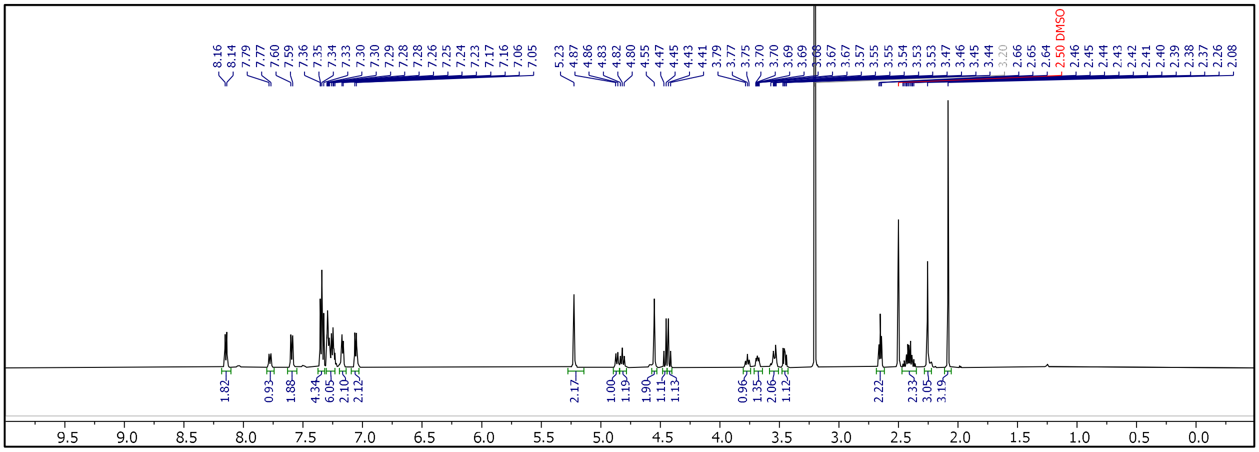

^{13}C NMR (151 MHz, DMSO) of **4**:

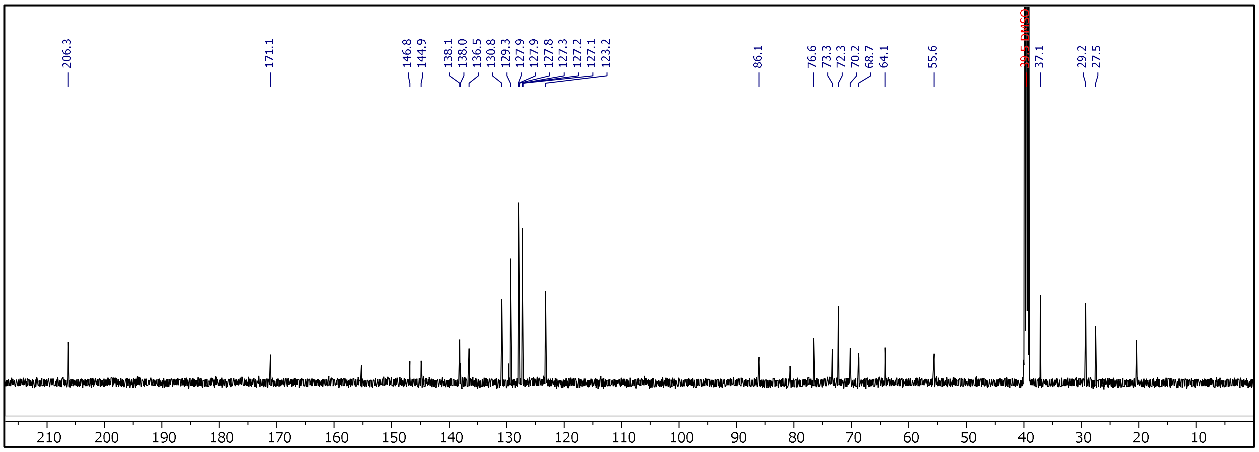

***p*-Methylphenyl 4,6-*O*-benzylidene-2-deoxy-2-(4-nitrobenzyloxycarbonylamino)-1-thio-β-D-glucopyranoside (S4)**

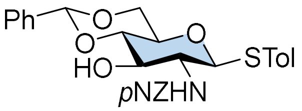

Compound **1** (16.93 mmol, 1.0 equiv.) was dissolved in MeCN (100 mL) and THF (100 mL), and benzaldehyde dimethylacetal ($PhCH(OMe)_2$, 7.78 mL, 50.79 mmol, 3.0 equiv.) and camphor sulfonic acid (CSA, 802 mg, 3.39 mmol, 0.2 equiv.) were added under an N_2 atmosphere. The reaction was stirred for 3 h or until the complete disappearance of the starting material as judged by TLC. Upon reaction completion, the mixture was extracted with CH_2Cl_2 and $NaHCO_3$, and the combined organic phase was dried over $MgSO_4$, filtered, and concentrated. Compound **S4** (7.4 g, 13.37 mmol, 79%) was obtained as a white solid after washing with hot EtOH.

^{1}H NMR (600 MHz, DMSO, 50°C) δ 8.20 (d, *J* = 8.7 Hz, 2H), 7.64 (d, *J* = 8.7 Hz, 2H), 7.53 (d, *J* = 9.4 Hz, 1H, N*H*), 7.49 – 7.42 (m, 2H), 7.41 – 7.35 (m, 3H), 7.31 (d, *J* = 8.1 Hz, 2H), 7.15 (d, *J* = 8.1 Hz, 2H), 5.61 (s, 1H), 5.42 (s, 1H), 5.26 (d, *J* = 14.1 Hz, 1H), 5.19 (d, J = 14.1 Hz, 1H), 4.89 (d, *J* = 10.4 Hz, 1H, **H_1**), 4.20 (dd, *J* = 10.4, 5.0 Hz, 1H), 3.71 (t, *J* = 10.4 Hz, 1H), 3.68 – 3.64 (m, 1H), 3.55 – 3.45 (m, 1H), 3.44 – 3.38 (m, 2H), 2.29 (s, 3H).

^{13}C NMR (151 MHz, DMSO, 50°C) δ 155.5, 146.8, 145.1, 137.5, 136.5, 130.7, 129.4, 128.6, 127.9, 127.8, 126.1, 123.2, 100.5, 87.0 (**C_1**), 80.7, 71.5, 69.9, 67.5, 64.0, 57.1, 39.5, 20.4.

HRMS (QToF): Calcd for $C_{28}H_{28}N_2O_8SNa$ $[M + Na]^+$ 575.1458; found 575.1467.

^{1}H NMR (600 MHz, DMSO) of **S4**:

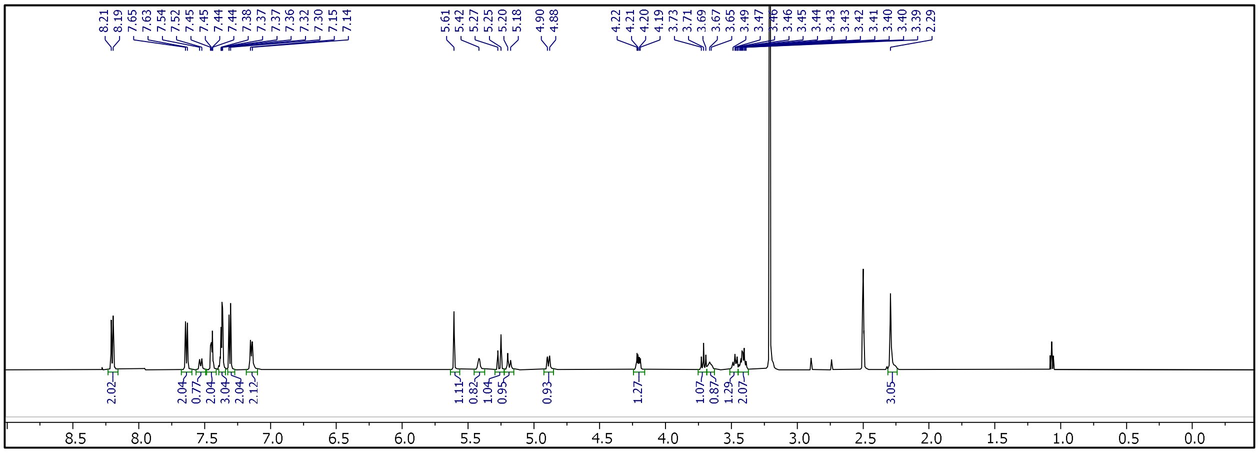

^{13}C NMR (151 MHz, $CDCl_3$) of **S4**:

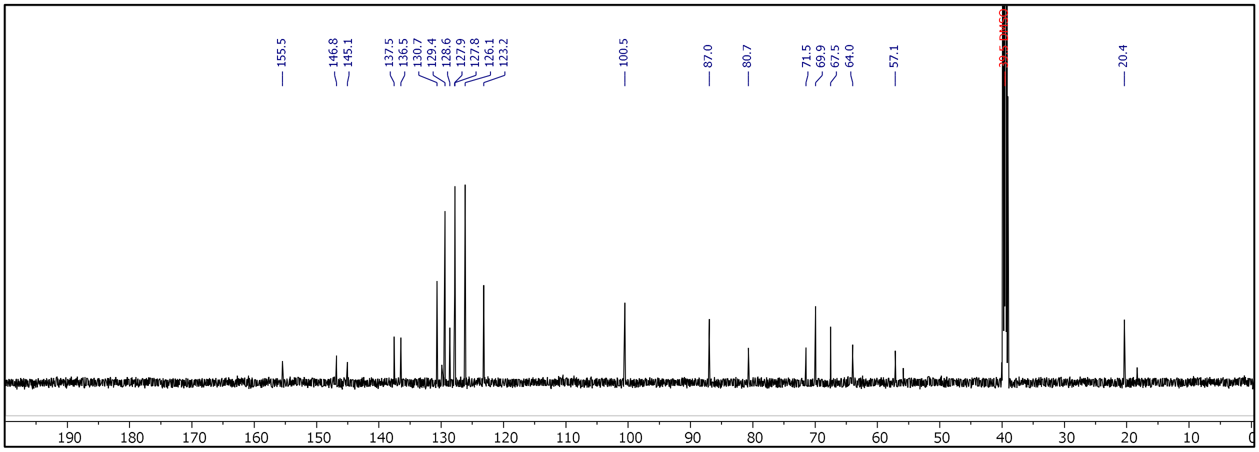

***p*-Methylphenyl 4,6-*O*-benzylidene-2-deoxy-2-(4-nitrobenzyloxycarbonylamino)-3-*O*-fluorenylmethoxycarbonyl 1-thio-β-D-glucopyranoside (S5)**

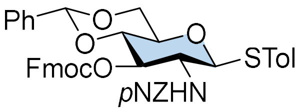

Compound **S4** (7 g, 12.67 mmol, 1.0 equiv.) was dissolved in anhydrous CH_2Cl_2 (100 mL), and anhydrous pyridine (1.56 mL, 19.00 mmol, 1.5 equiv.) was added, followed by FmocCl (5.02 g, 19.00 mmol, 1.5 equiv.) at 0 °C and stirred for 1 h. Aqueous citric acid solution was added, and the mixture was warmed to room temperature. The aqueous phase was extracted with CH_2Cl_2, and the combined organic phase was dried over $MgSO_4$, filtered, and concentrated. Compound **S5** (8.4 g, 10.77 mmol, 85%) was obtained as a white solid after washing with hot EtOH.

^{1}H NMR (400 MHz, $CDCl_3$) δ 7.93 (d, *J* = 8.3 Hz, 2H), 7.79 – 7.65 (m, 2H), 7.55 – 7.46 (m, 2H), 7.46 – 7.40 (m, 2H), 7.41 – 7.29 (m, 7H), 7.28 – 7.16 (m, 4H), 7.11 (d, *J* = 7.5 Hz, 1H), 5.54 (s, 1H), 5.38 (d, *J* = 9.0 Hz, 1H), 5.34 – 5.22 (m, 1H), 5.12 (d, *J* = 13.6 Hz, 1H), 5.01 (d, *J* = 13.8 Hz, 1H), 4.95 (d, *J* = 10.2 Hz, 1H, **H_1**), 4.44 – 4.29 (m, 2H), 4.23 (t, *J* = 9.0 Hz, 1H), 4.19 – 4.05 (m, 1H), 3.79 (q, *J* = 10.0 Hz, 3H), 3.60 – 3.54 (m, 1H), 2.35 (s, 3H).

^{13}C NMR (151 MHz, $CDCl_3$) δ 155.3, 147.5, 143.5, 143.2, 142.9, 141.3, 141.2, 138.8, 136.9, 133.3, 130.0, 129.3, 128.4, 128.2, 128.1, 127.8, 127.4, 127.3, 126.3, 125.2, 125.2, 123.6, 120.2, 101.7, 87.7 (**C_1**), 78.5, 77.2, 76.6, 70.7, 70.6, 68.6, 65.5, 56.0, 46.5, 29.8, 21.3.

HRMS (QToF): Calcd for $C_{43}H_{38}N_2O_{10}SNa$ $[M + Na]^+$ 797.2139; found 797.2166.

^{1}H NMR (400 MHz, $CDCl_3$) of **S5**:

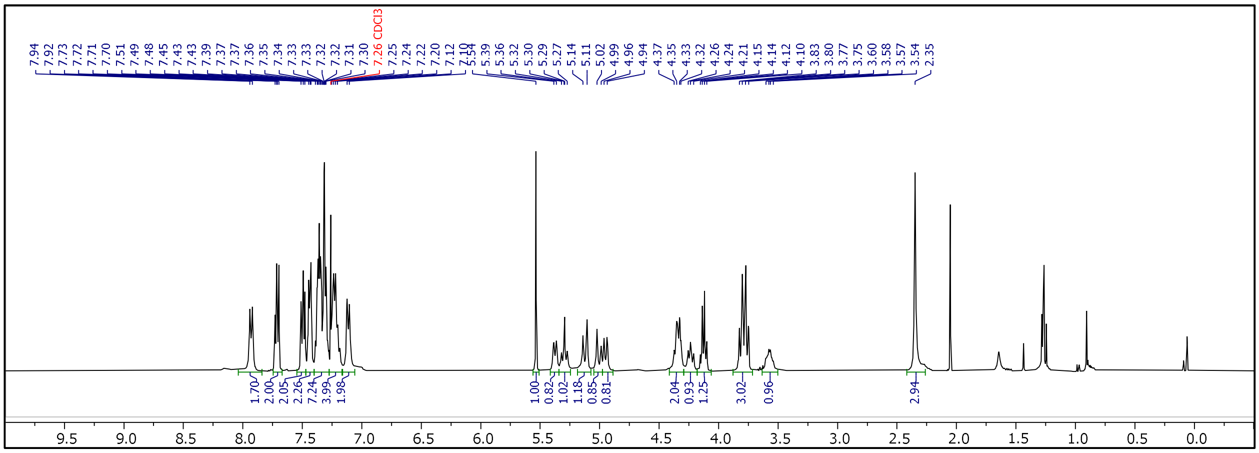

^{13}C NMR (151 MHz, $CDCl_3$) of **S5**:

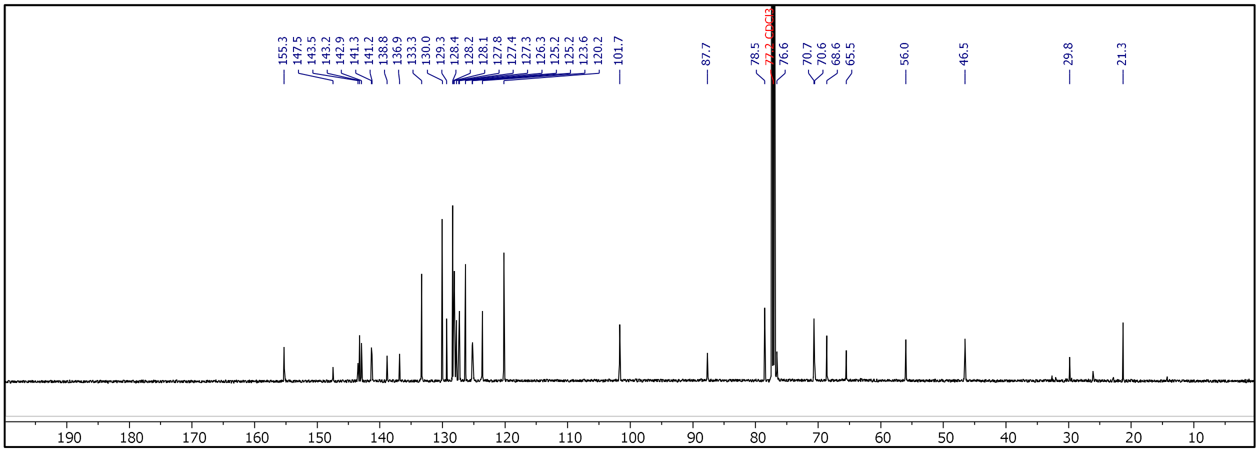

***p*-Methylphenyl 4-*O*-benzyl-2-deoxy-2-(4-nitrobenzyloxycarbonylamino)-3-*O*-fluorenylmethoxycarbonyl-1-thio-β-D-glucopyranoside (5)**

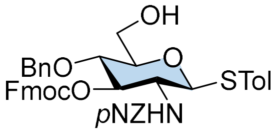

Compound **S5** (5 g, 6.45 mmol, 1.0 equiv.) in CH_2Cl_2 with molecular sieves (4Å, 500 mg) was stirred at room temperature under N_2 atmosphere for 30 min. The mixture was cooled to -78 °C, and then Et_3SiH (3.15 mL, 19.35 mmol, 3.0 equiv.) and dichlorophenylborane ($PhBCl_2$, 2.9 mL, 21.93 mmol, 3.4 equiv.) were added successively. After 1 h at -78 °C, $NaHCO_3$ and MeOH were added, and the solution was filtered through celite and washed with CH_2Cl_2. The filtrate was extracted with CH_2Cl_2, and the combined organic phase was dried over $MgSO_4$, filtered, and concentrated. Compound **5** (4.0 g, 5.03 mmol, 78%) was obtained as a white solid after washing with hot EtOH.

^{1}H NMR (600 MHz, DMSO, 50°C) δ 8.08 (d, *J* = 8.6 Hz, 2H), 7.87 – 7.82 (m, 2H), 7.79 (dd, *J* = 8.1, 1.4 Hz, 1H), 7.65 (d, *J* = 9.4 Hz, 1H, N*H*), 7.58 (d, *J* = 7.5 Hz, 1H), 7.53 (d, *J* = 7.5 Hz, 1H), 7.48 (d, *J* = 8.6 Hz, 2H), 7.42 – 7.31 (m, 5H), 7.29 – 7.22 (m, 4H), 7.19 (d, *J* = 7.0 Hz, 2H), 7.13 (d, *J* = 8.1 Hz, 2H), 5.17 (d, *J* = 14.1 Hz, 1H), 5.09 (d, *J* = 14.1 Hz, 1H), 5.01 (t, *J* = 9.6 Hz, 1H), 4.95 (d, *J* = 10.4 Hz, 1H, **H_1**), 4.78 (dd, *J* = 6.1, 5.1 Hz, 1H, OH), 4.58 (d, J = 11.3 Hz, 1H), 4.47 (d, J = 11.3 Hz, 1H), 4.42 (dd, *J* = 10.5, 6.7 Hz, 1H), 4.32 (dd, *J* = 10.5, 7.1 Hz, 1H), 4.17 (t, *J* = 6.7 Hz, 1H), 3.77 – 3.68 (m, 1H), 3.63 (t, *J* = 9.6 Hz, 1H), 3.61 – 3.52 (m, 2H), 3.39 (dd, *J* = 9.6, 3.3 Hz, 1H), 2.29 (s, 3H).

^{13}C NMR (151 MHz, DMSO, 50°C) δ 155.2, 154.1, 146.7, 144.7, 143.0, 142.8, 140.5, 137.8, 136.6, 133.8, 130.8, 129.7, 129.4, 127.9, 127.5, 127.4, 127.3, 127.1, 126.9, 124.7, 124.5, 123.1, 119.9, 85.6 (**C_1**), 80.3, 79.4, 75.6, 73.4, 69.0, 64.0, 60.1, 54.6, 46.0, 39.5, 20.4.

HRMS (QToF): Calcd for $C_{43}H_{40}N_2O_{10}SNa$ $[M + Na]^+$ 799.2296; found 799.2333.

^{1}H NMR (600 MHz, DMSO) of **5**:

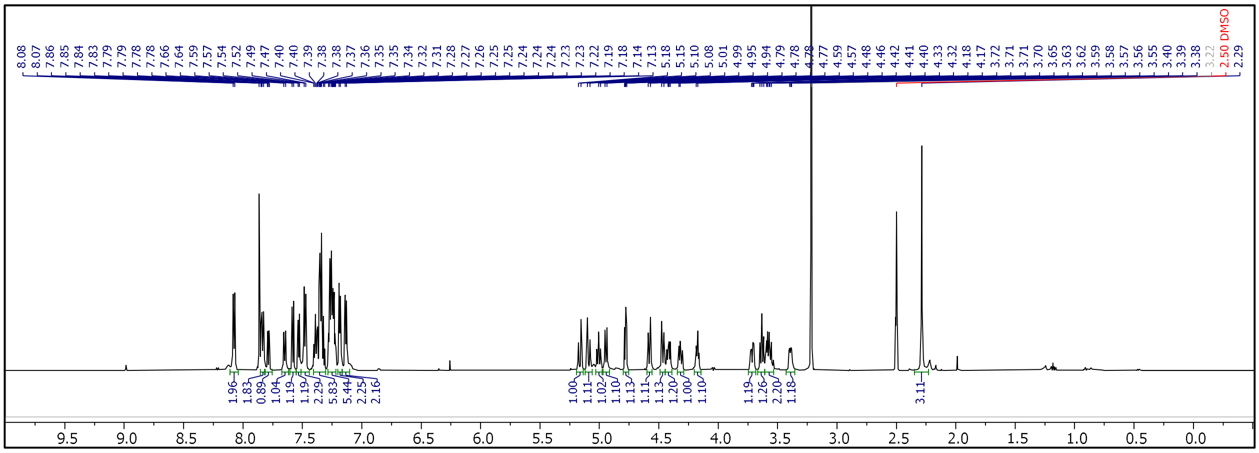

^{13}C NMR (151 MHz, DMSO) of **5**:

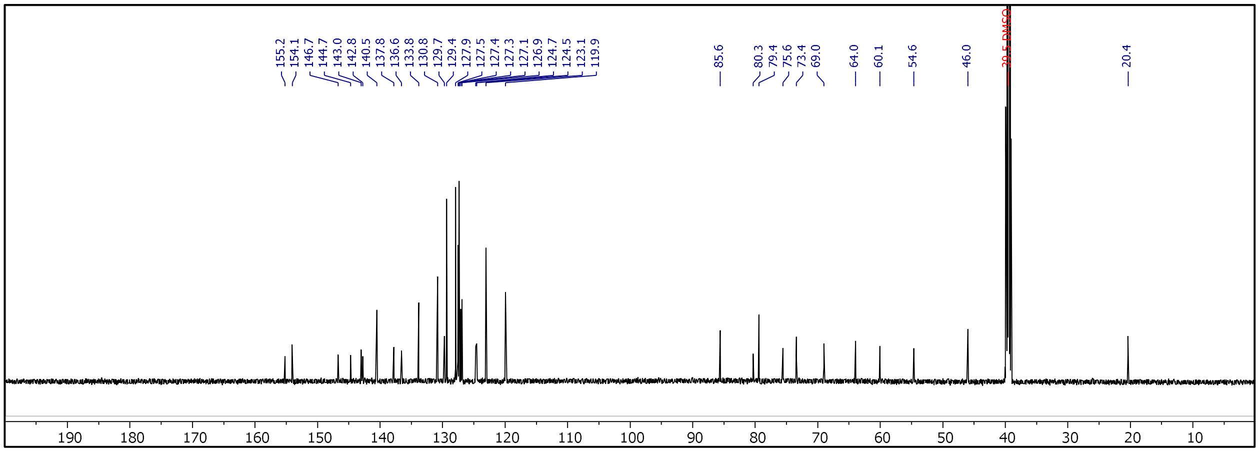

p-Methylphenyl 4,6-di-O-benzyl-2-deoxy-2-(4-nitrobenzyloxycarbonylamino)-3-O-fluorenylmethoxycarbonyl 1-thio-β-D-glucopyranoside (6)

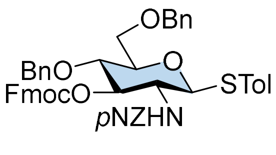

TMSOTf (0.14 mL, 0.77 mmol, 0.2 equiv.) was added at room temperature under N_2 to a suspension of the compound **5** (3 g, 3.86 mmol, 1.0 equiv.) and HMDS (0.84 mL, 3.86 mmol, 1.0 equiv.) in CH_2Cl_2 (50 mL). The suspension disappeared in minutes, and the reaction was monitored by TLC. Upon reaction completion, the mixture was concentrated under reduced pressure, and the crude product was used in the next step without further purification. A solution

of the crude product and benzaldehyde (1.59 mL, 15.44 mmol, 4.0 equiv.) in CH_2Cl_2 was stirred at room temperature under an N_2 atmosphere for 1 h. The mixture was cooled to 0 °C, and Et_3SiH (1.26 mL, 7.72 mmol, 2.0 equiv.) and TfOH (0.70 mL, 0.77 mmol, 0.2 equiv.) were added successively. After 1 h at 0 °C, the reaction was monitored by TLC. Upon reaction completion, $NaHCO_3$ (1.5 mL) and MeOH (1 mL) were added successively, and the solution was filtered through celite and washed with CH_2Cl_2. The filtrate was extracted with CH_2Cl_2, and the combined organic phase was dried over $MgSO_4$, filtered, and concentrated. Compound **6** (2.5 g, 2.86 mmol, 74%) was obtained as a white solid after washing with hot EtOH.

^{1}H NMR (600 MHz, DMSO) δ 8.08 (d, *J* = 8.7 Hz, 2H), 7.85 (dd, *J* = 14.8, 7.6 Hz, 2H), 7.78 (d, *J* = 9.5 Hz, 1H, N*H*), 7.58 (d, *J* = 7.6 Hz, 1H), 7.52 (d, *J* = 7.6 Hz, 1H), 7.46 (d, *J* = 8.7 Hz, 2H), 7.39 (t, *J* = 7.5 Hz, 1H), 7.37 – 7.29 (m, 8H), 7.28 – 7.20 (m, 5H), 7.10 (dd, *J* = 7.1, 2.1 Hz, 2H), 7.07 (d, *J* = 8.1 Hz, 2H), 5.16 (d, *J* = 14.2 Hz, 1H), 5.09 (d, *J* = 14.2 Hz, 1H), 4.97 (t, *J* = 8.7 Hz, 1H), 4.93 (d, *J* = 10.4 Hz, 1H, **H_1**), 4.52 (d, *J* = 12.0 Hz, 1H), 4.49 – 4.43 (m, 3H), 4.40 (d, *J* = 11.3 Hz, 1H), 4.33 (dd, *J* = 10.6, 7.0 Hz, 1H), 4.16 (t, *J* = 6.6 Hz, 1H), 3.69 (d, *J* = 10.3 Hz, 1H), 3.65 – 3.60 (m, 3H), 3.56 (q, *J* = 10.0 Hz, 1H), 2.25 (s, 3H).

^{13}C NMR (151 MHz, DMSO) δ 155.4, 154.2, 146.7, 144.9, 143.2, 142.9, 140.7, 138.2, 137.7, 136.9, 131.2, 129.6, 129.4, 128.2, 128.2, 127.8, 127.7, 127.6, 127.5, 127.4, 127.1, 124.9, 124.7, 123.3, 120.2, 85.4 (**C_1**), 80.3, 77.9, 75.8, 73.7, 72.3, 69.1, 68.6, 64.1, 54.6, 46.1, 39.5, 20.6

HRMS (QToF): Calcd for $C_{50}H_{46}N_2O_{10}SNa$ $[M + Na]^+$ 889.2765; found 889.2800.

^{1}H NMR (600 MHz, DMSO) of **6**:

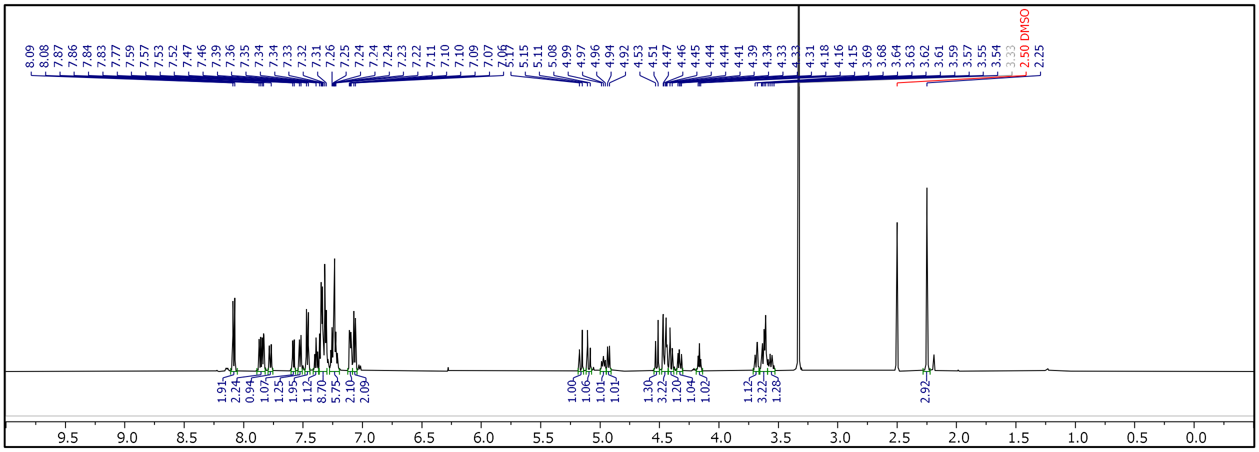

^{13}C NMR (151 MHz, DMSO) of **6**:

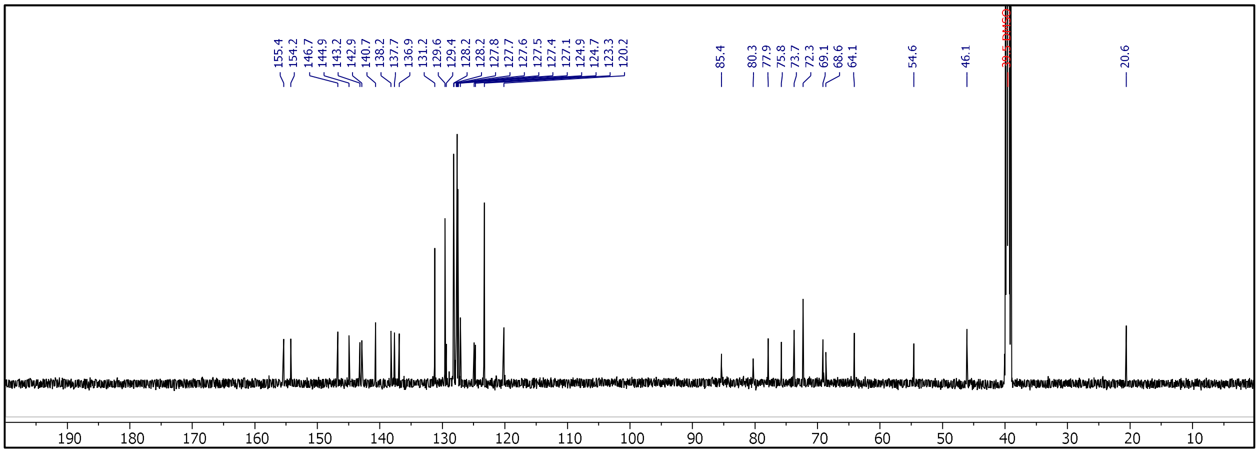

p-Methylphenyl 4,6-O-benzylidene-2-deoxy-2-(4-nitrobenzyloxycarbonylamino)-3-O-levulinoyl-1-thio-β-D-glucopyranoside (S6)

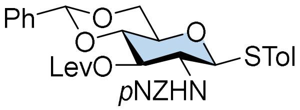

To a solution of **S4** (10.0 g, 18.10 mmol, 1.0 equiv.) and LevOH (3.78 mL, 36.19 mmol, 2.0 equiv.) in anhydrous CH_2Cl_2 (200 mL) was added EDC (8.60 g, 54.3 mmol, 3.0 equiv.) and DMAP (451 mg, 3.62 mmol, 0.2 equiv.) at 0 °C. The reaction was stirred at room temperature overnight. The reaction mixture was filtered through Celite, the filtrate was washed with aqueous $NaHCO_3$ and the aqueous phase was extracted with CH_2Cl_2. The combined organic phase was dried over $MgSO_4$, filtered and concentrated. Compound **S6** (10.4 g, 15.93 mmol, 88%) was obtained as a white solid after washing with hot EtOH.

^{1}H NMR (600 MHz, DMSO, 50°C) δ 8.21 (d, *J* = 8.6 Hz, 2H), 7.64 (d, *J* = 9.5 Hz, 1H, N*H*), 7.61 (d, *J* = 8.6 Hz, 2H), 7.42 – 7.27 (m, 7H), 7.16 (d, *J* = 7.9 Hz, 2H), 5.63 (s, 1H), 5.32 – 5.14 (m, 3H), 5.04 (d, J = 10.4 Hz, 1H, **H_1**), 4.25 (dd, *J* = 10.1, 5.0 Hz, 1H), 3.75 (q, *J* = 10.1 Hz, 1H), 3.60 (q, *J* = 9.8 Hz, 1H), 3.53 (td, *J* = 9.8, 5.0 Hz, 1H), 2.69 – 2.55 (m, 2H), 2.47 – 2.34 (m, 2H), 2.30 (s, 3H), 2.04 (s, 3H).

^{13}C NMR (151 MHz, DMSO, 50°C) δ 205.8, 171.3, 155.2, 146.9, 144.9, 137.1, 137.0, 131.3, 129.5, 128.6, 127.9, 127.8, 125.8, 123.2, 100.0, 86.1 (**C_1**), 77.7, 72.7, 69.7, 67.3, 64.2, 54.7, 39.5, 37.2, 29.1, 27.5, 20.4

HRMS (QToF): Calcd for $C_{33}H_{34}N_2O_{10}SNa$ $[M + Na]^+$ 673.1826; found 673.1851.

^1H NMR (600 MHz, DMSO) of **S6**:

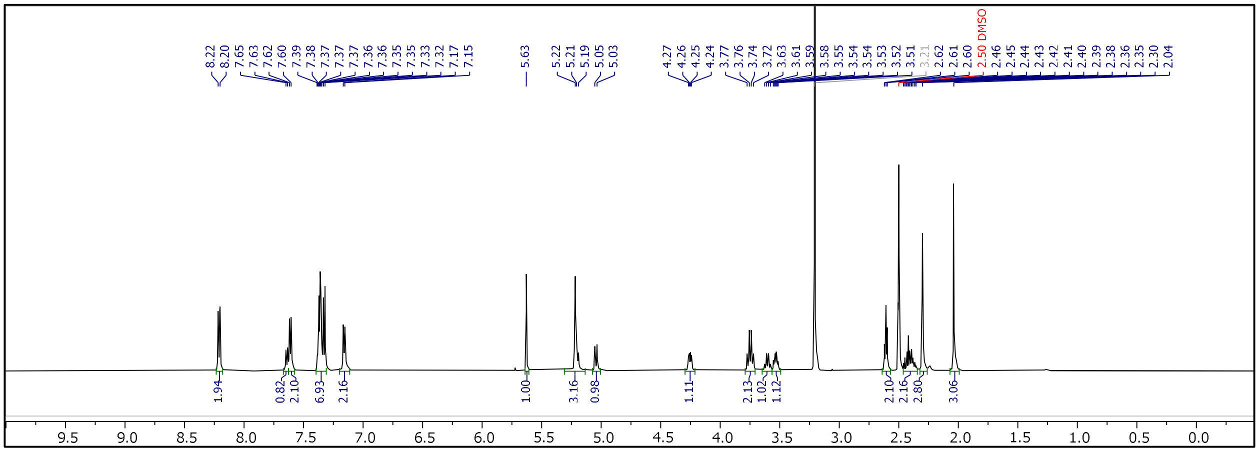

^{13}C NMR (151 MHz, DMSO) of **S6**:

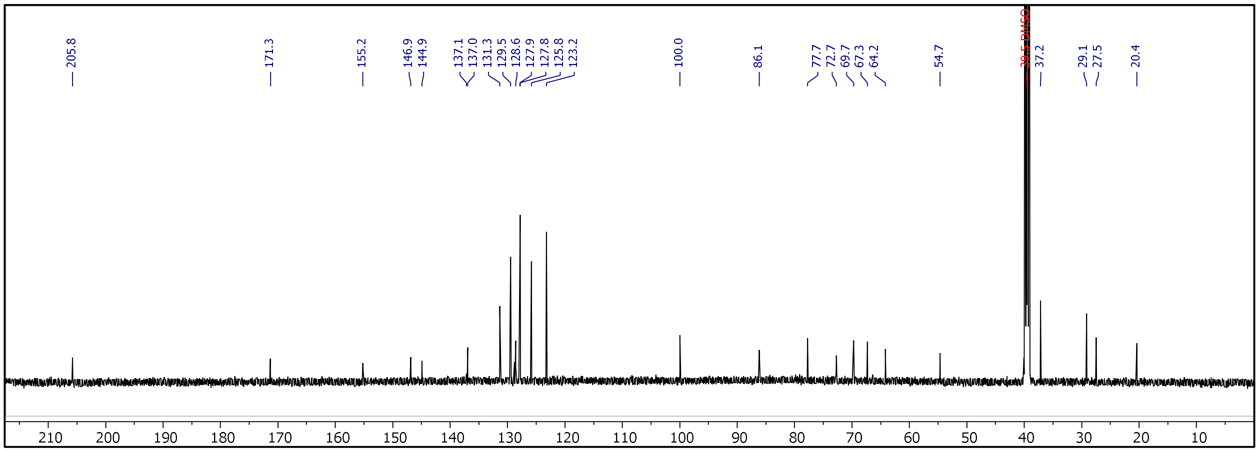

p-Methylphenyl 6-O-benzyl-2-deoxy-2-(4-nitrobenzyloxycarbonylamino)-3-O-levulinoyl-1-thio-β-D-glucopyranoside (7)

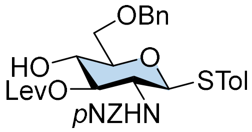

To compound **S6** (8 g, 12.29 mmol, 1.0 equiv.) in anhydrous CH_2Cl_2 (100 mL), TES (12.02 mL, 73.74 mmol, 6.0 equiv.) and TFAA (1.74 mL, 12.29 mmol, 1.0 equiv.) were added at 0 °C. Dropwise, trifluoroacetic acid (TFA, 5.76 mL, 73.74 mmol, 6.0 equiv.) was added. The mixture was allowed to warm to room temperature and stirred for 2 h. The solution was diluted with CH_2Cl_2

and quenched with aq. sat. $NaHCO_3$. The aqueous phase was extracted twice with CH_2Cl_2, and the combined organic phase was dried over $MgSO_4$, filtered, and concentrated. Compound **7** (6.65 g, 10.2 mmol, 83%) was obtained as a white solid after washing with hot EtOH.

^{1}H NMR (600 MHz, $CDCl_3$) δ 8.19 (d, *J* = 8.7 Hz, 2H), 7.52 (d, *J* = 8.7 Hz, 2H), 7.44 – 7.28 (m, 7H), 7.03 (d, *J* = 7.8 Hz, 2H), 5.25 (d, *J* = 13.7 Hz, 1H), 5.20 (d, *J* = 13.7 Hz, 1H), 5.17 (d, *J* = 9.2 Hz, 1H, N*H*), 5.08 (t, *J* = 9.6 Hz, 1H), 4.76 (d, *J* = 10.3 Hz, 1H, **H_1**), 4.57 (d, *J* = 11.9 Hz, 1H), 4.55 (d, *J* = 11.9 Hz, 1H), 3.82 (dd, *J* = 10.6, 3.7 Hz, 1H), 3.77 (dd, *J* = 10.6, 5.3 Hz, 1H), 3.70 (t, *J* = 9.2 Hz, 1H), 3.65 (q, *J* = 10.3 Hz, 1H), 3.59 – 3.53 (m, 1H), 3.26 (br., 1H, O*H*), 2.83 – 2.68 (m, 2H), 2.59 – 2.39 (m, 2H), 2.30 (s, 3H), 2.12 (s, 3H).

^{13}C NMR (151 MHz, $CDCl_3$) δ 207.6, 173.4, 155.5, 147.7, 144.2, 138.3, 138.0, 132.9, 129.9, 128.6, 128.1, 127.9, 127.8, 123.8, 87.2 (**C_1**), 78.6, 77.2, 73.8, 70.3, 70.2, 65.5, 54.8, 38.4, 29.8, 28.3, 21.3.

HRMS (QToF): Calcd for $C_{33}H_{36}N_2O_{10}SNa$ $[M + Na]^+$ 675.1988; found 675.2004.

^{1}H NMR (600 MHz, $CDCl_3$) of **7**:

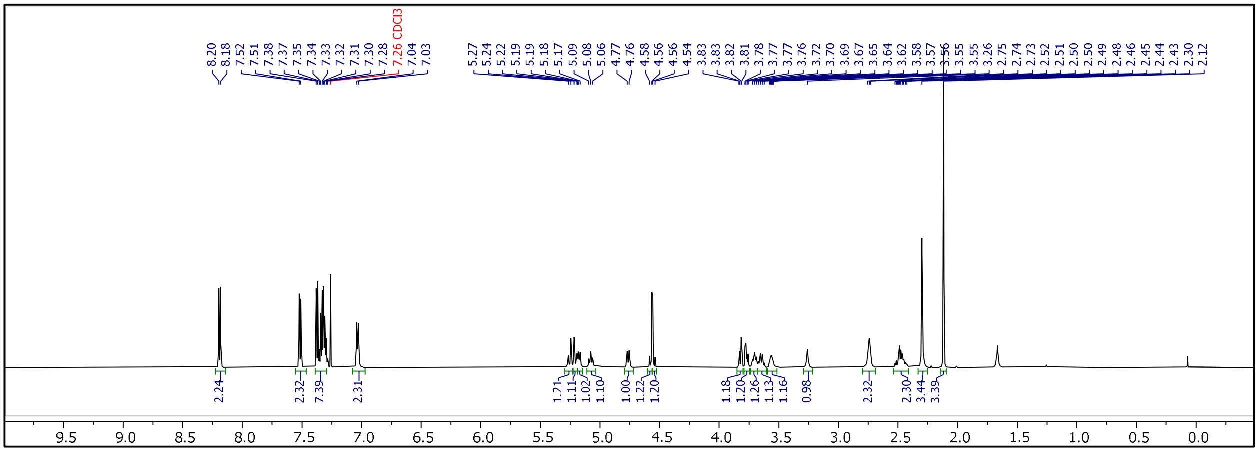

^{13}C NMR (151 MHz, $CDCl_3$) of **7**:

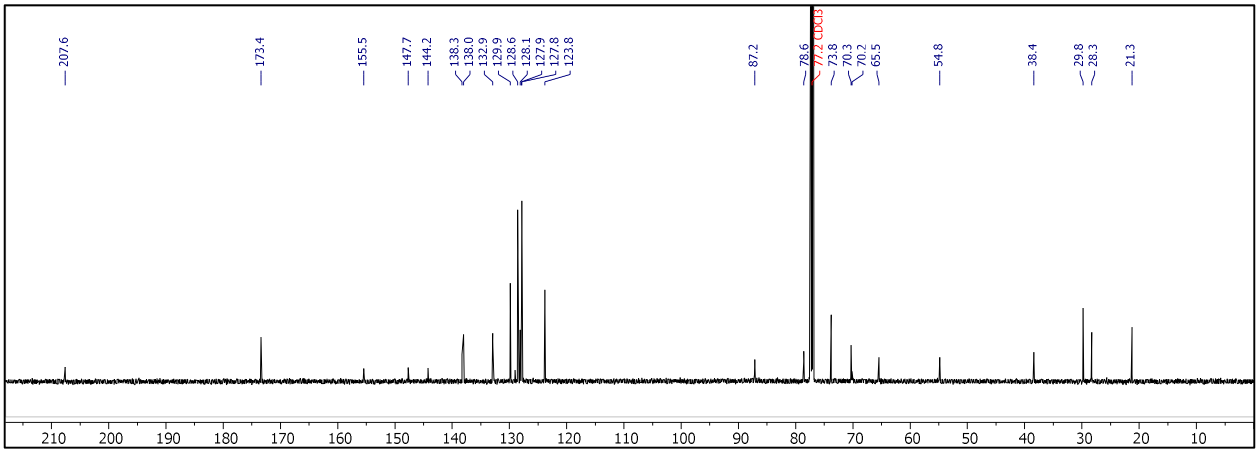

***p*-Methylphenyl 6-*O*-benzyl-2-deoxy-2-(4-nitrobenzyloxycarbonylamino)-4-*O*-fluorenylmethoxycarbonyl-3-*O*-levulinoyl-1-thio-β-D-glucopyranoside (8)**

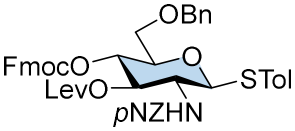

Compound **7** (7 g, 10.72 mmol, 1.0 equiv.) was dissolved in anhydrous CH_2Cl_2 (100 mL), and anhydrous pyridine (1.32 mL, 16.08 mmol, 1.5 equiv.) was added, followed by FmocCl (4.2 g, 16.08 mmol, 1.5 equiv.) at 0 °C and stirred for 1 h. Aqueous citric acid solution was added, and the mixture was warmed to room temperature. The aqueous phase was extracted with CH_2Cl_2, and the combined organic phase was dried over $MgSO_4$, filtered, and concentrated. Compound **8** (8.1 g, 9.22 mmol, 86%) was obtained as a white solid after washing with hot EtOH.

^{1}H NMR (600 MHz, $CDCl_3$) δ 8.21 (d, *J* = 8.6 Hz, 2H), 7.76 (dd, *J* = 7.5, 3.3 Hz, 2H), 7.60 – 7.47 (m, 4H), 7.47 – 7.36 (m, 4H), 7.34 – 7.28 (m, 6H), 7.25 – 7.22 (m, 1H), 7.04 (d, *J* = 7.8 Hz, 2H), 5.42 (t, *J* = 9.7 Hz, 1H), 5.28 (d, *J* = 13.7 Hz, 1H), 5.21 (d, *J* = 13.7 Hz, 1H), 5.17 (d, J = 8.6 Hz, 1H, N*H*), 4.96 (d, *J* = 10.3 Hz, 1H, **H_1**), 4.91 (t, *J* = 9.6 Hz, 1H), 4.53 (d, *J* = 11.8 Hz, 1H), 4.50 (d, *J* = 11.8 Hz, 1H), 4.39 (dd, *J* = 10.4, 7.6 Hz, 1H), 4.28 (dd, *J* = 10.4, 7.6 Hz, 1H), 4.19 (t, *J* = 7.6 Hz, 1H), 3.82 – 3.72 (m, 1H), 3.69 – 3.62 (m, 2H), 3.59 (q, *J* = 9.6 Hz, 1H), 2.75 – 2.50 (m, 2H), 2.54 – 2.33 (m, 2H), 2.31 (s, 3H), 2.01 (s, 3H).

^{13}C NMR (151 MHz, $CDCl_3$) δ 172.4, 154.3, 143.3, 141.4, 138.6, 133.3, 129.9, 128.5, 128.2, 128.1, 127.8, 127.3, 125.3, 125.3, 123.8, 120.2, 86.5 (**C_1**), 77.2, 73.7, 73.2, 70.5, 69.2, 65.6, 55.6, 46.7, 37.9, 29.7, 28.0, 21.3.

HRMS (QToF): Calcd for $C_{48}H_{46}N_2O_{12}SNa$ $[M + Na]^+$ 897.2663; found 897.2701.

^{1}H NMR (600 MHz, $CDCl_3$) of **8**:

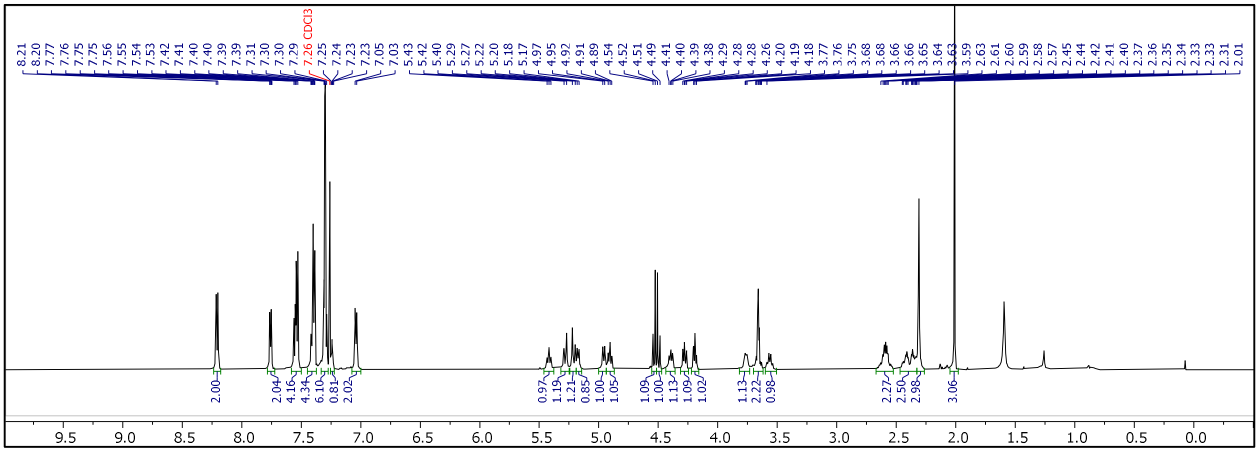

^{13}C NMR (151 MHz, $CDCl_3$) of **8**:

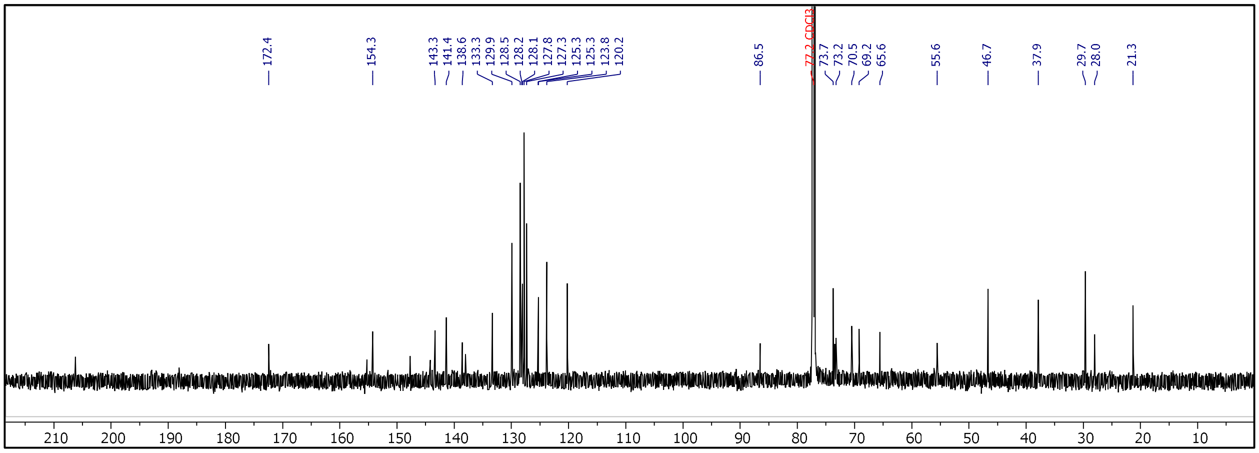

4. Materials and Conditions for Automated Synthesis

4.1 Materials and Measurements

Solvents used for dissolving all building blocks and making of various solutions were taken from Solvent Dispensing System (J.C. Meyer). Wash solvents were HPLC grade. The building blocks were purchased from GlycoUniverse GmbH & Co. KGaA or synthesized if stated. Prior to

automated synthesis, the building blocks were weighed and co-evaporated three times with anhydrous toluene and dried for overnight under high vacuum prior to use. All solutions were freshly prepared and kept under argon during the automation process. Isolated yields of products were calculated on the basis of resin loading. Functionalized resin was synthesized as previously reported and resin loading (0.33 mmol/g) was determined following a published protocol.[2] Resin was placed in the reaction vessel and was swollen in dichloromethane for 20 minutes at room temperature before starting the first module. During this time, all reagent lines involved in the synthesis were washed and primed.

4.2 Preparation of Stock Solutions

Preparation of anhydrous CH_2Cl_2 (1 ppm): The storage bottle with a capacity of 1 L was dried overnight in the oven. After 24 h, the 4Å molecular sieves were dried using a microwave at 700 W for 7 minutes, then cooled down *in vacuo*. The drying and cooling process was repeated three times. Afterward, the 4Å MS were transferred to the storage bottle and cooled down again in vacuo. Under argon, CH_2Cl_2 from the solvent dispensing system was added to the 1 L storage bottle, and the solvent was left to stand for 24 h. Finally, the water content of CH_2Cl_2 was measured using a KF-Gerat 756 coulometer.

Building Block Solution: Thioglycoside building block was dissolved in 1 mL (per cycle) of anhydrous CH_2Cl_2.

Acidic Wash Solution: TMSOTf (50 µL, 0.28 mmol) was added to 40 mL of anhydrous CH_2Cl_2.

Activator Solution: Recrystallized NIS (1.45 g, 6.45 mmol) was dissolved in 40 mL of a 4:1 mixture of anhydrous CH_2Cl_2/dioxane, followed by addition of triflic acid (55 µL, 0.62 mmol). The solution was kept under ice-bath cooling for the duration of the automated run.

Pre-capping Solution: Pyridine (10 mL) was added to 90 mL of DMF.

Capping Solution: Methanesulfonic acid (0.6 mL, 9.24 mmol), acetic anhydride (6 mL, 63.5 mmol) were added to 50 mL of anhydrous CH_2Cl_2.

Lev Deprotection Solution: N_2H_4 HOAc (725 mg, 7.87 mmol) was dissolved in 50 mL of a 4:1:0.25 mixture of pyridine/acetic acid/water.

Fmoc Deprotection Solution: Piperidine (20 mL) was added to 80 mL Dimethylformamide (DMF).

4.3 Modules for Automated Synthesis

Initiation: The resin loaded in the reaction vessel is washed with DMF, THF, and CH_2Cl_2 (3 x 3 mL for 15 s, respectively). The resin is then swollen in 2 mL CH_2Cl_2 for 20 minutes while the temperature of the reaction vessel is cooled to -20 °C.

Acidic Wash: Once the temperature of the reaction vessel has adjusted to the desired temperature of -20 °C by the cooling device, 1 mL of the **Acidic Wash Solution** is delivered to the reaction vessel. After three minutes, the solution is drained. Finally, the resin is washed with 3 mL CH_2Cl_2 (bubbling = 15 s) and drained.

Glycosylation: Upon draining the CH_2Cl_2 in the reaction vessel, 1 mL of **Building Block Solution** containing the appropriate building block is delivered from the building block storing component to the reaction vessel through. After the temperature reaches the desired temperature (T_1), **Activator Solution** (1 mL) is delivered to the reaction vessel from the respective activator storing component to the reaction vessel. The glycosylation mixture is incubated for the selected duration (t_1) at the desired T_1, then the reaction temperature is linearly ramped to T_2. Once T_2 is reached, it is maintained and the reaction mixture is incubated for an additional time (t_2). Once the incubation time is finished, the reaction mixture is drained and the resin is washed with CH_2Cl_2 (1 x 2 mL for 15 s), then dioxane (1 x 2 mL for 15 s), and finally CH_2Cl_2 (2 x 2 mL for 15 s).

Pyridine wash: The resin is washed with DMF (2 x 3 mL for 15 s). Then **Pre-capping Solution** (2 mL) is delivered into the reaction vessel and incubated for 3 minutes. The resin is then washed with CH_2Cl_2 (3 x 2 mL for 15 s).

Capping: The resin is washed with DMF (2 x 3 mL for 15 s). Then **Pre-capping Solution** (2 mL) is delivered into the reaction vessel and incubated for 3 minutes. The resin is then washed with CH_2Cl_2 (3 x 2 mL for 15 s). Upon washing, **Capping Solution** (4 mL) is delivered and the temperature is adjusted and maintained 25 °C. The resin and the reagents are incubated for 20 minutes. The solution is then drained from the reactor vessel and the resin is washed with CH_2Cl_2 (3 x 3 mL for 15 s).

Fmoc Deprotection: The resin is first washed with DMF (3 x 3 mL for 15 s), and then **Fmoc Deprotection Solution** (2 mL) is delivered to the reaction vessel. After 5 minutes the reaction solution is drained and the resin is washed with DMF (3 x 3 mL for 15 s) and CH_2Cl_2 (3 x 3 mL for 15 s). After this module the resin is ready for the next glycosylation cycle.

Lev Deprotection: The resin was washed with DMF (3 × 30 s) and DCM (1.3 mL) added to the reaction vessel. **Lev Deprotection Solution** (0.8 mL) was added to the reaction vessel, and the temperature was adjusted to 25 °C. After 30 minutes, the reaction solution was drained and the entire cycle was repeated twice more. After Lev deprotection was complete, the resin was washed with DMF, THF and DCM.

4.4 Post-synthesizer Manipulation

4.4.1 Cleavage from Solid Support: Protected Oligosaccharides

After automated synthesis, the resin was removed from the reaction vessel, suspended in CH_2Cl_2 (20 mL), and photocleaved in a continuous-flow photoreactor. A Vapourtec E-Series easy-MedCHem, equipped with a UV-150 Photochemical reactor having a UV-150 Medium-Pressure Mercury Lamp (arc length 27.9 cm, 450 W) surrounded by a long-pass UV filter (Pyrex, 50% transmittance at 305 nm) was used. A Pump 11 Elite Series (Harvard Apparatus syringe pump at a flow rate of 1.0 mL/min was used to pump the mixture through a FEP tubing (i.d. 3.0 inch, volume: 12 mL) at 20 °C. The reactor was washed with 20 mL CH_2Cl_2 at a flow rate of 2.0 mL/min. The output solution was filtered to remove the resin and the solvent was evaporated *in vacuo*.

4.4.2 General Deprotection Procedure:

Protected glycan, $B_2(OH)_4$ (22 mg), and 4,4'-bipyridine (0.1 mg) were all added to a sample vial. Then, DMF (3 mL) was added to a reaction mixture. Over 10 minutes, the reaction mixture changed color from transparent to purple and subsequently to yellow. After 15 minutes, 1 M of HCl in dioxane (50 µL) was added, and the reaction mixture was stirred at 40 °C for overnight. After the reaction, the reaction mixture was diluted with water and ethyl acetate. The organic layer was collected and washed with brine and evaporated *in vacuo*. Then, the crude compound was dissolved in Ac_2O/pyridine (4 mL, 1:1) and stirred for 3 hr. Upon the completion of acetylation, the solvent was evaporated *in vacuo*. The acetylated crude compound was dissolved in THF (5 mL) and sodium methoxide (0.5 M solution in MeOH, 0.5 mL) was added. The mixture was stirred at room temperature for 2 hours. Amberlite IR-120 (H^+ form) was then added to quench. After neutralization, the reaction mixture was filtered and the solvent was removed *in vacuo*. The crude compound was used for hydrogenolysis without further purification. The crude compound from methanolysis was dissolved in a mixture of EtOAc:^{t}BuOH:H_2O (2:1:1, 4 mL) with AcOH (0.1 mL). Pd/C (10%) was added, and the solution was purged with N_2 and H_2 for 10 minutes. The suspension was stirred under H_2 balloon for overnight. The insoluble material was removed by a

CHROMAFIL ®Xtra, RC 0.45 syringe filter. The solid was washed once with MeOH and several times with water. The filtrate was collected and concentrated *in vacuo*.

4.5 Purification

4.5.1 Analytical NP-HPLC

The crude product was dissolved in 4 mL of ethyl acetate (EtOAc) and analyzed using analytical HPLC (Agilent 1200 Series system). A YMC-Diol-300-NP column (150 mm x 4.600 mm I.D.) was used with a flow rate of 1.00 mL/min.

Method A: Hexane/EtOAc as eluent (5 minutes isocratic 20% EtOAc in hexane, 30 minutes linear gradient 20 to 60% EtOAc in hexane, 10 minutes linear gradient 60 to 100% EtOAc in hexane)

Method B: Hexane/EtOAc as eluent (5 minutes isocratic 20% EtOAc in hexane, 30 minutes linear gradient 20 to 80% EtOAc in hexane, 10 minutes linear gradient 80 to 100% EtOAc in hexane)

4.5.2 Preparative NP-HPLC

The crude products was dissolved in a 1:2 mixture of hexane and EtOAc and conducted on an Agilent 1200 Series system. A YMC-Diol-300-NP column (150 mm x 20 mm I.D.) was used with a flow rate of 15.00 mL/min.

Method C: Hexane/EtOAc as eluent (5 minutes isocratic 20% EtOAc in hexane, 30 minutes linear gradient 20 to 60% EtOAc in hexane, 10 minutes linear gradient 60 to 100% EtOAc in hexane)

Method D: Hexane/EtOAc as eluent (5 minutes isocratic 20% EtOAc in hexane, 30 minutes linear gradient 20 to 80% EtOAc in hexane, 10 minutes linear gradient 80 to 100% EtOAc in hexane)

4.5.3 Analytical RP-HPLC

Solvent was evaporated *in vacuo* and the crude products was dissolved in 3 mL of H_2O and analyzed using analytical HPLC (Agilent 1200 Series system). A Hypercarb column (150 mm x 4.6 mm I.D.) was used with a flow rate of 0.7 mL/min.

Method E: H_2O (0.1% formic acid)/ Acetonitrile (MeCN) as eluent (5 minutes isocratic 100% H_2O (0.1% formic acid), 30 minutes linear gradient 100 to 70% H_2O (0.1% formic acid) in Acetonitrile, 5 minutes linear gradient to 100% MeCN)

Method F: H_2O (0.1% formic acid)/ Acetonitrile (MeCN) as eluent (5 minutes isocratic 100% H_2O (0.1% formic acid), 30 minutes linear gradient 100 to 75% H_2O (0.1% formic acid) in Acetonitrile, 5 minutes linear gradient to 100% MeCN)

Method G: H_2O (0.1% formic acid)/ Acetonitrile (MeCN) as eluent (5 minutes isocratic 100% H_2O (0.1% formic acid), 30 minutes linear gradient 100 to 80% H_2O (0.1% formic acid) in Acetonitrile, 5 minutes linear gradient to 100% MeCN)

4.5.4 Preparative RP-HPLC

The crude products was dissolved in H_2O and conducted on an Agilent 1200 Series system. A Hypercarb column (150 mm x 10 mm I.D.) was used with a flow rate of 3.0 mL/min.

Method H: H_2O (0.1% formic acid)/ Acetonitrile (MeCN) as eluent (5 minutes isocratic 100% H_2O (0.1% formic acid), 30 minutes linear gradient 100 to 70% H_2O (0.1% formic acid) in Acetonitrile, 5 minutes linear gradient to 100% MeCN)

Method I: H_2O (0.1% formic acid)/ Acetonitrile (MeCN) as eluent (5 minutes isocratic 100% H_2O (0.1% formic acid), 30 minutes linear gradient 100 to 75% H_2O (0.1% formic acid) in Acetonitrile, 5 minutes linear gradient to 100% MeCN)

Method J: H_2O (0.1% formic acid)/ Acetonitrile (MeCN) as eluent (5 minutes isocratic 100% H_2O (0.1% formic acid), 30 minutes linear gradient 100 to 80% H_2O (0.1% formic acid) in Acetonitrile, 5 minutes linear gradient to 100% MeCN)

5. Automated Glycan Assembly of Oligo-LacNAc and Human Milk Oligosaccharides

5.1 Protected Oligo-LacNAc: Tetramer (**10**)

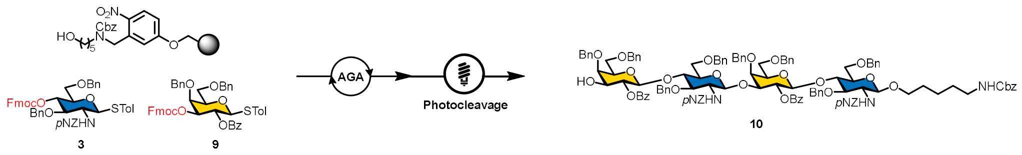

	Building blocks	Modules		Glycosylation condition
AGA		**Initiation** (40 mg resin)		
	3	Acidic wash<br>Thioglycoside glycosylation<br>Pyridine wash	x2	6.5 eq. / t (min)<br>T_1 = -10 °C / 30
		Capping<br>Fmoc deprotection		
	9	Acidic wash<br>Thioglycoside glycosylation<br>Capping<br>Fmoc deprotection		6.5 eq. / t (min)<br>T_1 = -40 °C / 5<br>T_2 = -20 °C / 20
Post AGA		Photocleavage<br>NP-HPLC Purification		

Protected **10** (10.6 mg, 4.9 µmol, 37%) was obtained as a colorless syrup by purification using preparative NP-HPLC (**Method C**).

Analytical NP-HPLC of the crude **10** (**Method A**, ELSD trace, t_R = 31.3 min)

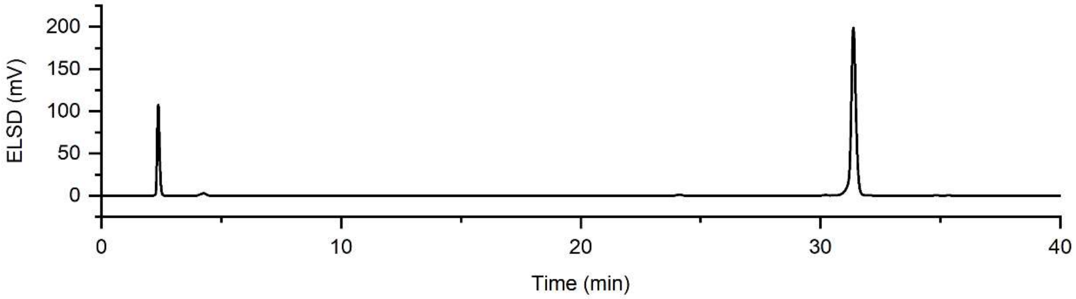

^{1}H NMR (700 MHz, $CDCl_3$) δ 8.12 – 8.03 (m, 2H), 8.02 – 7.96 (m, 4H), 7.87 (d, *J* = 7.6 Hz, 2H), 7.55 (t, *J* = 7.4 Hz, 1H), 7.52 (t, *J* = 7.3 Hz, 1H), 7.46 – 7.34 (m, 7H), 7.34 – 7.14 (m, 39H), 7.14 – 7.07 (m, 7H), 5.51 (dd, *J* = 9.9, 7.9 Hz, 1H), 5.20 (dd, *J* = 10.0, 7.9 Hz, 1H), 5.17 – 5.10 (m, 2H), 5.09 – 5.03 (m, 2H), 5.01 – 4.94 (m, 1H), 4.91 – 4.79 (m, 3H), 4.69 – 4.58 (m, 6H, **H_1**), 4.57 –

4.52 (m, 3H), 4.52 – 4.46 (m, 3H, **H₁**), 4.37 – 4.23 (m, 8H), 4.20 (d, *J* = 11.7 Hz, 1H), 4.01 (d, *J* = 3.0 Hz, 1H), 3.93 (q, *J* = 7.8 Hz, 2H), 3.89 (d, *J* = 3.7 Hz, 1H), 3.77 – 3.59 (m, 6H), 3.57 (d, *J* = 10.5 Hz, 1H), 3.53 – 3.47 (m, 3H), 3.47 – 3.42 (m, 2H), 3.42 – 3.33 (m, 3H), 3.33 – 3.28 (m, 1H), 3.27 – 3.19 (m, 2H), 3.16 – 3.05 (m, 3H), 2.25 (d, *J* = 10.3 Hz, 1H, O*H*), 1.49 – 1.35 (m, 4H), 1.33 – 1.16 (m, 2H).

^{13}C NMR (176 MHz, $CDCl_3$) δ 166.4, 156.5, 147.5, 147.4, 144.3, 144.1, 139.0, 138.7, 138.3, 138.1, 138.1, 138.0, 137.7, 136.8, 133.5, 133.4, 129.9, 129.8, 129.7, 128.7, 128.7, 128.7, 128.7, 128.6, 128.6, 128.5, 128.5, 128.5, 128.2, 128.2, 128.1, 128.1, 128.0, 128.0, 128.0, 128.0, 127.9, 127.9, 127.9, 127.8, 127.8, 127.5, 127.4, 123.8, 123.6, 100.4 (**C_1**), 100.1 (**C_1**), 77.2, 76.5, 76.2, 75.6, 75.0, 74.7, 74.6, 73.6, 73.6, 73.5, 73.5, 73.3, 72.9, 68.3, 67.7, 66.7, 65.1, 57.8, 41.0, 32.1, 29.8, 29.5, 28.9, 23.2, 22.8, 14.3.

HRMS (QToF): Calcd for $C_{123}H_{127}N_5O_{31}Na$ $[M + Na]^+$ 2192.8407; found 2192.8423.

^{1}H NMR (700 MHz, $CDCl_3$) of **10**:

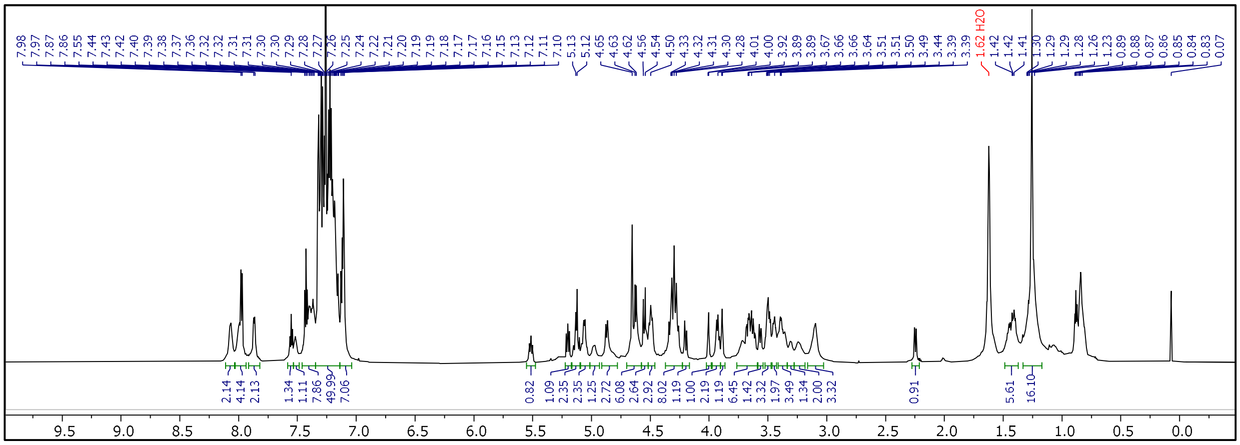

^{13}C NMR (176 MHz, $CDCl_3$) of **10**:

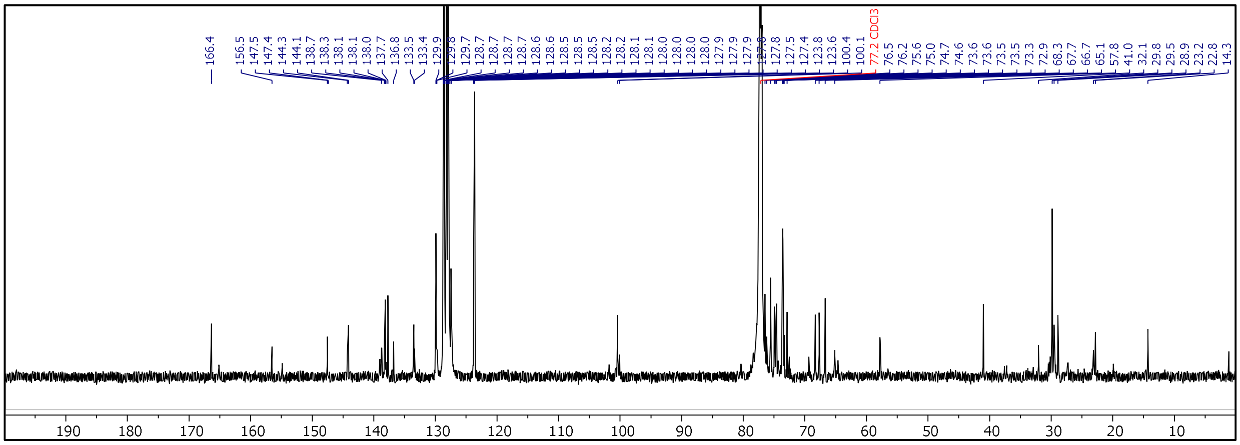

5.2 Protected Oligo-LacNAc: Hexamer (**18**)

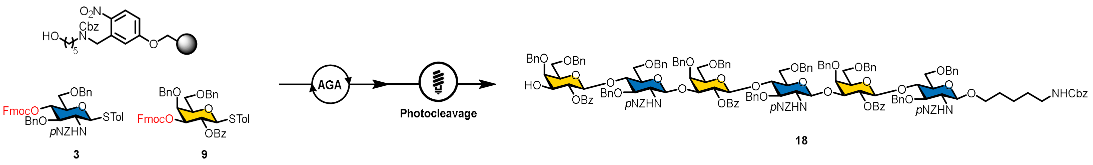

	Building blocks	Modules	Glycosylation condition
		**Initiation** (40 mg resin)	
**AGA**	**3**	**Acidic wash**<br>**Thioglycoside glycosylation**<br>**Pyridine wash** (x2: Acidic wash – Pyridine wash)<br>**Capping**<br>**Fmoc deprotection**	6.5 eq. — t (min)<br>T_1 = -10 °C — 30
**AGA**	**9**	**Acidic wash**<br>**Thioglycoside glycosylation**<br>**Capping**<br>**Fmoc deprotection**	6.5 eq. — t (min)<br>T_1 = -40 °C — 5<br>T_2 = -20 °C — 20
**Post AGA**		**Photocleavage**<br>**NP-HPLC Purification**	

Protected **18** (10.8 mg, 3.4 µmol, 26%) was obtained as a colorless syrup by purification using preparative NP-HPLC (**Method D**).

Analytical NP-HPLC of the crude **18** (**Method B**, ELSD trace, t_R = 28.2 min)

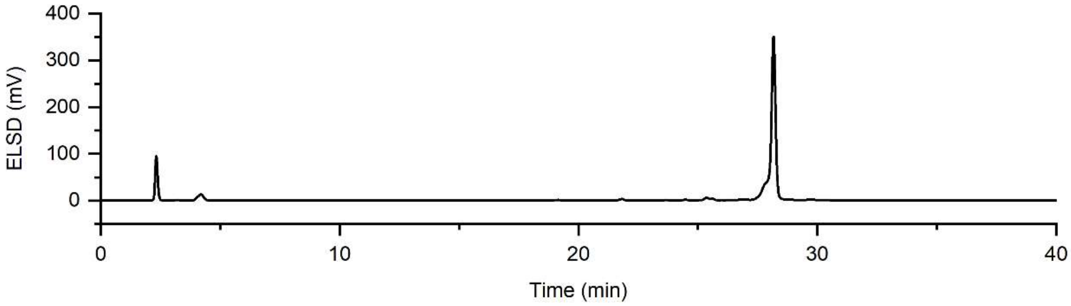

^{1}H NMR (700 MHz, CDCl$_3$) δ 8.09 – 7.94 (m, 8H), 7.92 – 7.82 (m, 4H), 7.56 (t, *J* = 7.4 Hz, 1H), 7.53 – 7.48 (m, 2H), 7.46 – 7.35 (m, 9H), 7.34 – 7.09 (m, 64H), 7.07 – 6.98 (m, 4H), 5.53 – 5.47 (m, 1H), 5.20 (dd, *J* = 9.9, 8.0 Hz, 1H), 5.17 – 5.02 (m, 4H), 5.01 – 4.92 (m, 2H), 4.88 (d, *J* = 10.9 Hz, 3H), 4.76 – 4.57 (m, 8H), 4.58 – 4.40 (m, 10H), 4.41 – 4.14 (m, 13H), 4.10 (d, *J* = 11.9 Hz, 1H), 4.07 – 3.82 (m, 6H), 3.82 – 3.54 (m, 10H), 3.53 – 3.31 (m, 14H), 3.32 – 3.17 (m, 5H), 3.15 – 3.03 (m, 3H), 2.23 (d, *J* = 10.4 Hz, 1H, O*H*), 1.51 – 1.36 (m, 4H), 1.35 – 1.17 (m, 2H).

^{13}C NMR (176 MHz, $CDCl_3$) δ 166.3, 165.1, 164.9, 156.5, 154.9, 147.5, 147.4, 144.3, 144.1, 139.07, 138.99, 138.7, 138.3, 138.2, 138.1, 138.1, 138.0, 137.7, 136.8, 133.5, 133.4, 129.9, 129.8, 128.7, 128.6, 128.6, 128.5, 128.4, 128.4, 128.2, 128.2, 128.2, 128.1, 128.1, 128.0, 128.0, 127.9, 127.7, 127.5, 127.4, 127.4, 123.8, 123.6, 101.8 (**C_1**), 100.6 (**C_1**), 100.4 (**C_1**), 100.1 (**C_1**), 80.5, 77.2, 76.5, 76.2, 75.6, 75.0, 74.9, 74.7, 74.6, 73.6, 73.6, 73.5, 73.5, 73.3, 72.9, 69.3, 68.3, 68.2, 67.7, 66.7, 65.1, 64.6, 57.8, 57.7, 41.0, 32.1, 29.8, 29.5, 28.9, 23.2, 22.8, 14.3.

HRMS (QToF): Calcd for $C_{178}H_{181}N_7O_{45}Na$ $[M + Na]^+$ 3159.1982; found 3159.2095.

^{1}H NMR (700 MHz, $CDCl_3$) of **18**:

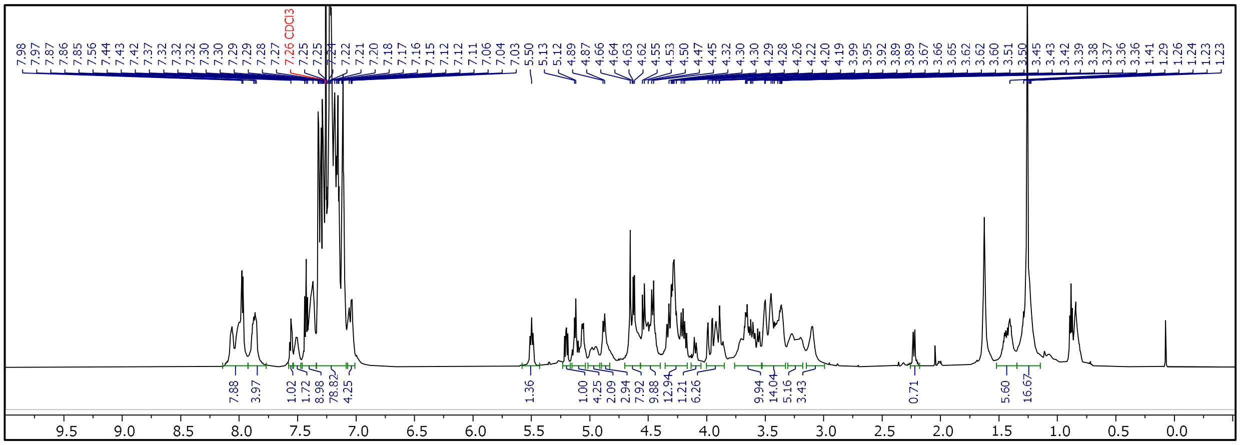

^{13}C NMR (176 MHz, $CDCl_3$) of **18**:

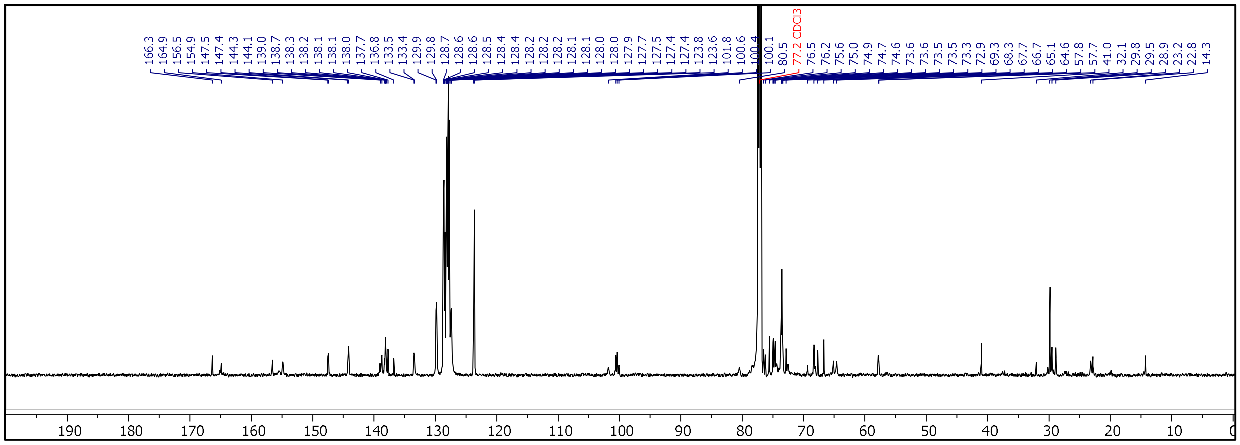

5.3 Protected Oligo-LacNAc: Octamer (**19**)

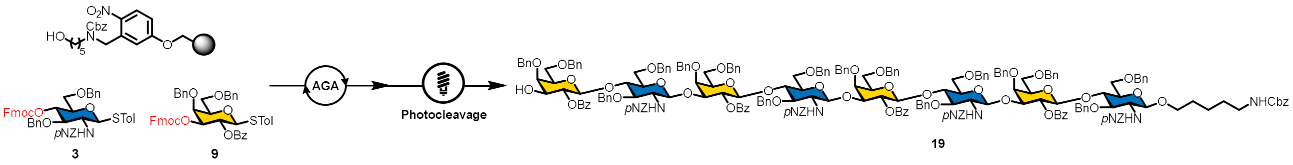

	Building blocks	Modules	Glycosylation condition
AGA		**Initiation** (40 mg resin)	
	**3**	**Acidic wash**<br>**Thioglycoside glycosylation**<br>**Pyridine wash** (**x2**: Acidic wash – Pyridine wash)<br>**Capping**<br>**Fmoc deprotection**	6.5 eq. / t (min)<br>T_1 = -10 °C / 30
	**9**	**Acidic wash**<br>**Thioglycoside glycosylation**<br>**Capping**<br>**Fmoc deprotection**	6.5 eq. / t (min)<br>T_1 = -40 °C / 5<br>T_2 = -20 °C / 20<br>Last coupling<br>6.5 eq. / t (min)<br>T_1 = -40 °C / 10<br>T_2 = -20 °C / 25
Post AGA		**Photocleavage**<br>**NP-HPLC Purification**	

Protected **19** (10.8 mg, 2.64 μmol, 20%) was obtained as a colorless syrup by purification using preparative NP-HPLC (**Method D**).

Analytical NP-HPLC of the crude **19** (**Method B,** ELSD trace, t_R = 29.3 min)

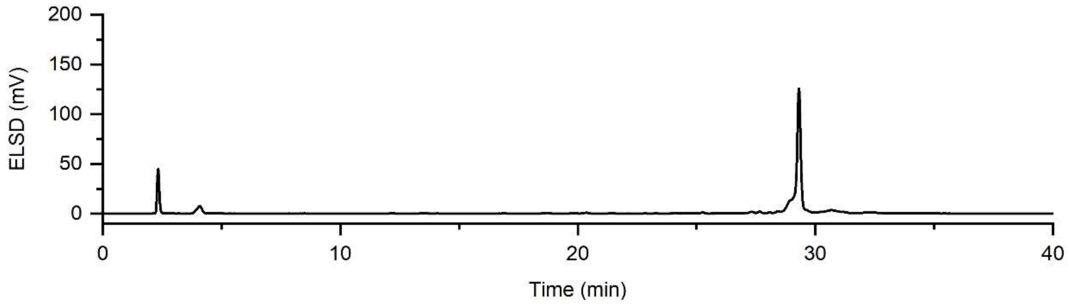

^{1}H NMR (600 MHz, $CDCl_3$) δ 8.06 (d, *J* = 8.1 Hz, 2H), 8.04 – 7.94 (m, 7H), 7.91 – 7.82 (m, 6H), 7.57 – 7.53 (m, 1H), 7.53 – 7.47 (m, 3H), 7.44 – 7.33 (m, 12H), 7.33 – 7.08 (m, 71H), 7.08 – 6.99 (m, 9H), 5.52 – 5.44 (m, 3H), 5.20 (dd, *J* = 10.0, 7.9 Hz, 1H), 5.17 – 5.09 (m, 2H), 5.09 – 5.02 (m, 2H), 5.01 – 4.91 (m, 4H), 4.91 – 4.80 (m, 5H), 4.70 – 4.55 (m, 9H, **H_1**), 4.56 – 4.38 (m, 14H, **H_1**), 4.36 – 4.23 (m, 11H), 4.19 (dt, *J* = 11.9, 7.5 Hz, 6H), 4.09 (t, *J* = 11.3 Hz, 2H), 3.99 (d, *J* = 3.0 Hz, 1H), 3.96 – 3.87 (m, 5H), 3.85 (t, *J* = 8.8 Hz, 2H), 3.74 – 3.53 (m, 11H), 3.53 – 3.47 (m, 4H), 3.47

– 3.40 (m, 6H), 3.40 – 3.31 (m, 7H), 3.30 – 3.22 (m, 4H), 3.22 – 3.15 (m, 4H), 3.10 (d, J = 6.5 Hz, 3H), 2.22 (d, J = 10.2 Hz, 1H), 1.48 – 1.36 (m, 4H), 1.31 – 1.20 (m, 2H).

^{13}C NMR (151 MHz, CDCl$_3$) δ 166.4, 165.2, 164.9, 156.5, 154.9, 147.5, 147.4, 144.3, 144.1, 139.1, 139.0, 138.8, 138.3, 138.2, 138.1, 138.0, 137.7, 136.8, 133.5, 133.4, 129.9, 129.8, 128.7, 128.7, 128.7, 128.6, 128.6, 128.6, 128.5, 128.5, 128.4, 128.4, 128.2, 128.2, 128.2, 128.1, 128.1, 128.1, 128.0, 128.0, 128.0, 127.9, 127.9, 127.9, 127.8, 127.8, 127.7, 127.7, 127.5, 127.4, 127.4, 123.8, 123.6, 123.6, 100.6 (**C_1**), 100.6 (**C_1**), 100.4 (**C_1**), 100.1 (**C_1**), 80.5, 77.2, 76.5, 76.3, 75.6, 75.0, 74.9, 74.7, 74.7, 73.7, 73.6, 73.5, 73.5, 73.4, 72.9, 69.3, 68.4, 68.3, 68.1, 67.7, 66.7, 65.2, 64.7, 57.8, 57.8, 57.7, 41.0, 30.5, 29.8, 29.5, 28.9.

HRMS (QToF): Calcd for $C_{233}H_{235}N_9O_{59}Na_2$ [M + 2Na]$^{2+}$ 2074.2725; found 2074.2805.

^{1}H NMR (600 MHz, CDCl$_3$) of **19**:

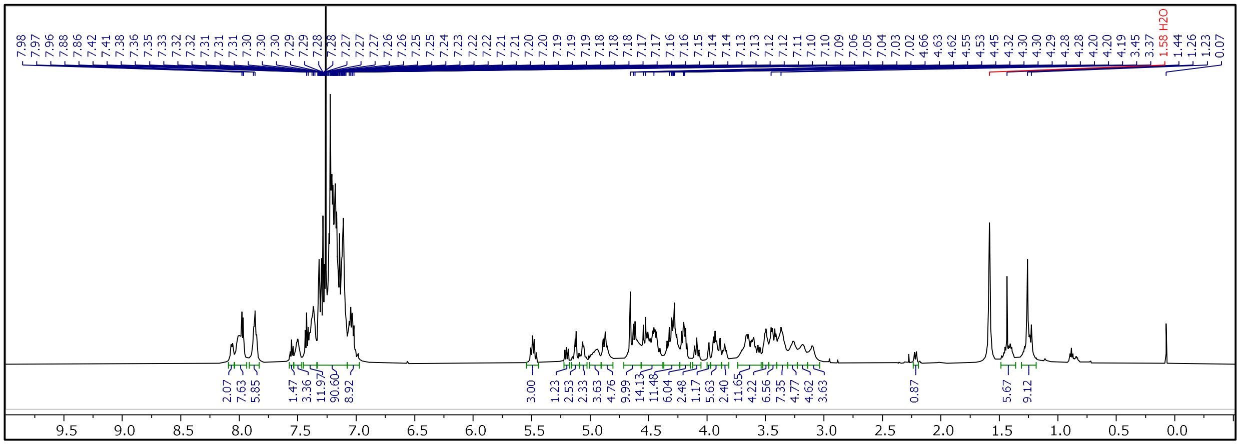

^{13}C NMR (151 MHz, CDCl$_3$) of **19**:

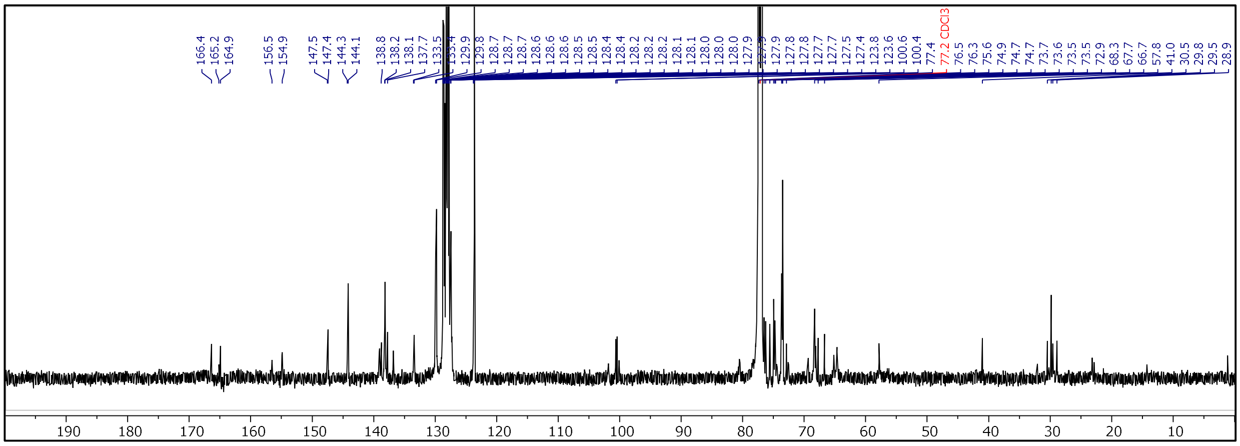

5.4 Protected Lacto-*N*-neotetraose (**20**)

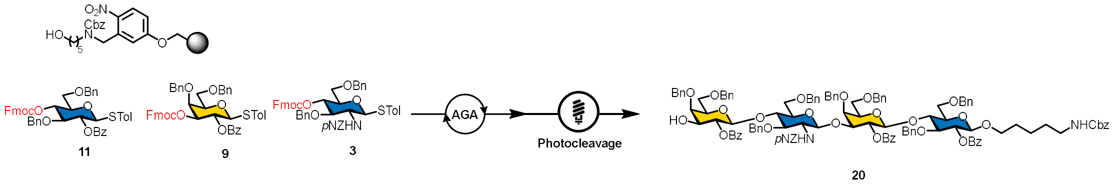

	Building blocks	Modules	Glycosylation condition
AGA		**Initiation** (40 mg resin)	
	**11**	**Acidic wash**<br>**Thioglycoside glycosylation**<br>**Capping**<br>**Fmoc deprotection**	**6.5 eq.** / **t (min)**<br>**T_1 = -20 °C** / **5**<br>**T_2 = 0 °C** / **20**
	**9**	**Acidic wash**<br>**Thioglycoside glycosylation**<br>**Capping**<br>**Fmoc deprotection**	**6.5 eq.** / **t (min)**<br>**T_1 = -40 °C** / **5**<br>**T_2 = -20 °C** / **20**
	**3**	**Acidic wash**<br>**Thioglycoside glycosylation**<br>**Pyridine wash** (**x2** for the three modules above)<br>**Capping**<br>**Fmoc deprotection**	**6.5 eq.** / **t (min)**<br>**T_1 = -10 °C** / **30**
	**9**	**Acidic wash**<br>**Thioglycoside glycosylation**<br>**Capping**<br>**Fmoc deprotection**	**6.5 eq.** / **t (min)**<br>**T_1 = -40 °C** / **5**<br>**T_2 = -20 °C** / **20**
**Post AGA**		**Photocleavage**<br>**NP-HPLC Purification**	

Protected **20** (9.96 mg, 4.8 µmol, 36%) was obtained as a colorless syrup by purification using preparative NP-HPLC (**Method C**).

Analytical NP-HPLC of the crude **20** (**Method A**, ELSD trace, t_R = 27.3 min)

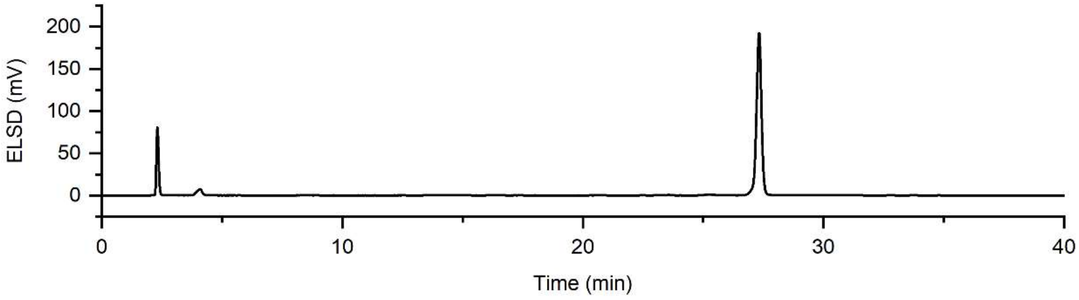

^{1}H NMR (600 MHz, CDCl$_3$) δ 8.04 – 7.99 (m, 2H), 7.96 (ddd, *J* = 13.4, 8.2, 1.4 Hz, 4H), 7.86 (d, *J* = 7.7 Hz, 2H), 7.56 (td, *J* = 7.4, 1.4 Hz, 1H), 7.53 – 7.48 (m, 2H), 7.43 (t, *J* = 7.8 Hz, 3H), 7.39 – 7.35 (m, 5H), 7.34 (d, *J* = 5.9 Hz, 5H), 7.32 – 7.18 (m, 25H), 7.18 – 7.13 (m, 5H), 7.12 – 7.06 (m, 7H), 7.06 – 7.01 (m, 1H), 6.96 (t, *J* = 7.6 Hz, 2H), 5.53 (dd, *J* = 10.1, 7.9 Hz, 1H), 5.20 (dd, *J* = 10.0, 7.9 Hz, 1H), 5.15 (dd, *J* = 9.4, 8.0 Hz, 1H), 5.04 (s, 2H), 5.00 (d, *J* = 11.6 Hz, 1H), 4.91 (d, *J* = 11.1 Hz, 1H), 4.88 (d, *J* = 11.1 Hz, 1H), 4.70 – 4.61 (m, 4H), 4.60 – 4.53 (m, 5H), 4.52 – 4.46 (m, 2H), 4.37 – 4.23 (m, 8H), 4.15 (d, *J* = 11.8 Hz, 1H), 4.02 – 3.96 (m, 2H), 3.92 (t, *J* = 8.9 Hz, 1H), 3.89 (d, *J* = 3.7 Hz, 1H), 3.77 – 3.59 (m, 6H), 3.56 (d, *J* = 10.6 Hz, 1H), 3.50 (td, *J* = 10.2, 5.7 Hz, 4H), 3.46 – 3.38 (m, 3H), 3.38 – 3.32 (m, 2H), 3.31 – 3.22 (m, 3H), 3.22 – 3.16 (m, 1H), 2.91 – 2.82 (m, 2H), 2.22 (d, *J* = 10.4 Hz, 1H), 1.48 – 1.31 (m, 4H), 1.17 – 1.03 (m, 2H).

^{13}C NMR (151 MHz, CDCl$_3$) δ 166.4, 165.2, 164.9, 147.4, 144.2, 139.3, 138.8, 138.4, 138.3, 138.2, 138.0, 137.8, 136.9, 133.5, 133.3, 133.1, 130.2, 129.9, 129.8, 128.7, 128.7, 128.6, 128.6, 128.5, 128.5, 128.2, 128.2, 128.1, 128.1, 128.0, 127.9, 127.8, 127.7, 127.4, 127.1, 123.6, 101.3, 100.7, 100.4, 80.7, 77.2, 76.5, 76.4, 75.6, 75.0, 75.0, 74.8, 74.7, 74.6, 73.7, 73.6, 73.5, 73.5, 72.9, 72.7, 69.5, 68.4, 68.3, 67.9, 67.7, 66.6, 57.8, 40.9, 29.9, 29.5, 29.0, 23.2.

HRMS (QToF): Calcd for $C_{122}H_{125}N_3O_{29}Na$ [M + Na]$^+$ 2118.8291; found 2118.8315.

^{1}H NMR (600 MHz, CDCl$_3$) of **20**:

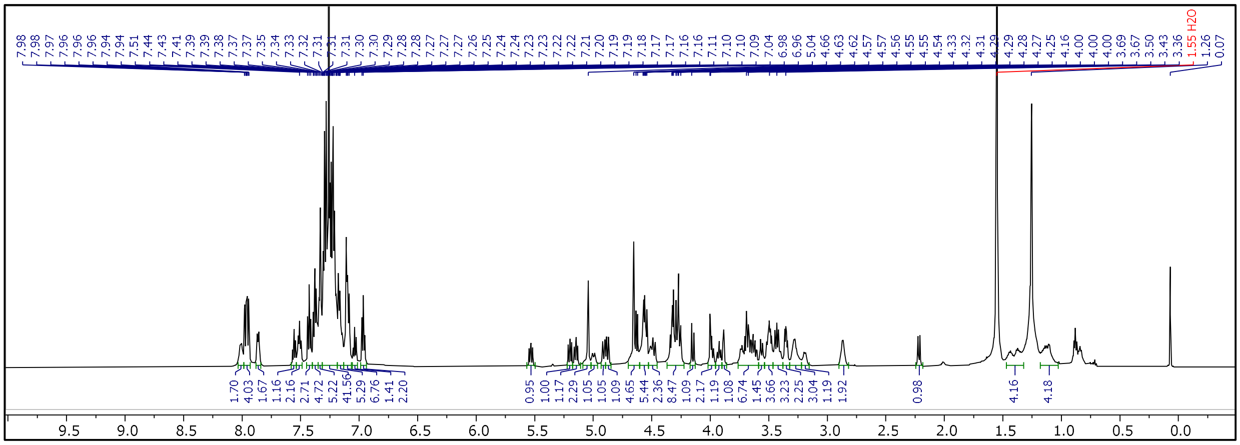

^{13}C NMR (151 MHz, $CDCl_3$) of **20**:

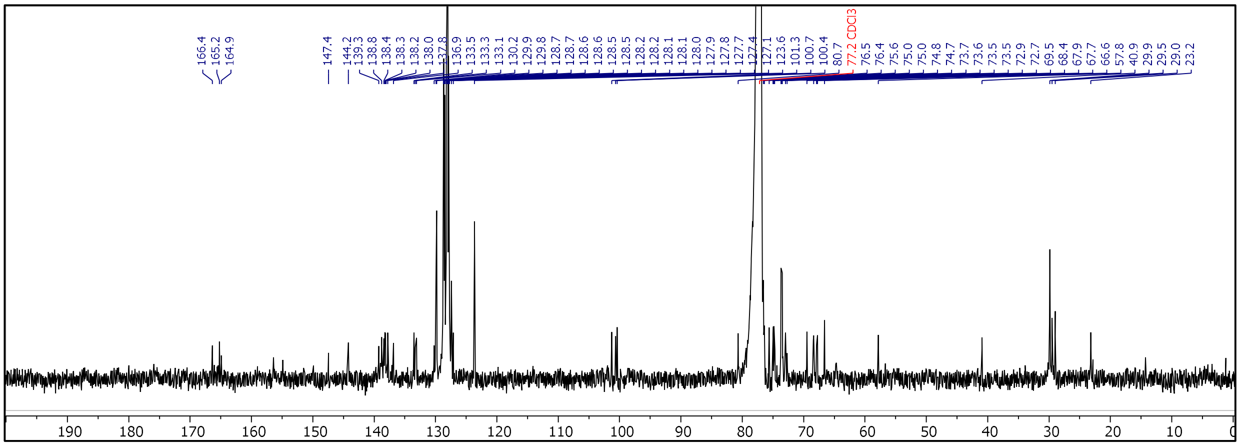

5.5 Protected *para*-Lacto-*N*-neohexaose (**21**)

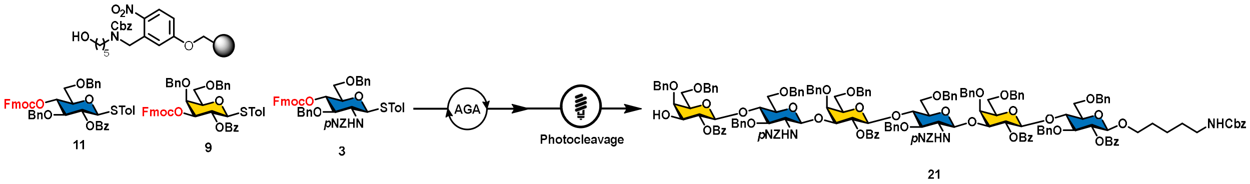

	Building blocks	Modules	Glycosylation condition
AGA		**Initiation** (40 mg resin)	
	11	Acidic wash<br>Thioglycoside glycosylation<br>Capping<br>Fmoc deprotection	6.5 eq. / t (min)<br>T_1 = -20 °C / 5<br>T_2 = 0 °C / 20
	9	Acidic wash<br>Thioglycoside glycosylation<br>Capping<br>Fmoc deprotection	6.5 eq. / t (min)<br>T_1 = -40 °C / 5<br>T_2 = -20 °C / 20
	3	Acidic wash<br>Thioglycoside glycosylation<br>Pyridine wash (x2)<br>Capping<br>Fmoc deprotection	6.5 eq. / t (min)<br>T_1 = -10 °C / 30
	9	Acidic wash<br>Thioglycoside glycosylation<br>Capping<br>Fmoc deprotection	6.5 eq. / t (min)<br>T_1 = -40 °C / 5<br>T_2 = -20 °C / 20
Post AGA		Photocleavage<br>NP-HPLC Purification	

Protected **21** (11.3 mg, 3.7 μmol, 28%) was obtained as a colorless syrup by purification using preparative NP-HPLC (**Method D**).

Analytical NP-HPLC of the crude **21** (**Method B**, ELSD trace, t_R = 26.1 min)

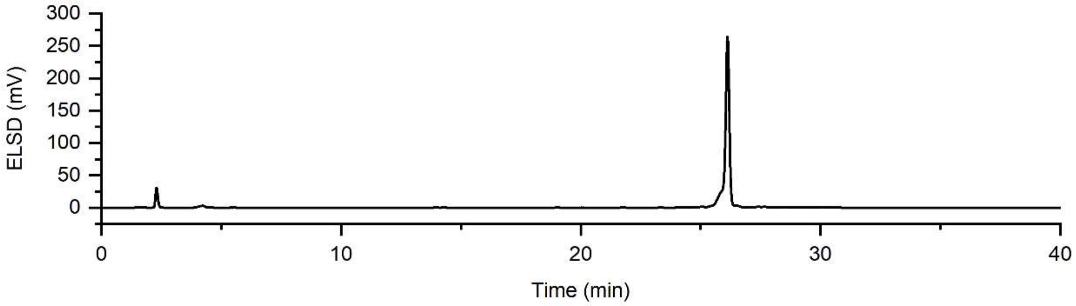

^{1}H NMR (600 MHz, $CDCl_3$) δ 8.05 – 8.00 (m, 3H), 7.96 (dd, *J* = 15.0, 7.5 Hz, 4H), 7.87 (dd, *J* = 15.0, 7.4 Hz, 3H), 7.64 – 7.48 (m, 5H), 7.43 (d, *J* = 6.9 Hz, 3H), 7.40 – 7.14 (m, 60H), 7.14 – 7.01 (m, 13H), 6.96 (t, *J* = 7.5 Hz, 2H), 5.51 (q, *J* = 9.0 Hz, 2H), 5.20 (dd, *J* = 9.5, 8.2 Hz, 1H), 5.14 (t, *J* = 8.6 Hz, 1H), 5.04 (s, 2H), 5.01 – 4.94 (m, 2H), 4.93 – 4.86 (m, 3H), 4.66 (s, 2H), 4.63 (d, *J* = 7.9 Hz, 2H, **H_1**), 4.59 – 4.49 (m, 6H, **H_1**), 4.49 – 4.42 (m, 3H), 4.38 – 4.18 (m, 11H, **H_1**), 4.16 – 4.08 (m, 2H), 4.05 – 3.82 (m, 7H), 3.79 – 3.54 (m, 11H), 3.54 – 3.32 (m, 13H), 3.32 – 3.13 (m, 8H), 2.94 – 2.81 (m, 2H), 2.23 (d, *J* = 10.3 Hz, 1H, O*H*), 1.49 – 1.31 (m, 4H), 1.30 – 1.04 (m, 2H).

^{13}C NMR (151 MHz, $CDCl_3$) δ 166.4, 165.2, 164.9, 156.4, 154.90, 154.86, 147.44, 147.40, 144.2, 144.1, 138.7, 138.4, 138.3, 138.2, 138.1, 138.02, 138.00, 137.7, 136.9, 133.5, 133.4, 133.3, 133.1, 130.2, 129.9, 129.8, 128.7, 128.6, 128.6, 128.5, 128.4, 128.2, 128.2, 128.1, 128.1, 128.0, 128.0, 128.0, 127.9, 127.9, 127.8, 127.7, 127.5, 127.3, 127.1, 123.6, 101.3 (**C_1**), 100.64 (**C_1**), 100.61 (**C_1**), 100.4 (**C_1**), 80.7, 77.2, 76.5, 76.4, 76.3, 75.6, 75.0, 74.9, 74.8, 74.7, 74.7, 73.7, 73.6, 73.50, 73.49, 73.47, 73.4, 72.9, 69.5, 68.4, 68.3, 67.7, 66.6, 57.8, 57.7, 40.9, 29.8, 29.5, 29.0, 23.2.

HRMS (QToF): Calcd for $C_{177}H_{179}N_5O_{43}Na$ [M + Na]$^+$ 3085.1866; found 3085.1982.

^{1}H NMR (600 MHz, $CDCl_3$) of **21**:

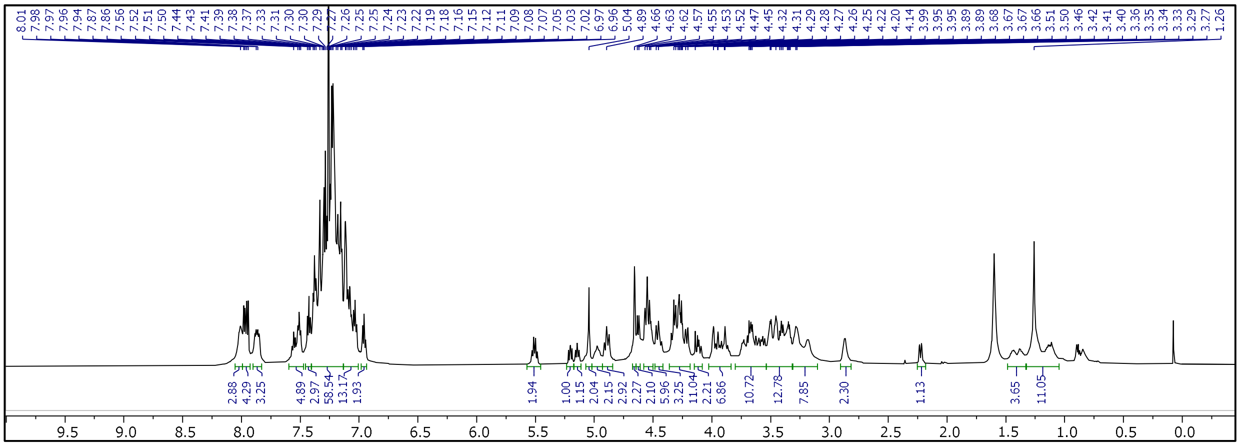

^{13}C NMR (151 MHz, $CDCl_3$) of **21**:

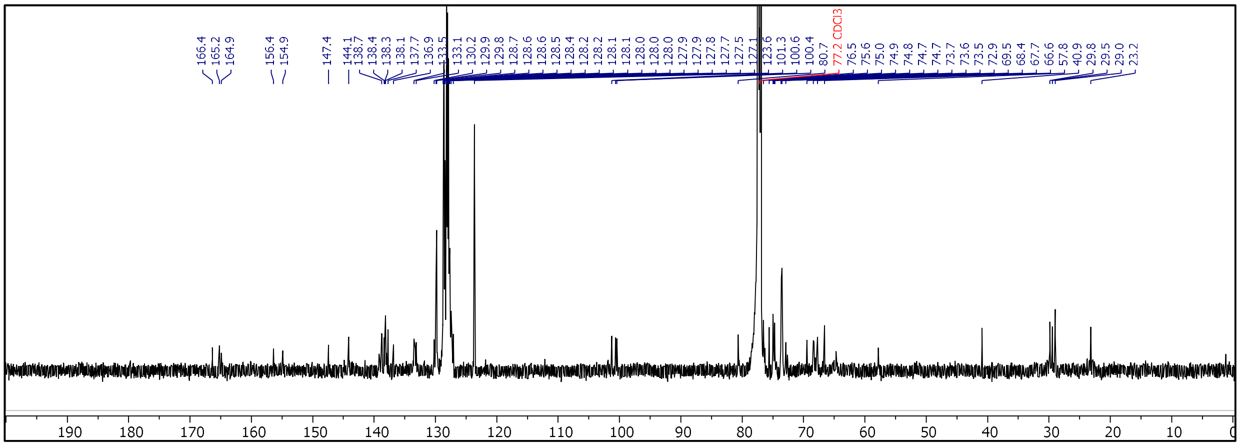

5.6 Protected Lacto-*N*-tetraose (**22**)

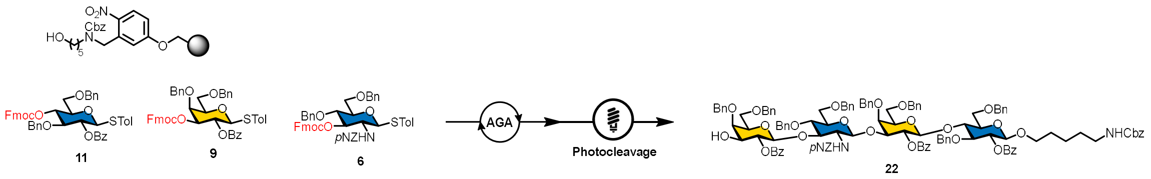

	Building blocks	Modules	Glycosylation condition
AGA		**Initiation** (40 mg resin)	
	11	Acidic wash<br>Thioglycoside glycosylation<br>Capping<br>Fmoc deprotection	6.5 eq. / t (min)<br>T_1 = -20 °C / 5<br>T_2 = 0 °C / 20
	9	Acidic wash<br>Thioglycoside glycosylation<br>Capping<br>Fmoc deprotection	6.5 eq. / t (min)<br>T_1 = -40 °C / 5<br>T_2 = -20 °C / 20
	6	Acidic wash<br>Thioglycoside glycosylation<br>Pyridine wash (x2)<br>Capping<br>Fmoc deprotection	6.5 eq. / t (min)<br>T_1 = 0 °C / 40
	9	Acidic wash<br>Thioglycoside glycosylation<br>Capping<br>Fmoc deprotection	6.5 eq. / t (min)<br>T_1 = -40 °C / 5<br>T_2 = -20 °C / 20
Post AGA		Photocleavage<br>NP-HPLC Purification	

Protected **22** (8.85 mg, 4.2 μmol, 32%) was obtained as a colorless syrup by purification using preparative NP-HPLC (**Method D**).

Analytical NP-HPLC of the crude **22** (**Method B**, ELSD trace, t_R = 23.3 min)

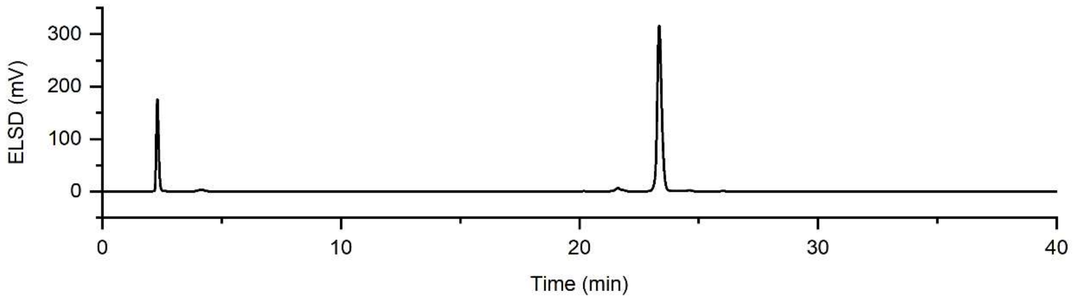

^{1}H NMR (600 MHz, CDCl$_3$) δ 8.15 (d, *J* = 8.2 Hz, 1H), 7.99 – 7.92 (m, 3H), 7.85 (d, *J* = 7.8 Hz, 1H), 7.57 – 7.49 (m, 3H), 7.38 (t, *J* = 7.6 Hz, 4H), 7.36 – 7.31 (m, 10H), 7.31 – 7.13 (m, 34H), 7.12 – 7.08 (m, 4H), 7.04 (td, *J* = 7.2, 1.4 Hz, 2H), 6.97 (t, *J* = 7.5 Hz, 2H), 5.46 (dd, *J* = 10.1, 7.9 Hz, 1H), 5.14 (t, *J* = 8.8 Hz, 2H), 5.05 (s, 2H), 4.96 (d, *J* = 10.5 Hz, 1H), 4.91 (d, *J* = 11.2 Hz, 1H), 4.82 (d, *J* = 12.0 Hz, 1H), 4.74 (d, *J* = 8.2 Hz, 1H), 4.67 – 4.54 (m, 6H), 4.54 – 4.45 (m, 5H), 4.40 (d, *J* = 10.4 Hz, 1H), 4.37 – 4.28 (m, 4H), 4.28 – 4.21 (m, 3H), 4.18 (d, *J* = 11.8 Hz, 1H), 4.02 (d, *J* = 3.1 Hz, 1H), 3.99 (t, *J* = 9.1 Hz, 1H), 3.80 – 3.71 (m, 4H), 3.71 – 3.64 (m, 3H), 3.55 – 3.49 (m, 2H), 3.47 – 3.31 (m, 7H), 3.30 – 3.25 (m, 2H), 3.18 – 3.10 (m, 2H), 2.87 (q, *J* = 7.0 Hz, 2H), 2.82 – 2.74 (m, 1H), 2.29 (d, *J* = 10.0 Hz, 1H), 1.48 – 1.33 (m, 4H), 1.18 – 1.02 (m, 2H).

^{13}C NMR (151 MHz, CDCl$_3$) δ 166.7, 165.2, 165.1, 156.4, 154.7, 147.8, 144.1, 139.3, 138.8, 138.5, 138.3, 138.2, 138.1, 137.8, 136.9, 133.6, 133.3, 133.1, 130.3, 130.1, 129.8, 128.7, 128.6, 128.6, 128.5, 128.4, 128.3, 128.2, 128.2, 128.1, 128.0, 127.9, 127.9, 127.7, 127.6, 127.3, 127.1, 123.7, 101.3, 100.8, 100.6, 100.5, 80.6, 79.2, 77.2, 76.5, 76.2, 75.7, 75.03, 74.97, 74.9, 74.8, 74.6, 73.8, 73.7, 73.6, 73.4, 73.3, 73.0, 69.4, 68.4, 67.7, 66.6, 64.8, 58.9, 40.9, 29.9, 29.5, 29.0, 23.2.
HRMS (QToF): Calcd for $C_{122}H_{125}N_3O_{29}Na$ $[M + Na]^+$ 2118.8291; found 2118.8306.

^{1}H NMR (600 MHz, CDCl$_3$) of **22**:

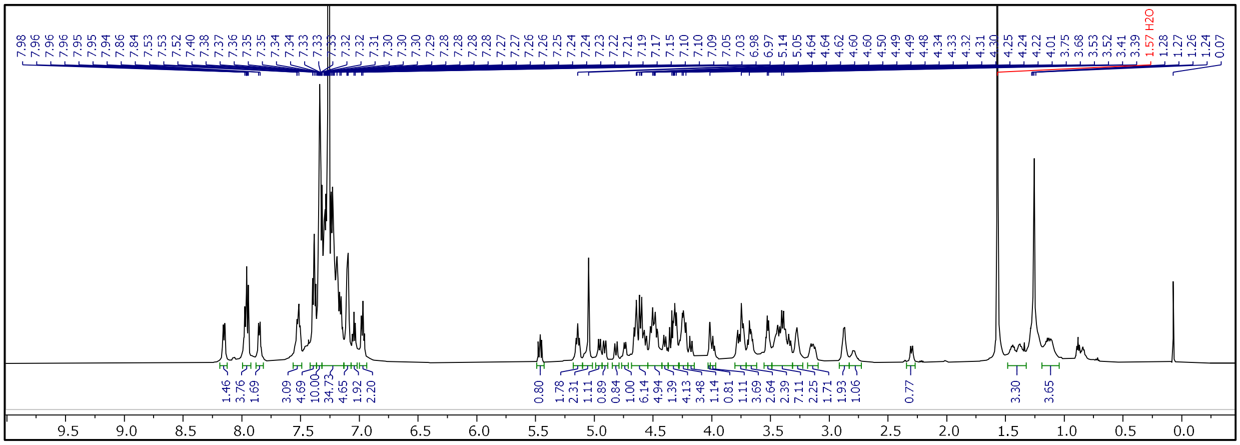

^{13}C NMR (151 MHz, $CDCl_3$) of **22**:

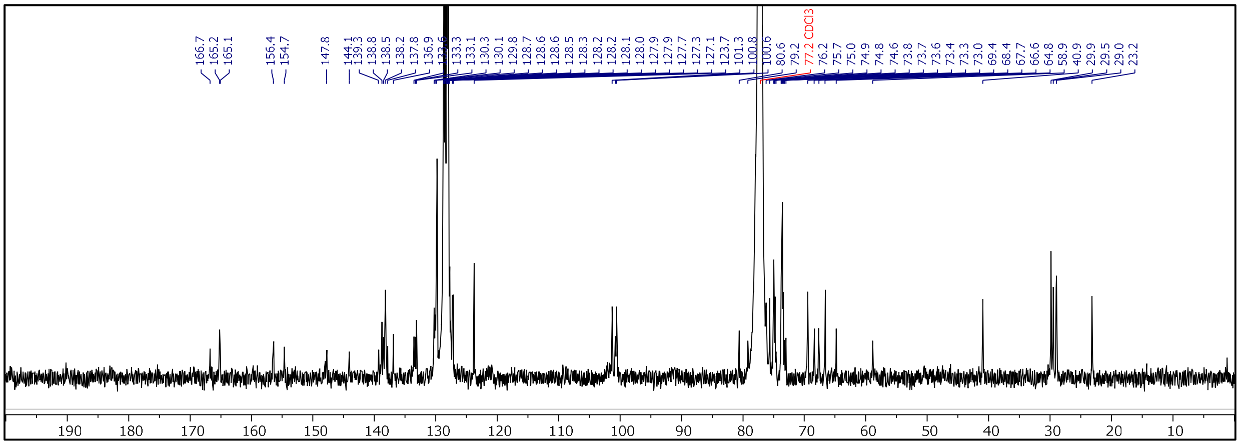

5.7 Protected Lacto-*N*-fucopentaose III (**23**)

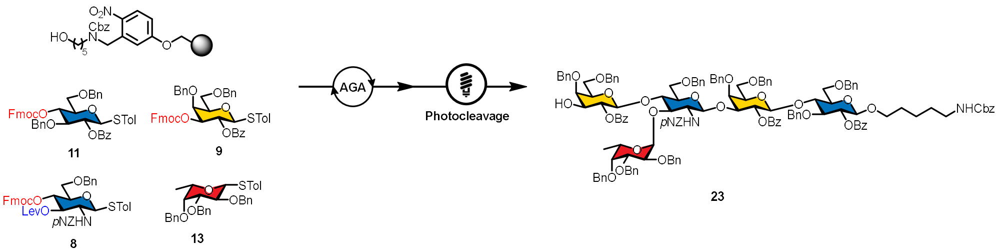

	Building blocks	Modules	Glycosylation condition
AGA		**Initiation** (40 mg resin)	
	11	Acidic wash<br>Thioglycoside glycosylation<br>Capping<br>Fmoc deprotection	6.5 eq. t (min)<br>T_1 = -20 °C 5<br>T_2 = 0 °C 20
	9	Acidic wash<br>Thioglycoside glycosylation<br>Capping<br>Fmoc deprotection	6.5 eq. t (min)<br>T_1 = -40 °C 5<br>T_2 = -20 °C 20
	8	Acidic wash<br>Thioglycoside glycosylation<br>Pyridine wash (x2)<br>Capping<br>Lev deprotection	6.5 eq. t (min)<br>T_1 = 0 °C 30
	13	Acidic wash<br>Thioglycoside glycosylation<br>Capping<br>Fmoc deprotection	8.0 eq. t (min)<br>T_1 = -40 °C 5<br>T_2 = -20 °C 20
	9	Acidic wash<br>Thioglycoside glycosylation<br>Capping<br>Fmoc deprotection	6.5 eq. t (min)<br>T_1 = -40 °C 5<br>T_2 = -20 °C 20
Post AGA		Photocleavage<br>NP-HPLC Purification	

Protected **23** (9.29 mg, 3.8 µmol, 29%) was obtained as a colorless syrup by purification using preparative NP-HPLC (**Method D**).

Analytical NP-HPLC of the crude **23** (**Method B**, ELSD trace, t_R = 28.9 min)

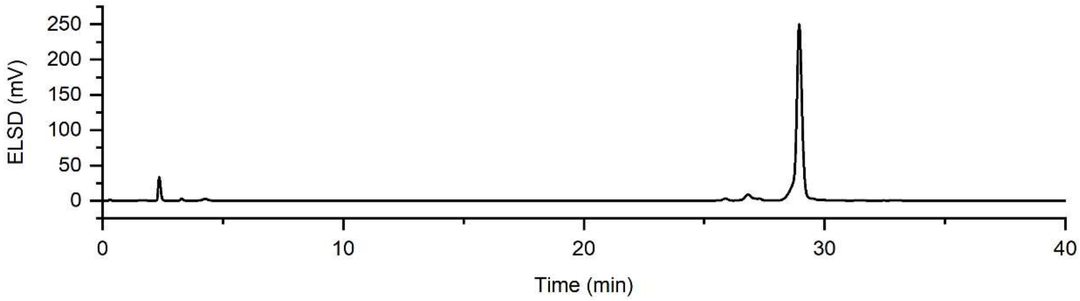

^{1}H NMR (700 MHz, $CDCl_3$) δ 8.05 (d, *J* = 8.3 Hz, 1H), 7.97 – 7.93 (m, 3H), 7.77 (d, *J* = 7.7 Hz, 1H), 7.57 (t, *J* = 7.4 Hz, 1H), 7.51 (t, *J* = 7.5 Hz, 1H), 7.44 (t, *J* = 7.7 Hz, 3H), 7.38 (t, *J* = 7.6 Hz, 3H), 7.33 (dq, *J* = 11.2, 6.4 Hz, 10H), 7.31 – 7.27 (m, 11H), 7.27 – 7.18 (m, 27H), 7.18 – 7.12 (m, 5H), 7.11 – 7.02 (m, 7H), 6.96 (t, *J* = 7.5 Hz, 1H), 5.52 (dd, *J* = 10.1, 8.0 Hz, 1H), 5.16 – 5.10 (m, 2H), 5.03 (s, 2H), 4.92 (d, *J* = 11.0 Hz, 1H), 4.87 (s, 1H), 4.76 – 4.66 (m, 5H), 4.63 – 4.42 (m, 11H), 4.39 (d, *J* = 11.9 Hz, 1H), 4.37 – 4.29 (m, 3H), 4.25 (dd, *J* = 17.6, 11.9 Hz, 2H), 4.15 (d, *J* = 11.3 Hz, 1H), 4.11 (d, *J* = 11.7 Hz, 1H), 4.06 (t, *J* = 9.2 Hz, 1H), 4.01 (d, *J* = 3.0 Hz, 1H), 3.99 (t, *J* = 9.2 Hz, 2H), 3.94 (d, *J* = 3.6 Hz, 1H), 3.87 (dd, *J* = 11.3, 3.1 Hz, 1H), 3.84 – 3.78 (m, 2H), 3.75 – 3.59 (m, 7H), 3.49 (d, *J* = 6.8 Hz, 1H), 3.47 – 3.43 (m, 2H), 3.40 (t, *J* = 8.3 Hz, 1H), 3.37 – 3.32 (m, 3H), 3.30 – 3.23 (m, 2H), 3.15 – 3.11 (m, 1H), 2.89 – 2.83 (m, 2H), 2.31 (d, *J* = 9.4 Hz, 1H), 1.34 – 1.17 (m, 4H), 1.17 – 1.04 (m, 6H).

^{13}C NMR (176 MHz, $CDCl_3$) δ 166.4, 165.2, 165.1, 156.4, 154.5, 147.5, 143.7, 139.3, 139.2, 139.1, 138.7, 138.4, 138.3, 138.22, 138.15, 138.0, 137.7, 136.8, 133.6, 133.42, 133.36, 133.3, 133.1, 130.2, 129.9, 129.8, 129.7, 129.6. 128.9, 128.7, 128.7, 128.7, 128.64, 128.56, 128.5, 128.5, 128.4, 128.3, 128.2, 128.1, 128.0, 127.9, 127.7, 127.7, 127.6, 127.5, 127.4, 127.1, 123.6, 101.3, 100.8, 99.4, 97.3, 80.7, 80.1, 79.8, 78.5, 77.2, 76.5, 76.4, 76.3, 75.8, 75.1, 75.0, 74.9, 74.9, 74.8, 74.3, 73.8, 73.7, 73.5, 73.2, 72.8, 72.7, 72.6, 69.5, 68.4, 68.2, 67.7, 67.5, 66.6, 65.0, 40.9, 29.9, 29.5, 29.0, 23.2, 22.8, 16.6, 14.3.

HRMS (QToF): Calcd for $C_{142}H_{147}N_3O_{37}Na$ [M + Na]$^+$ 2444.9809; found 2444.9832.

^{1}H NMR (700 MHz, $CDCl_3$) of **23**:

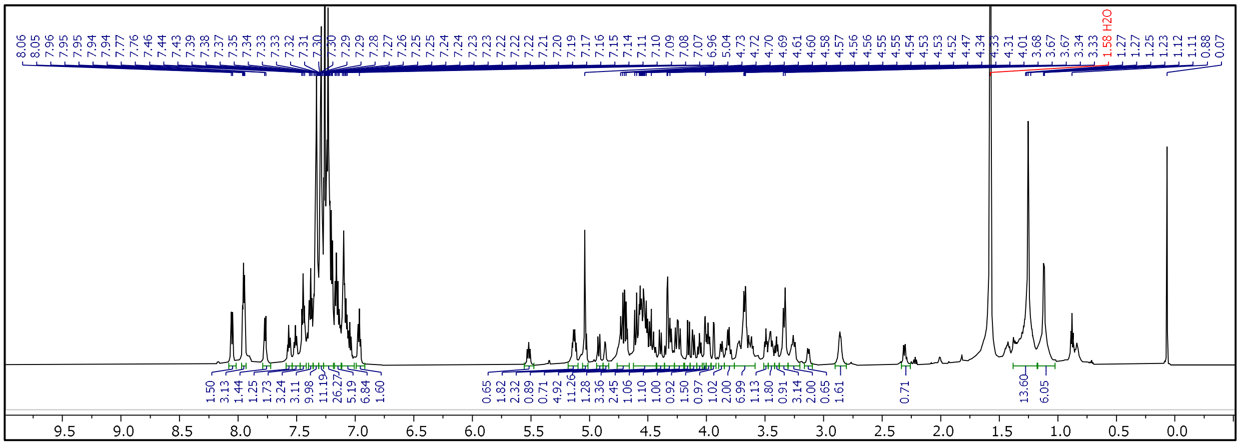

^{13}C NMR (176 MHz, $CDCl_3$) of **23**:

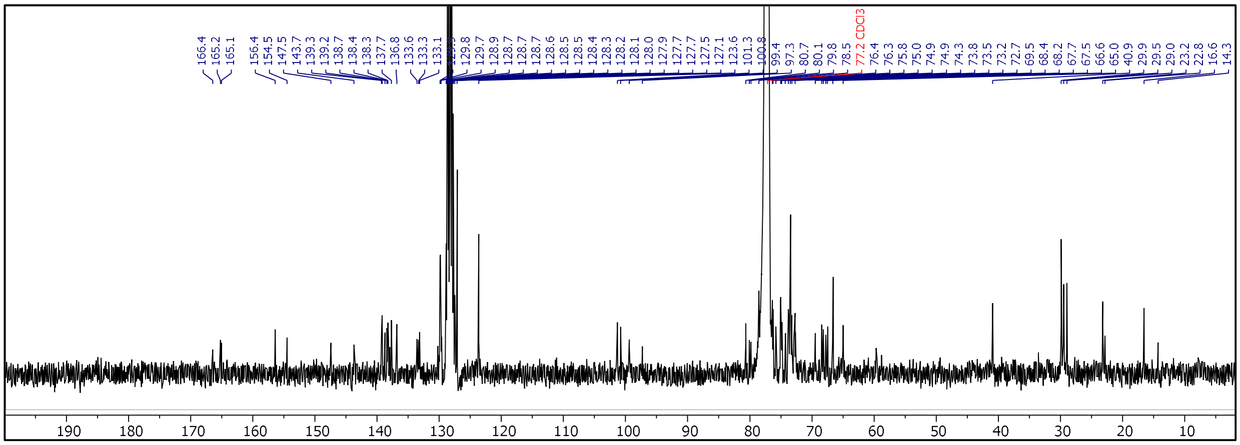

5.8 Protected Lacto-*N*-fucopentaose II (**24**)

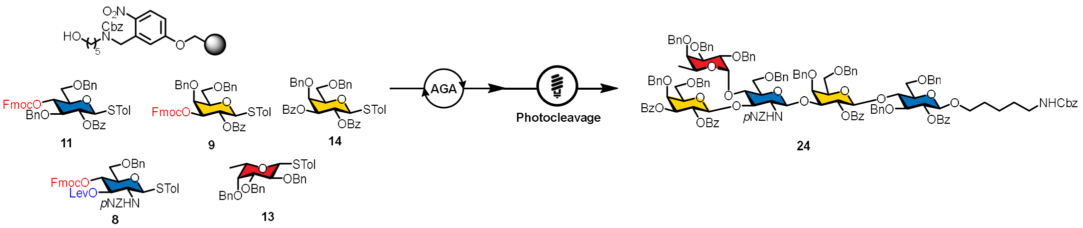

	Building blocks	Modules	Glycosylation condition
AGA		**Initiation** (40 mg resin)	
	**11**	**Acidic wash**<br>**Thioglycoside glycosylation**<br>**Capping**<br>**Fmoc deprotection**	**6.5 eq.** — **t (min)**<br>**T_1 = -20 °C** — **5**<br>**T_2 = 0 °C** — **20**
	**9**	**Acidic wash**<br>**Thioglycoside glycosylation**<br>**Capping**<br>**Fmoc deprotection**	**6.5 eq.** — **t (min)**<br>**T_1 = -40 °C** — **5**<br>**T_2 = -20 °C** — **20**
	**8**	**Acidic wash** (x2)<br>**Thioglycoside glycosylation** (x2)<br>**Pyridine wash** (x2)<br>**Capping**<br>**Lev deprotection**	**6.5 eq.** — **t (min)**<br>**T_1 = 0 °C** — **30**
	**14**	**Acidic wash**<br>**Thioglycoside glycosylation**<br>**Capping**<br>**Fmoc deprotection**	**6.5 eq.** — **t (min)**<br>**T_1 = -20 °C** — **10**<br>**T_2 = 0 °C** — **20**
	**13**	**Acidic wash**<br>**Thioglycoside glycosylation**<br>**Capping**	**8.0 eq.** — **t (min)**<br>**T_1 = -40 °C** — **5**<br>**T_2 = -20 °C** — **20**
Post AGA		**Photocleavage**<br>**NP-HPLC Purification**	

Protected **24** (7.0 mg, 2.7 μmol, 21%) was obtained as a colorless syrup by purification using preparative NP-HPLC (**Method C**).

Analytical NP-HPLC of the crude **24** (**Method A**, ELSD trace, t_R = 29.1 min)

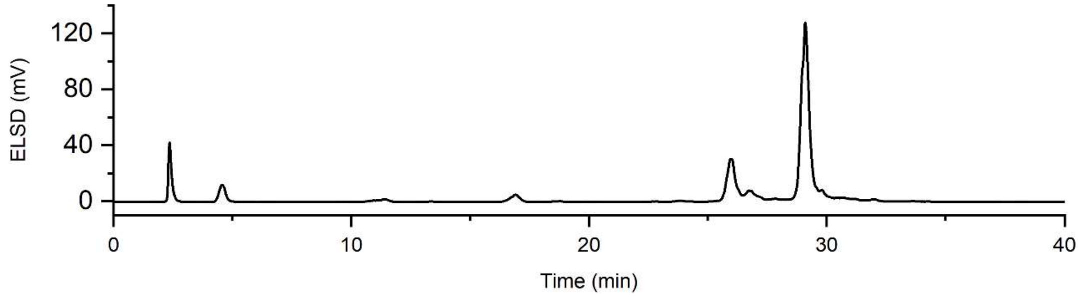

^{1}H NMR (700 MHz, $CDCl_3$) δ 8.29 (d, *J* = 8.4 Hz, 2H), 7.99 (d, *J* = 7.6 Hz, 2H), 7.95 (d, *J* = 7.4 Hz, 2H), 7.92 (d, *J* = 7.6 Hz, 2H), 7.88 (d, *J* = 7.4 Hz, 2H), 7.61 (t, *J* = 7.4 Hz, 1H), 7.55 – 7.44 (m, 5H), 7.43 – 7.01 (m, 63H), 6.95 (t, *J* = 7.5 Hz, 2H), 5.61 (dd, *J* = 9.8, 8.6 Hz, 1H), 5.43 (dd, *J* = 10.0, 8.1 Hz, 1H), 5.14 (t, *J* = 8.7 Hz, 1H), 5.05 (s, 2H), 4.95 (dd, *J* = 10.4, 3.3 Hz, 1H), 4.93 – 4.88 (m, 2H), 4.85 (d, *J* = 5.7 Hz, 1H), 4.78 (d, *J* = 11.6 Hz, 1H), 4.73 (d, *J* = 12.0 Hz, 1H), 4.69 – 4.39 (m, 15H), 4.39 – 4.14 (m, 10H), 4.10 – 3.94 (m, 6H), 3.82 – 3.72 (m, 3H), 3.71 – 3.65 (m, 3H), 3.65 – 3.59 (m, 2H), 3.57 – 3.53 (m, 1H), 3.52 – 3.48 (m, 1H), 3.48 – 3.41 (m, 3H), 3.40 – 3.36 (m, 1H), 3.33 (d, *J* = 10.4 Hz, 1H), 3.31 – 3.26 (m, 1H), 3.22 (s, 1H), 3.11 (d, *J* = 9.3 Hz, 1H), 2.88 (d, *J* = 6.0 Hz, 2H), 1.49 – 1.42 (m, 2H), 1.41 – 1.36 (m, 2H), 1.31 – 1.24 (m, 3H), 1.20 – 1.08 (m, 2H).

^{13}C NMR (151 MHz, $CDCl_3$) δ 166.0, 165.2, 156.4, 154.5, 148.1, 143.9, 139.4, 139.2, 138.8, 138.6, 138.3, 138.1, 137.8, 136.9, 133.6, 133.3, 133.1, 130.3, 130.0, 129.8, 129.5, 129.1, 128.9, 128.8, 128.7, 128.7, 128.6, 128.6, 128.5, 128.5, 128.4, 128.2, 128.2, 128.2, 128.1, 128.0, 128.0, 128.0, 127.9, 127.8, 127.7, 127.6, 127.6, 127.4, 127.3, 127.1, 124.1, 101.3, 101.1, 100.6, 100.0, 97.7, 80.6, 80.5, 79.0, 78.4, 77.4, 77.2, 76.9, 76.1, 76.0, 75.4, 75.2, 75.0, 74.9, 74.8, 74.3, 73.8, 73.6, 73.6, 73.4, 73.3, 72.9, 72.4, 72.0, 70.2, 69.4, 68.3, 67.7, 66.9, 66.6, 64.8, 59.7, 40.9, 33.9, 32.1, 30.5, 30.3, 29.9, 29.5, 29.0, 23.2, 22.8, 16.7, 14.3.

HRMS (QToF): Calcd for $C_{149}H_{151}N_3O_{34}Na$ $[M + Na]^+$ 2549.0077; found 2549.0146.

^{1}H NMR (700 MHz, $CDCl_3$) of **24**:

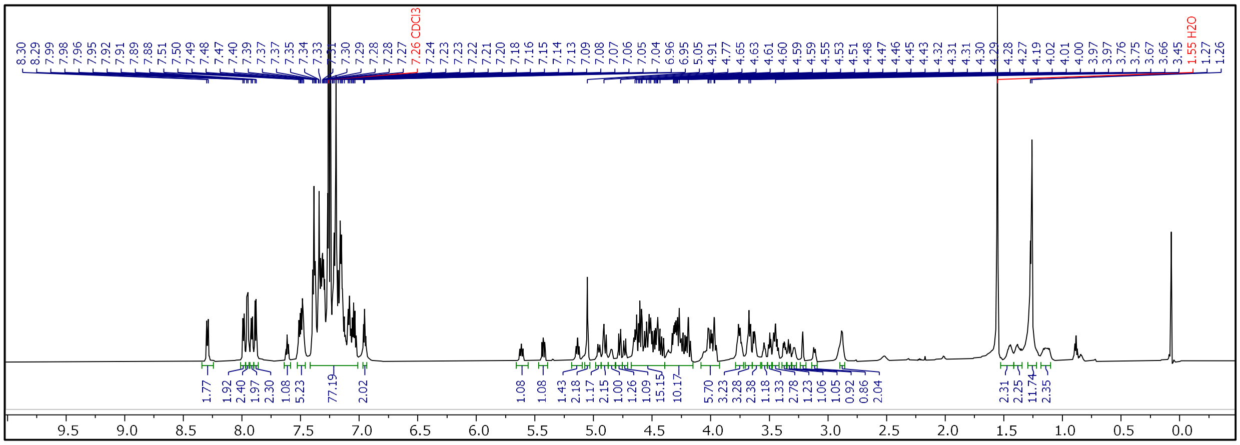

^{13}C NMR (151 MHz, $CDCl_3$) of **24**:

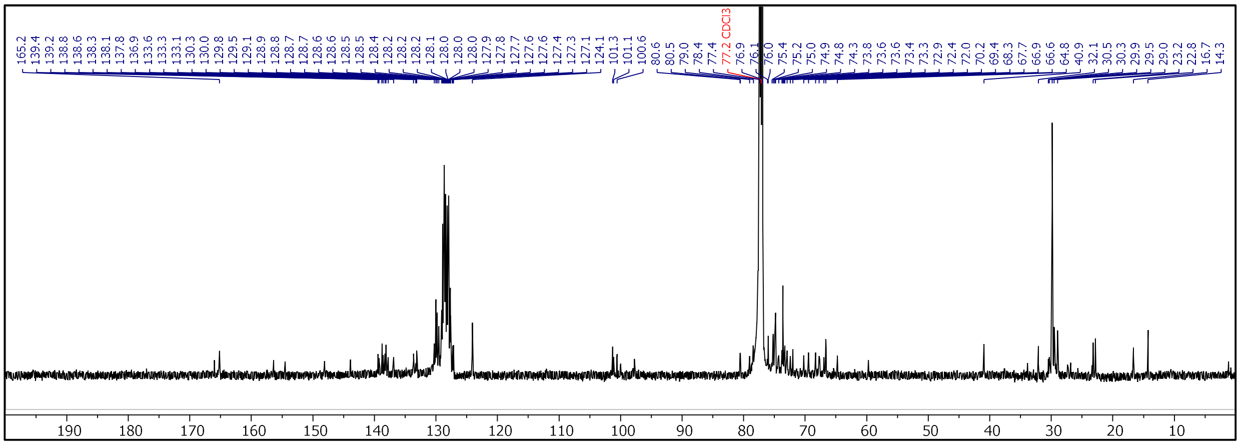

5.9.1 *p*NZ-protected Lacto-*N*-neohexaose (**25a**)

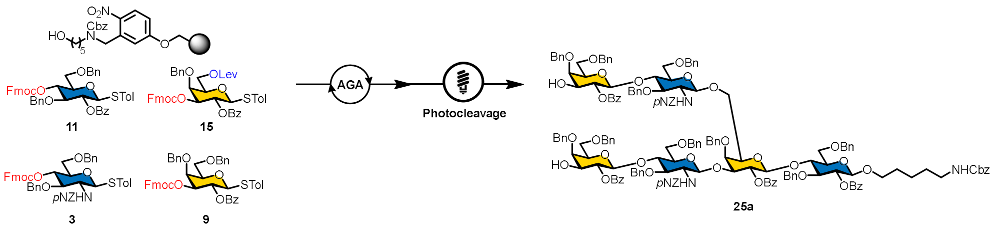

	Building blocks	Modules	Glycosylation condition
AGA		**Initiation** (40 mg resin)	
	**11**	**Acidic wash**<br>**Thioglycoside glycosylation**<br>**Capping**<br>**Fmoc deprotection**	**6.5 eq.** / **t (min)**<br>**T_1 = -20 °C** / **5**<br>**T_2 = 0 °C** / **20**
	**15**	**Acidic wash**<br>**Thioglycoside glycosylation**<br>**Capping**<br>**Lev deprotection**<br>**Fmoc deprotection**	**6.5 eq.** / **t (min)**<br>**T_1 = -20 °C** / **5**<br>**T_2 = 0 °C** / **20**
	**3**	**Acidic wash**<br>**Thioglycoside glycosylation**<br>**Pyridine wash** (**x4**: Acidic wash – Pyridine wash)<br>**Capping**<br>**Fmoc deprotection**	**6.5 eq.** / **t (min)**<br>**T_1 = -10 °C** / **30**
	**9**	**Acidic wash**<br>**Thioglycoside glycosylation**<br>**Pyridine wash** (**x2**: Acidic wash – Pyridine wash)<br>**Capping**<br>**Fmoc deprotection**	**6.5 eq.** / **t (min)**<br>**T_1 = -40 °C** / **5**<br>**T_2 = -20 °C** / **20**
**Post AGA**		**Photocleavage**<br>**NP-HPLC Purification**	

Protected **25a** (11.18 mg, 3.8 µmol, 29%) was obtained as a colorless syrup by purification using preparative NP-HPLC (**Method C**).

Analytical NP-HPLC of the crude **25a** (**Method A**, ELSD trace, t_R = 27.4 min)

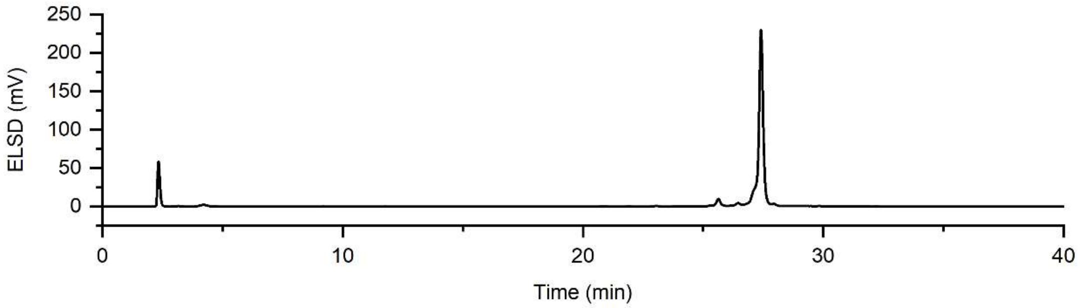

^{1}H NMR (600 MHz, $CDCl_3$) δ 8.07 – 7.95 (m, 7H), 7.86 (d, *J* = 7.9 Hz, 2H), 7.74 (d, *J* = 8.4 Hz, 2H), 7.64 – 7.52 (m, 4H), 7.51 – 7.38 (m, 10H), 7.37 – 7.19 (m, 46H), 7.19 – 7.07 (m, 10H), 7.08 – 6.93 (m, 5H), 6.73 (t, *J* = 7.6 Hz, 1H), 6.38 (t, *J* = 7.5 Hz, 1H), 5.81 – 5.67 (m, 2H), 5.53 (dd, *J* = 10.0, 8.0 Hz, 1H), 5.26 – 5.10 (m, 4H), 5.08 – 4.94 (m, 4H), 4.91 (d, *J* = 11.1 Hz, 1H), 4.86 (d, *J* = 10.8 Hz, 1H), 4.79 (d, *J* = 10.9 Hz, 1H), 4.72 – 4.54 (m, 10H), 4.52 – 4.39 (m, 6H), 4.38 – 4.24 (m, 9H), 4.20 (d, *J* = 11.7 Hz, 1H), 3.94 (t, *J* = 8.9 Hz, 1H), 3.90 (d, *J* = 3.7 Hz, 1H), 3.84 (d, *J* = 3.8 Hz, 1H), 3.78 – 3.43 (m, 17H), 3.42 – 3.33 (m, 4H), 3.33 – 3.23 (m, 4H), 3.22 – 3.10 (m, 3H), 2.91 (d, *J* = 8.7 Hz, 1H), 2.79 (q, *J* = 6.9 Hz, 2H), 2.67 (t, *J* = 9.6 Hz, 1H), 2.23 (d, *J* = 10.2 Hz, 1H), 2.17 (d, *J* = 10.7 Hz, 1H), 2.03 (d, *J* = 10.1 Hz, 1H), 1.35 – 1.18 (m, 4H), 1.11 (p, *J* = 8.2 Hz, 2H).

^{13}C NMR (151 MHz, $CDCl_3$) δ 166.3, 166.1, 165.5, 164.9, 156.4, 156.0, 147.5, 146.9, 145.7, 144.1, 139.7, 138.8, 138.6, 138.5, 138.3, 138.2, 137.9, 137.81, 137.76, 136.8, 133.6, 133.43, 133.39, 133.35, 130.2, 129.9, 129.9, 129.76, 129.73, 128.9, 128.8, 128.7, 128.7, 128.6, 128.6, 128.6, 128.4, 128.3, 128.3, 128.2, 128.2, 128.1, 128.1, 128.0, 128.0, 128.0, 127.9, 127.9, 127.9, 127.8, 127.7, 127.6, 127.4, 127.2, 126.5, 123.6, 123.2, 103.26, 103.25, 101.4, 100.8, 100.4, 100.2, 83.0, 81.5, 77.2, 76.6, 76.5, 76.0, 75.6, 75.5, 75.0, 74.8, 74.7, 74.6, 74.4, 74.2, 73.7, 73.6, 73.54, 73.50, 73.4, 73.0, 72.9, 72.8, 71.1, 69.5, 68.3, 67.9, 67.7, 67.52, 67.46, 66.6, 57.9, 56.5, 40.9, 29.9, 29.5, 29.0, 22.9.

HRMS (QToF): Calcd for $C_{170}H_{173}N_5O_{43}Na$ $[M + Na]^+$ 2995.1396; found 2995.1318.

^{1}H NMR (600 MHz, $CDCl_3$) of **25a**:

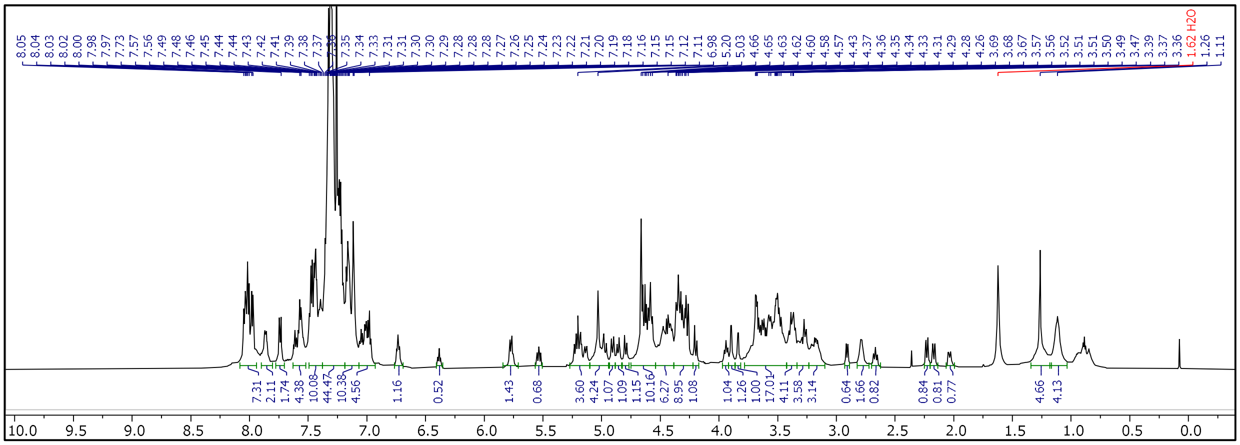

^{13}C NMR (151 MHz, $CDCl_3$) of **25a**:

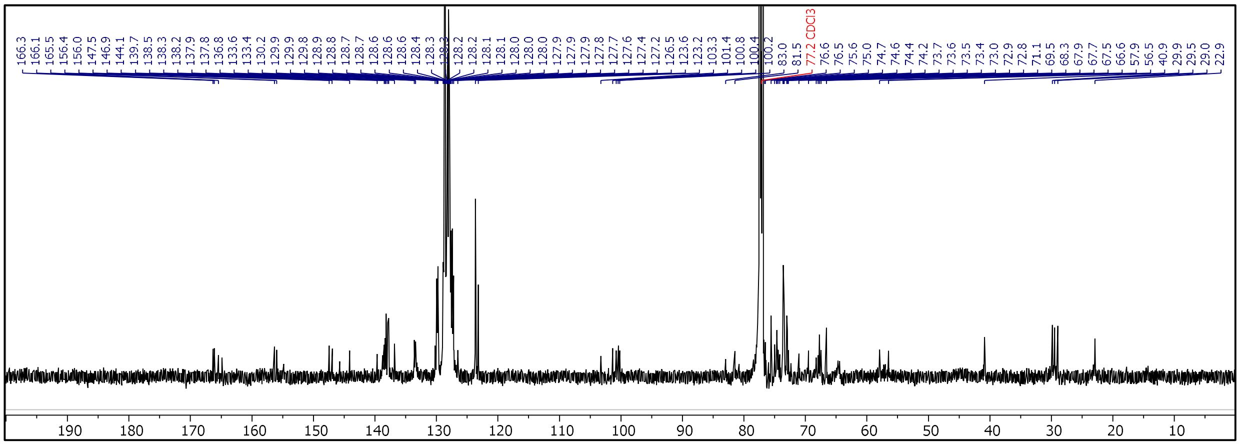

5.9.2 TCA-protected Lacto-*N*-neohexaose (**25b**)

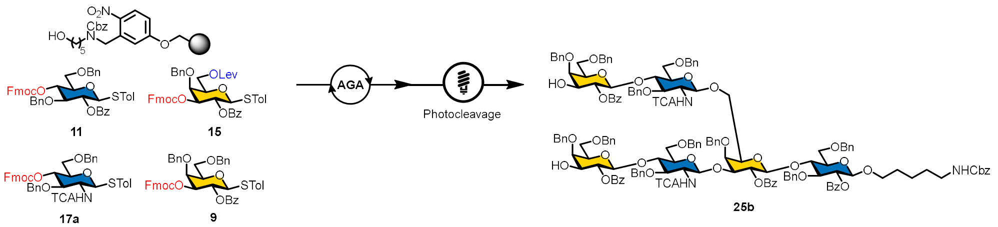

	Building blocks	Modules		Glycosylation condition
AGA		**Initiation** (40 mg resin)		
	**11**	**Acidic wash**<br>**Thioglycoside glycosylation**<br>**Capping**<br>**Fmoc deprotection**		6.5 eq. / t (min)<br>T_1 = -20 °C / 5<br>T_2 = 0 °C / 20
	**15**	**Acidic wash**<br>**Thioglycoside glycosylation**<br>**Capping**<br>**Lev deprotection**<br>**Fmoc deprotection**		6.5 eq. / t (min)<br>T_1 = -20 °C / 5<br>T_2 = 0 °C / 20
	**17a**	**Acidic wash**<br>**Thioglycoside glycosylation**<br>**Pyridine wash**<br>**Capping**<br>**Fmoc deprotection**	**x2** (Acidic wash – Pyridine wash)	6.5 eq. / t (min)<br>T_1 = -20 °C / 5<br>T_2 = 0 °C / 20
	**9**	**Acidic wash**<br>**Thioglycoside glycosylation**<br>**Pyridine wash**<br>**Capping**<br>**Fmoc deprotection**	**x2** (Acidic wash – Pyridine wash)	6.5 eq. / t (min)<br>T_1 = -40 °C / 5<br>T_2 = -20 °C / 20
Post AGA		**Photocleavage**<br>**NP-HPLC Purification**		

Protected **25b** (6.1 mg, 2.1 µmol, 16%) was obtained as a colorless syrup by purification using preparative NP-HPLC (**Method C**).

Analytical NP-HPLC of the crude **25b** (**Method A**, ELSD trace, t_R = 30.1 min)

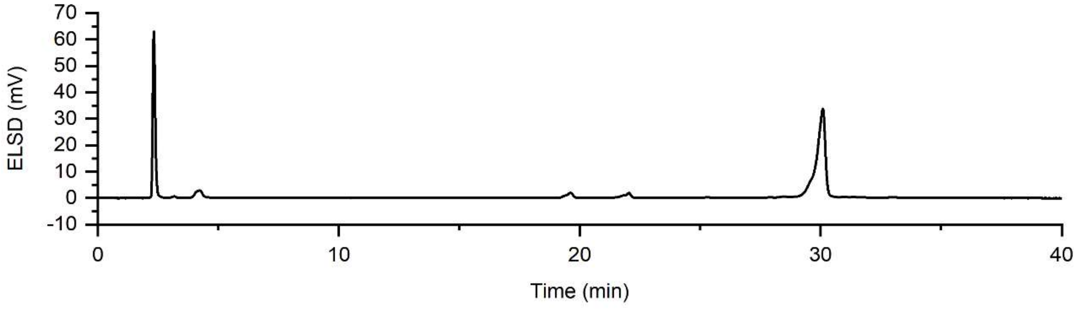

^{1}H NMR (600 MHz, $CDCl_3$) δ 8.03 – 7.95 (m, 5H), 7.90 – 7.84 (m, 2H), 7.61 – 7.55 (m, 3H), 7.55 – 7.50 (m, 4H), 7.45 (q, *J* = 6.8 Hz, 5H), 7.40 (t, *J* = 7.7 Hz, 3H), 7.37 – 7.27 (m, 32H), 7.27 – 7.20 (m, 10H), 7.20 – 7.10 (m, 11H), 6.82 (d, *J* = 7.5 Hz, 2H), 6.51 – 6.44 (m, 2H), 6.26 (t, *J* = 7.4 Hz, 1H), 5.47 (dd, *J* = 10.2, 7.9 Hz, 1H), 5.26 (dd, *J* = 10.0, 7.9 Hz, 1H), 5.20 (dd, *J* = 10.0, 7.9 Hz, 1H), 5.15 (dd, *J* = 9.9, 8.0 Hz, 1H), 5.06 – 5.01 (m, 3H), 4.97 (d, *J* = 10.8 Hz, 1H), 4.95 (d, *J* = 11.6 Hz, 1H), 4.92 (d, *J* = 10.7 Hz, 1H), 4.75 – 4.70 (m, 2H), 4.70 – 4.65 (m, 4H), 4.64 (d, *J* = 11.8 Hz, 1H), 4.60 (d, *J* = 11.4 Hz, 1H), 4.57 (d, *J* = 11.4 Hz, 1H), 4.56 – 4.52 (m, 1H), 4.50 (d, *J* = 11.8 Hz, 2H), 4.48 (d, *J* = 10.7 Hz, 1H), 4.46 (d, *J* = 12.3 Hz, 1H), 4.43 (d, *J* = 11.7 Hz, 1H), 4.40 – 4.34 (m, 4H), 4.32 (d, *J* = 12.2 Hz, 1H), 4.30 – 4.25 (m, 3H), 4.20 (d, *J* = 12.4 Hz, 1H), 4.02 (t, *J* = 8.2 Hz, 1H), 3.95 (t, *J* = 8.8 Hz, 1H), 3.92 – 3.85 (m, 4H), 3.84 (d, *J* = 3.0 Hz, 1H), 3.80 (d, *J* = 8.2 Hz, 1H), 3.78 – 3.71 (m, 3H), 3.67 – 3.55 (m, 7H), 3.55 – 3.47 (m, 5H), 3.46 (d, *J* = 9.3 Hz, 1H), 3.44 – 3.39 (m, 3H), 3.39 – 3.32 (m, 5H), 3.30 (dd, *J* = 10.9, 1.8 Hz, 1H), 3.18 (dd, *J* = 11.1, 2.2 Hz, 1H), 3.15 (ddd, *J* = 9.8, 4.1, 2.0 Hz, 1H), 2.95 (q, *J* = 6.7 Hz, 2H), 2.23 (d, *J* = 9.4 Hz, 2H), 1.51 – 1.39 (m, 2H), 1.37 – 1.29 (m, 2H), 1.23 – 1.15 (m, 2H).

^{13}C NMR (151 MHz, $CDCl_3$) δ 166.4, 166.2, 165.8, 164.9, 162.4, 161.7, 156.4, 139.2, 138.9, 138.7, 138.6, 138.4, 138.3, 138.3, 138.2, 137.9, 137.9, 137.8, 136.8, 133.7, 133.6, 133.6, 133.5, 133.4, 130.1, 130.0, 129.9, 129.9, 129.8, 129.7, 129.7, 128.8, 128.8, 128.7, 128.6, 128.6, 128.6, 128.5, 128.5, 128.4, 128.4, 128.2, 128.2, 128.1, 128.1, 128.1, 128.0, 128.0, 128.0, 127.9, 127.9, 127.8, 127.8, 127.5, 127.5, 127.1, 126.9, 101.1 (**C_1**), 100.8 (**C_1**), 100.7 (**C_1**), 100.4 (**C_1**), 100.0 (**C_1**), 92.9, 92.1, 81.6, 79.3, 79.3, 77.9, 77.2, 76.6, 76.5, 76.4, 76.1, 75.8, 75.6, 75.5, 75.1, 74.8, 74.5, 74.5, 74.4, 74.2, 74.0, 73.8, 73.7, 73.6, 73.6, 73.5, 73.4, 73.3, 73.0, 72.9, 72.8, 72.1, 69.3, 68.3, 68.0, 67.7, 67.7, 66.6, 57.9, 56.9, 41.0, 29.9, 29.6, 29.1, 23.2.

HRMS (QToF): Calcd for $C_{158}H_{161}Cl_6N_3O_{37}Na$ $[M + Na]^+$ 2924.8838; found 2924.8955.

^{1}H NMR (600 MHz, $CDCl_3$) of **25b**:

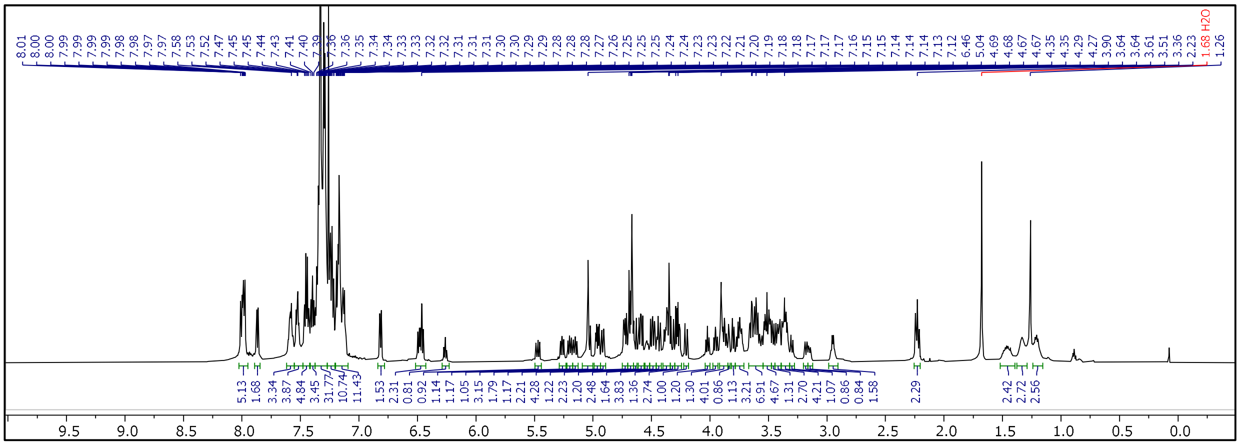

^{13}C NMR (151 MHz, $CDCl_3$) of **25b**:

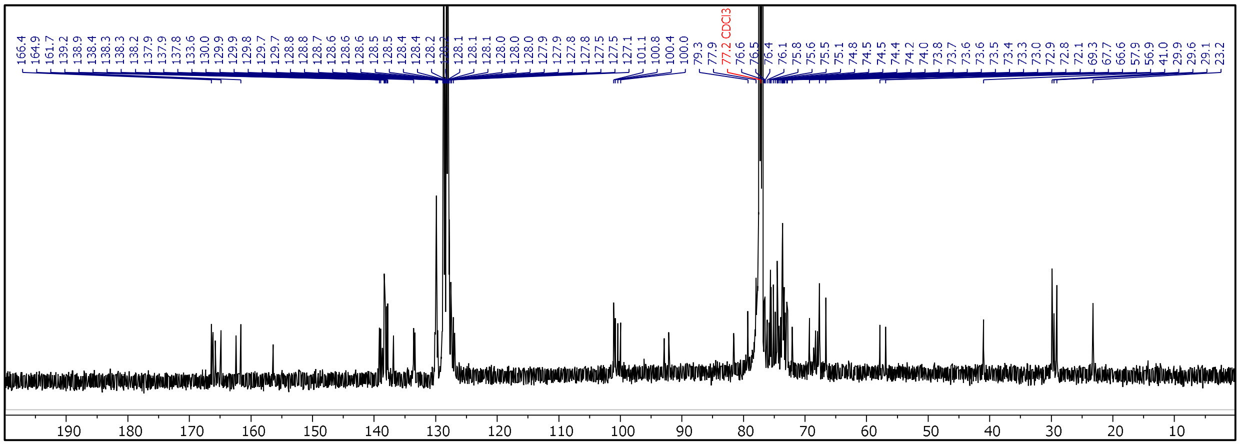

5.9.3 Cbz-protected Lacto-*N*-neohexaose (**25c**)

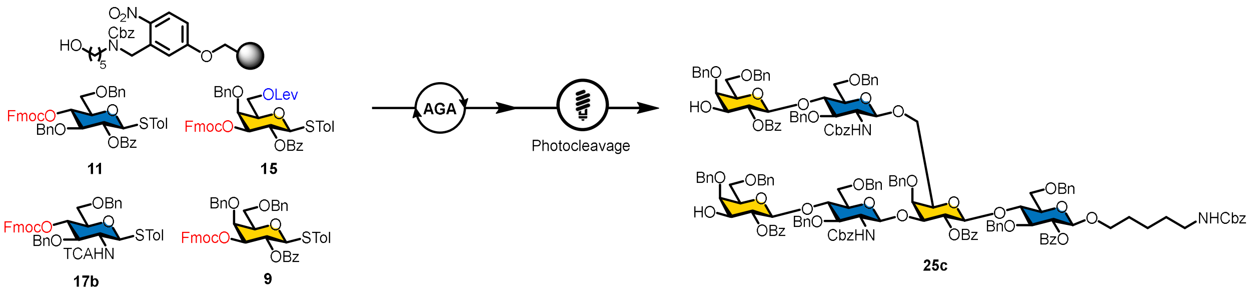

	Building blocks	Modules	Glycosylation condition
		**Initiation** (40 mg resin)	
**AGA**	**11**	**Acidic wash**<br>**Thioglycoside glycosylation**<br>**Capping**<br>**Fmoc deprotection**	**6.5 eq.** / **t (min)**<br>**T_1 = -20 °C** / **5**<br>**T_2 = 0 °C** / **20**
	**15**	**Acidic wash**<br>**Thioglycoside glycosylation**<br>**Capping**<br>**Lev deprotection**<br>**Fmoc deprotection**	**6.5 eq.** / **t (min)**<br>**T_1 = -20 °C** / **5**<br>**T_2 = 0 °C** / **20**
	**17b**	**Acidic wash**<br>**Thioglycoside glycosylation**<br>**Pyridine wash** (Acidic wash to Pyridine wash **x4**)<br>**Capping**<br>**Fmoc deprotection**	**6.5 eq.** / **t (min)**<br>**T_1 = -20 °C** / **5**<br>**T_2 = 0 °C** / **20**
	**9**	**Acidic wash**<br>**Thioglycoside glycosylation**<br>**Pyridine wash** (Acidic wash to Pyridine wash **x2**)<br>**Capping**<br>**Fmoc deprotection**	**6.5 eq.** / **t (min)**<br>**T_1 = -40 °C** / **5**<br>**T_2 = -20 °C** / **20**
**Post AGA**		**Photocleavage**<br>**NP-HPLC Purification**	

Protected **25c** (2.3 mg, 3.8 μmol, 6%) was obtained as a colorless syrup by purification using preparative NP-HPLC (**Method C**).

Analytical NP-HPLC of the crude Protected **25c** (**Method A**, ELSD trace, t_R = 37.1 min)

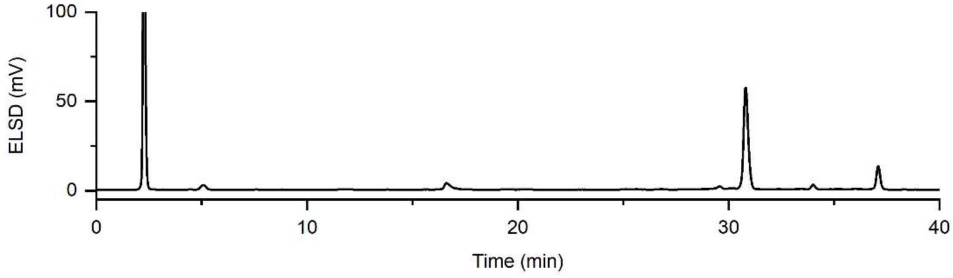

^{1}H NMR (700 MHz, CDCl$_3$) δ 8.05 – 7.91 (m, 6H), 7.92 – 7.82 (m, 2H), 7.64 – 7.49 (m, 4H), 7.49 – 7.37 (m, 9H), 7.38 – 7.19 (m, 44H), 7.19 – 7.04 (m, 19H), 7.02 – 6.90 (m, 3H), 6.60 (t, *J* = 7.6 Hz, 2H), 6.23 (t, *J* = 7.5 Hz, 1H), 5.76 (t, *J* = 8.9 Hz, 1H), 5.66 (d, *J* = 10.1 Hz, 1H), 5.53 (dd, *J* = 10.1, 8.0 Hz, 1H), 5.24 – 5.15 (m, 3H), 5.10 (d, *J* = 10.7 Hz, 1H), 5.07 – 4.94 (m, 4H), 4.84 (d, *J* = 10.7 Hz, 1H), 4.79 – 4.49 (m, 14H), 4.48 – 4.18 (m, 15H), 4.14 (d, *J* = 10.3 Hz, 1H), 3.97 – 3.83 (m, 3H), 3.79 (s, 1H), 3.75 – 3.67 (m, 3H), 3.66 – 3.47 (m, 12H), 3.46 – 3.36 (m, 6H), 3.36 – 3.26 (m, 4H), 3.24 (d, *J* = 10.7 Hz, 1H), 3.18 – 3.09 (m, 1H), 2.93 (d, *J* = 8.7 Hz, 1H), 2.86 – 2.74 (m, 2H), 2.70 (t, *J* = 9.6 Hz, 1H), 2.22 (s, 1H), 1.96 (d, *J* = 10.1 Hz, 1H), 1.34 – 1.12 (m, 4H), 1.10 – 0.98 (m, 2H).

^{13}C NMR (176 MHz, CDCl$_3$) δ 132.83, 132.51, 129.28, 129.12, 127.99, 127.83, 127.83, 127.67, 127.51, 127.35, 127.35, 127.19, 127.03, 126.87, 102.52, 101.23, 101.07, 100.42, 100.26, 99.62, 99.29, 82.36, 80.27, 80.27, 77.53, 76.24, 76.07, 75.91, 75.91, 75.75, 75.59, 74.95, 74.79, 74.14, 74.14, 74.14, 74.14, 74.14, 73.98, 73.66, 72.85, 72.85, 72.85, 72.85, 72.85, 72.69, 72.53, 72.53, 72.37, 72.21, 71.40, 69.79, 69.79, 68.50, 68.34, 67.85, 67.21, 67.05, 67.05, 65.92, 65.43, 65.43, 65.43, 57.53, 55.92, 40.28, 29.15, 28.83, 28.51, 22.54

HRMS (QToF): Calcd for $C_{170}H_{173}N_5O_{43}Na$ [M + Na$_2$]$^{2+}$ 1465.0872; found 1465.0835.

^{1}H NMR (700 MHz, CDCl$_3$) of **25c**:

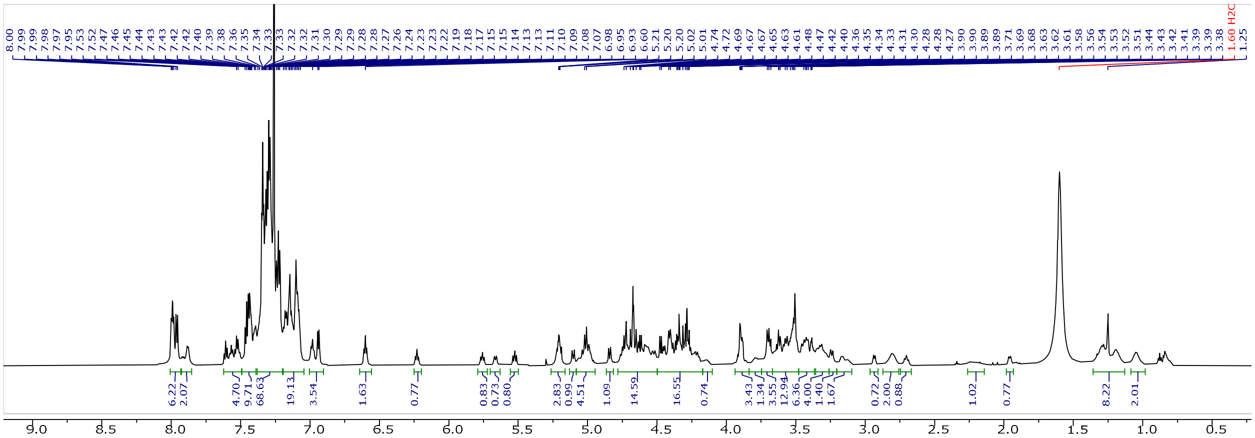

HSQC NMR of **25c**:

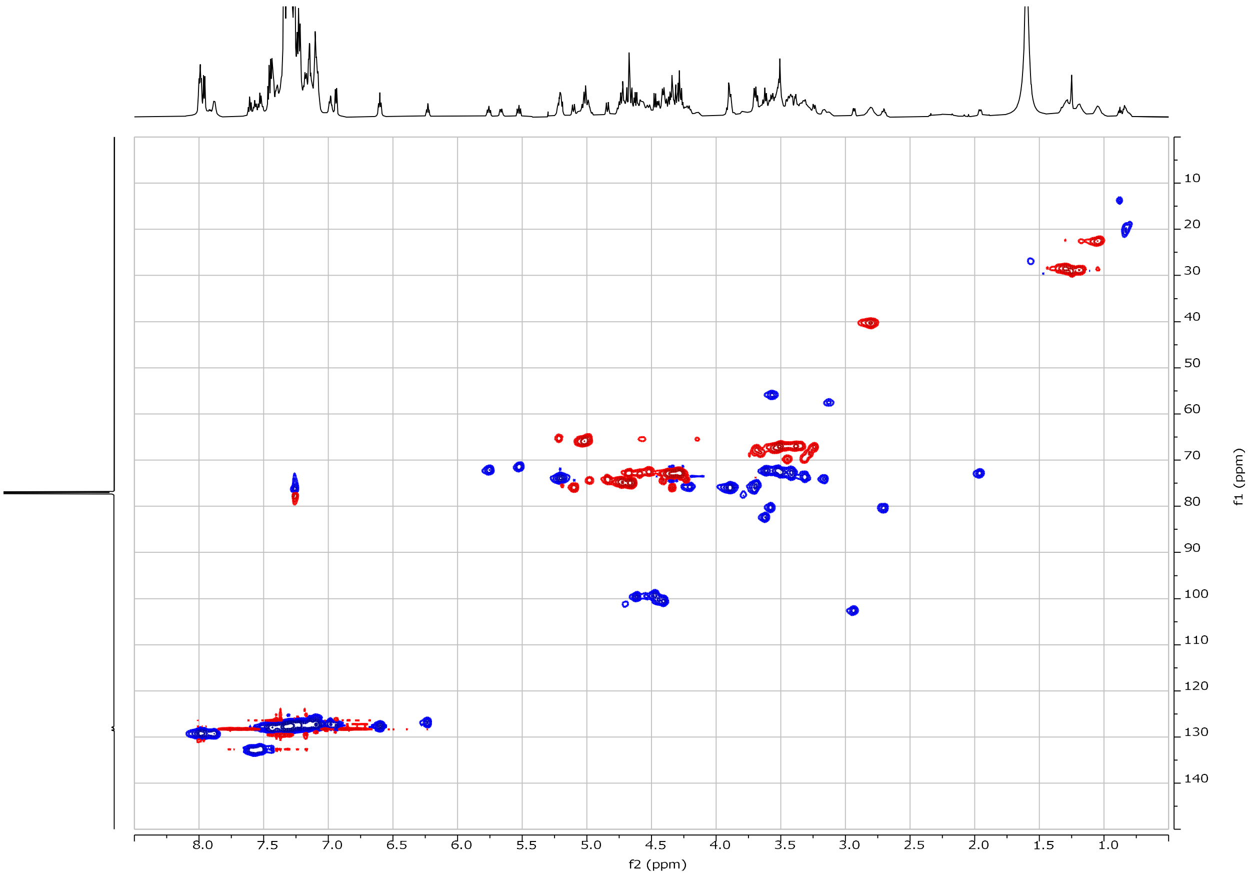

5.10 Protected Difucosyllacto-*N*-neohexaose (**26**)

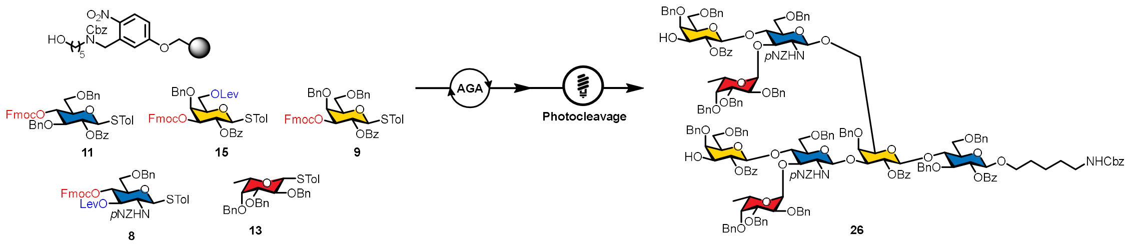

	Building blocks	Modules	Glycosylation condition
AGA		**Initiation** (40 mg resin)	
AGA	11	Acidic wash<br>Thioglycoside glycosylation<br>Capping<br>Fmoc deprotection	6.5 eq. — t (min)<br>T_1 = -20 °C — 5<br>T_2 = 0 °C — 20
AGA	15	Acidic wash<br>Thioglycoside glycosylation<br>Capping<br>Lev deprotection<br>Fmoc deprotection	6.5 eq. — t (min)<br>T_1 = -20 °C — 5<br>T_2 = 0 °C — 20
AGA	8	Acidic wash<br>Thioglycoside glycosylation<br>Pyridine wash<br>(Acidic wash to Pyridine wash x4)<br>Capping<br>Lev deprotection	6.5 eq. — t (min)<br>T_1 = 0 °C — 30
AGA	13	Acidic wash<br>Thioglycoside glycosylation<br>Pyridine wash<br>(Acidic wash to Pyridine wash x2)<br>Capping<br>Fmoc deprotection	10.0 eq. — t (min)<br>T_1 = -40 °C — 5<br>T_2 = -20 °C — 20
AGA	9	Acidic wash<br>Thioglycoside glycosylation<br>Pyridine wash<br>(Acidic wash to Pyridine wash x2)<br>Capping<br>Fmoc deprotection	10.0 eq. — t (min)<br>T_1 = -40 °C — 5<br>T_2 = -20 °C — 20
Post AGA		Photocleavage<br>NP-HPLC Purification	

Protected **26** (9.09 mg, 2.5 µmol, 19%) was obtained as a colorless syrup by purification using preparative NP-HPLC (**Method D**).

Analytical NP-HPLC of the crude **26** (**Method B**, ELSD trace, t_R = 27.6 min)

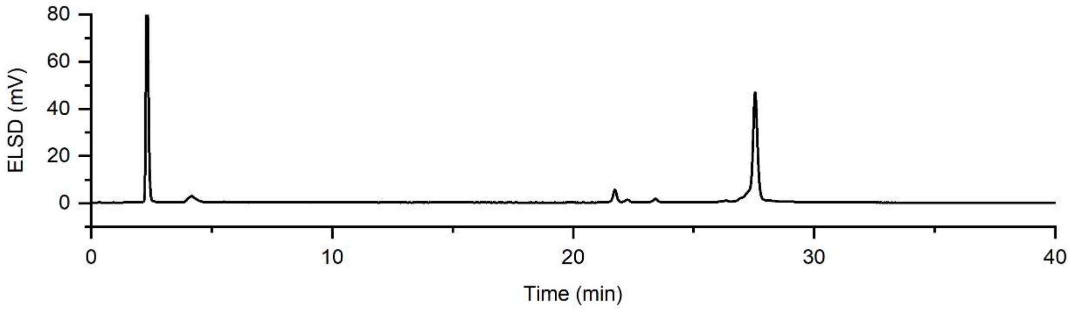

^{1}H NMR (600 MHz, $CDCl_3$) δ 8.10 – 8.01 (m, 5H), 7.98 (d, *J* = 7.8 Hz, 2H), 7.76 (d, *J* = 7.8 Hz, 2H), 7.69 (t, *J* = 7.5 Hz, 1H), 7.59 (d, *J* = 8.1 Hz, 2H), 7.54 (t, *J* = 7.7 Hz, 3H), 7.50 – 7.43 (m, 4H), 7.41 – 7.27 (m, 36H), 7.27 – 7.16 (m, 32H), 7.15 – 7.04 (m, 16H), 6.99 – 6.94 (m, 3H), 6.89 (d, *J* = 7.6 Hz, 1H), 6.47 (t, *J* = 7.6 Hz, 1H), 6.10 (t, *J* = 7.5 Hz, 1H), 5.88 (d, *J* = 10.7 Hz, 1H), 5.76 (dd, *J* = 92.6, 9.0 Hz, 1H), 5.61 (d, *J* = 3.8 Hz, 1H), 5.53 (t, *J* = 9.0 Hz, 1H), 5.22 – 5.11 (m, 3H), 5.09 – 4.95 (m, 5H), 4.88 (s, 1H), 4.84 – 4.77 (m, 3H), 4.76 – 4.63 (m, 8H), 4.63 – 4.52 (m, 8H), 4.51 – 4.45 (m, 4H), 4.45 – 4.36 (m, 5H), 4.36 – 4.28 (m, 4H), 4.29 – 4.21 (m, 3H), 4.21 – 4.09 (m, 4H), 4.07 (d, *J* = 11.1 Hz, 1H), 4.04 – 3.98 (m, 2H), 3.97 – 3.87 (m, 5H), 3.86 – 3.78 (m, 3H), 3.78 – 3.51 (m, 14H), 3.51 – 3.19 (m, 12H), 3.17 – 3.05 (m, 2H), 2.96 – 2.84 (m, 1H), 2.83 – 2.73 (m, 2H), 2.30 (d, *J* = 9.0 Hz, 1H), 2.19 (d, *J* = 9.8 Hz, 1H), 1.93 (d, *J* = 10.0 Hz, 1H), 1.36 – 1.08 (m, 12H).

^{13}C NMR (151 MHz, $CDCl_3$) δ 166.4, 166.1, 165.8, 165.1, 156.9, 156.4, 154.5, 147.5, 146.9, 144.6, 143.6, 140.1, 140.0, 139.17, 139.15, 139.08, 138.8, 138.6, 138.4, 138.2, 138.2, 137.9, 137.74, 137.68, 136.9, 136.8, 133.6, 133.5, 133.4, 133.0, 130.5, 129.98, 129.95, 129.8, 129.7, 129.3, 128.9, 128.8, 128.7, 128.7, 128.7, 128.6, 128.6, 128.6, 128.6, 128.5, 128.5, 128.44, 128.40, 128.3, 128.3, 128.2, 128.2, 128.2, 128.1, 128.1, 128.1, 128.0, 128.0, 127.9, 127.9, 127.8, 127.7, 127.7, 127.7, 127.6, 127.6, 127.5, 127.4, 127.2, 127.1, 126.9, 126.7, 123.6, 123.2, 103.8, 101.6, 101.5, 99.5, 99.3, 97.4, 96.6, 83.2, 80.3, 79.8, 78.9, 78.5, 77.2, 76.42, 76.36, 76.3, 76.1, 75.9, 75.6, 75.4, 75.1, 75.0, 74.7, 74.4, 74.3, 74.2, 73.9, 73.9, 73.63, 73.61, 73.50, 73.47, 73.4, 73.31, 73.28, 73.2, 73.1, 72.9, 72.7, 72.6, 72.0, 70.7, 69.9, 67.5, 66.64, 66.59, 65.9, 65.0, 64.6, 57.7, 41.0, 29.8, 29.6, 29.3, 23.1, 16.9, 16.6.

HRMS (QToF): Calcd for $C_{210}H_{217}N_5O_{51}Na$ [M + 2Na]$^{2+}$ 1835.2162; found 1835.2229.

^{1}H NMR (600 MHz, $CDCl_3$) of **26**:

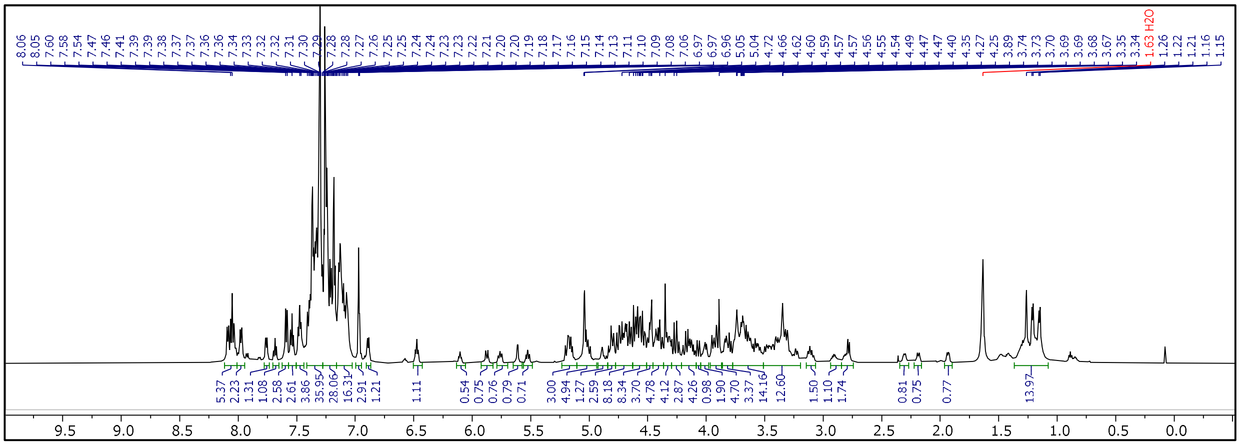

^{13}C NMR (151 MHz, $CDCl_3$) of **26**:

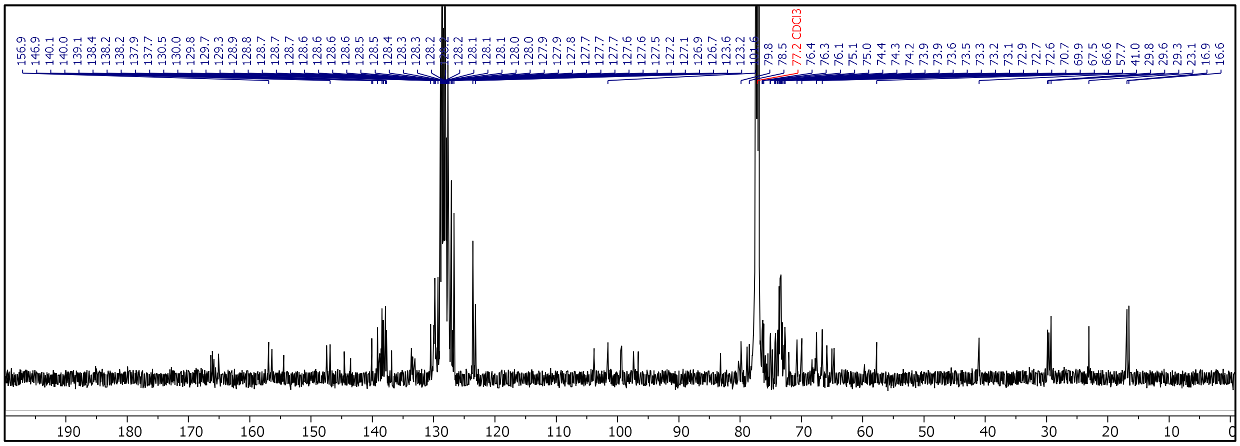

5.11 Protected *iso*-Lacto-*N*-decaose (**27**)

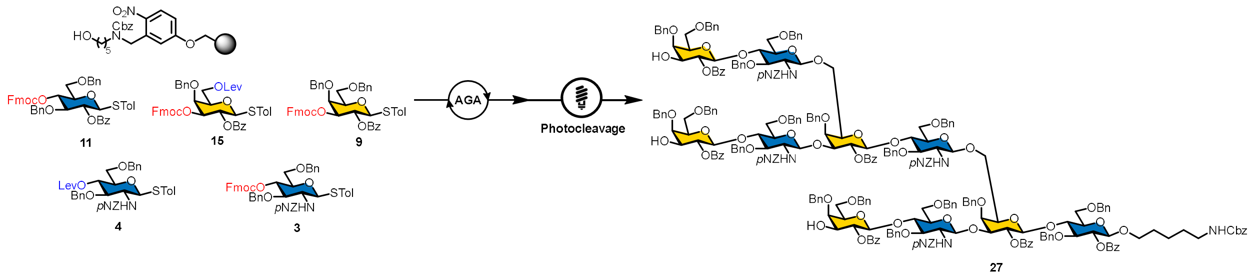

	Building blocks	Modules	Glycosylation condition
AGA		**Initiation** (40 mg resin)	
	**11**	**Acidic wash**<br>**Thioglycoside glycosylation**<br>**Capping**<br>**Fmoc deprotection**	**6.5 eq.** — t (min)<br>T_1 = -20 °C — 5<br>T_2 = 0 °C — 20
	**15**	**Acidic wash**<br>**Thioglycoside glycosylation**<br>**Capping**<br>**Lev deprotection**	**6.5 eq.** — t (min)<br>T_1 = -20 °C — 5<br>T_2 = 0 °C — 20
	**4**	**Acidic wash**<br>**Thioglycoside glycosylation**<br>**Pyridine wash** (Acidic wash to Pyridine wash **x2**)<br>**Capping**<br>**Lev deprotection**	**6.5 eq.** — t (min)<br>T_1 = -10 °C — 30
	**15**	**Acidic wash**<br>**Thioglycoside glycosylation**<br>**Capping**<br>**Lev deprotection**<br>**Fmoc deprotection**	**6.5 eq.** — t (min)<br>T_1 = -20 °C — 5<br>T_2 = 0 °C — 20
	**3**	**Acidic wash**<br>**Thioglycoside glycosylation**<br>**Pyridine wash** (Acidic wash to Pyridine wash **x6**)<br>**Capping**<br>**Fmoc deprotection**	**6.5 eq.** — t (min)<br>T_1 = 0 °C — 30
	**9**	**Acidic wash**<br>**Thioglycoside glycosylation**<br>**Pyridine wash** (Acidic wash to Pyridine wash **x3**)<br>**Capping**<br>**Fmoc deprotection**	**10.0 eq.** — t (min)<br>T_1 = -40 °C — 5<br>T_2 = -20 °C — 20
**Post AGA**		**Photocleavage**<br>**NP-HPLC Purification**	

Protected **27** (10.2 mg, 2.1 µmol, 16%) was obtained as a colorless syrup by purification using preparative NP-HPLC (**Method D**).

Analytical NP-HPLC of the crude **27** (**Method B**, ELSD trace, t_R = 30.5 min)

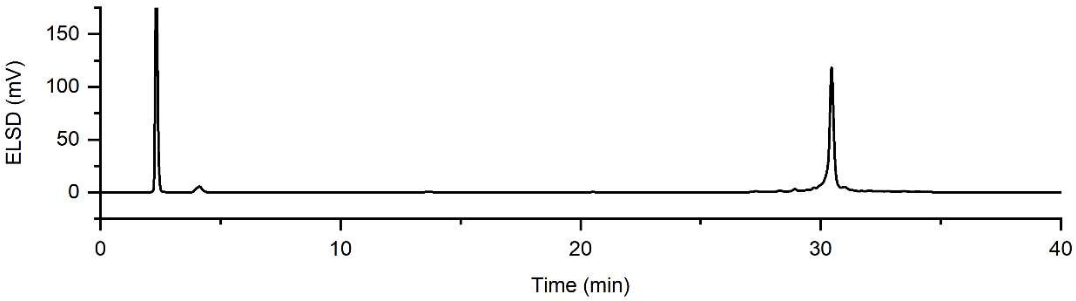

^{1}H NMR (600 MHz, $CDCl_3$) δ 8.13 – 7.93 (m, 12H), 7.90 – 7.78 (m, 5H), 7.69 (d, *J* = 8.5 Hz, 2H), 7.64 – 7.53 (m, 6H), 7.54 – 7.36 (m, 15H), 7.35 – 7.17 (m, 68H), 7.15 – 7.08 (m, 22H), 6.97 (t, *J* = 7.7 Hz, 2H), 6.94 (d, *J* = 6.5 Hz, 3H), 6.70 – 6.64 (m, 2H), 6.58 (t, *J* = 7.5 Hz, 2H), 6.35 – 6.28 (m, 1H), 6.21 (t, *J* = 7.1 Hz, 1H), 5.80 – 5.74 (m, 2H), 5.71 (d, *J* = 8.5 Hz, 1H), 5.55 (t, *J* = 7.8 Hz, 1H), 5.50 (t, *J* = 8.8 Hz, 1H), 5.24 – 5.07 (m, 6H), 5.06 – 4.94 (m, 5H), 4.94 – 4.87 (m, 2H), 4.86 – 4.81 (m, 2H), 4.72 – 4.55 (m, 16H), 4.54 – 4.49 (m, 3H), 4.48 – 4.15 (m, 24H), 4.04 – 3.97 (m, 2H), 3.94 (d, *J* = 9.3 Hz, 2H), 3.91 – 3.83 (m, 4H), 3.78 – 3.04 (m, 44H), 2.97 – 2.91 (m, 2H), 2.87 – 2.76 (m, 3H), 2.73 (t, *J* = 9.4 Hz, 1H), 2.64 – 2.57 (m, 1H), 2.33 (d, *J* = 9.9 Hz, 1H), 2.27 – 2.20 (m, 1H), 2.03 – 1.99 (m, 1H), 1.89 (d, *J* = 9.5 Hz, 1H), 1.82 (d, *J* = 8.8 Hz, 1H), 1.38 – 1.15 (m, 2H), 1.09 – 0.95 (m, 2H), 0.88 (t, *J* = 7.0 Hz, 1H), 0.86 – 0.81 (m, 1H).

^{13}C NMR (151 MHz, $CDCl_3$) δ 166.3, 166.2, 165.6, 165.3, 164.7, 164.7, 164.5, 156.4, 156.3, 156.2, 156.0, 154.7, 147.3, 147.3, 146.6, 146.2, 145.3, 144.0, 144.0, 139.3, 139.2, 138.7, 138.5, 138.4, 138.3, 138.1, 138.0, 137.9, 137.8, 137.8, 137.7, 137.6, 136.7, 133.5, 133.5, 133.3, 133.3, 133.2, 133.1, 129.9, 129.7, 129.7, 129.7, 129.6, 129.5, 128.8, 128.6, 128.6, 128.5, 128.5, 128.3, 128.2, 128.0, 127.9, 127.9, 127.8, 127.8, 127.8, 127.7, 127.7, 127.6, 127.5, 127.3, 127.3, 127.0, 123.5, 123.5, 123.3, 122.9, 102.9, 102.9, 101.3, 100.8, 100.3, 100.3, 100.1, 100.0, 83.0, 82.8, 81.1, 81.0, 77.0, 76.4, 76.4, 75.5, 75.5, 74.9, 74.8, 74.4, 74.3, 74.0, 74.0, 73.6, 73.5, 73.3, 73.3, 72.9, 72.8, 72.7, 72.7, 72.2, 72.1, 68.1, 67.8, 67.6, 66.4, 64.7, 64.6, 64.0, 57.8, 56.5, 55.8, 40.8, 40.6, 29.7, 29.4, 28.9, 22.9.

HRMS (QToF): Calcd for $C_{273}H_{275}N_9O_{71}Na_2$ $[M + 2Na]^{2+}$ 2430.3985; found 2430.4106.

^{1}H NMR (600 MHz, $CDCl_3$) of **27**:

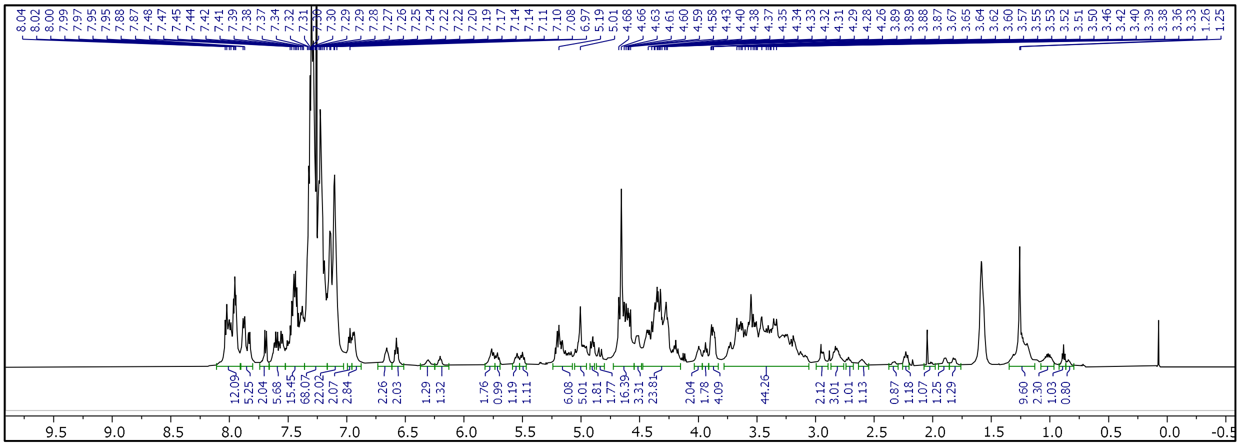

^{13}C NMR (151 MHz, $CDCl_3$) of **27**:

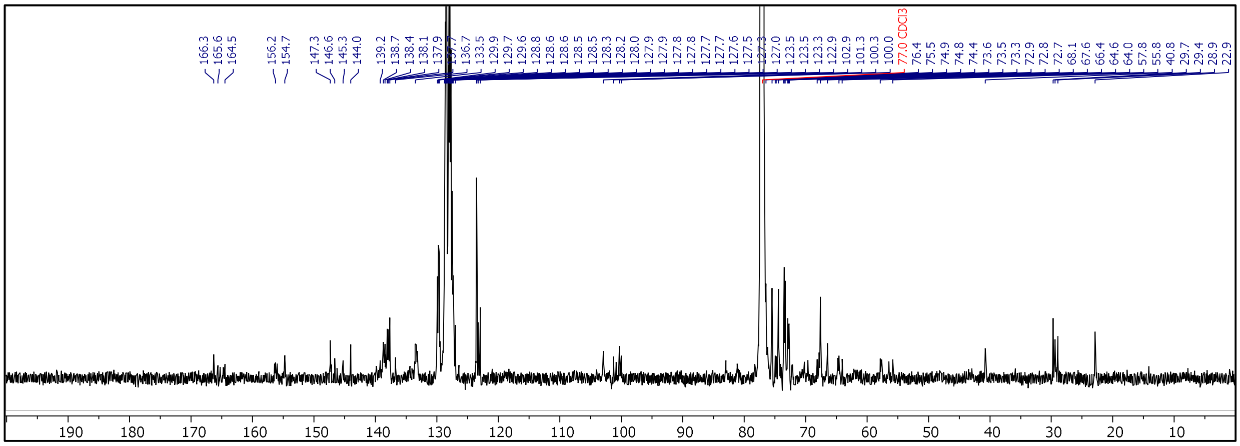

5.12 Protected Fucosyllacto-*N*-neohexaose II (**28**)

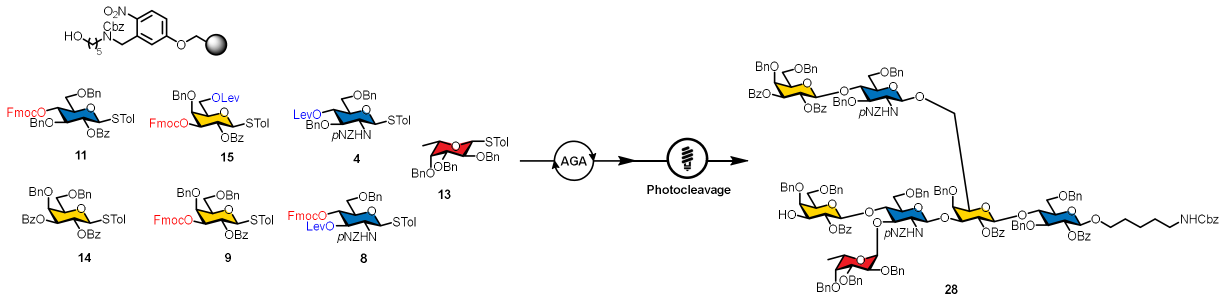

	Building blocks	Modules	Glycosylation condition
AGA		**Initiation** (40 mg resin)	
	**11**	**Acidic wash**<br>**Thioglycoside glycosylation**<br>**Capping**<br>**Fmoc deprotection**	**6.5 eq.** / **t (min)**<br>**T_1 = -20 °C** / **5**<br>**T_2 = 0 °C** / **20**
	**15**	**Acidic wash**<br>**Thioglycoside glycosylation**<br>**Capping**<br>**Lev deprotection**	**6.5 eq.** / **t (min)**<br>**T_1 = -20 °C** / **5**<br>**T_2 = 0 °C** / **20**
	**4**	**Acidic wash** (x2)<br>**Thioglycoside glycosylation** (x2)<br>**Pyridine wash** (x2)<br>**Capping**<br>**Lev deprotection**	**6.5 eq.** / **t (min)**<br>**T_1 = -10 °C** / **30**
	**14**	**Acidic wash**<br>**Thioglycoside glycosylation**<br>**Capping**<br>**Fmoc deprotection**	**6.5 eq.** / **t (min)**<br>**T_1 = -20 °C** / **5**<br>**T_2 = 0 °C** / **20**
	**8**	**Acidic wash** (x2)<br>**Thioglycoside glycosylation** (x2)<br>**Pyridine wash** (x2)<br>**Capping**<br>**Lev deprotection**	**6.5 eq.** / **t (min)**<br>**T_1 = 0 °C** / **30**
	**13**	**Acidic wash**<br>**Thioglycoside glycosylation**<br>**Capping**<br>**Fmoc deprotection**	**8.0 eq.** / **t (min)**<br>**T_1 = -40 °C** / **5**<br>**T_2 = -20 °C** / **20**
	**9**	**Acidic wash**<br>**Thioglycoside glycosylation**<br>**Capping**<br>**Fmoc deprotection**	**6.5 eq.** / **t (min)**<br>**T_1 = -40 °C** / **5**<br>**T_2 = -20 °C** / **20**
**Post AGA**		**Photocleavage**<br>**NP-HPLC Purification**	

Protected **28** (7.63 mg, 2.2 μmol, 17%) was obtained as a colorless syrup by purification using preparative NP-HPLC (**Method D**).

Analytical NP-HPLC of the crude **28** (**Method B**, ELSD trace, t_R = 26.7 min)

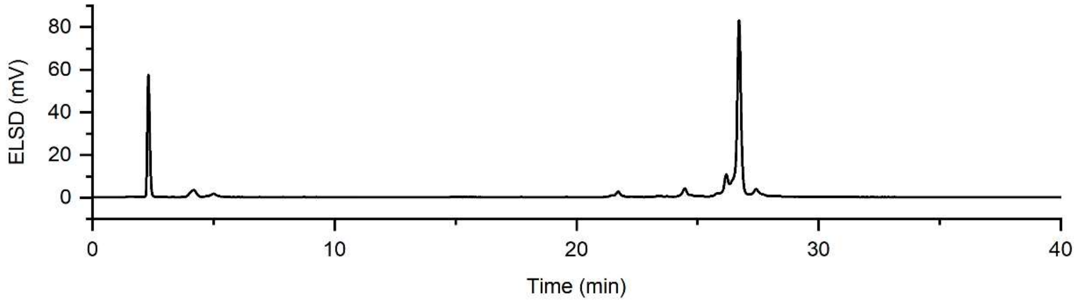

^{1}H NMR (700 MHz, $CDCl_3$) δ 8.02 (t, *J* = 8.5 Hz, 4H), 7.90 (dt, *J* = 14.5, 7.5 Hz, 8H), 7.72 (dd, *J* = 14.2, 8.0 Hz, 4H), 7.56 – 7.50 (m, 4H), 7.47 – 7.23 (m, 72H), 7.23 – 6.95 (m, 7H), 6.87 (t, *J* = 7.6 Hz, 2H), 6.73 (t, *J* = 7.5 Hz, 1H), 6.39 (t, *J* = 7.5 Hz, 1H), 5.78 – 5.67 (m, 3H), 5.48 (t, *J* = 8.0 Hz, 1H), 5.16 – 5.07 (m, 5H), 5.03 – 4.89 (m, 7H), 4.84 (s, 2H), 4.78 – 4.70 (m, 4H), 4.70 – 4.46 (m, 21H), 4.46 – 4.34 (m, 13H), 4.33 – 4.22 (m, 10H), 4.18 (d, *J* = 11.8 Hz, 2H), 4.14 – 4.02 (m, 6H), 3.96 (t, *J* = 9.6 Hz, 1H), 3.90 (d, *J* = 3.6 Hz, 1H), 3.84 (dd, *J* = 11.1, 3.1 Hz, 1H), 3.81 – 3.74 (m, 3H), 3.69 – 3.52 (m, 15H), 3.52 – 3.45 (m, 4H), 3.43 – 3.35 (m, 5H), 3.33 (t, *J* = 9.0 Hz, 3H), 3.30 – 3.26 (m, 4H), 3.23 (q, *J* = 9.8 Hz, 4H), 3.15 (d, *J* = 10.3 Hz, 2H), 3.11 – 3.04 (m, 2H), 2.80 (d, *J* = 8.7 Hz, 1H), 2.76 – 2.71 (m, 2H), 2.63 (t, *J* = 9.6 Hz, 1H), 2.25 (d, *J* = 8.7 Hz, 1H), 1.97 (d, *J* = 9.1 Hz, 1H), 1.31 – 1.15 (m, 6H), 1.15 – 1.00 (m, 5H).

^{13}C NMR (176 MHz, $CDCl_3$) δ 166.4, 165.9, 165.5, 165.12, 165.07, 156.3, 156.0, 154.4, 147.5, 146.9, 145.7, 143.6, 139.5, 139.1, 139.1, 138.7, 138.3, 138.3, 138.2, 138.2, 138.1, 137.7, 137.6, 136.8, 133.6, 133.4, 130.2, 130.0, 129.8, 129.84, 129.82, 129.77, 129.7, 129.6, 129.5, 129.4, 128.9, 128.8, 128.7, 128.7, 128.7, 128.7, 128.6, 128.6, 128.6, 128.6, 128.5, 128.5, 128.4, 128.3, 128.3, 128.2, 128.2, 128.2, 128.2, 128.1, 128.1, 128.0, 128.0, 127.8, 127.8, 127.7, 127.7, 127.6, 127.5, 127.4, 127.3, 127.1, 126.5, 123.6, 123.2, 103.3, 101.3, 100.9, 100.3, 99.4, 97.4, 82.9, 81.3, 80.2, 79.9, 78.5, 77.2, 76.7, 76.3, 76.3, 76.0, 75.8, 75.2, 75.1, 75.0, 74.9, 74.65, 74.63, 74.3, 74.3, 74.2, 74.1, 73.8, 73.6, 73.5, 73.4, 73.0, 72.9, 72.8, 72.7, 72.3, 71.2, 71.1, 69.5, 68.2, 67.7, 67.45, 67.42, 67.3, 66.6, 65.0, 64.4, 59.7, 56.4, 40.8, 32.1, 29.8, 29.6, 29.5, 28.9, 22.9, 19.8, 16.5, 14.3.

HRMS (QToF): Calcd for $C_{197}H_{199}N_5O_4Na$ $[M + 2Na]^{2+}$ 1724.1535; found 1724.1619.

^{1}H NMR (700 MHz, $CDCl_3$) of **28**:

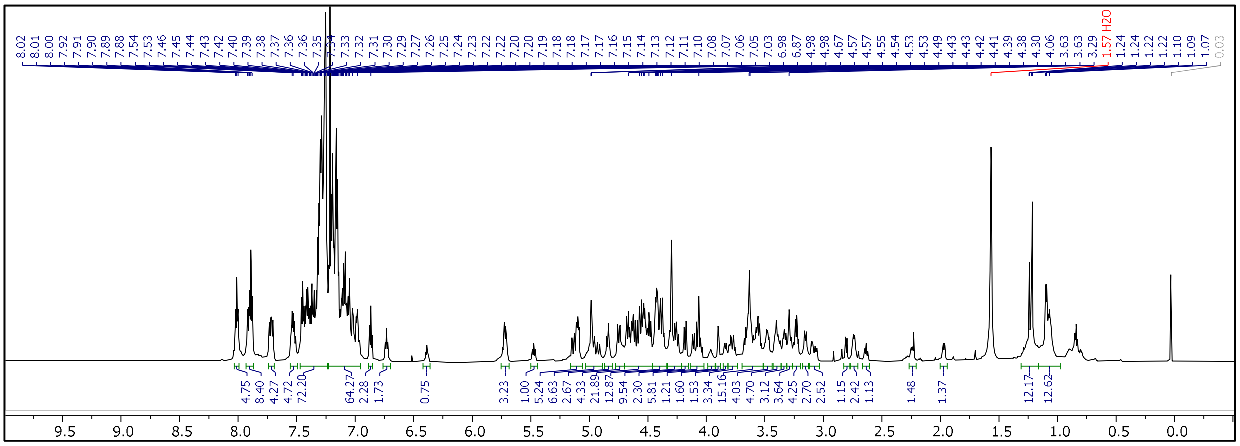

^{13}C NMR (176 MHz, $CDCl_3$) of **28**:

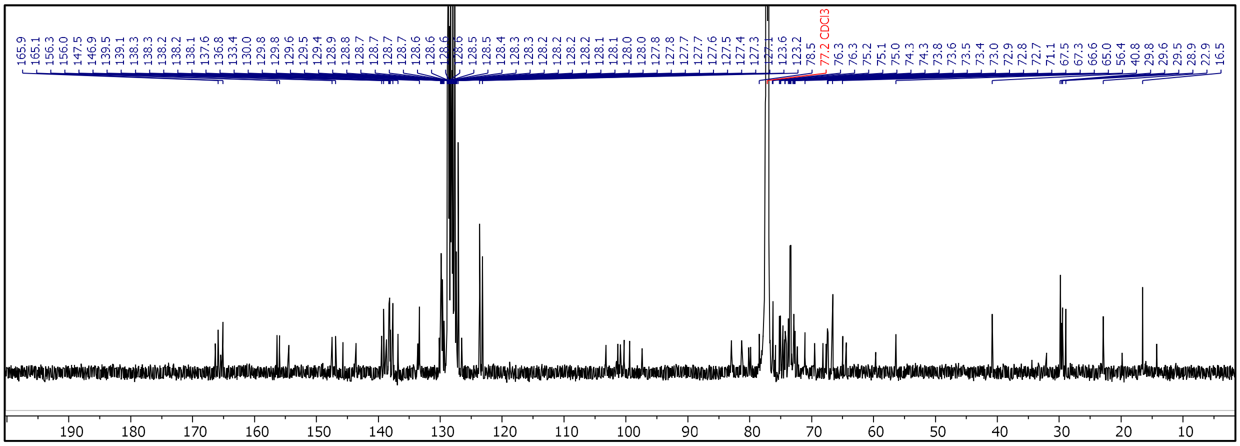

5.13 Protected Lacto-*N*-difuco-hexoase II (**29**)

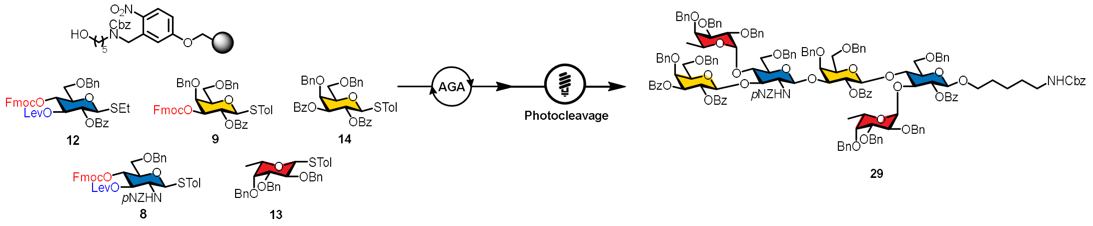

	Building blocks	Modules	Glycosylation condition
AGA		**Initiation** (40 mg resin)	
	**12**	**Acidic wash**<br>**Thioglycoside glycosylation**<br>**Capping**<br>**Lev deprotection**	6.5 eq. — t (min)<br>T_1 = -20 °C — 5<br>T_2 = 0 °C — 20
	**13**	**Acidic wash**<br>**Thioglycoside glycosylation**<br>**Capping**<br>**Fmoc deprotection**	8.0 eq. — t (min)<br>T_1 = -40 °C — 5<br>T_2 = -20 °C — 20
	**9**	**Acidic wash**<br>**Thioglycoside glycosylation**<br>**Capping**<br>**Fmoc deprotection**	6.5 eq. — t (min)<br>T_1 = -40 °C — 5<br>T_2 = -20 °C — 20
	**8**	**Acidic wash** ⎤<br>**Thioglycoside glycosylation** ⎥ **x2**<br>**Pyridine wash** ⎦<br>**Capping**<br>**Lev deprotection**	6.5 eq. — t (min)<br>T_1 = 0 °C — 45
	**14**	**Acidic wash**<br>**Thioglycoside glycosylation**<br>**Capping**<br>**Fmoc deprotection**	6.5 eq. — t (min)<br>T_1 = -20 °C — 10<br>T_2 = 0 °C — 20
	**13**	**Acidic wash**<br>**Thioglycoside glycosylation**<br>**Capping**	8.0 eq. — t (min)<br>T_1 = -40 °C — 5<br>T_2 = -20 °C — 20
Post AGA		**Photocleavage**<br>**NP-HPLC Purification**	

Protected **29** (6.4 mg, 2.2 µmol, 17%) was obtained as a colorless syrup by purification using preparative NP-HPLC (**Method C**).

Analytical NP-HPLC of the crude **29** (**Method A**, ELSD trace, t_R = 30.7 min)

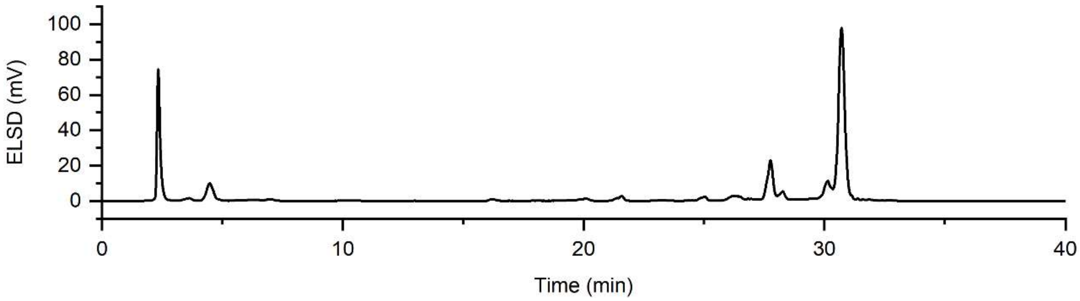

^{1}H NMR (700 MHz, $CDCl_3$) δ 8.32 (d, *J* = 8.3 Hz, 1H), 7.99 (d, *J* = 7.6 Hz, 2H), 7.97 – 7.84 (m, 5H), 7.72 – 7.01 (m, 77H), 6.98 (t, *J* = 7.4 Hz, 2H), 6.91 (d, *J* = 7.4 Hz, 2H), 5.61 (t, *J* = 8.7 Hz, 1H), 5.34 (d, *J* = 8.9 Hz, 1H), 5.29 – 5.24 (m, 1H), 5.20 (d, *J* = 3.4 Hz, 1H), 5.16 – 5.03 (m, 3H), 4.96 (d, *J* = 9.6 Hz, 1H), 4.92 – 4.18 (m, 34H), 4.17 – 3.87 (m, 9H), 3.88 – 3.34 (m, 16H), 3.32 – 3.15 (m, 2H), 3.01 (d, *J* = 9.3 Hz, 1H), 2.95 – 2.87 (m, 1H), 2.86 – 2.77 (m, 1H), 1.48 – 1.37 (m, 2H), 1.35 – 1.15 (m, 8H), 1.15 – 0.99 (m, 2H).

^{13}C NMR (151 MHz, $CDCl_3$) δ 166.0, 165.2, 164.6, 164.5, 156.4, 154.5, 148.2, 143.9, 139.5, 139.4, 139.4, 139.1, 138.7, 138.5, 138.3, 138.2, 138.1, 137.9, 137.9, 137.7, 136.9, 133.6, 133.6, 133.5, 133.3, 130.4, 130.3, 130.1, 130.0, 129.9, 129.8, 129.6, 129.4, 129.3, 129.1, 129.0, 128.9, 128.8, 128.7, 128.7, 128.6, 128.6, 128.6, 128.4, 128.4, 128.3, 128.3, 128.2, 128.2, 128.1, 128.0, 127.9, 127.8, 127.6, 127.5, 127.4, 127.2, 127.1, 124.1, 101.6, 101.1, 100.4, 100.0, 97.8, 97.3, 80.5, 79.9, 79.8, 79.4, 79.3, 79.2, 79.1, 79.0, 78.9, 78.8, 78.6, 78.6, 78.4, 77.4, 77.2, 76.9, 76.5, 76.3, 76.2, 76.1, 76.0, 75.9, 75.8, 75.7, 75.7, 75.6, 75.5, 75.4, 75.4, 75.2, 75.1, 75.0, 74.9, 74.8, 74.7, 74.6, 74.3, 74.2, 74.2, 74.1, 74.0, 73.9, 73.8, 73.8, 73.7, 73.6, 73.5, 73.4, 73.2, 73.2, 73.1, 73.0, 72.9, 72.7, 72.6, 72.5, 72.4, 72.3, 72.2, 72.1, 72.0, 70.2, 69.5, 68.0, 68.0, 67.9, 67.9, 67.8, 67.3, 67.0, 66.8, 66.7, 66.7, 66.6, 66.6, 64.7, 59.7, 40.9, 32.1, 29.9, 29.8, 29.7, 29.6, 29.5, 29.5, 29.4, 29.4, 29.3, 29.0, 28.9, 27.4, 27.3, 23.1, 22.8, 16.7, 16.4, 14.3.

HRMS (QToF): Calcd for $C_{169}H_{173}N_3O_{38}Na$ $[M + Na]^+$ 2875.1595; found 2875.1699.

^{1}H NMR (700 MHz, $CDCl_3$) of **29**:

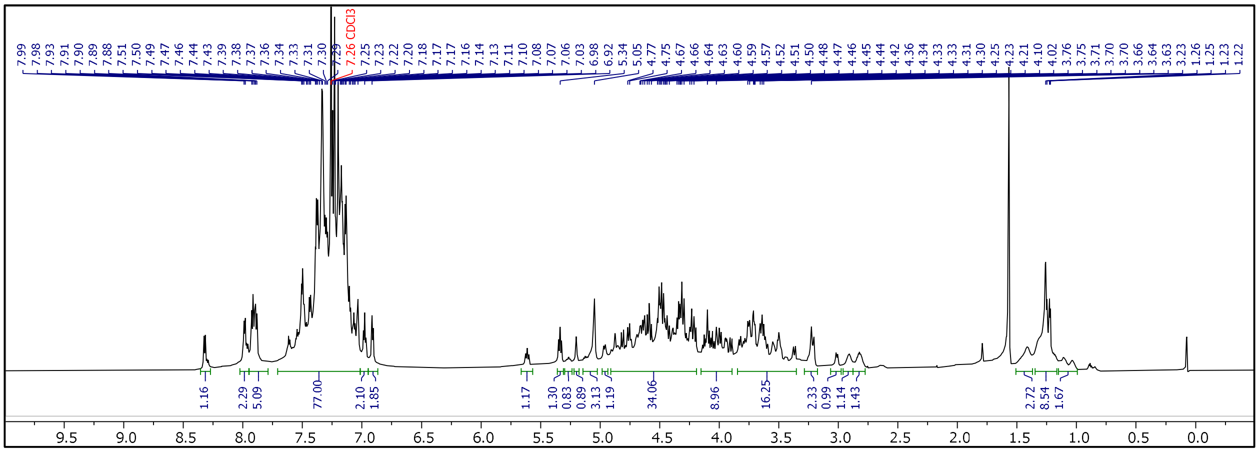

^{13}C NMR (151 MHz, $CDCl_3$) of **29**:

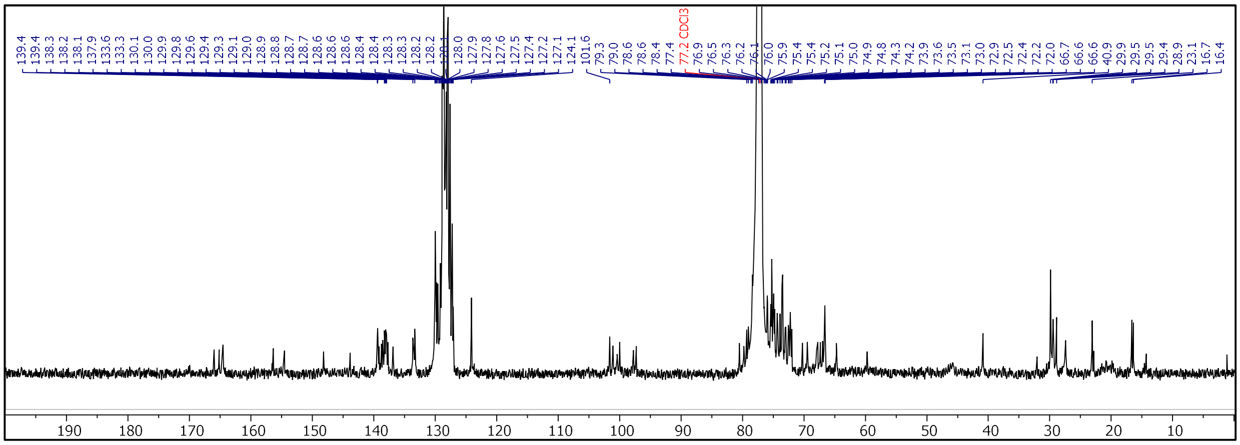

5.14 Protected Lacto-*N*-fucopentaose I (**30**)

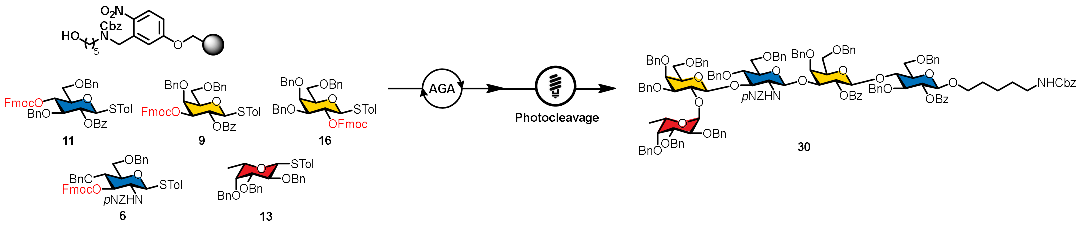

	Building blocks	Modules	Glycosylation condition
AGA		**Initiation** (40 mg resin)	
	**11**	**Acidic wash**<br>**Thioglycoside glycosylation**<br>**Capping**<br>**Fmoc deprotection**	**6.5 eq.** — **t (min)**<br>T_1 = -20 °C — 5<br>T_2 = 0 °C — 20
	**9**	**Acidic wash**<br>**Thioglycoside glycosylation**<br>**Capping**<br>**Fmoc deprotection**	**6.5 eq.** — **t (min)**<br>T_1 = -40 °C — 5<br>T_2 = -20 °C — 20
	**6**	**Acidic wash**<br>**Thioglycoside glycosylation** **x2**<br>**Pyridine wash**<br>**Capping**<br>**Fmoc deprotection**	**6.5 eq.** — **t (min)**<br>T_1 = 0 °C — 30
	**16**	**Acidic wash**<br>**Thioglycoside glycosylation**<br>**Capping**<br>**Fmoc deprotection**	**6.5 eq.** — **t (min)**<br>T_1 = -20 °C — 10<br>T_2 = 0 °C — 20
	**13**	**Acidic wash**<br>**Thioglycoside glycosylation**<br>**Capping**	**8.0 eq.** — **t (min)**<br>T_1 = -40 °C — 5<br>T_2 = -20 °C — 20
Post AGA		**Photocleavage**<br>**NP-HPLC Purification**	

Protected **30** (6.23 mg, 2.6 μmol, 20%) was obtained as a colorless syrup by purification using preparative NP-HPLC (**Method C**).

Analytical NP-HPLC of the crude **30** (**Method A**, ELSD trace, t_R = 27.2 min)

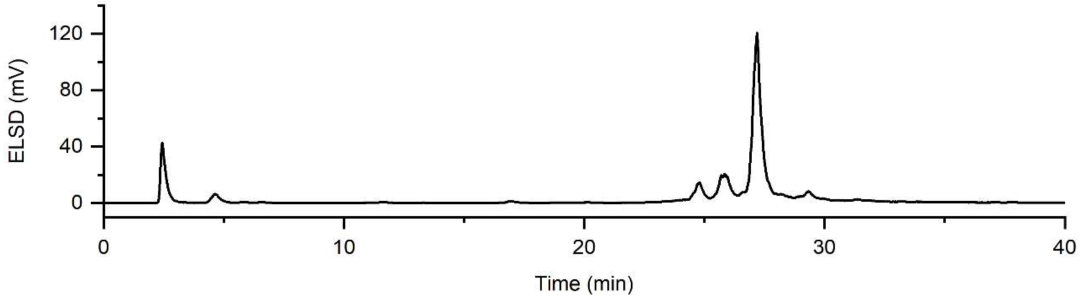

^{1}H NMR (600 MHz, $CDCl_3$) δ 8.04 (d, *J* = 8.4 Hz, 2H), 7.96 (d, *J* = 7.4 Hz, 2H), 7.82 (d, *J* = 7.6 Hz, 2H), 7.51 (q, *J* = 7.1 Hz, 2H), 7.44 – 7.09 (m, 60H), 7.10 – 7.05 (m, 4H), 7.06 – 6.96 (m, 4H), 6.94 (t, *J* = 7.6 Hz, 2H), 6.91 (d, *J* = 7.4 Hz, 1H), 5.60 (d, *J* = 3.6 Hz, 1H), 5.54 (dd, *J* = 10.0, 8.1 Hz, 1H), 5.16 (dd, *J* = 9.3, 8.0 Hz, 1H), 5.12 – 5.03 (m, 3H), 5.00 (d, *J* = 10.1 Hz, 1H), 4.95 – 4.89 (m, 2H), 4.85 – 4.78 (m, 3H), 4.76 – 4.69 (m, 3H), 4.67 (d, *J* = 12.1 Hz, 1H), 4.63 (d, *J* = 12.3 Hz, 1H), 4.60 – 4.45 (m, 9H), 4.44 – 4.30 (m, 8H), 4.26 (d, *J* = 12.0 Hz, 3H), 4.20 (d, *J* = 11.8 Hz, 1H), 4.18 – 4.13 (m, 2H), 4.08 (d, *J* = 2.7 Hz, 1H), 4.02 (t, *J* = 8.9 Hz, 1H), 3.97 (dd, *J* = 10.4, 3.8 Hz, 1H), 3.87 (d, *J* = 3.4 Hz, 1H), 3.84 – 3.79 (m, 2H), 3.77 – 3.63 (m, 5H), 3.61 – 3.33 (m, 11H), 3.33 – 3.27 (m, 1H), 3.24 – 3.17 (m, 2H), 2.91 – 2.84 (m, 2H), 1.51 – 1.35 (m, 4H), 1.16 (d, *J* = 6.5 Hz, 3H), 1.13 – 1.07 (m, 2H).

^{13}C NMR (151 MHz, $CDCl_3$) δ 165.2, 164.8, 156.4, 155.2, 147.4, 144.3, 139.5, 139.4, 139.4, 139.3, 139.2, 138.7, 138.7, 138.6, 138.4, 138.3, 138.3, 138.2, 138.1, 138.1, 136.9, 136.8, 133.5, 133.1, 130.3, 129.9, 129.8, 129.7, 128.9, 128.8, 128.6, 128.6, 128.5, 128.4, 128.3, 128.2, 128.2, 128.0, 128.0, 128.0, 127.9, 127.9, 127.8, 127.7, 127.6, 127.5, 127.4, 127.2, 127.1, 126.2, 123.8, 102.0, 101.3, 101.3, 100.5, 98.3, 83.8, 80.8, 79.5, 79.4, 77.4, 77.2, 76.9, 76.5, 76.4, 76.0, 75.3, 75.1, 75.0, 74.9, 74.6, 73.8, 73.7, 73.6, 73.5, 73.3, 73.2, 73.1, 72.4, 72.2, 71.2, 69.5, 69.4, 68.6, 68.4, 67.8, 66.6, 66.4, 65.1, 58.5, 40.9, 32.1, 29.5, 29.0, 23.2, 22.8, 16.9.

HRMS (QToF): Calcd for $C_{149}H_{155}N_3O_{32}Na$ $[M + Na]^+$ 2521.0491; found 2521.0576.

^{1}H NMR (600 MHz, $CDCl_3$) of **30**:

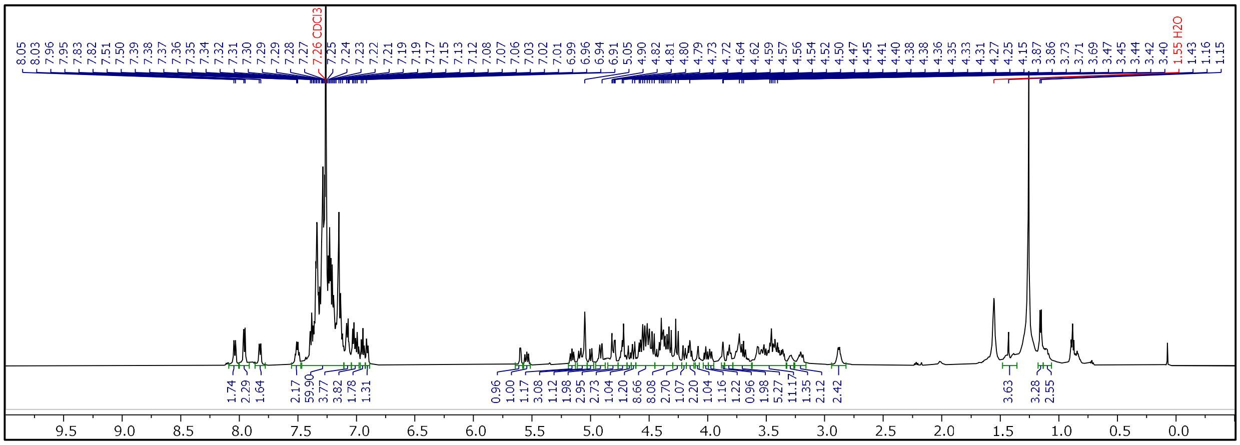

^{13}C NMR (151 MHz, $CDCl_3$) of **30**:

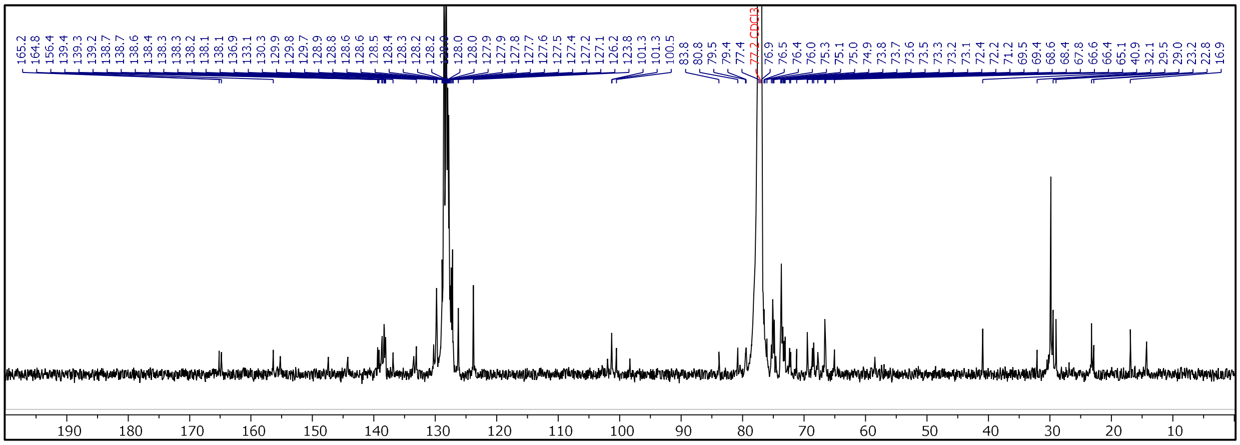

5.15 Protected Lacto-*N*-difuco-hexoase I (**31**)

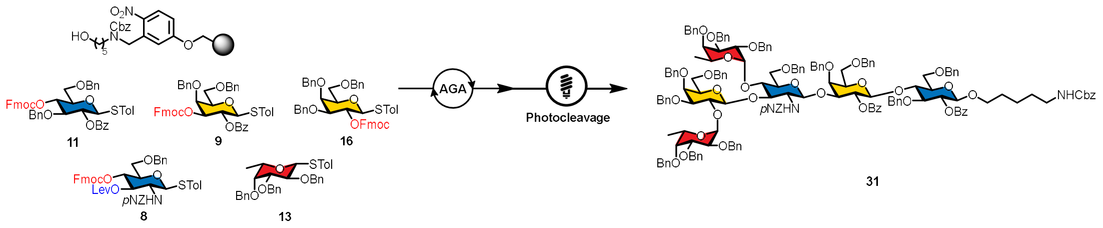

	Building blocks	Modules	Glycosylation condition
AGA		**Initiation** (40 mg resin)	
	**11**	**Acidic wash**<br>**Thioglycoside glycosylation**<br>**Capping**<br>**Fmoc deprotection**	**6.5 eq.** / **t (min)**<br>T_1 = -20 °C / 5<br>T_2 = 0 °C / 20
	**9**	**Acidic wash**<br>**Thioglycoside glycosylation**<br>**Capping**<br>**Fmoc deprotection**	**6.5 eq.** / **t (min)**<br>T_1 = -40 °C / 5<br>T_2 = -20 °C / 20
	**8**	**Acidic wash** ⎤<br>**Thioglycoside glycosylation** ⎥ **x2**<br>**Pyridine wash** ⎦<br>**Capping**<br>**Fmoc deprotection**	**6.5 eq.** / **t (min)**<br>T_1 = 0 °C / 30
	**16**	**Acidic wash**<br>**Thioglycoside glycosylation**<br>**Capping**<br>**Fmoc deprotection**	**6.5 eq.** / **t (min)**<br>T_1 = -20 °C / 10<br>T_2 = 0 °C / 20
	**13**	**Acidic wash** ⎤<br>**Thioglycoside glycosylation** ⎥ **x2**<br>**Pyridine wash** ⎦<br>**Capping**	**8.0 eq.** / **t (min)**<br>T_1 = -40 °C / 5<br>T_2 = -20 °C / 20
Post AGA		**Photocleavage**<br>**NP-HPLC Purification**	

Protected **31** (5.6 mg, 2.0 µmol, 15%) was obtained as a colorless syrup by purification using preparative NP-HPLC (**Method C**).

Analytical NP-HPLC of the crude **31** (**Method A**, ELSD trace, t_R = 27.8 min)

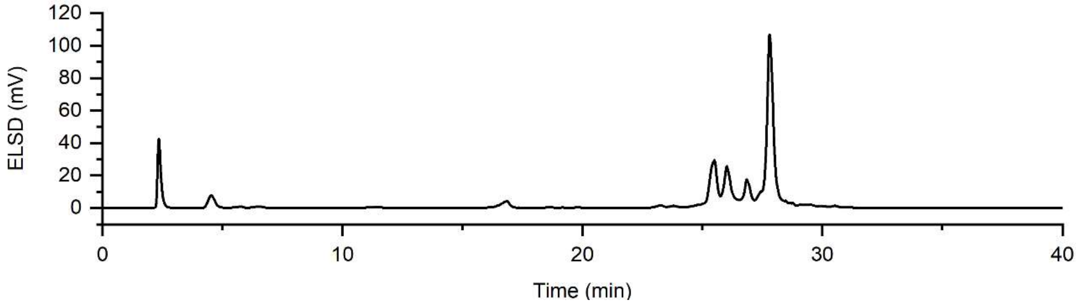

^{1}H NMR (700 MHz, $CDCl_3$) δ 8.03 (d, *J* = 8.5 Hz, 2H), 7.96 (d, *J* = 7.7 Hz, 2H), 7.85 (d, *J* = 7.5 Hz, 2H), 7.55 (t, *J* = 7.3 Hz, 1H), 7.51 (t, *J* = 7.3 Hz, 1H), 7.43 – 7.01 (m, 76H), 6.99 (d, *J* = 7.2 Hz, 2H), 6.94 (t, *J* = 7.5 Hz, 2H), 6.87 (d, *J* = 6.0 Hz, 1H), 5.59 (d, *J* = 3.8 Hz, 1H), 5.53 (dd, *J* = 9.9, 8.3 Hz, 1H), 5.20 – 5.12 (m, 1H), 5.11 – 5.02 (m, 4H), 4.94 (d, *J* = 3.4 Hz, 1H), 4.91 (d, *J* = 11.0 Hz, 2H), 4.85 – 4.70 (m, 6H), 4.70 – 4.57 (m, 6H), 4.58 – 4.23 (m, 21H), 4.18 (d, *J* = 11.8 Hz, 2H), 4.10 – 3.96 (m, 5H), 3.94 – 3.83 (m, 3H), 3.81 – 3.72 (m, 4H), 3.72 – 3.62 (m, 3H), 3.63 – 3.58 (m, 2H), 3.57 – 3.46 (m, 4H), 3.45 – 3.34 (m, 4H), 3.32 – 3.26 (m, 2H), 3.23 – 3.10 (m, 2H), 2.87 (s, 1H), 1.51 – 1.34 (m, 4H), 1.22 – 1.08 (m, 8H).

^{13}C NMR (176 MHz, $CDCl_3$) δ 165.1, 164.6, 156.3, 155.0, 147.4, 144.1, 139.3, 139.1, 139.1, 139.0, 138.9, 138.7, 138.6, 138.5, 138.3, 138.2, 138.2, 138.1, 137.8, 137.6, 136.7, 133.3, 133.0, 130.1, 129.9, 129.8, 129.7, 128.8, 128.7, 128.7, 128.6, 128.5, 128.5, 128.5, 128.4, 128.4, 128.2, 128.1, 128.1, 128.0, 128.0, 127.9, 127.8, 127.7, 127.6, 127.6, 127.3, 127.3, 127.2, 127.2, 127.0, 127.0, 127.0, 126.1, 123.7, 101.2, 100.4, 98.7, 97.9, 83.6, 80.6, 80.4, 79.3, 79.2, 78.3, 77.9, 77.7, 77.7, 77.2, 77.0, 76.9, 76.3, 76.2, 75.8, 75.5, 75.4, 75.2, 75.0, 74.9, 74.9, 74.8, 74.7, 74.7, 74.6, 74.5, 74.5, 73.7, 73.6, 73.5, 73.4, 73.3, 73.1, 73.0, 72.9, 72.8, 72.7, 71.9, 71.6, 71.2, 69.4, 68.2, 67.6, 67.4, 67.0, 66.6, 66.5, 64.8, 40.8, 29.7, 29.4, 28.9, 23.1, 16.3, 16.3.

HRMS (QToF): Calcd for $C_{169}H_{177}N_3O_{36}Na$ [M + Na]$^+$ 2847.2009; found 2847.2078.

^{1}H NMR (700 MHz, $CDCl_3$) of **31**:

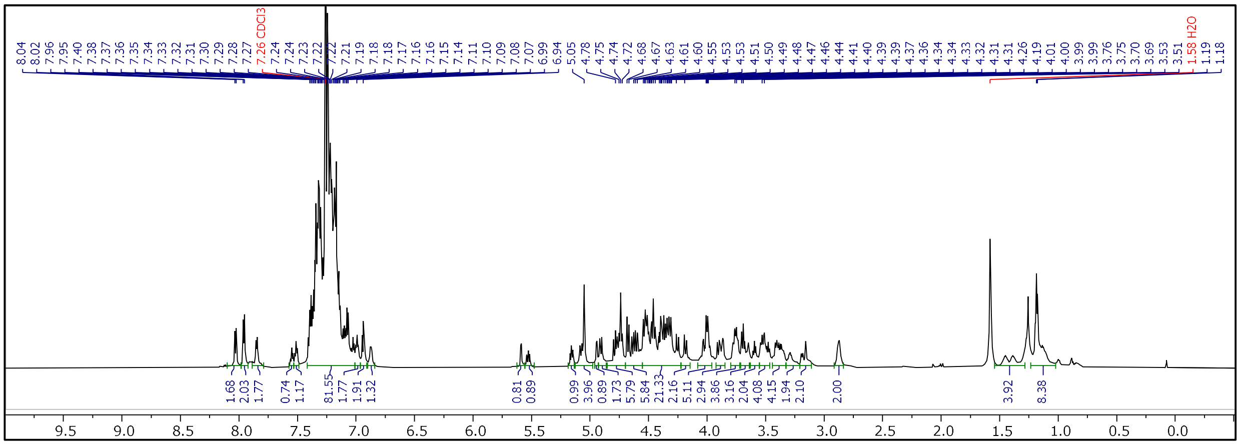

^{13}C NMR (176 MHz, $CDCl_3$) of **31**:

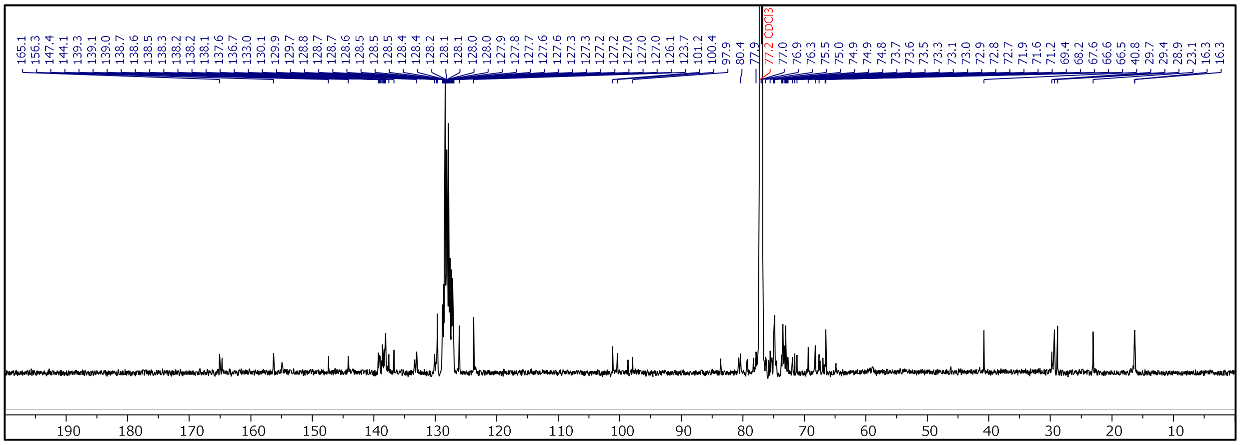

6. Optimization of *p*NZ group deprotection

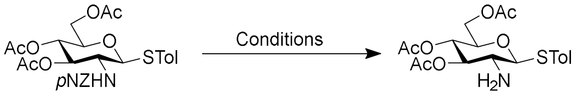

Condition A:

To a solution of compound **S2** (50 mg, 0.08 mmol, 1.0 equiv.) in DMF (3 mL) was added $SnCl_2$ (500 mg) and 1 M of HCl in dioxane (50 μL). The mixture was stirred at room temperature for overnight. The insoluble materials were filtered off and the filtrate was concentrated in *vacuo*.[3,4] Compound **S7** (23 mg, 0.056 mmol, 70%) was obtained as a white solid after purification by column chromatography (SiO_2, Hex/EtOAc = 2:1).

Condition B:

Compound **S2** (50 mg, 0.08 mmol, 1.0 equiv.), $B_2(OH)_4$ (22 mg, 0.24 mmol, 3.0 equiv.), and 4,4'-bipyridine (0.13 mg, 0.8 μmol, 0.01 equiv.) were all added to a sample vial. Then, DMF (3 mL) was added to a reaction mixture. Over ten minutes, the reaction mixture changed color from transparent to purple and subsequently to yellow. After 15 minutes, 1 M of HCl in dioxane (50 μL) was added, and the reaction mixture was stirred at room temperature for overnight. The consumption of the starting material was monitored by TLC. After the reaction, the reaction mixture was diluted with water and ethyl acetate. The organic layer was collected and washed with brine.[5] Compound **S7** (29 mg, 0.0696 mmol, 87%) was obtained as a white solid after purification by column chromatography (SiO_2, Hex/EtOAc = 2:1).

Condition C:

To a solution of compound **S2** (50mg, 0.08 mmol) in AcOH (5 mL) was added Zn-Cu (500 mg) and the mixture was stirred at 50°C for overnight. The insoluble materials were filtered off and the filtrate was concentrated *in vacuo*.[6] Compound **S7** (27 mg, 0.0648 mmol, 81%) was obtained as a white solid after purification by column chromatography (SiO_2, Hex/EtOAc = 2:1).

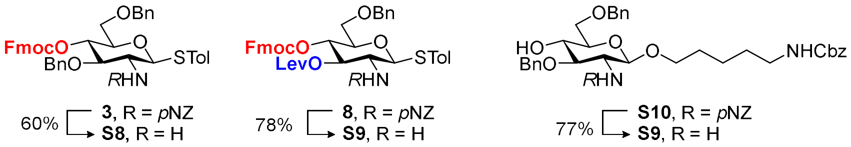

_p_-Methylphenyl 1,3,4,6-tetra-O-acetyl-2-deoxy-2-amino-1-thio-β-D-glucopyranoside (S7)

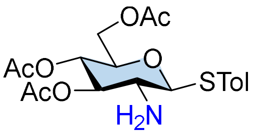

^{1}H NMR (600 MHz, $CDCl_3$) δ 7.44 (d, *J* = 8.1 Hz, 1H), 7.13 (d, *J* = 8.1 Hz, 1H), 4.97 (t, *J* = 9.5 Hz, 1H), 4.94 (t, *J* = 9.5 Hz, 1H), 4.40 (d, *J* = 9.9 Hz, 1H, **H_1**), 4.23 (dd, *J* = 12.2, 5.1 Hz, 1H), 4.17 (dd, *J* = 12.2, 2.4 Hz, 1H), 3.69 (ddd, *J* = 9.5, 5.1, 2.4 Hz, 1H), 2.82 (t, *J* = 9.5 Hz, 1H), 2.36 (s, 3H), 2.09 (s, 3H), 2.05 (s, 3H), 2.01 (s, 3H).

^{13}C NMR (151 MHz, $CDCl_3$) δ 170.8, 169.9, 138.9, 134.0, 129.9, 127.4, 89.7 (**C_1**), 77.2, 75.9, 68.8, 62.6, 54.6, 29.9, 21.3, 20.95, 20.93, 20.8.

HRMS (QToF): Calcd for $C_{19}H_{25}NO_7SNa$ $[M + Na]^+$ 434.1244; found 434.1234.

^{1}H NMR (600 MHz, $CDCl_3$) of **S7**:

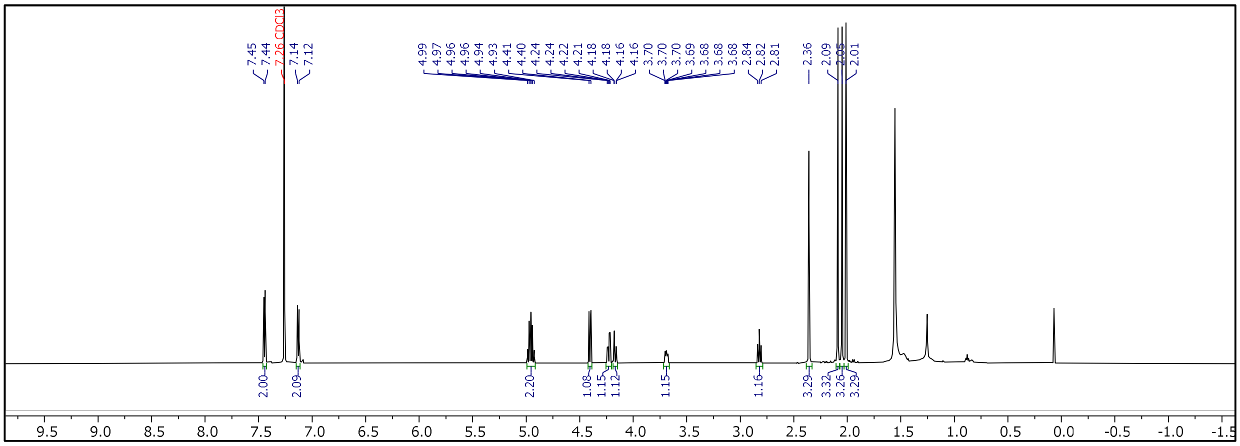

^{13}C NMR (151 MHz, $CDCl_3$) of **S7**:

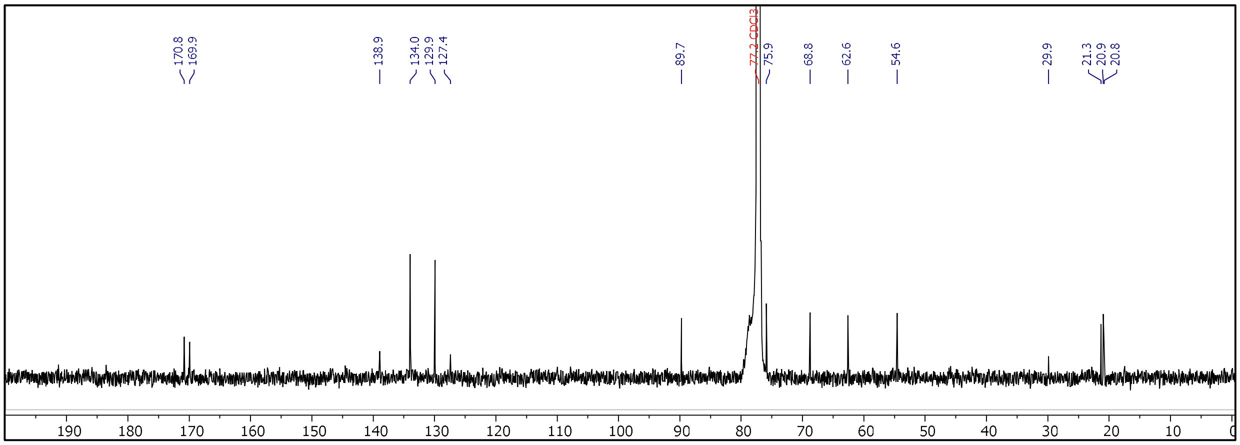

***p*-Methylphenyl 3,6-di-*O*-benzyl-2-deoxy-2-amino-4-*O*-fluorenylmethoxycarbonyl-1-thio-β-D-glucopyranoside (S8)**

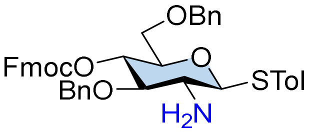

Deprotection procedure was shown as **Condition B** to afford title compound **S8** (36 mg, 0.052 mmol, 65%)

^{1}H NMR (400 MHz, $CDCl_3$) δ 7.74 (dd, *J* = 7.6, 2.4 Hz, 2H), 7.56 (d, *J* = 7.5 Hz, 1H), 7.51 (d, *J* = 7.5 Hz, 1H), 7.47 – 7.40 (m, 2H), 7.40 – 7.29 (m, 6H), 7.29 – 7.20 (m, 8H), 7.04 (d, *J* = 7.9 Hz, 2H), 4.85 (t, *J* = 9.4 Hz, 1H), 4.73 (d, *J* = 11.3 Hz, 1H), 4.63 (d, *J* = 11.3 Hz, 1H), 4.53 (s, 2H), 4.43 (d, *J* = 9.8 Hz, 1H, $\mathbf{H_1}$), 4.31 (dd, *J* = 7.2, 1.8 Hz, 2H), 4.11 (t, *J* = 7.1 Hz, 1H), 3.73 – 3.62 (m, 3H), 3.54 (t, *J* = 9.4 Hz, 1H), 2.86 (t, *J* = 9.6 Hz, 1H), 2.31 (s, 3H).

^{13}C NMR (101 MHz, $CDCl_3$) δ 154.6, 143.4, 143.2, 141.4, 141.4, 138.5, 138.2, 137.9, 133.6, 129.9, 128.6, 128.5, 128.1, 128.0, 128.0, 128.0, 127.9, 127.7, 127.7, 127.3, 125.2, 125.1, 120.21, 120.19, 89.0 ($\mathbf{C_1}$), 84.3, 77.2, 76.2, 75.2, 73.7, 70.2, 69.9, 55.2, 46.8, 29.9, 21.3.

HRMS (QToF): Calcd for $C_{42}H_{41}NO_6SNa$ $[M + Na]^+$ 710.2547; found 710.2575.

^{1}H NMR (400 MHz, $CDCl_3$) of **S8**:

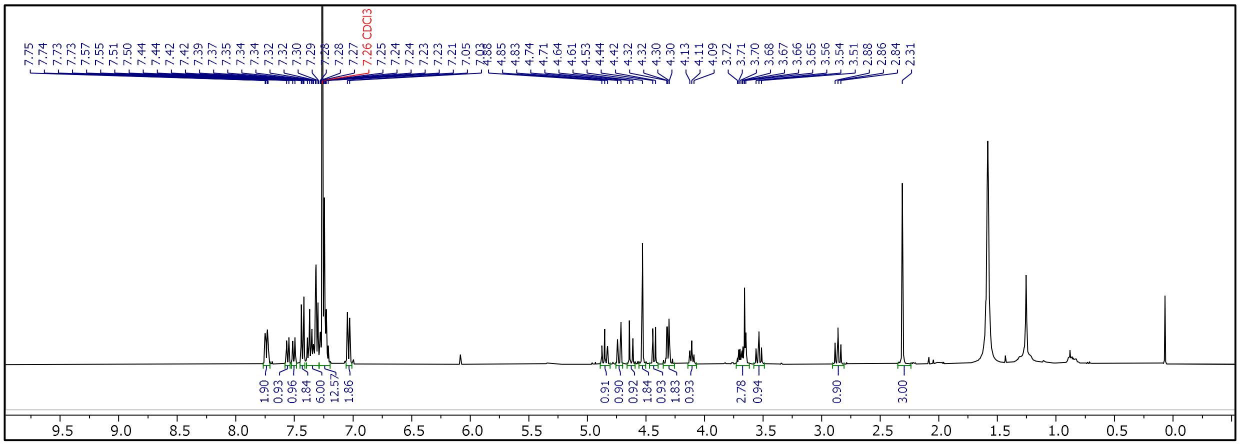

^{13}C NMR (101 MHz, $CDCl_3$) of **S8**:

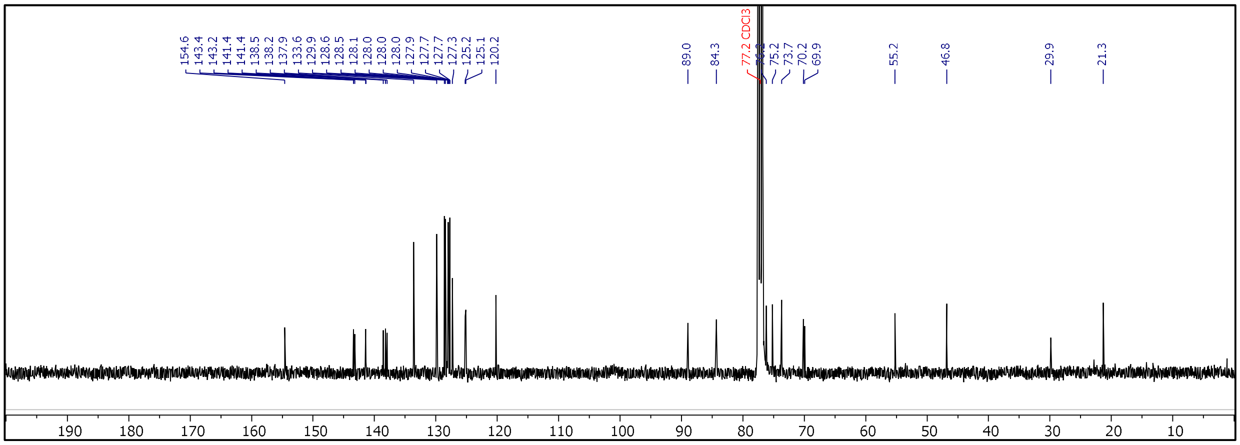

***p*-Methylphenyl 6-*O*-benzyl-2-deoxy-2-amino-4-*O*-fluorenylmethoxycarbonyl-3-*O*-levulinoyl-1-thio-β-D-glucopyranoside (S9)**

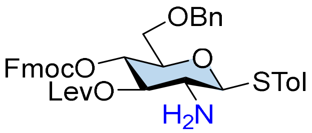

Deprotection procedure was shown as **Condition B** to afford title compound **S9** (44 mg, 0.064 mmol, 80%)

^{1}H NMR (400 MHz, $CDCl_3$) 7.76 (d, *J* = 7.5 Hz, 2H), 7.57 (dd, *J* = 10.7, 7.6 Hz, 2H), 7.46 (d, *J* = 8.1 Hz, 2H), 7.40 (t, *J* = 7.5 Hz, 3H), 7.34 – 7.20 (m, 6H), 7.07 (d, *J* = 7.9 Hz, 2H), 5.11 (t, *J* = 9.6

Hz, 1H), 4.86 (t, J = 9.7 Hz, 1H), 4.56 (d, J = 12.0 Hz, 1H), 4.51 (d, J = 12.0 Hz, 1H), 4.46 (d, J = 9.9 Hz, 1H, **H_1**), 4.39 (dd, J = 10.1, 7.3 Hz, 1H), 4.26 (dd, J = 10.1, 7.6 Hz, 1H), 4.19 (t, J = 7.4 Hz, 1H), 3.81 – 3.72 (m, 1H), 3.72 – 3.63 (m, 2H), 2.89 (t, J = 9.8 Hz, 1H), 2.76 – 2.58 (m, 2H), 2.57 – 2.48 (m, 1H), 2.48 – 2.37 (m, 1H), 2.33 (s, 3H), 2.05 (s, 3H).

^{13}C NMR (100 MHz, $CDCl_3$) δ 206.6, 172.5, 154.4, 143.5, 143.4, 141.4, 141.4, 138.6, 138.1, 133.7, 129.9, 128.5, 128.0, 128.0, 127.8, 127.8, 127.7, 127.3, 125.4, 125.4, 120.2, 120.2, 89.6 (**C_1**), 77.4, 77.3, 77.2, 73.7, 73.4, 70.5, 69.4, 55.0, 46.7, 38.1, 29.7, 28.1, 21.3.

HRMS (QToF): Calcd for $C_{40}H_{42}NO_8S$ $[M + H]^+$ 696.2626; found 696.2648.

^{1}H NMR (400 MHz, $CDCl_3$) of **S9**:

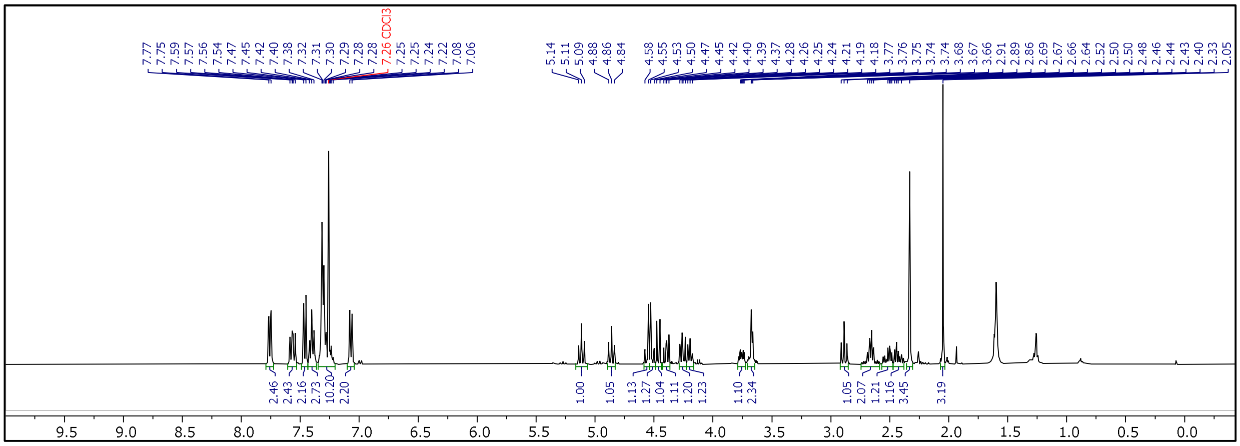

^{13}C NMR (100 MHz, $CDCl_3$) of **S9**:

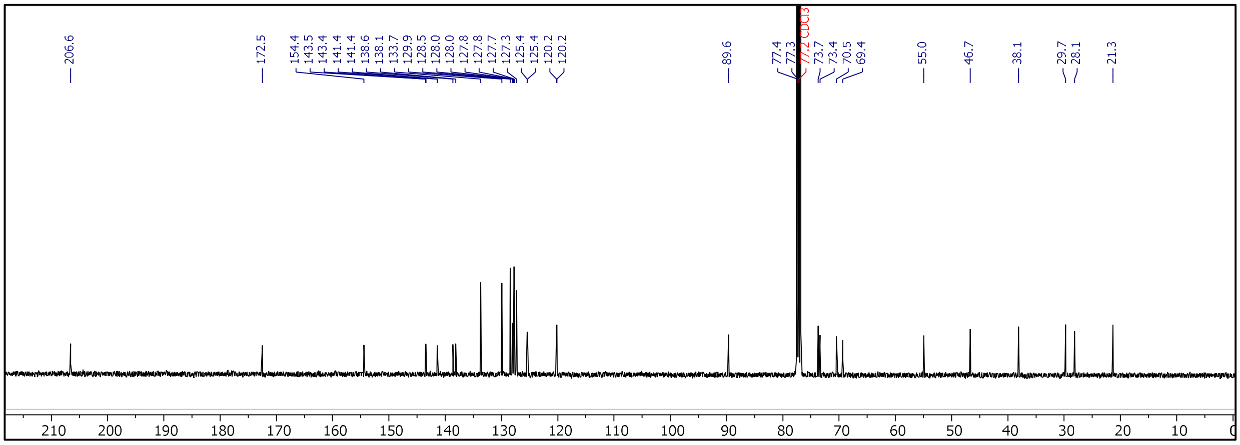

***N*-benzyloxycarbonyl-5-amino-pentanyl 3,6-di-*O*-benzyl-2-deoxy-2-(4-nitrobenzyloxycarbonyl amino)-β-D-glucopyranoside (S10)**

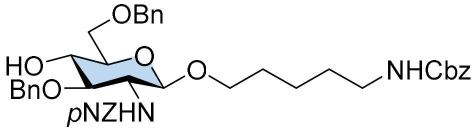

To the mixture of compound **3** (100 mg, 0.12 mmol, 1.0 equiv.) and 4Å molecular sieves (100 mg) in anhydrous CH_2Cl_2 (5 mL) was added 5-*N*-benzyloxycarbonyl aminopentanol[7] (43 mg, 0.18 mmol, 1.5 equiv.) and the solution was kept stirring at 0 °C for 30 minutes. NIS (41 mg, 0.18 mmol, 1.5 equiv.) and TfOH (3.2 μL, 0.036 mmol, 0.3 equiv.) were added to the reaction mixture at 0 °C and stirred at same temperature for 1-2 h judged by TLC (EtOAc/Heaxane 1:4). After completion of the reaction, the solution was quenched by TEA (1 mL) for 3 h. Then, the solution was filtered through celite and washed with CH_2Cl_2. The filtrate was evaporated *in vacuo* to furnish the crude product, which was purified by flash column chromatography to give the product **S10** (77 mg, 85%).

^{1}H NMR (600 MHz, DMSO) δ 8.12 (d, *J* = 8.5 Hz, 2H), 7.56 (d, *J* = 9.4 Hz, 1H), 7.53 (d, *J* = 8.4 Hz, 2H), 7.40 – 7.31 (m, 8H), 7.32 – 7.19 (m, 8H), 5.42 (d, *J* = 6.0 Hz, 1H), 5.20 (d, *J* = 14.1 Hz, 1H), 5.13 (d, *J* = 14.1 Hz, 1H), 4.99 (s, 2H), 4.79 (d, *J* = 11.5 Hz, 1H), 4.57 (d, *J* = 11.5 Hz, 1H), 4.54 (s, 2H), 4.32 (d, *J* = 8.3 Hz, 1H, **H_1**), 3.76 (d, *J* = 10.5 Hz, 1H), 3.73 – 3.67 (m, 1H), 3.57 (dd, *J* = 10.8, 5.4 Hz, 1H), 3.50 – 3.38 (m, 2H), 3.38 – 3.28 (m, 3H), 2.95 (q, *J* = 6.5 Hz, 2H), 1.53 – 1.45 (m, 2H), 1.43 – 1.33 (m, 2H), 1.31 – 1.21 (m, 2H).

^{13}C NMR (151 MHz, DMSO) δ 156.1, 155.7, 146.8, 145.4, 139.1, 138.6, 137.3, 128.3, 128.2, 127.9, 127.9, 127.7, 127.3, 127.3, 127.2, 127.1, 123.4, 100.9 (**C_1**), 82.7, 75.6, 73.6, 72.3, 70.2, 69.4, 68.5, 65.1, 63.9, 56.3, 40.2, 39.5, 29.1, 28.7, 22.6.

HRMS (QToF): Calcd for $C_{41}H_{47}N_3O_{11}Na$ $[M + Na]^+$ 780.3103; found 780.3132.

^{1}H NMR (600 MHz, DMSO) of **S10**:

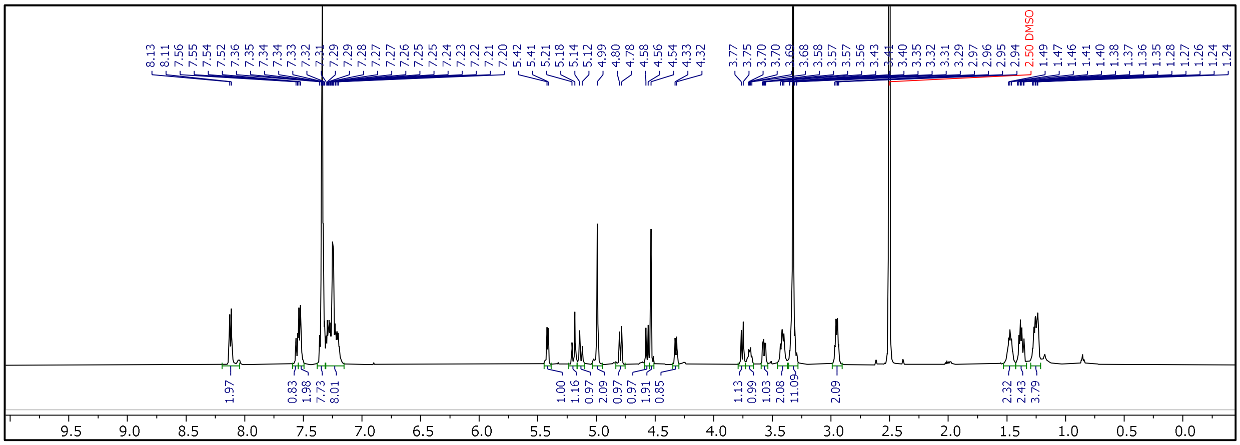

^{13}C NMR (151 MHz, DMSO) of **S10**:

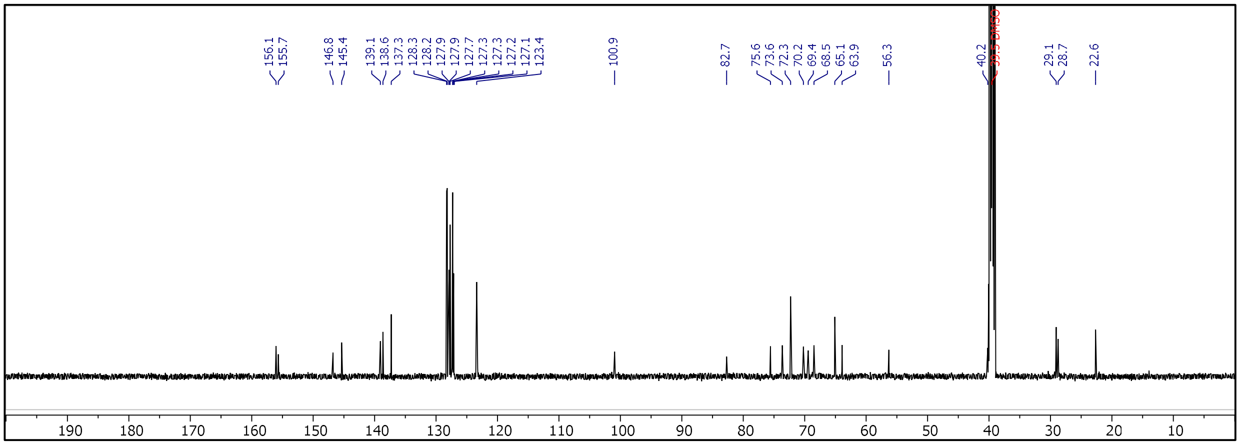

***N*-benzyloxycarbonyl-5-amino-pentanyl 3,6-di-*O*-benzyl-2-deoxy-2-amino-β-D-glucopyranoside (S11)**

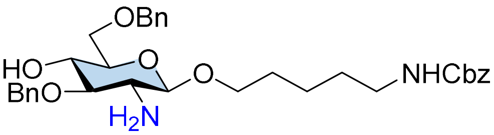

Deprotection procedure was shown as **Condition B** to afford title compound **S11** (36 mg, 0.062 mmol, 77%)

^{1}H NMR (600 MHz, $CDCl_3$) 7.46 – 7.27 (m, 15H), 5.08 (s, 2H), 4.95 (d, *J* = 11.5 Hz, 1H), 4.82 (br., 1H), 4.75 (d, *J* = 11.5 Hz, 1H), 4.60 (d, *J* = 11.9 Hz, 1H), 4.55 (d, *J* = 11.9 Hz, 1H), 4.19 (d, *J* = 7.9 Hz, 1H, **H_1**), 3.91 – 3.82 (m, 1H), 3.77 (dd, *J* = 9.9, 5.0 Hz, 1H), 3.75 – 3.66 (m, 2H), 3.52 –

3.45 (m, 2H), 3.33 (t, J = 9.3 Hz, 1H), 3.24 – 3.14 (m, 2H), 2.81 (dd, J = 9.7, 7.9 Hz, 1H), 1.63 – 1.58 (m, 2H), 1.55 – 1.47 (m, 2H), 1.44 – 1.34 (m, 2H).

^{13}C NMR (151 MHz, $CDCl_3$) δ 138.8, 137.7, 136.8, 128.7, 128.7, 128.3, 128.1, 128.1, 128.1, 128.0, 104.0 (**C_1**), 84.9, 77.2, 75.0, 73.9, 73.6, 71.4, 69.8, 66.7, 56.3, 41.1, 29.7, 29.3, 23.3.

HRMS (QToF): Calcd for $C_{33}H_{43}N_2O_7$ $[M + H]^+$ 579.3065; found 579.3075.

^{1}H NMR (600 MHz, DMSO) of **S11**:

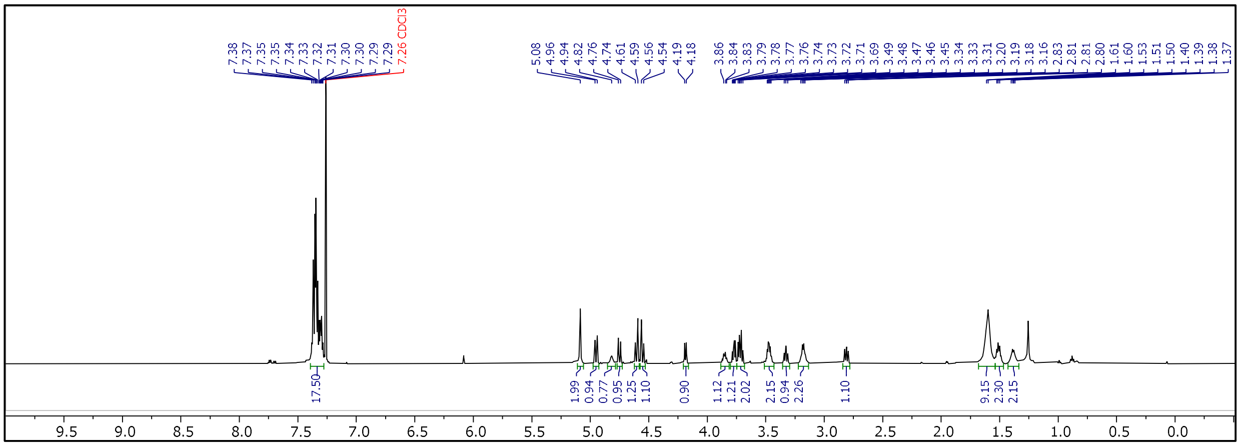

^{13}C NMR (151 MHz, DMSO) of **S11**:

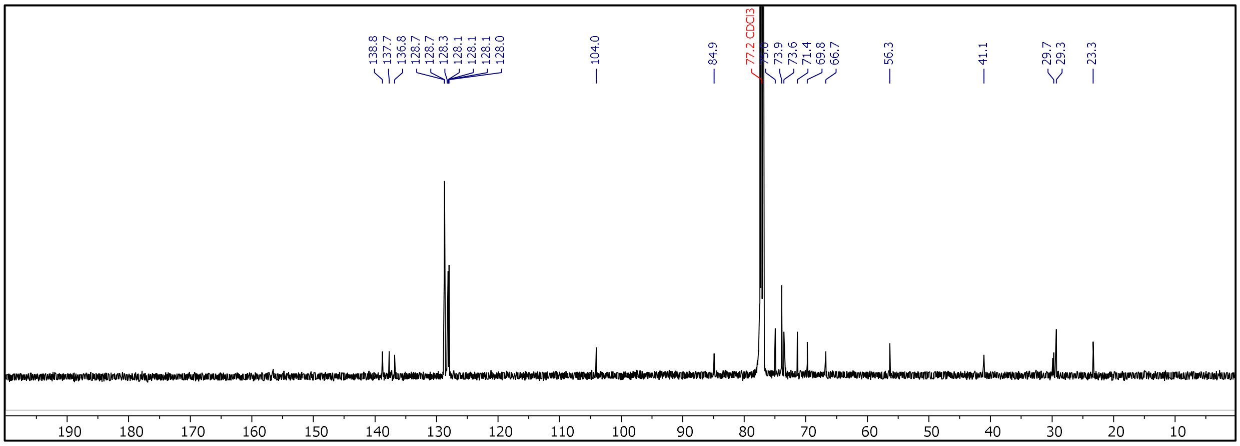

6.1 Oligo-LacNAc: Tetramer **32**

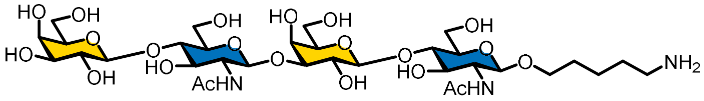

Deprotection procedure was shown as **General Deprotection Procedure** (Section: 4.4 Post-synthesizer Manipulation). Compound **32** (2.3 mg, 2.7 μmol, 55%) was obtained as a white solid by purification using preparative RP-HPLC (**Method H**) and lyophilizer.

(A) Analytical RP-HPLC of the crude **32** (**Method E,** ELSD trace, t_R = 35.6 min)

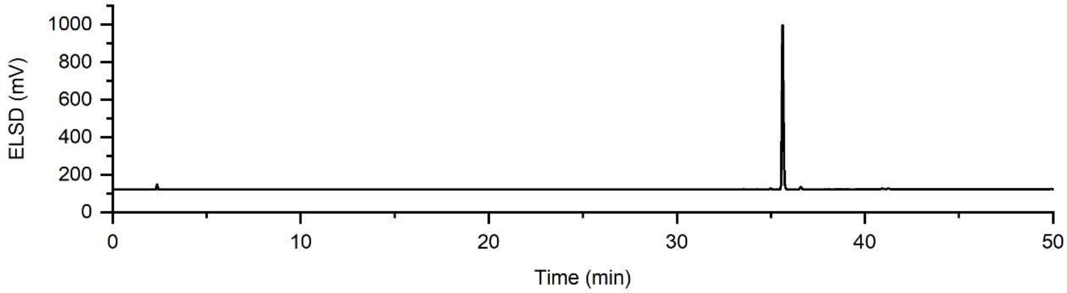

(B) Analytical RP-HPLC of the pure **32** (**Method E,** ELSD trace, t_R = 35.7 min)

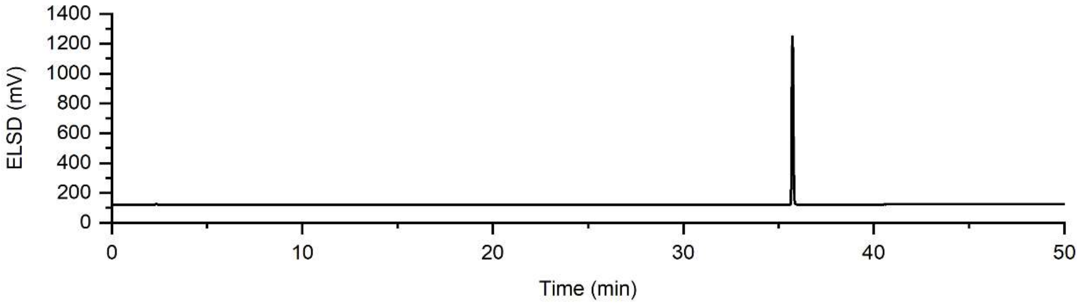

^{1}H NMR (600 MHz, D_2O) δ 4.71 (d, *J* = 8.4 Hz, 1H, **$H_1β$**), 4.53 (d, *J* = 7.9 Hz, 1H, **$H_1β$**), 4.49 (d, *J* = 7.8 Hz, 1H, **$H_1β$**), 4.47 (d, *J* = 7.9 Hz, 1H, **$H_1β$**), 4.17 (d, *J* = 3.3 Hz, 1H), 3.99 (dd, *J* = 12.2, 2.0 Hz, 1H), 3.96 (dd, *J* = 12.2, 2.0 Hz, 1H), 3.95 – 3.89 (m, 2H), 3.89 – 3.80 (m, 3H), 3.79 – 3.65 (m, 13H), 3.66 – 3.58 (m, 4H), 3.55 (dd, *J* = 9.9, 7.9 Hz, 1H), 3.00 (dd, *J* = 8.4, 6.9 Hz, 2H), 2.04 (s, 3H), 2.04 (s, 3H), 1.68 (p, *J* = 7.7 Hz, 2H), 1.61 (p, *J* = 6.5 Hz, 2H), 1.45 – 1.38 (m, 2H).

^{13}C NMR (151 MHz, D_2O) δ 174.9, 174.4, 102.9 (**C_1**), 102.9 (**C_1**), 102.7 (**C_1**), 101.1 (**C_1**), 82.1, 78.5, 78.2, 75.3, 74.9, 74.7, 74.5, 72.5, 72.4, 72.2, 70.9, 70.1, 69.9, 68.5, 68.3, 61.0, 60.9, 60.0, 59.8, 55.2, 55.0, 39.3, 28.1, 26.4, 22.2, 22.1, 22.1.

HRMS (QToF): Calcd for $C_{33}H_{60}N_3O_{21}$ a $[M + H]^+$ 834.3719; found 834.3757.

^{1}H NMR (600 MHz, D_2O) of **32**:

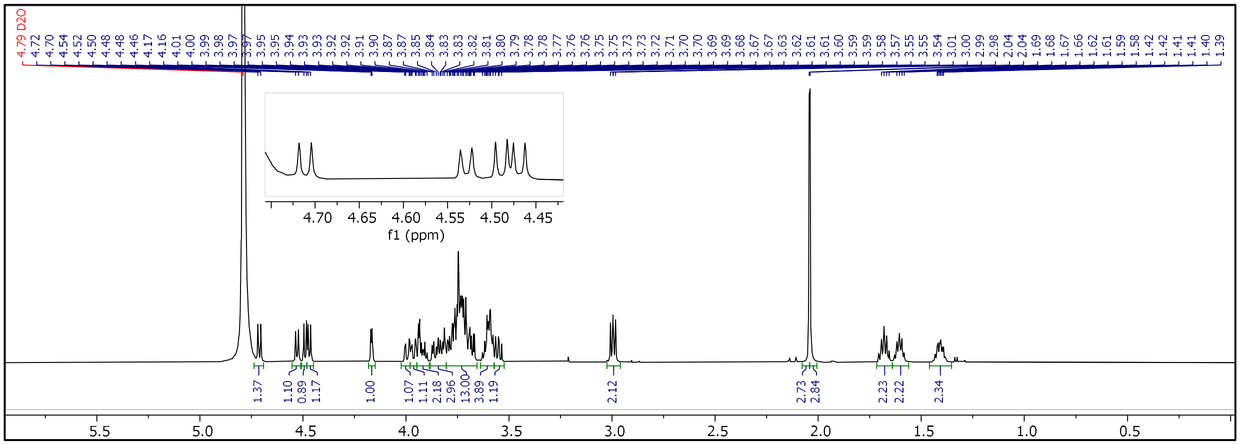

^{13}C NMR (151 MHz, D_2O) of **32**:

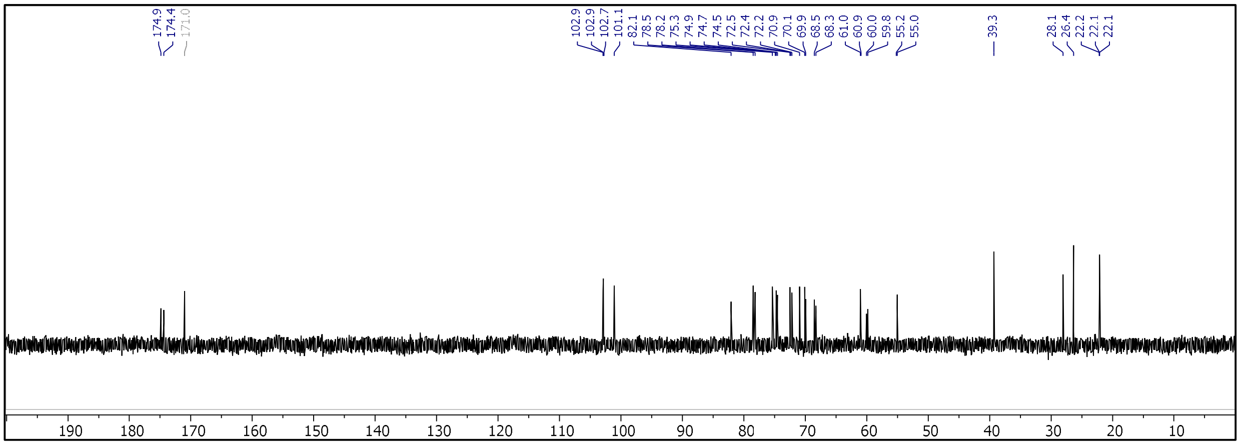

^1H-^{13}C HSQC NMR of **32**:

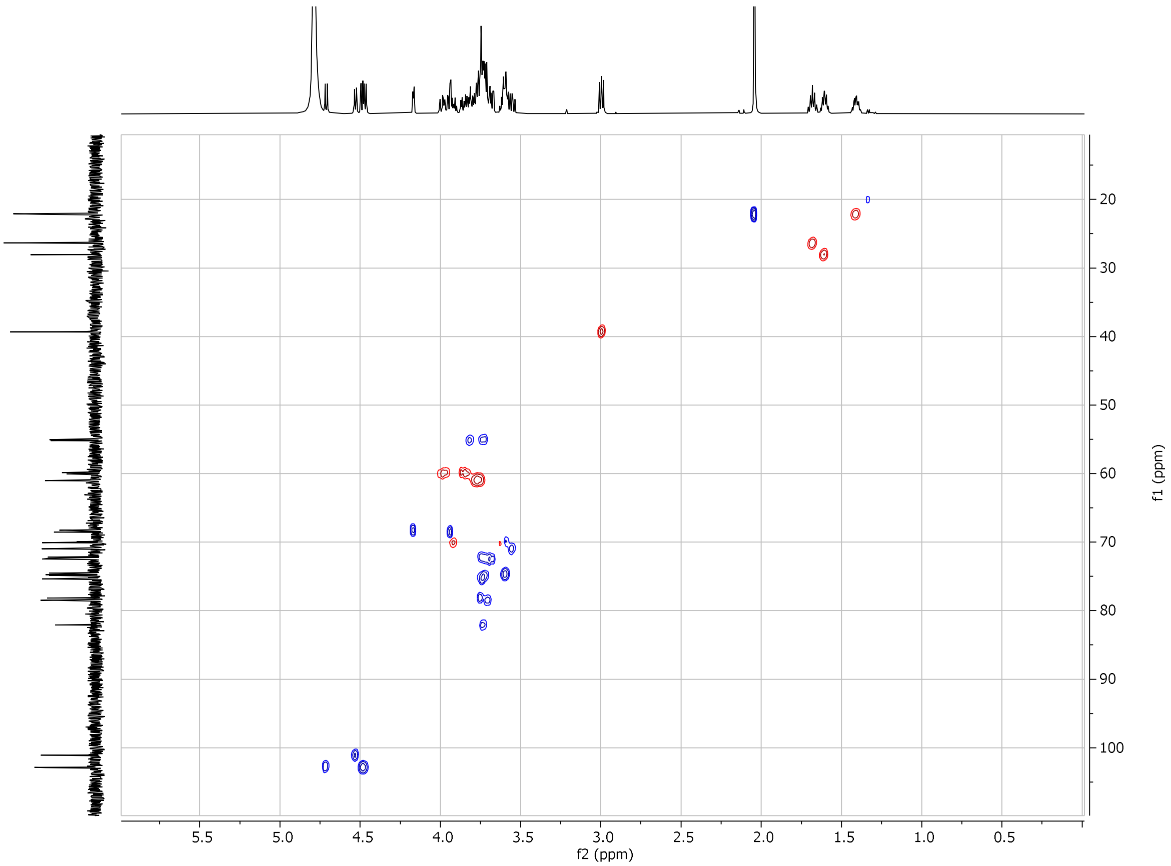

6.2 Oligo-LacNAc: Hexamer **33**

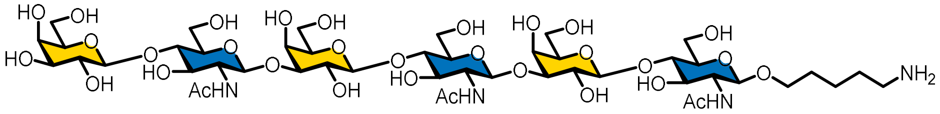

Deprotection procedure was shown as **General Deprotection Procedure** (Section: 4.4 Post-synthesizer Manipulation). Compound **33** (2.0 mg, 1.7 μmol, 50%) was obtained as a white solid by purification using preparative RP-HPLC (**Method H**) and lyophilizer.

(A) Analytical RP-HPLC of the crude **33** (**Method E,** ELSD trace, t_R = 28.2 min)

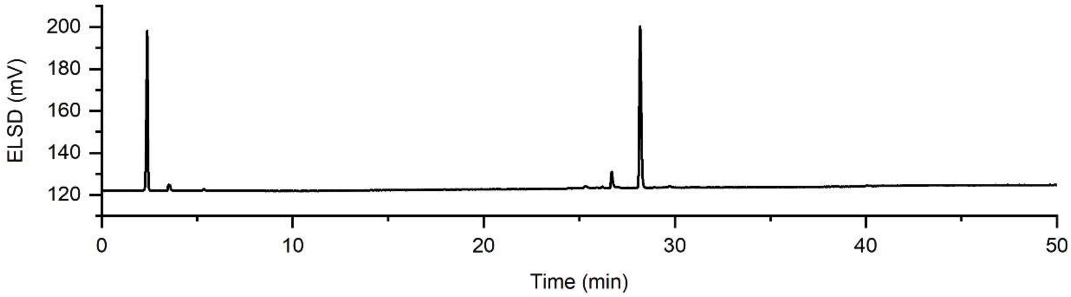

(B) Analytical RP-HPLC of the pure **33** (**Method E,** ELSD trace, t_R = 28.6 min)

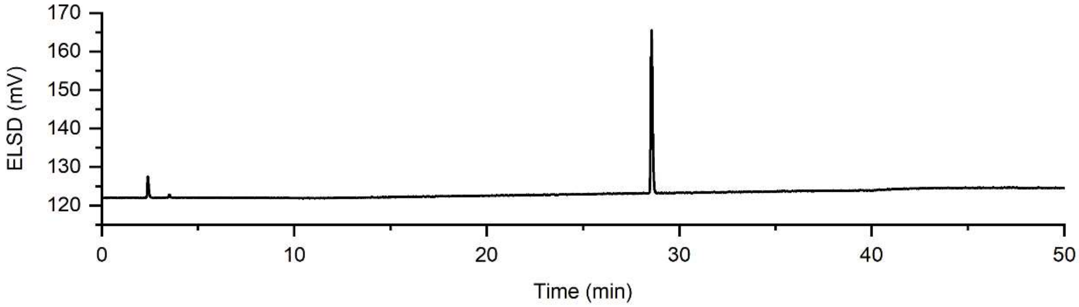

^{1}H NMR (700 MHz, D_2O) δ 4.67 (d, *J* = 8.4 Hz, 1H, **$H_1β$**), 4.67 (d, *J* = 8.4 Hz, 1H, **$H_1β$**), 4.49 (d, *J* = 7.9 Hz, 1H, **$H_1β$**), 4.45 (d, *J* = 7.7 Hz, 1H, **$H_1β$**), 4.44 (d, *J* = 7.6 Hz, 1H, **$H_1β$**), 4.43 (d, *J* = 7.6 Hz, 1H, **$H_1β$**), 4.13 (br., 2H), 4.01 – 3.86 (m, 5H), 3.85 – 3.63 (m, 24H), 3.63 – 3.54 (m, 6H), 3.51 (dd, *J* = 9.8, 8.0 Hz, 1H), 2.96 (d, *J* = 7.6 Hz, 2H), 2.01 (s, 9H), 1.65 (p, *J* = 7.7 Hz, 2H), 1.57 (p, *J* = 6.6 Hz, 2H), 1.42 – 1.34 (m, 2H).

^{13}C NMR (151 MHz, D_2O) δ δ 174.9, 174.4, 102.92 (2 × **C_1**), 102.89 (2 × **C_1**), 102.7 (**C_1**), 101.1 (**C_1**), 82.1, 78.5, 78.2, 75.4, 74.9, 74.8, 74.6, 72.5, 72.4, 72.2, 71.0, 70.1, 70.0, 68.6, 68.3, 61.0, 61.0, 60.1, 59.9, 55.21, 55.17, 55.1, 39.3, 28.1, 26.4, 22.19, 22.16, 22.1.

HRMS (QToF): Calcd for $C_{47}H_{83}N_4O_{31}$ $[M + H]^+$ 1199.5036; found 1199.5092.

^{1}H NMR (700 MHz, D_2O) of **33**:

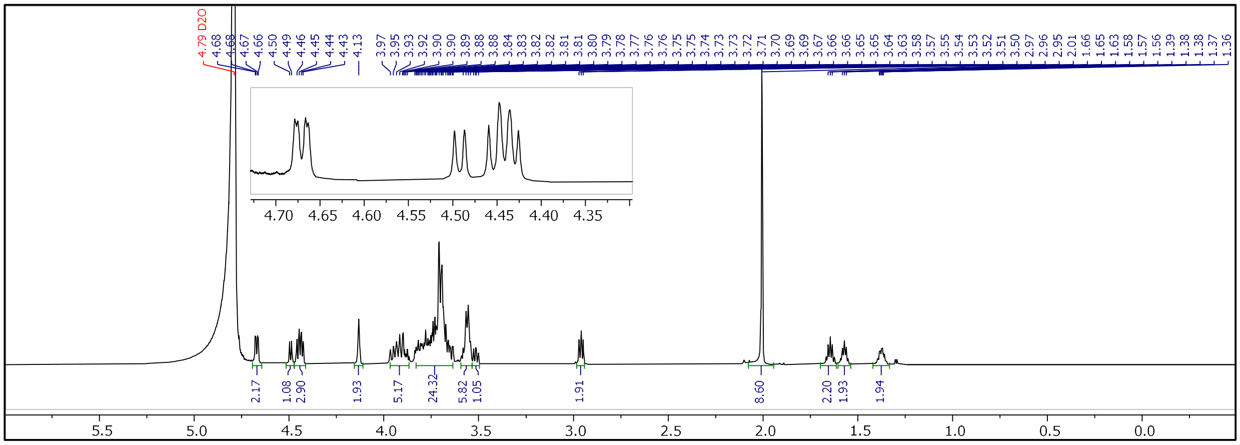

^{13}C NMR (151 MHz, D_2O) of **33**:

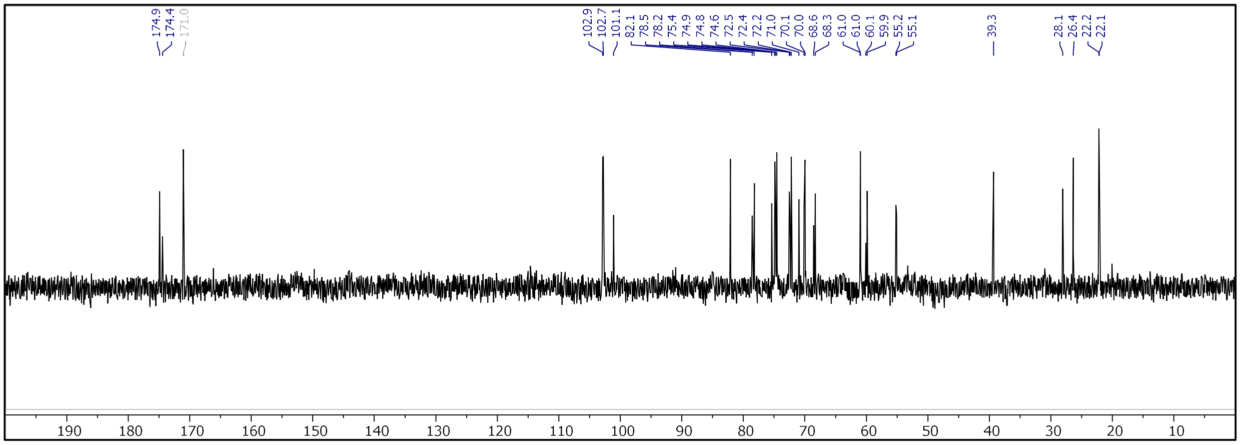

^{1}H-^{13}C HSQC NMR of **33**:

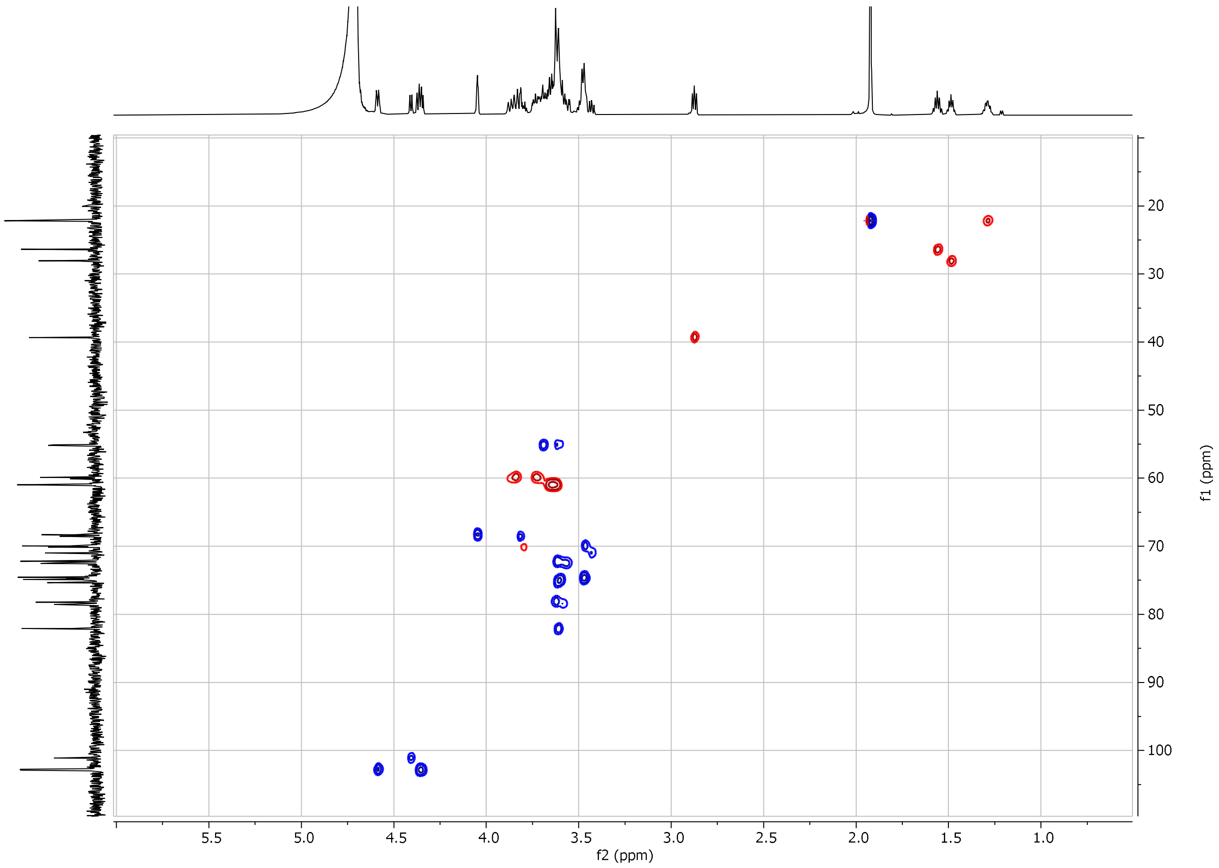

6.3 Oligo-LacNAc: Octamer **34**

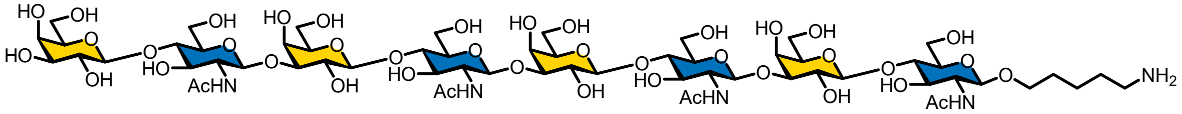

Deprotection procedure was shown as **General Deprotection Procedure** (Section4.4 Post-synthesizer Manipulation). Compound **34** (1.7 mg, 1.1 µmol, 43%) was obtained as a white solid by purification using preparative RP-HPLC (**Method H**) and lyophilizer.

(A) Analytical RP-HPLC of the crude **34** (**Method E**, ELSD trace, t_R = 33.0 min)

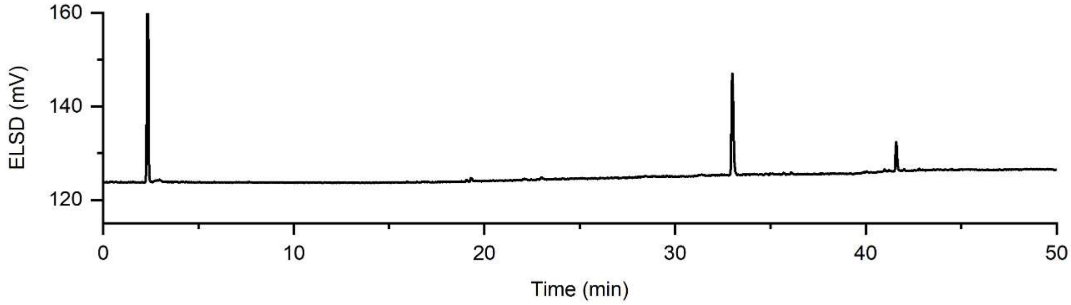

(B) Analytical RP-HPLC of the pure **34** (**Method E**, ELSD trace, t_R = 33.3 min)

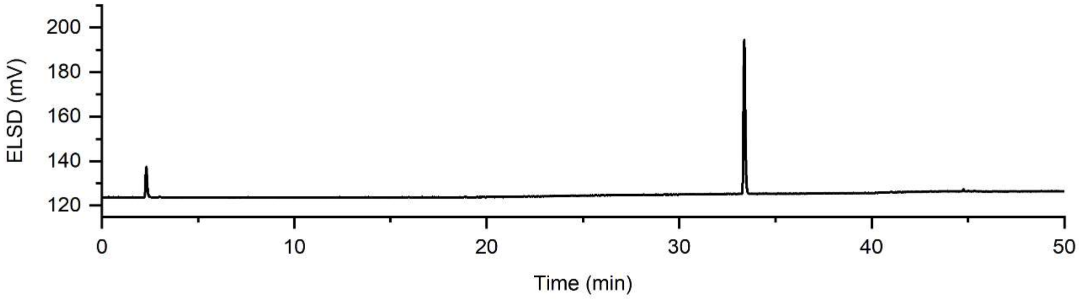

^{1}H NMR (600 MHz, D_2O) δ 4.73 – 4.69 (m, 3H, **H_1β, H_1β, H_1β**), 4.53 (d, *J* = 7.9 Hz, 1H, **H_1β**), 4.51 – 4.45 (m, 4H, **H_1β, H_1β, H_1β, H_1β**), 4.16 (d, *J* = 3.2 Hz, 3H), 4.02 – 3.89 (m, 6H), 3.88 – 3.66 (m, 32H), 3.63 – 3.52 (m, 9H), 2.99 (t, *J* = 7.5 Hz, 2H), 2.04 (s, 12H), 1.68 (p, *J* = 7.7 Hz, 2H), 1.64 – 1.57 (m, 2H), 1.45 – 1.37 (m, 2H).

^{13}C NMR (151 MHz, D_2O) δ 174.9, 171.0, 102.9 (**C_1**), 102.7 (**C_1**), 101.1 (**C_1**), 82.1, 78.1, 74.9, 74.7, 74.5, 72.5, 72.4, 72.2, 69.9, 68.5, 68.3, 61.0, 60.9, 59.8, 55.1, 55.0, 39.3, 28.0, 26.4, 22.1, 20.0.

HRMS (QToF): Calcd for $C_{61}H_{107}N_5O_{41}$ a $[M + 2H]^{2+}$ 782.8215; found 782.8223.

^{1}H NMR (600 MHz, D_2O) of **34**:

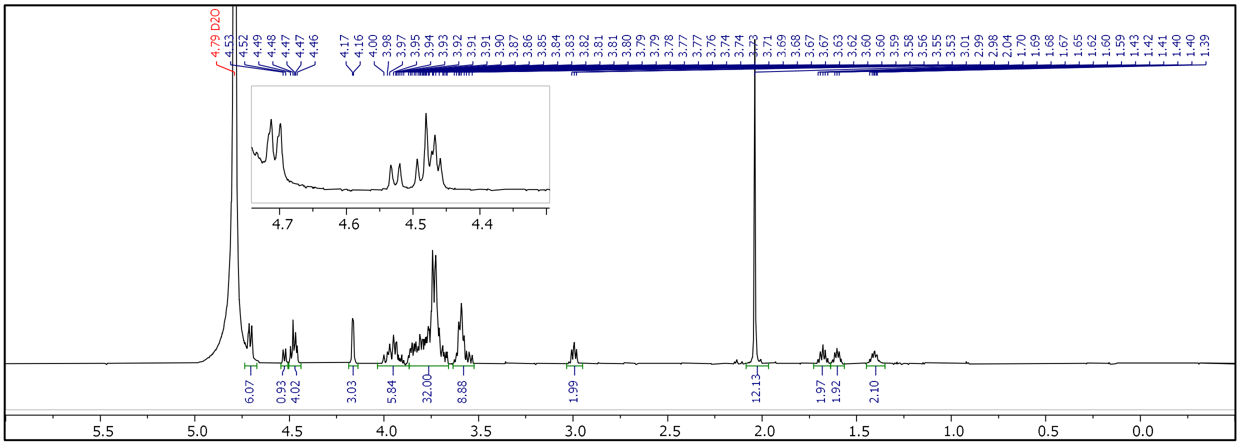

^{13}C NMR (151 MHz, D_2O) of **34**:

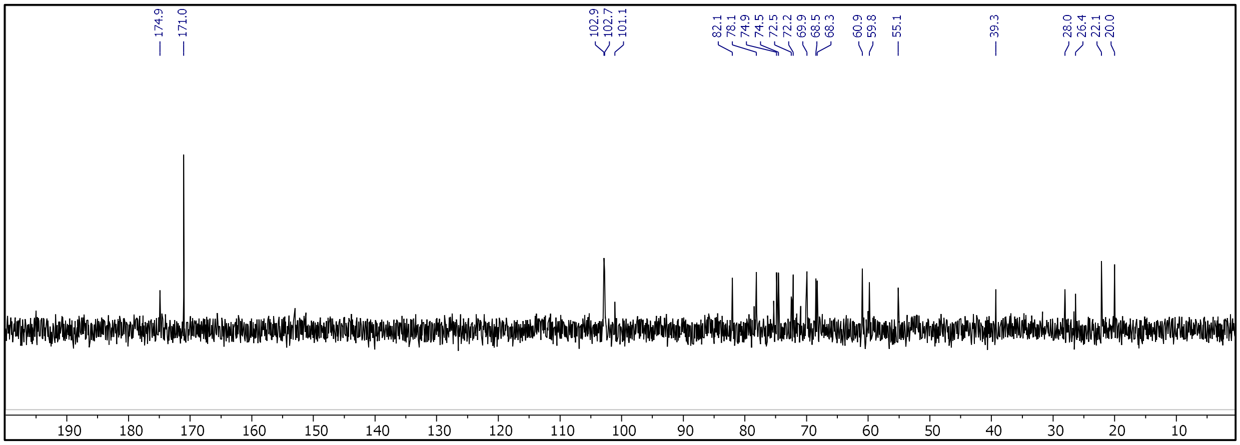

^{1}H-^{13}C HSQC NMR of **34**:

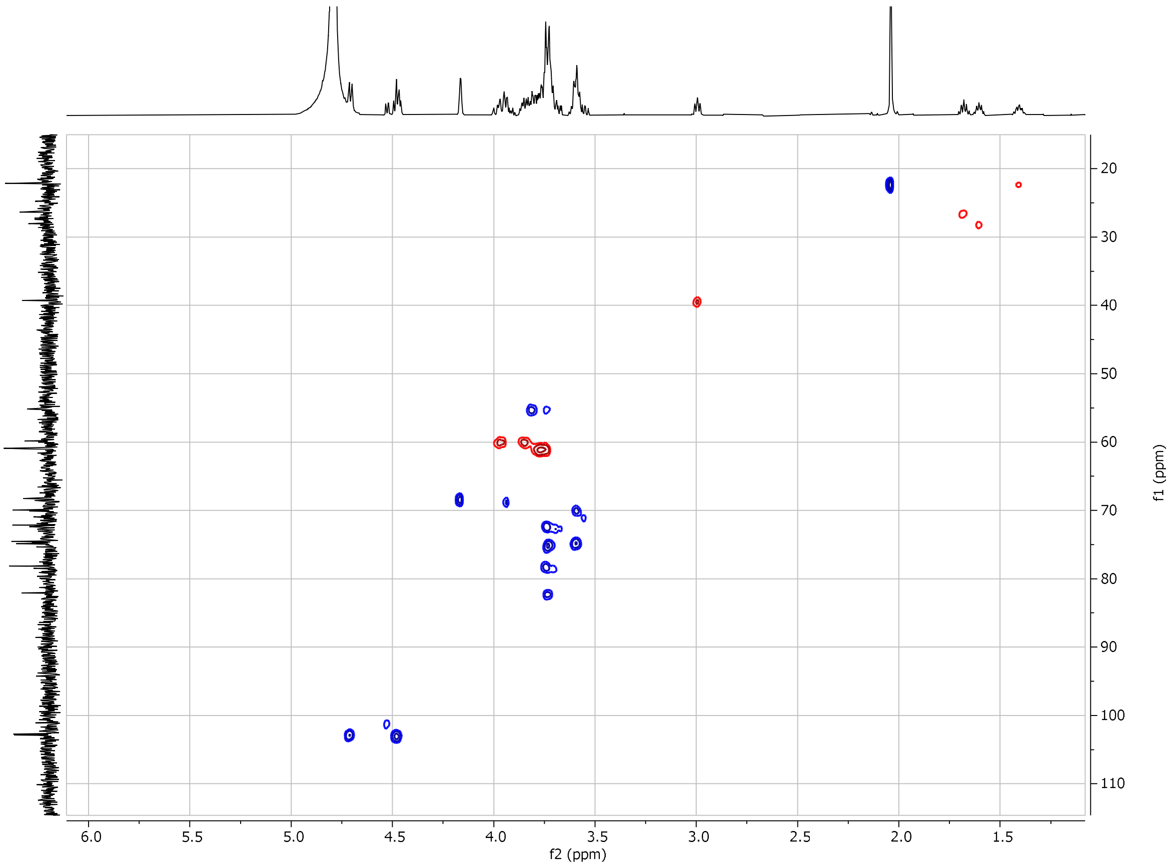

6.4 Lacto-N-neotetraose **LNnT 35**

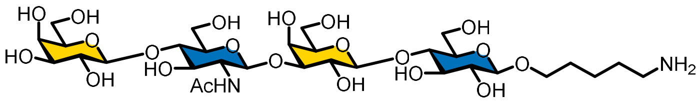

Deprotection procedure was shown as **General Deprotection Procedure** (Section: 4.4 Post-synthesizer Manipulation). Compound **LNnT (35)** (2.1 mg, 2.6 µmol, 55%) was obtained as a white solid by purification using preparative RP-HPLC (**Method H**) and lyophilizer.

(A) Analytical RP-HPLC of the crude **LNnT (35)** (**Method E**, ELSD trace, t_R = 26.5 min)

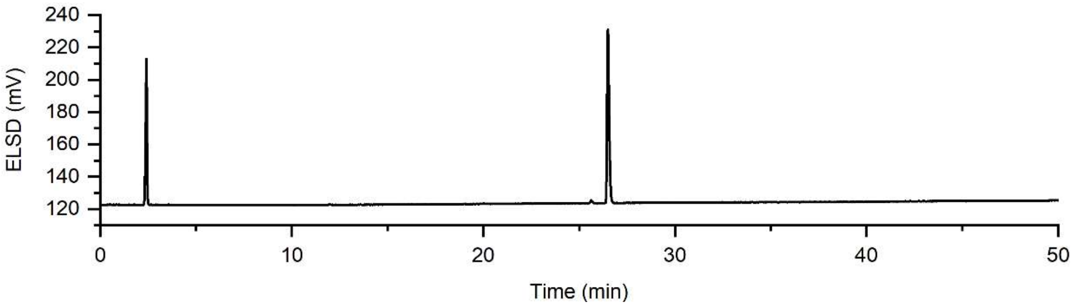

(B) Analytical RP-HPLC of the pure **LNnT (35)** (**Method E**, ELSD trace, t_R = 26.4 min)

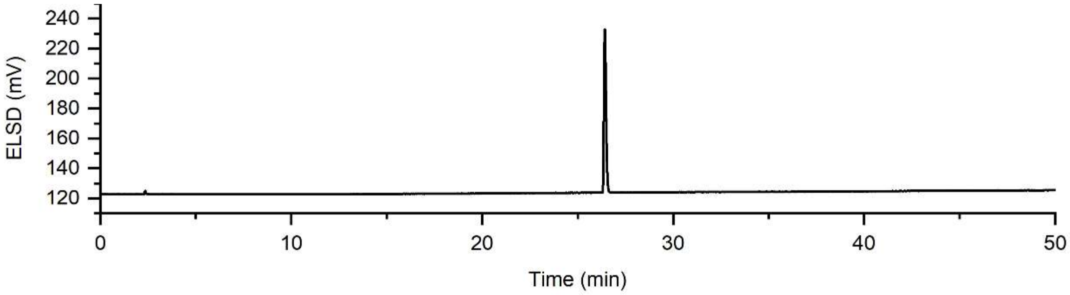

^{1}H NMR (600 MHz, D_2O) δ 4.71 (d, *J* = 8.3 Hz, 1H, **$H_1β$**), 4.49 (d, J = 7.9, 1H, **$H_1β$**), 4.49 (d, *J* = 7.9, 1H, **$H_1β$**), 4.44 (d, *J* = 7.9 Hz, 1H, **$H_1β$**), 4.17 (d, *J* = 3.4 Hz, 1H), 4.04 – 3.92 (m, 4H), 3.86 (dd, *J* = 12.4, 4.6 Hz, 1H), 3.84 – 3.66 (m, 13H), 3.67 – 3.63 (m, 2H), 3.63 – 3.58 (m, 3H), 3.55 (dd, *J* = 9.9, 7.8 Hz, 1H), 3.34 – 3.29 (m, 1H), 3.02 (d, *J* = 7.7 Hz, 2H), 2.04 (s, 3H), 1.76 – 1.62 (m, 4H), 1.47 (q, *J* = 8.1 Hz, 2H).

^{13}C NMR (151 MHz, D_2O) δ 174.9, 102.9 (**C_1**), 102.9 (**C_1**), 102.7 (**C_1**), 102.0 (**C_1**), 82.1, 78.4, 78.2, 75.3, 74.9, 74.8, 74.5, 74.4, 72.8, 72.5, 72.2, 71.0, 70.1, 69.9, 68.5, 68.3, 61.0, 60.9, 60.1, 59.9, 55.2, 39.3, 28.1, 26.4, 22.2, 22.1.

HRMS (QToF): Calcd for $C_{31}H_{57}N_2O_{21}$ a $[M + H]^+$ 793.3449; found 793.3465.

^{1}H NMR (600 MHz, D_2O) of **LNnT (35)**:

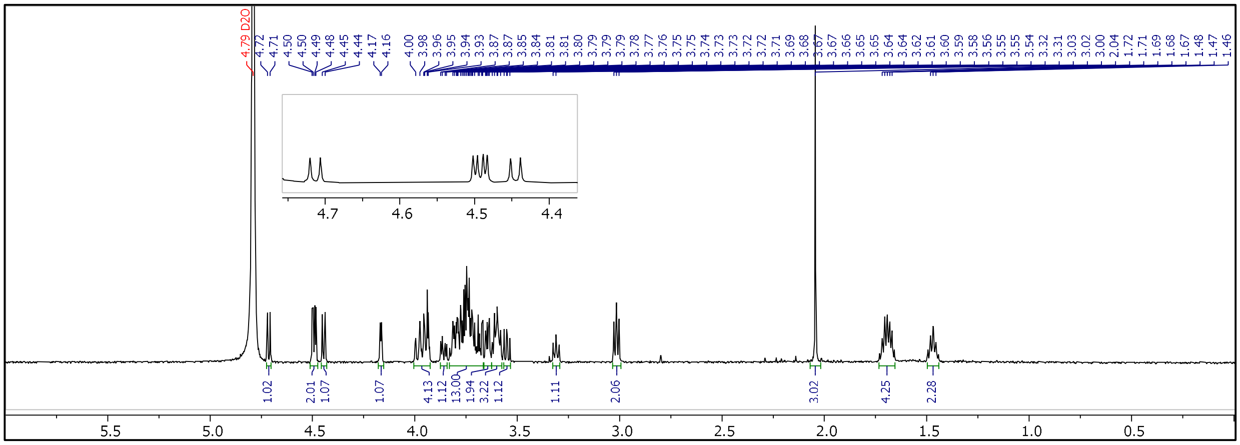

^{13}C NMR (151 MHz, D_2O) of **LNnT (35)**:

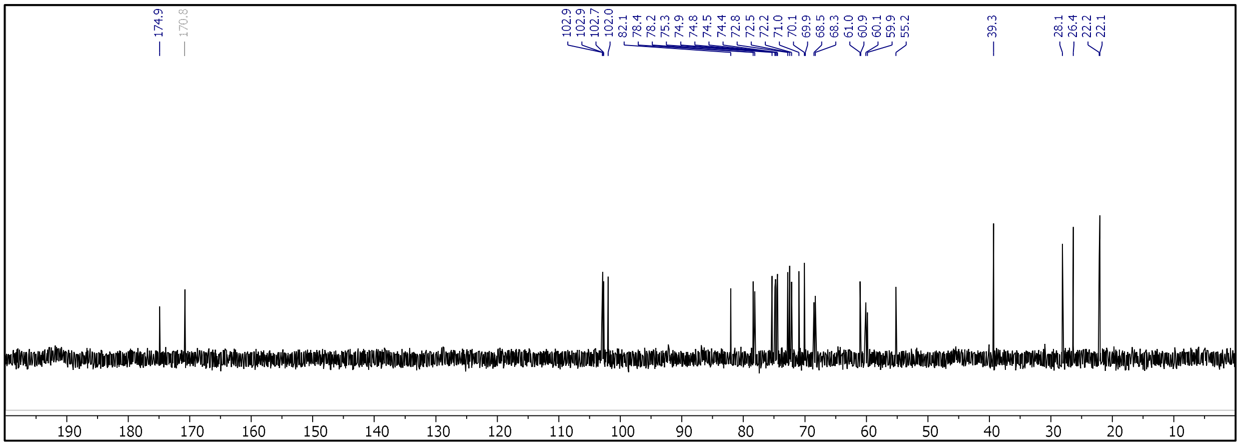

^{1}H-^{13}C HSQC NMR of **LNnT 35**:

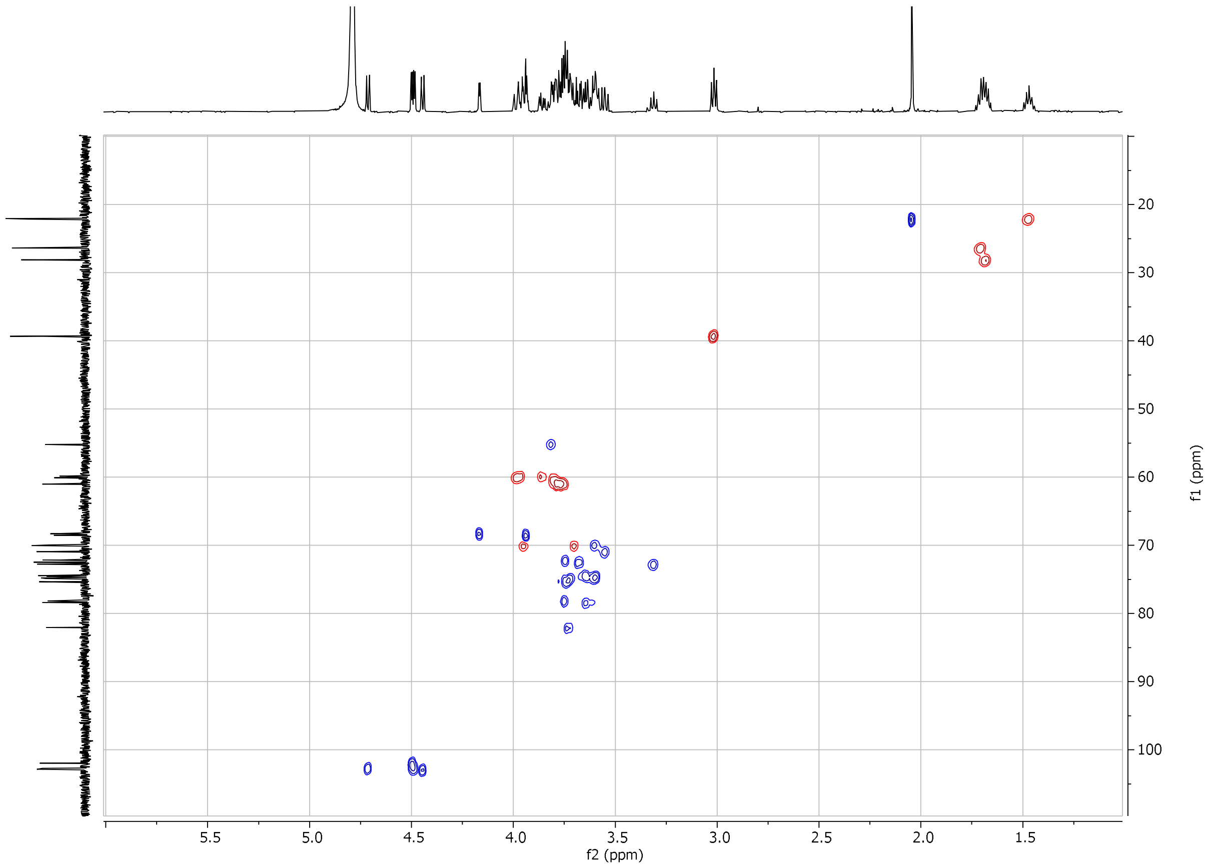

6.5 *para*-Lacto-*N*-neohexaose ***p*LNnH 36**

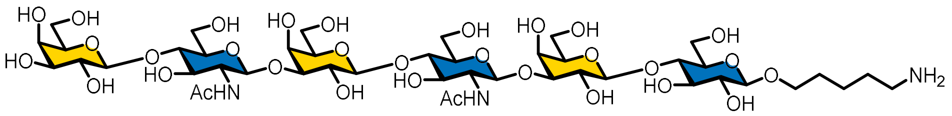

Deprotection procedure was shown as **General Deprotection Procedure** (Section: 4.4 Post-synthesizer Manipulation). Compound ***p*LNnH (36)** (2.2 mg, 1.9 µmol, 52%) was obtained as a white solid by purification using preparative RP-HPLC (**Method H**) and lyophilizer.

(A) Analytical RP-HPLC of the crude ***p*LNnH (36)** (**Method E**, ELSD trace, t_R = 31.5 min)

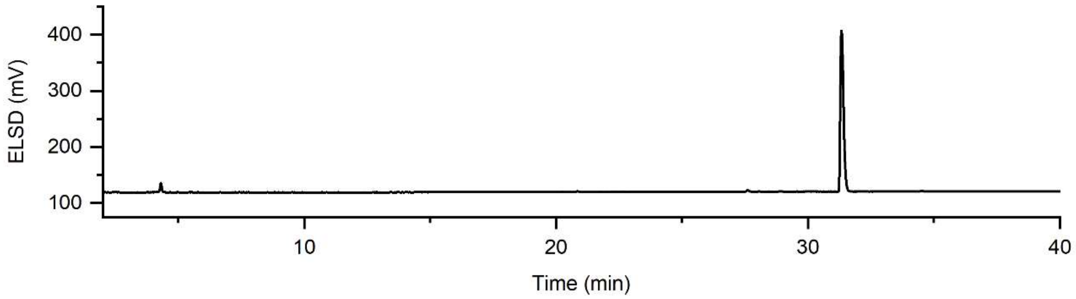

(B) Analytical RP-HPLC of the pure ***p*LNnH (36)** (**Method E**, ELSD trace, t_R = 31.3 min)

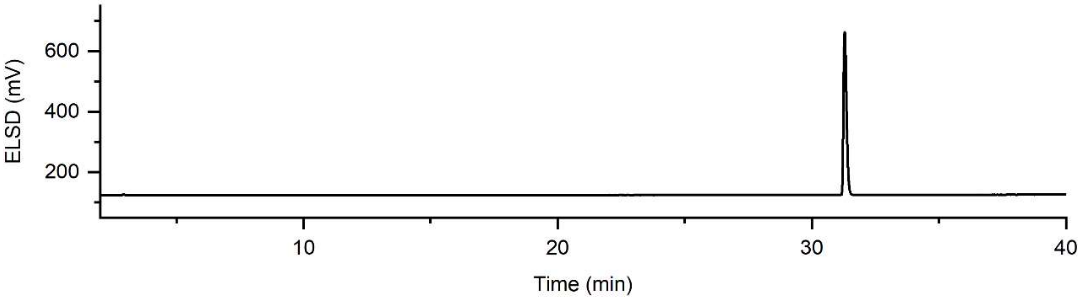

^{1}H NMR (600 MHz, D_2O) δ 4.71 (d, *J* = 8.3 Hz, 2H, **H_1β, H_1β**), 4.49 (d, *J* = 8.1 Hz, 1H, **H_1β**), 4.49 (d, *J* = 7.7 Hz, 1H, **H_1β**), 4.48 (d, *J* = 7.7 Hz, 1H, **H_1β**), 4.44 (d, J = 7.9 Hz, 1H, **H_1β**), 4.17 (d, *J* = 3.4 Hz, 1H), 4.16 (d, *J* = 3.1 Hz, 1H), 4.06 – 3.92 (m, 5H), 3.91 – 3.66 (m, 22H), 3.66 – 3.57 (m, 7H), 3.55 (dd, *J* = 9.9, 7.9 Hz, 1H), 3.37 – 3.27 (m, 1H), 3.02 (d, *J* = 7.1 Hz, 2H), 2.04 (s, 3H), 2.04 (s, 3H), 1.77 – 1.63 (m, 4H), 1.47 (p, *J* = 7.6 Hz, 2H).

^{13}C NMR (151 MHz, D_2O) δ 174.9, 102.93 (**C_1**), 102.88 (**C_1**), 102.85 (**C_1**), 102.74 (**C_1**), 102.73 (**C_1**), 102.0 (**C_1**), 82.07, 82.05, 78.4, 78.18, 78.15, 75.3, 74.9, 74.8, 74.5, 74.4, 72.8, 72.5, 72.2, 70.9, 70.1, 69.9, 68.5, 68.3, 61.0, 60.9, 60.1, 59.8, 55.17, 55.14, 39.3, 28.1, 26.4, 22.2, 22.1.

HRMS (QToF): Calcd for $C_{45}H_{80}N_3O_{31}$ $[M + H]^+$ 1158.4771; found 1158.4819.

^{1}H NMR (600 MHz, D_2O) of ***p*****LNnH (36)**:

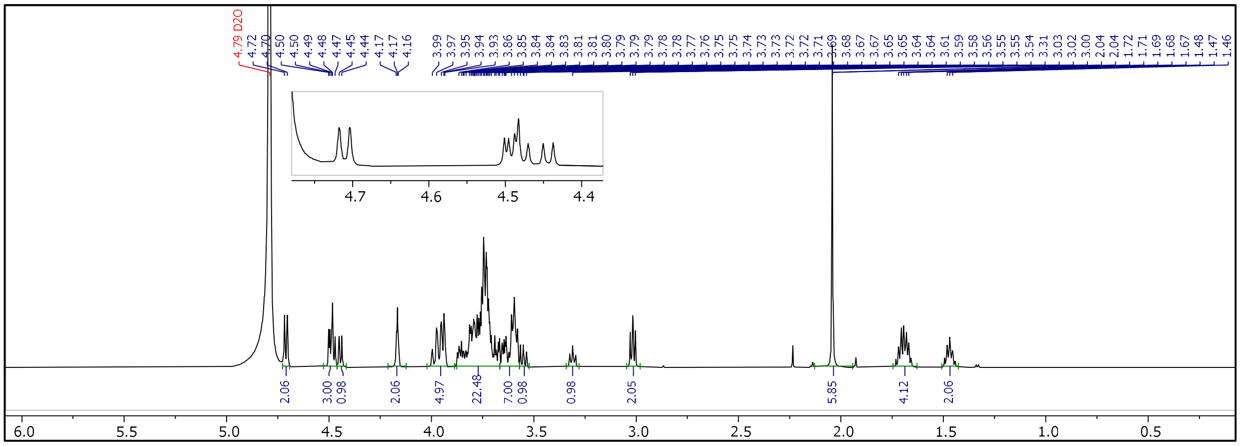

^{13}C NMR (151 MHz, D_2O) of ***p*****LNnH (36)**:

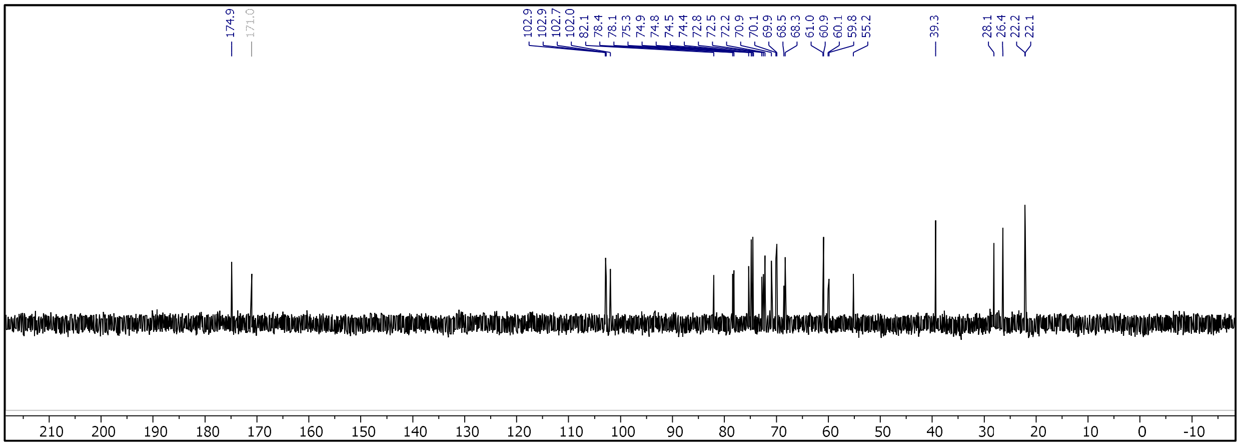

^{1}H-^{13}C HSQC NMR of ***p*LNnH 36**:

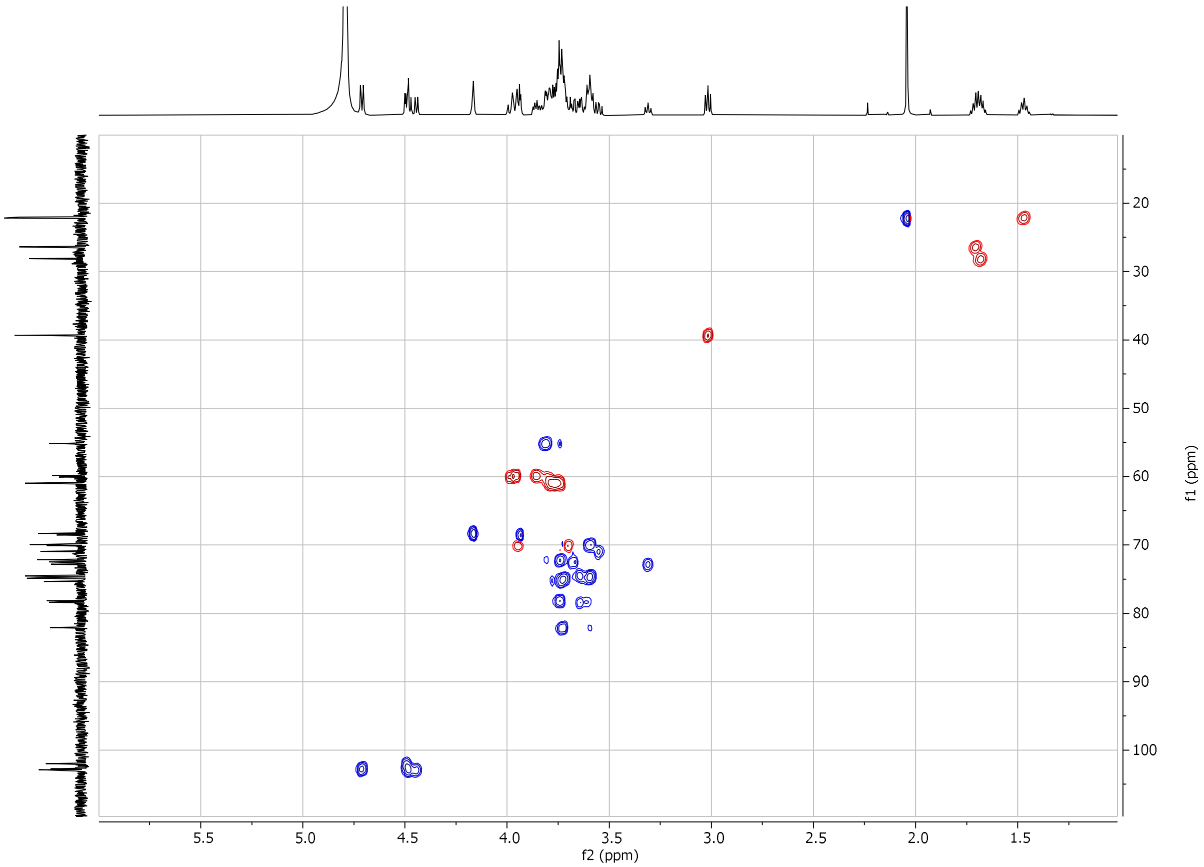

<u>6.6 Lacto-*N*-tetraose **LNT 37**</u>

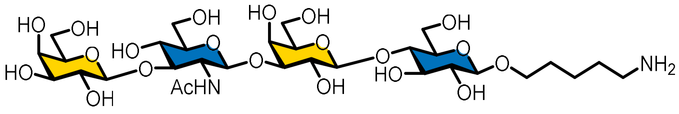

Deprotection procedure was shown as **General Deprotection Procedure** (Section: <u>4.4 Post-synthesizer Manipulation</u>). Compound **LNT (37)** (1.9 mg, 2.4 μmol, 57%) was obtained as a white solid by purification using preparative RP-HPLC (**Method H**) and lyophilizer.

(A) Analytical RP-HPLC of the crude **LNT (37)** (**Method E**, ELSD trace, t_R = 28.0 min)

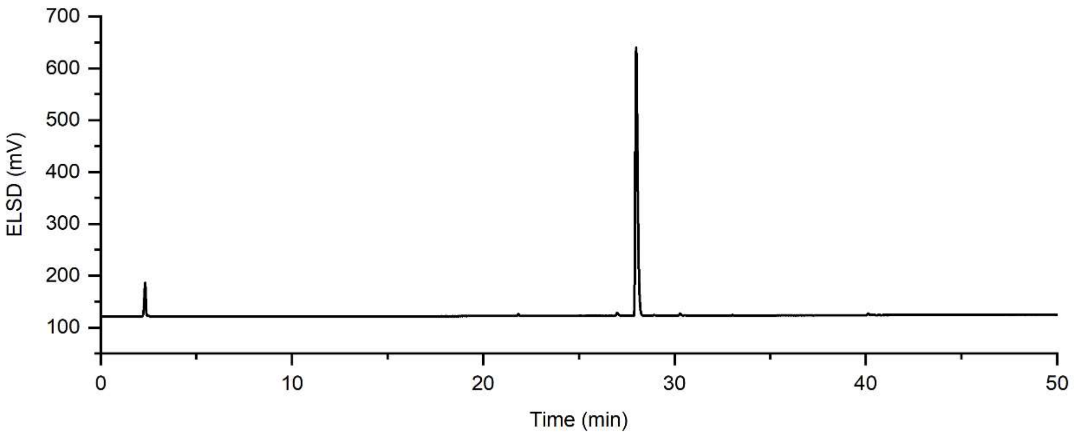

(B) Analytical RP-HPLC of the pure **LNT (37)** (**Method E**, ELSD trace, t_R = 27.7 min)

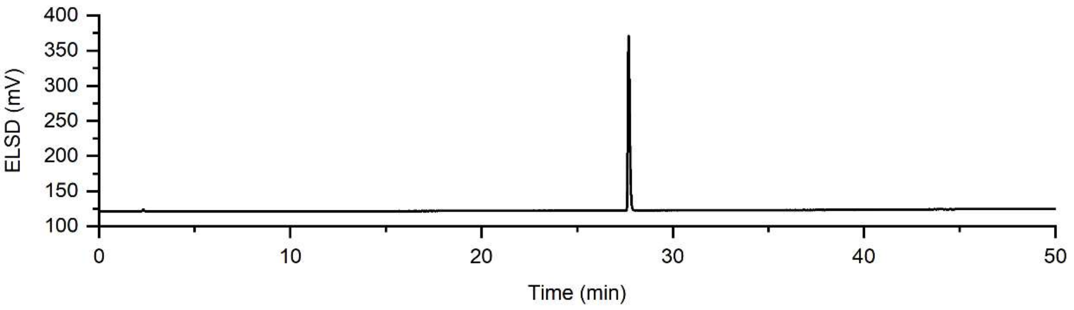

^{1}H NMR (600 MHz, D_2O) δ 4.74 (d, *J* = 8.5 Hz, 1H, **$H_1\beta$**), 4.50 (d, *J* = 8.0 Hz, 1H, **$H_1\beta$**), 4.45 (d, *J* = 7.7 Hz, 1H, **$H_1\beta$**), 4.45 (d, *J* = 7.8 Hz, 1H, **$H_1\beta$**), 4.17 (d, *J* = 3.1 Hz, 1H), 3.99 (dd, *J* = 12.3, 1.7 Hz, 1H), 3.96 – 3.88 (m, 4H), 3.87 – 3.74 (m, 7H), 3.74 – 3.67 (m, 4H), 3.67 – 3.50 (m, 7H), 3.49 (qd, J = 7.3, 2.6 Hz, 1H), 3.31 (t, *J* = 8.5 Hz, 1H), 3.02 (t, *J* = 7.5 Hz, 2H), 2.04 (s, 3H), 1.74 – 1.64 (m, 4H), 1.47 (p, *J* = 7.5 Hz, 2H).

^{13}C NMR (151 MHz, D_2O) δ 175.0, 103.5 (**C_1**), 102.9 (**C_1**), 102.5 (**C_1**), 102.0 (**C_1**), 82.1, 78.4, 75.3, 75.2, 74.9, 74.8, 74.4, 72.8, 72.5, 70.7, 70.1, 70.0, 68.5, 68.4, 68.3, 61.0, 60.9, 60.5, 60.1, 54.7, 39.3, 28.1, 26.4, 22.2, 22.1.

HRMS (QToF): Calcd for $C_{31}H_{57}N_2O_{21}$ a $[M + H]^+$ 793.3449; found 793.3465.

^{1}H NMR (600 MHz, D_2O) of **LNT (37)**:

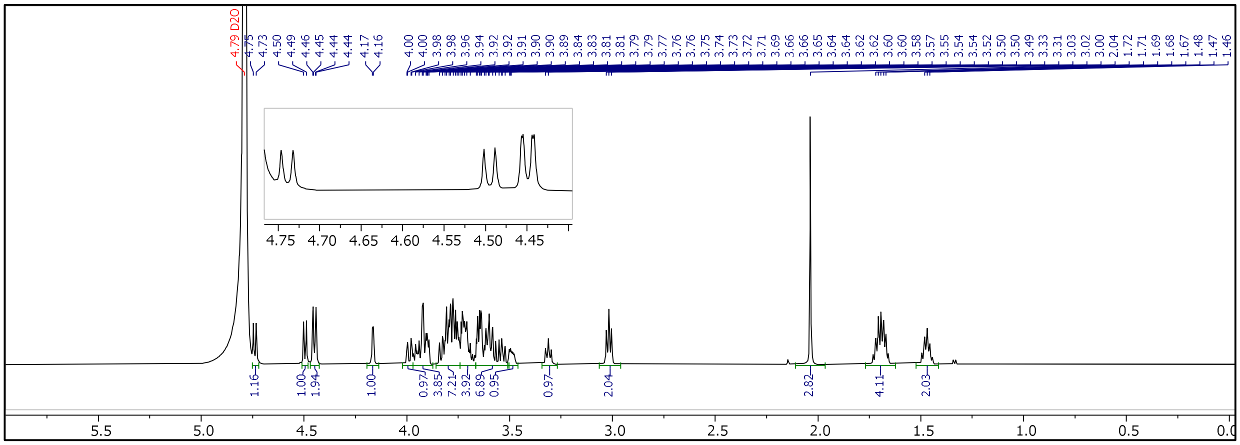

^{13}C NMR (151 MHz, D_2O) of **LNT 37**:

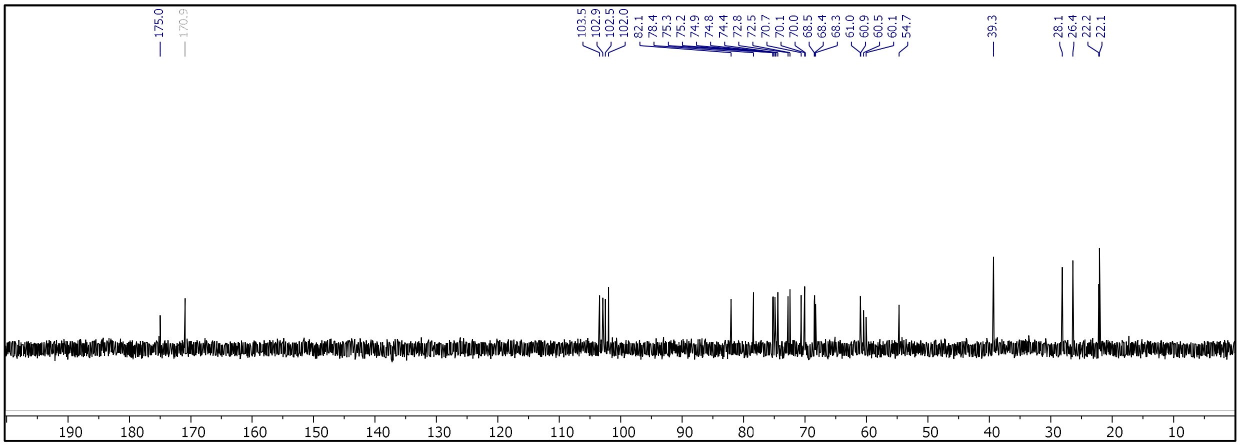

^1H-^{13}C HSQC NMR of **LNT 37**:

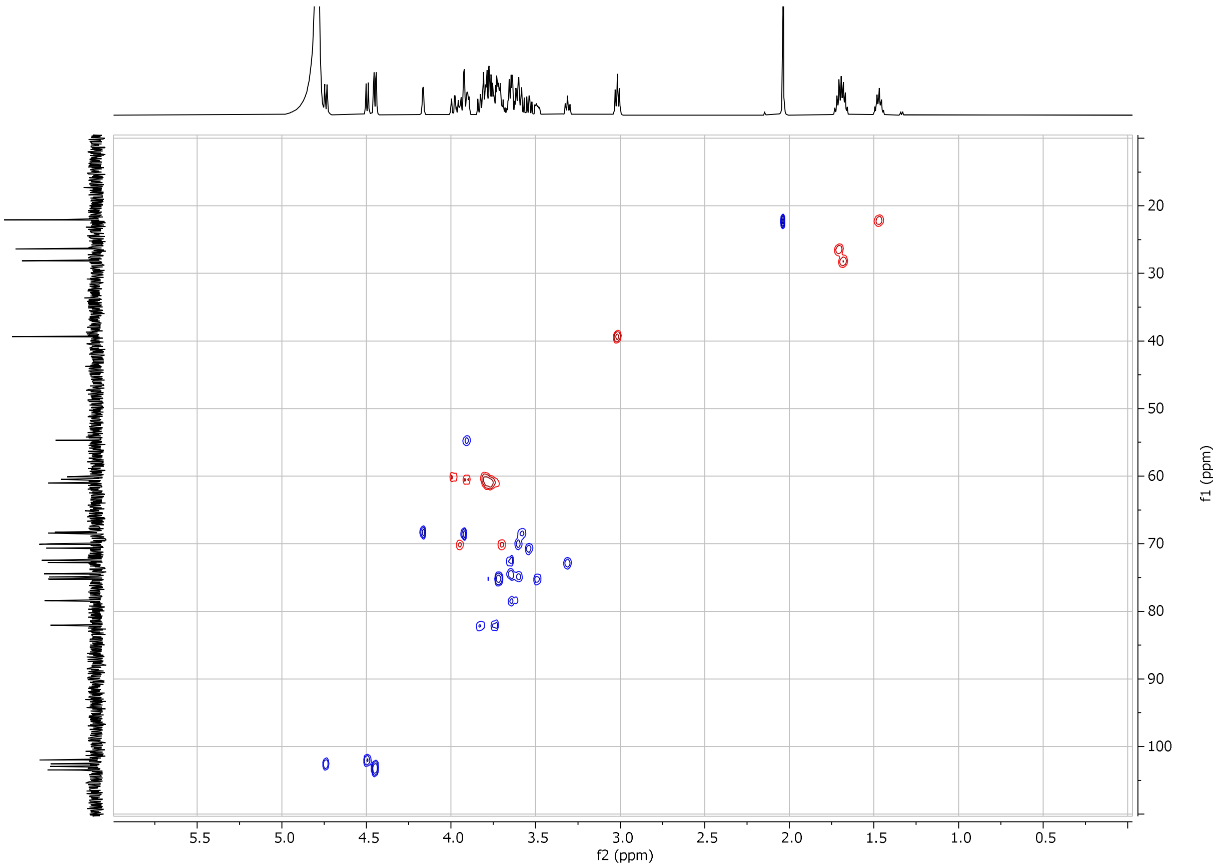

6.7 Lacto-*N*-fucopentaose III **LNFP III 38**

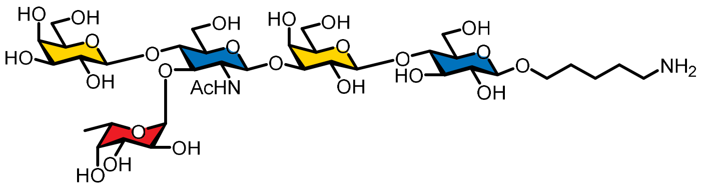

Deprotection procedure was shown as **General Deprotection Procedure** (Section: 4.4 Post-synthesizer Manipulation). Compound **LNFP III (38)** (2.0 mg, 2.1 µmol, 55%) was obtained as a white solid by purification using preparative RP-HPLC (**Method H**) and lyophilizer.

(A) Analytical RP-HPLC of the crude **LNFP III (38)** (**Method E**, ELSD trace, t_R = 22.4 min)

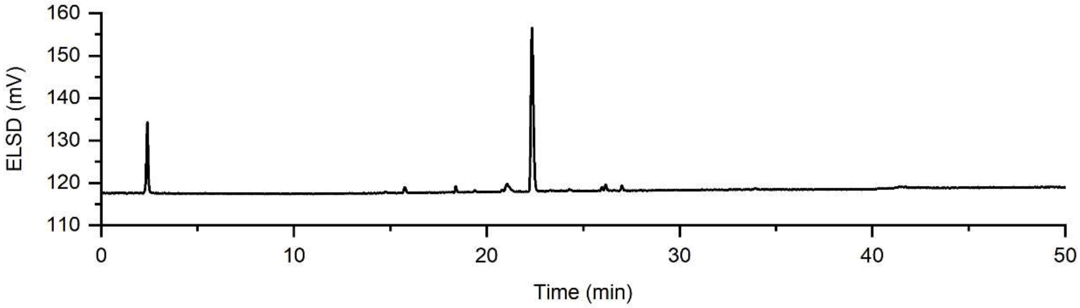

(B) Analytical RP-HPLC of the pure **LNFP III (38)** (**Method E**, ELSD trace, t_R = 22.3 min)

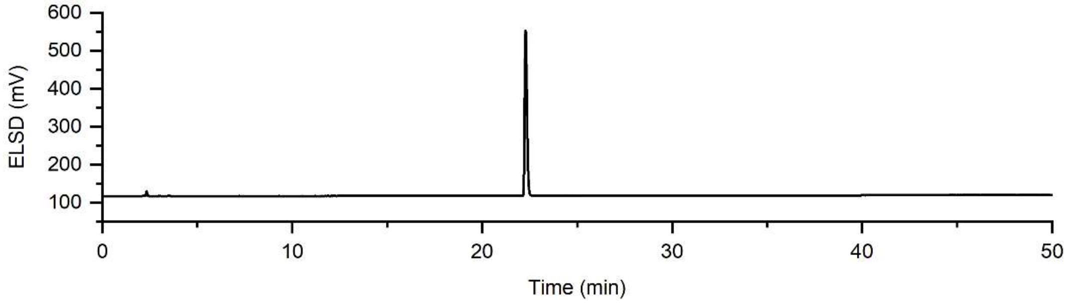

^{1}H NMR (600 MHz, D_2O) δ 5.14 (d, *J* = 4.0 Hz, 1H, **$H_1α$**), 4.85 (d, *J* = 6.9 Hz, 1H), 4.72 (d, *J* = 8.4 Hz, 1H, **$H_1β$**), 4.49 (d, *J* = 8.0 Hz, 1H, **$H_1β$**), 4.47 (d, *J* = 7.8 Hz, 1H, **$H_1β$**), 4.44 (d, *J* = 7.9 Hz, 1H, **$H_1β$**), 4.17 (d, *J* = 3.3 Hz, 1H), 4.03 – 3.85 (m, 9H), 3.85 – 3.68 (m, 10H), 3.68 – 3.56 (m, 7H), 3.51 (dd, *J* = 9.8, 7.9 Hz, 1H), 3.33 – 3.28 (m, 1H), 3.02 (t, *J* = 7.5 Hz, 2H), 2.03 (s, 3H), 1.75 – 1.63 (m, 4H), 1.47 (p, *J* = 7.5 Hz, 2H), 1.18 (d, *J* = 6.9 Hz, 3H).

^{13}C NMR (151 MHz, D_2O) δ 174.7, 102.9 (**C_1**), 102.5 (**C_1**), 102.0(**C_1**), 101.8(**C_1**), 98.6(**C_1**), 82.1, 78.4, 75.1, 74.9, 74.9, 74.8, 74.7, 74.4, 73.0, 72.8, 72.5, 71.9, 71.0, 70.1, 69.9, 69.2, 68.3, 68.3, 67.7, 66.7, 61.5, 60.9, 60.1, 59.6, 56.0, 39.3, 28.1, 26.4, 22.2, 22.1, 15.3.

HRMS (QToF): Calcd for $C_{37}H_{67}N_2O_{25}$ a $[M + H]^+$ 939.4033; found 939.4088.

^{1}H NMR (600 MHz, D_2O) of **LNFP III 38**:

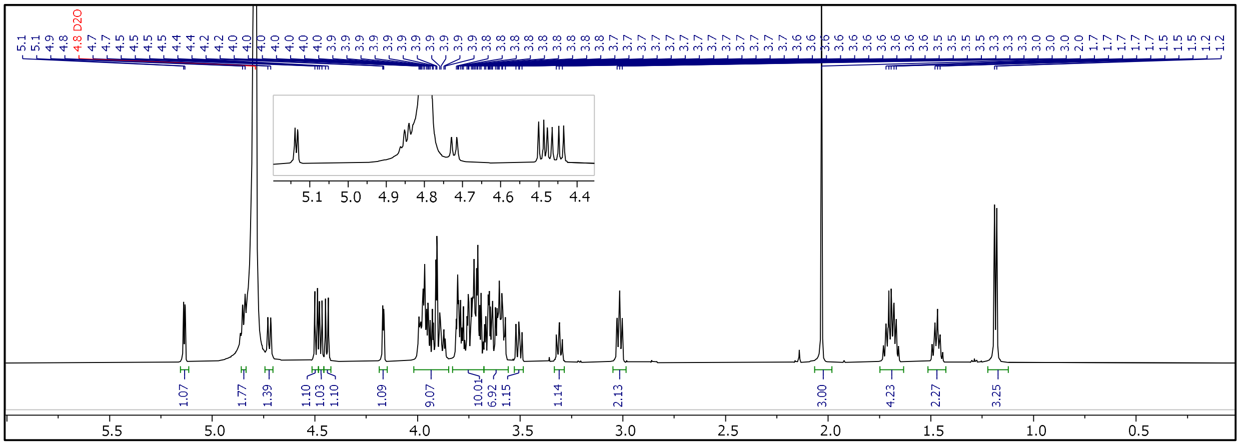

^{13}C NMR (151 MHz, D_2O) of **LNFP III 38**:

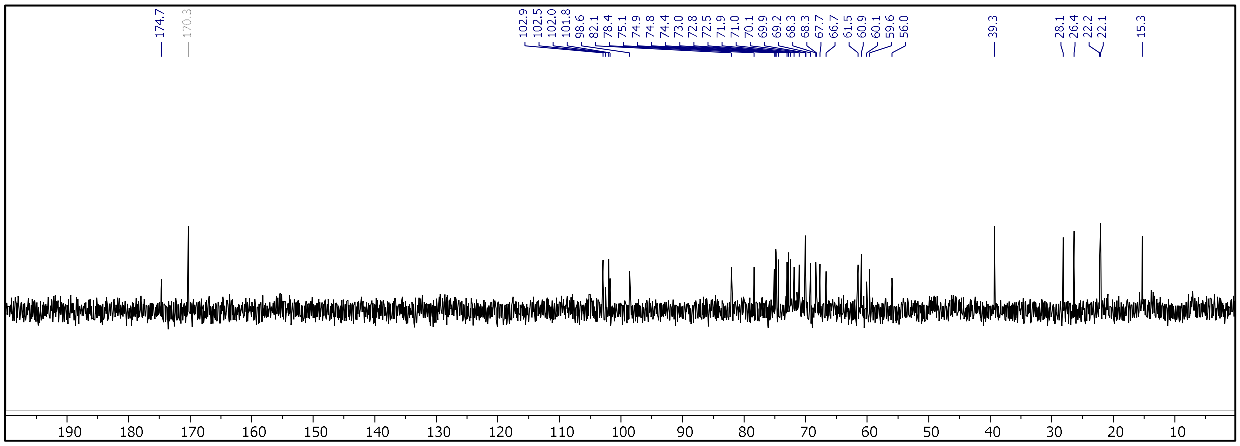

^{1}H-^{13}C HSQC NMR of **LNFP III 38**:

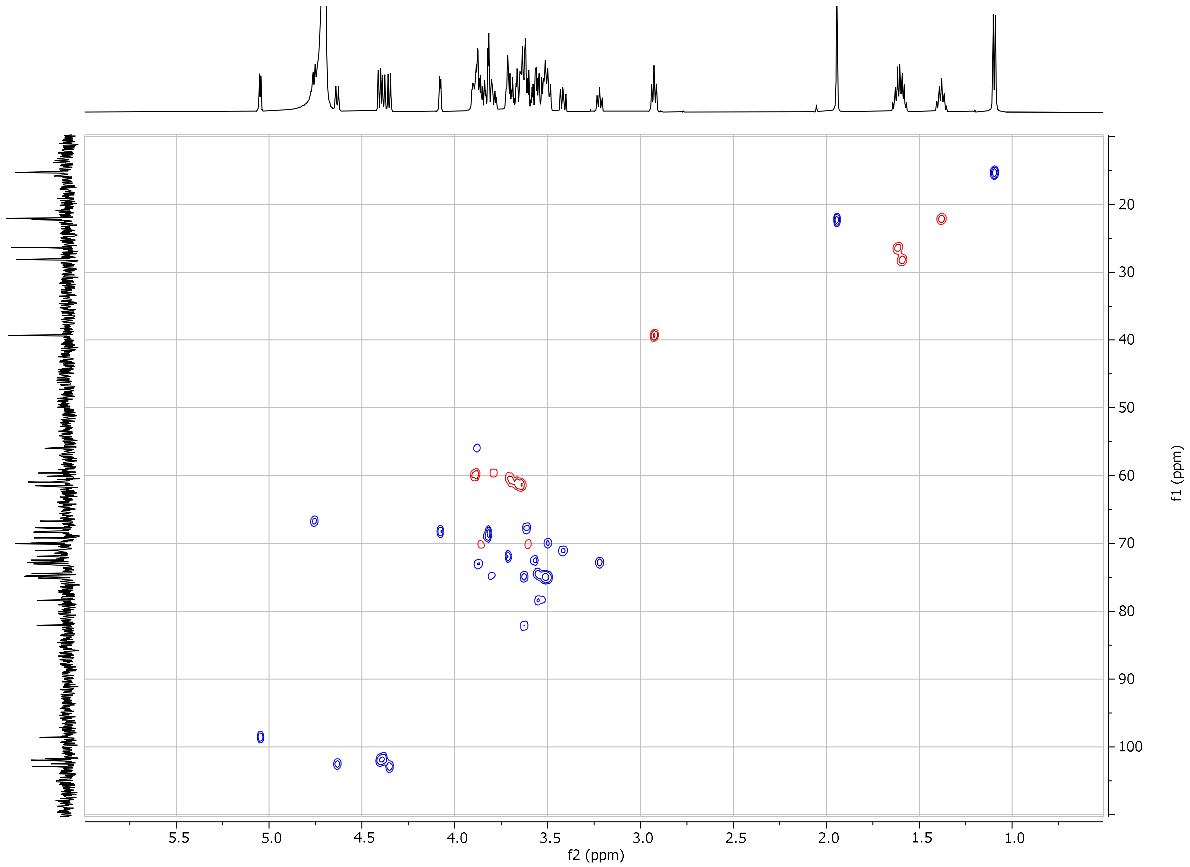

6.8 Lacto-*N*-fucopentaose II **LNFP II 39**

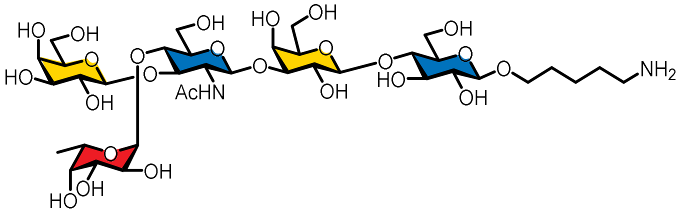

Deprotection procedure was shown as **General Deprotection Procedure** (Section: 4.4 Post-synthesizer Manipulation). Compound **LNFP II 39** (1.3 mg, 1.4 μmol, 50%) was obtained as a white solid by purification using preparative RP-HPLC (**Method J**) and lyophilizer.

(A) Analytical RP-HPLC of crude **LNFP II 39** (**Method G**, ELSD trace, t_R = 26.6 min)

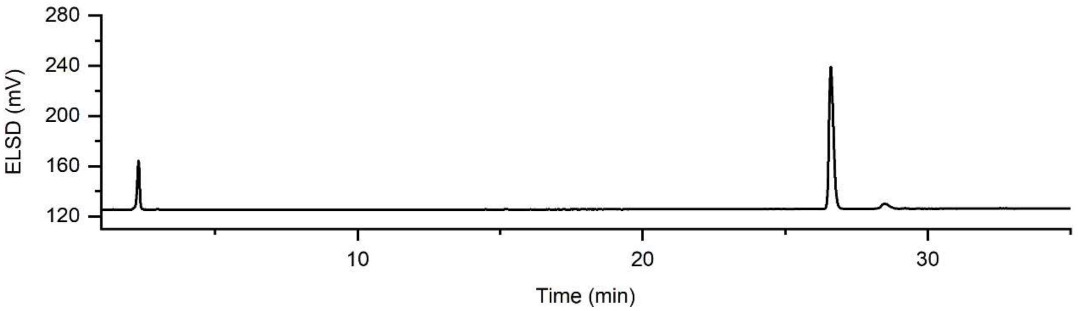

(B) Analytical RP-HPLC of pure **LNFP II 39** (**Method G**, ELSD trace, t_R = 28.4min)

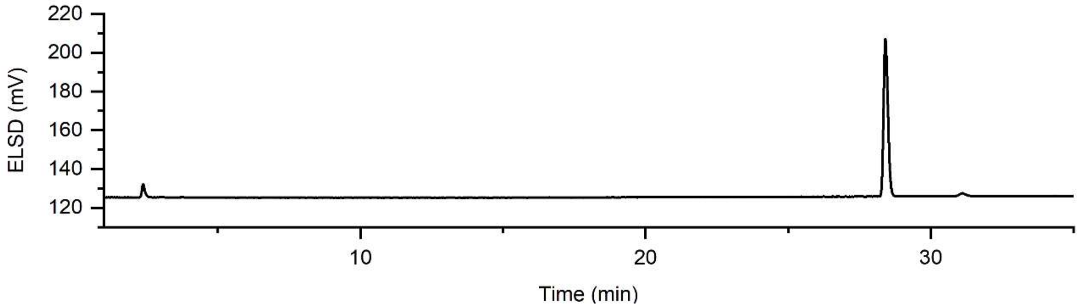

^{1}H NMR (700 MHz, D_2O) δ 5.04 (d, *J* = 3.9 Hz, 1H, **$H_1α$**), 4.89 (d, *J* = 6.5 Hz, 1H), 4.72 (d, *J* = 8.6 Hz, 1H, **$H_1β$**), 4.52 (d, *J* = 7.7 Hz, 1H, **$H_1β$**), 4.50 (d, *J* = 8.0 Hz, 1H, **$H_1β$**), 4.45 (d, *J* = 7.9 Hz, 1H, **$H_1β$**), 4.17 (d, *J* = 3.1 Hz, 1H), 4.10 (t, *J* = 9.8 Hz, 1H), 4.02 – 3.93 (m, 4H), 3.93 – 3.85 (m, 3H), 3.84 – 3.69 (m, 11H), 3.69 – 3.57 (m, 6H), 3.56 (dt, *J* = 9.8, 3.2 Hz, 1H), 3.50 (dd, *J* = 9.8, 7.8 Hz, 1H), 3.32 (t, *J* = 8.6 Hz, 1H), 3.02 (t, *J* = 7.5 Hz, 2H), 2.05 (s, 3H), 1.78 – 1.64 (m, 4H), 1.48 (p, *J* = 7.7 Hz, 2H), 1.20 (d, *J* = 6.5 Hz, 3H).

^{13}C NMR (151 MHz, D_2O) δ 174.8, 171.0, 102.9 (**C_1**), 102.8 (**C_1**), 102.6 (**C_1**), 102.0 (**C_1**), 98.0 (**C_1**), 82.1, 78.4, 75.9, 75.2, 74.9, 74.8, 74.8, 74.4, 72.8, 72.3, 72.1, 71.9, 70.5, 70.1, 69.9, 69.1, 68.3, 68.3, 67.8, 66.8, 61.6, 60.9, 60.1, 59.6, 55.9, 39.3, 28.1, 26.4, 22.3, 22.1, 15.3.

HRMS (QToF): Calcd for $C_{37}H_{67}N_2O_{25}$ a $[M + H]^+$ 939.4033; found 939.4032.

^{1}H NMR (700 MHz, D_2O) of **LNFP II (39)**:

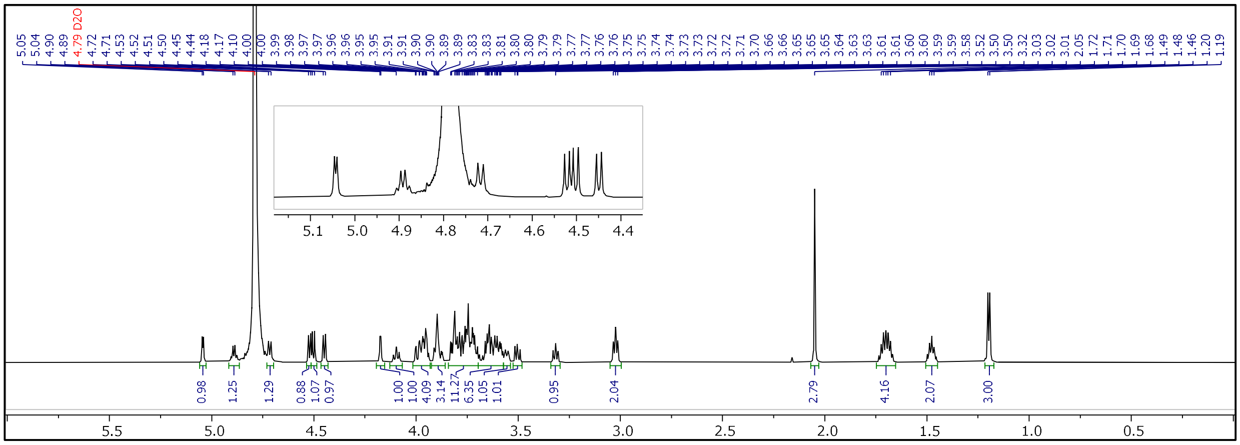

^{13}C NMR (151 MHz, D_2O) of **LNFP II (39)**:

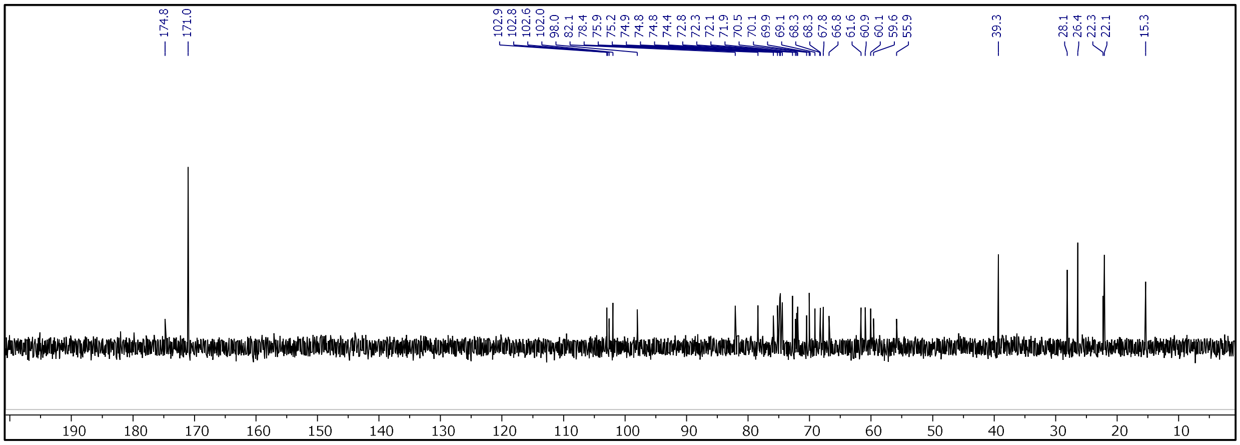

^{1}H-^{13}C HSQC NMR of **LNFP II 39**:

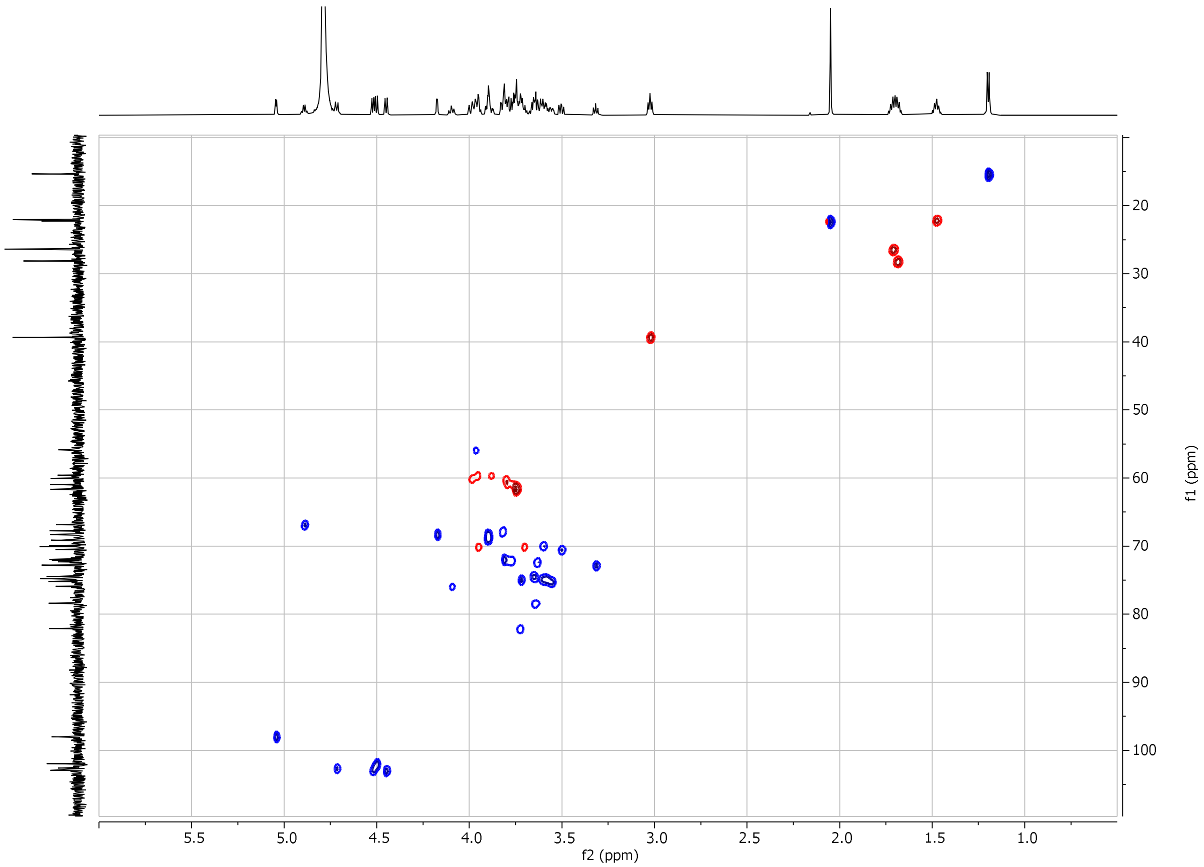

6.9 Lacto-*N*-neohexaose **LNnH 40**

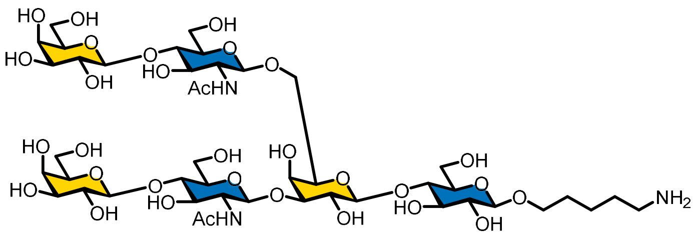

Deprotection procedure was shown as **General Deprotection Procedure** (Section: 4.4 Post-synthesizer Manipulation). Compound **LNnH (40)** (2.0 mg, 1.7 µmol, 45%) was obtained as a white solid by purification using preparative RP-HPLC (**Method H**) and lyophilizer.

(A) Analytical RP-HPLC of crude **LNnH 40** (**Method E**, ELSD trace, t_R = 28.5 min)

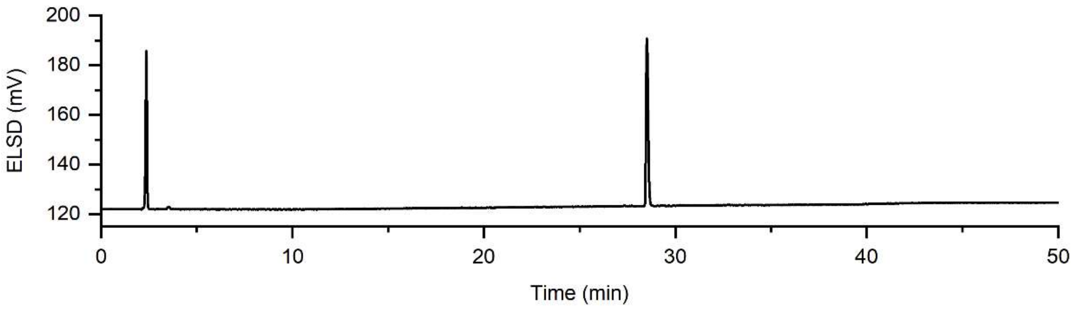

(B) Analytical RP-HPLC of pure **LNnH 40** (**Method E**, ELSD trace, t_R = 28.7 min)

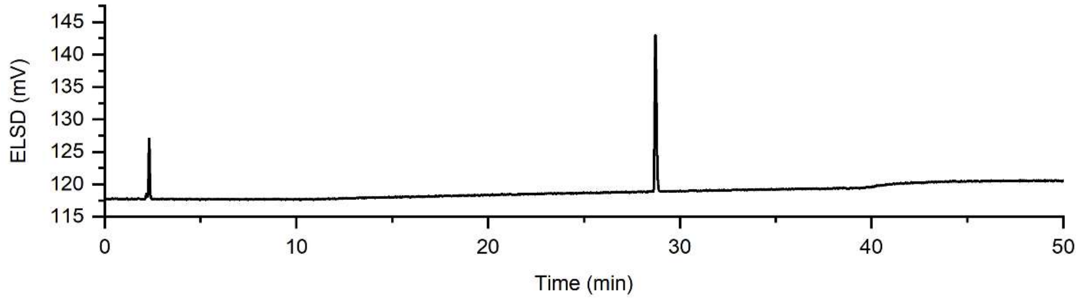

^{1}H NMR (700 MHz, D_2O) δ 4.73 (d, *J* = 8.4 Hz, 1H, **$H_1β$**), 4.66 (d, *J* = 8.1 Hz, 1H, **$H_1β$**), 4.52 (d, *J* = 8.3 Hz, 1H, **$H_1β$**), 4.51 (d, *J* = 7.9 Hz, 1H, **$H_1β$**), 4.50 (d, *J* = 7.8 Hz, 1H, **$H_1β$**), 4.46 (d, *J* = 7.9 Hz, 1H, **$H_1β$**), 4.18 (d, *J* = 3.1 Hz, 1H), 4.06 – 3.93 (m, 7H), 3.94 – 3.85 (m, 4H), 3.85 – 3.53 (m, 25H), 3.34 (dd, *J* = 9.2, 8.1 Hz, 1H), 3.04 (t, *J* = 7.5 Hz, 2H), 2.09 (s, 3H), 2.06 (s, 3H), 1.82 – 1.65 (m, 4H), 1.49 (q, *J* = 8.0 Hz, 2H).

^{13}C NMR (151 MHz, D_2O) δ 174.9, 174.5, 171.0, 103.0 (**C_1**), 102.88 (**C_1**), 102.85 (**C_1**), 102.7 (**C_1**), 102.0 (**C_1**), 101.0 (**C_1**), 81.8, 79.0, 78.4, 78.2, 75.3, 74.7, 74.7, 74.6, 74.5, 73.5, 72.9, 72.5, 72.2, 71.0, 70.1, 69.8, 68.5, 68.5, 68.4, 61.0, 60.04, 60.02, 59.9, 55.2, 55.0, 39.4, 28.1, 26.4, 23.2, 22.4, 22.2, 22.1.

HRMS (QToF): Calcd for $C_{45}H_{80}N_3O_{31}$ a $[M + H]^+$ 1158.4771; found 1158.4825.

^{1}H NMR (600 MHz, D_2O) of **LNnH 40**:

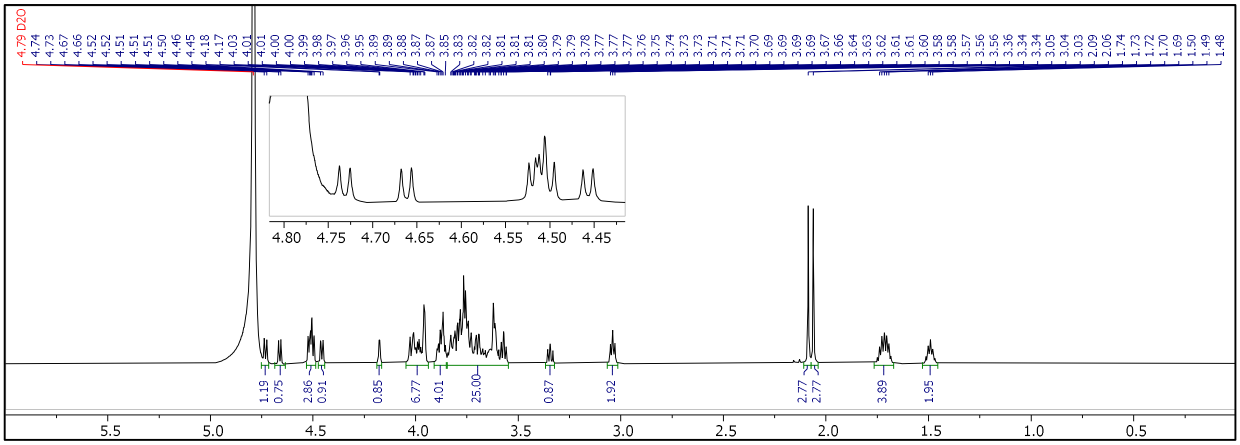

^{13}C NMR (151 MHz, D_2O) of **LNnH 40**:

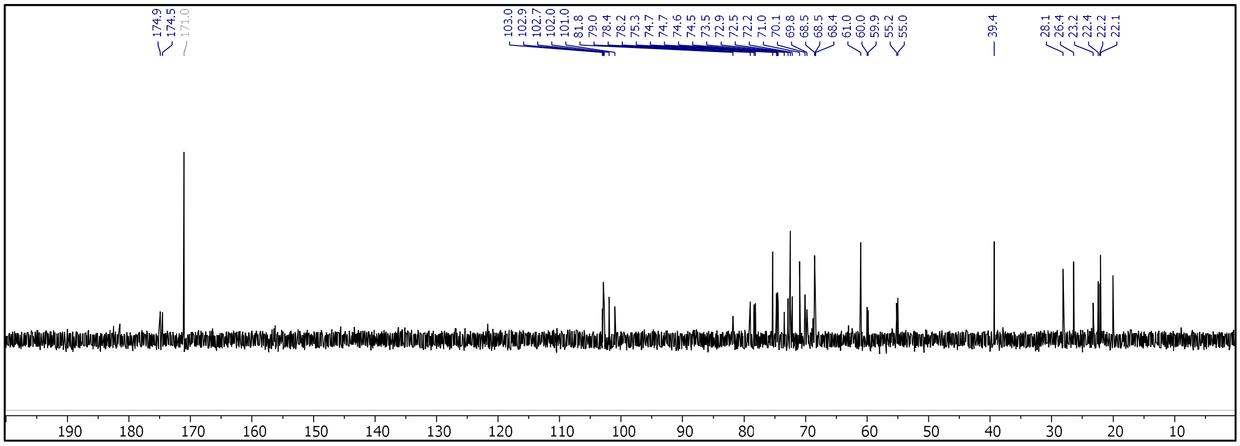

^{1}H-^{13}C HSQC NMR of **LNnH 40**:

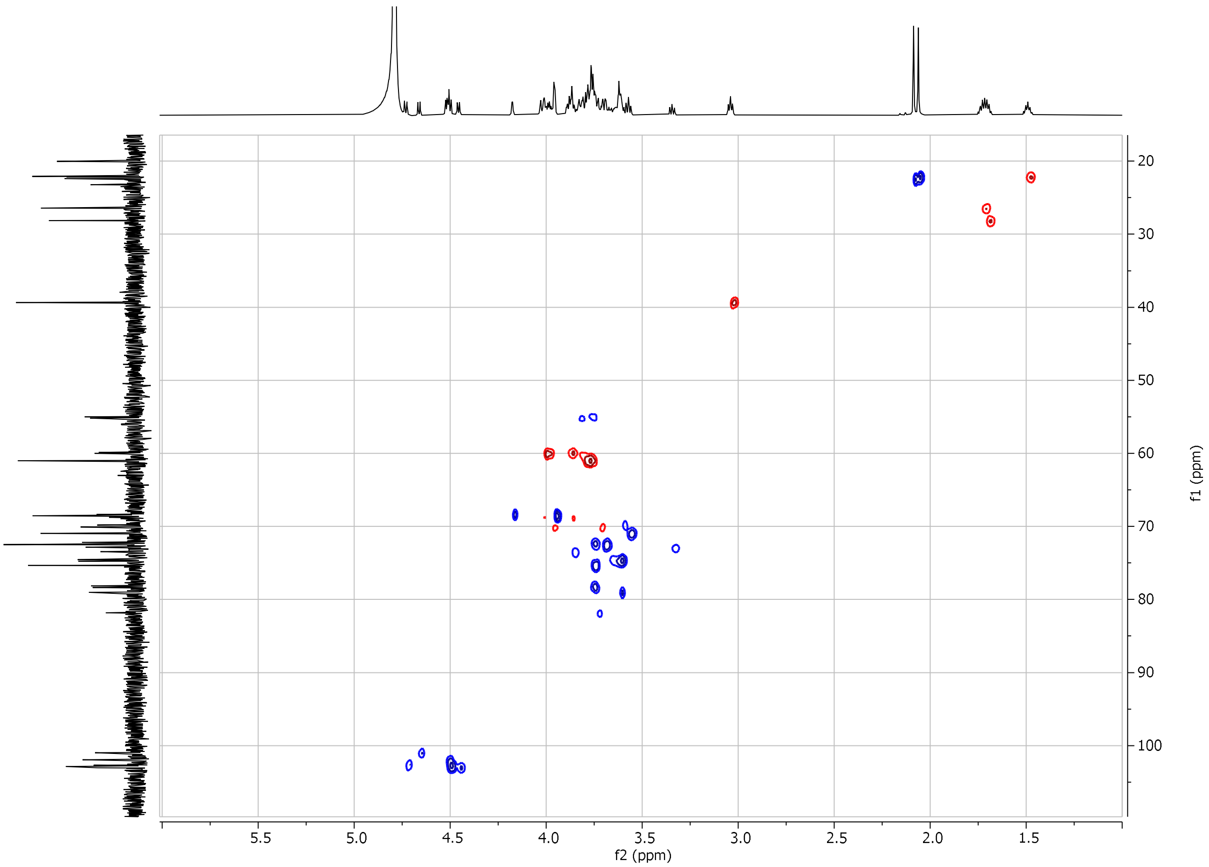

6.10 Difucosyllacto-*N*-neohexaose **DF-LNnH 41**

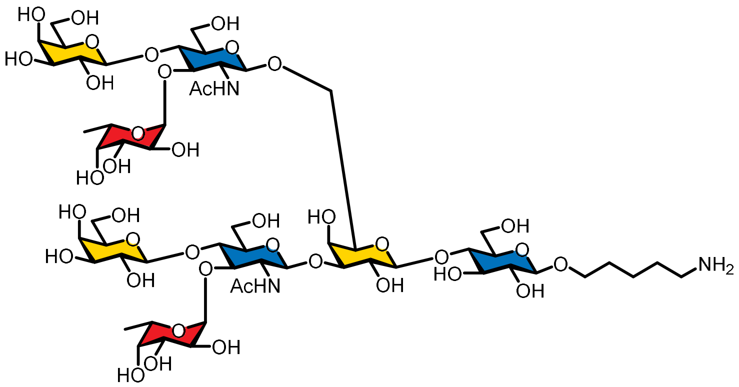

Deprotection procedure was shown as **General Deprotection Procedure** (Section: 4.4 Post-synthesizer Manipulation). Compound **DFLNnH 41** (1.6 mg, 1.1 µmol, 42%) was obtained as a white solid by purification using preparative RP-HPLC (**Method H**) and lyophilizer.

(A) Analytical RP-HPLC of crude **DFLNnH 41** (**Method E**, ELSD trace, t_R = 24.8 min)

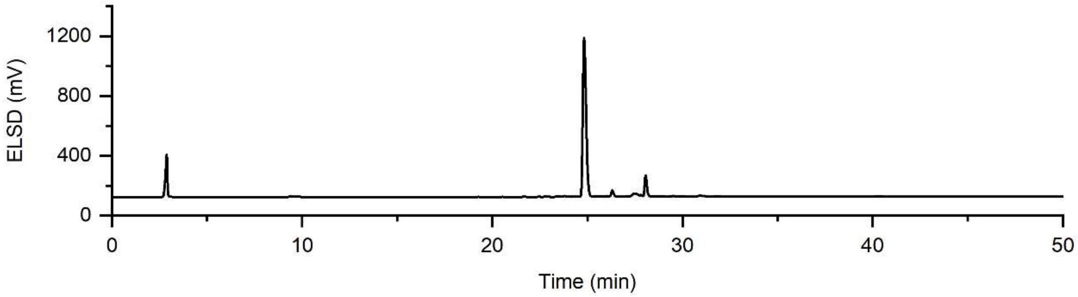

(B) Analytical RP-HPLC of the pure **DFLNnH 41** (**Method E**, ELSD trace, t_R = 24.6 min)

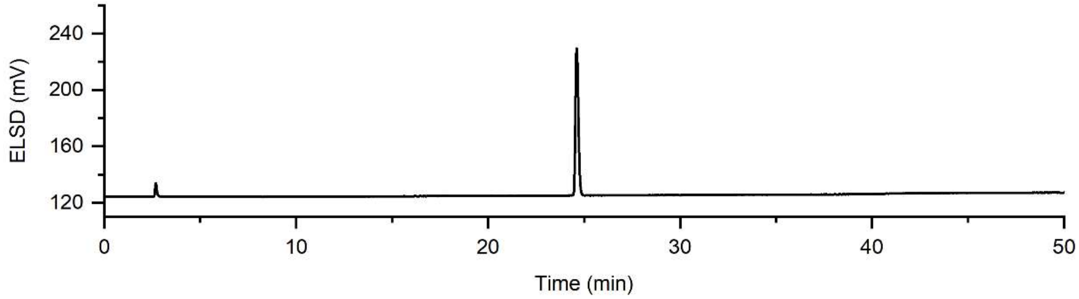

^{1}H NMR (700 MHz, D_2O) δ 5.10 (d, *J* = 3.7 Hz, 1H, **$H_1α$**), 5.08 (d, *J* = 3.9 Hz, 1H, **$H_1α$**), 4.68 (d, *J* = 8.2 Hz, 1H, **$H_1β$**), 4.61 (d, *J* = 7.7 Hz, 1H, **$H_1β$**), 4.46 (d, *J* = 8.0 Hz, 1H, **$H_1β$**), 4.44 (d, *J* = 8.6 Hz, 1H, **$H_1β$**), 4.43 (d, *J* = 9.2 Hz, 1H, **$H_1β$**), 4.40 (d, *J* = 7.8 Hz, 1H, **$H_1β$**), 4.12 (d, *J* = 3.5 Hz, 1H), 4.00 – 3.75 (m, 22H), 3.75 – 3.51 (m, 19H), 3.47 (t, *J* = 8.7 Hz, 2H), 3.28 (t, *J* = 8.7 Hz, 1H), 2.98

(t, J = 7.5 Hz, 2H), 2.02 (s, 3H), 1.99 (s, 3H), 1.73 – 1.61 (m, 4H), 1.44 (p, J = 7.7 Hz, 3H), 1.15 (d, J = 6.5 Hz, 6H).

^{13}C NMR (151 MHz, D$_2$O) δ 174.7, 174.3, 170.5 (formic acid), 103.1 (**C$_1$**), 102.5 (**C$_1$**), 101.9 (**C$_1$**), 101.8 (**C$_1$**), 101.8 (**C$_1$**), 100.8 (**C$_1$**), 98.63 (**C$_1$**), 98.60 (**C$_1$**), 81.8, 79.1, 75.4, 75.1, 74.91, 74.85, 74.72, 74.68, 74.5, 73.4, 73.3, 73.0, 72.8, 72.5, 71.9, 71.03, 71.01, 70.1, 69.8, 69.21, 69.18, 68.8, 68.3, 68.2, 67.7, 66.72, 66.69, 61.5, 60.0, 59.74, 59.72, 59.6, 56.0, 55.6, 50.2, 39.3, 28.1, 26.4, 22.5, 22.2, 22.1, 15.3.

HRMS (QToF): Calcd for $C_{57}H_{100}N_3O_{39}$ [M + H]$^+$ 1450.5929; found 1450.5986.

^{1}H NMR (700 MHz, D$_2$O) of **DFLNnH 41**:

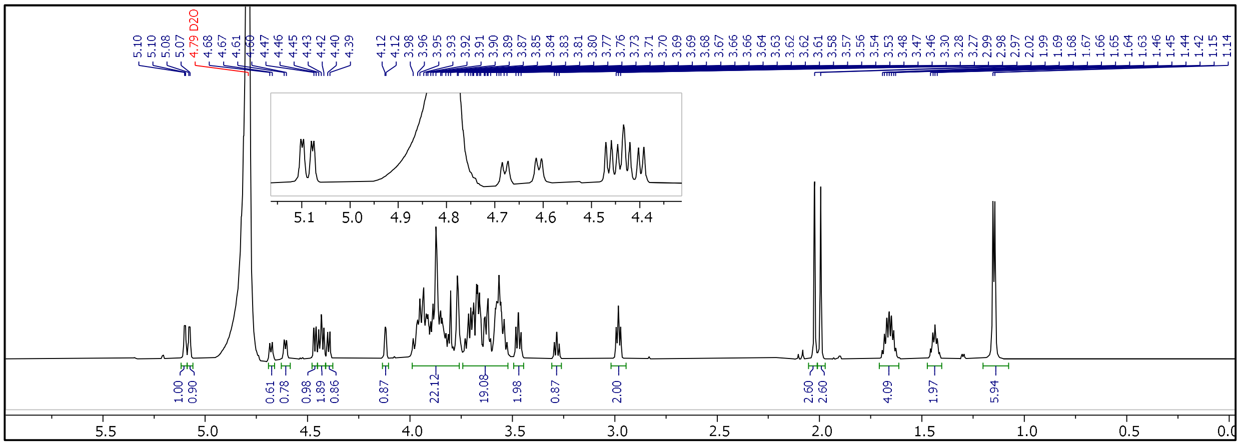

^{13}C NMR (151 MHz, D$_2$O) of **DFLNnH 41**:

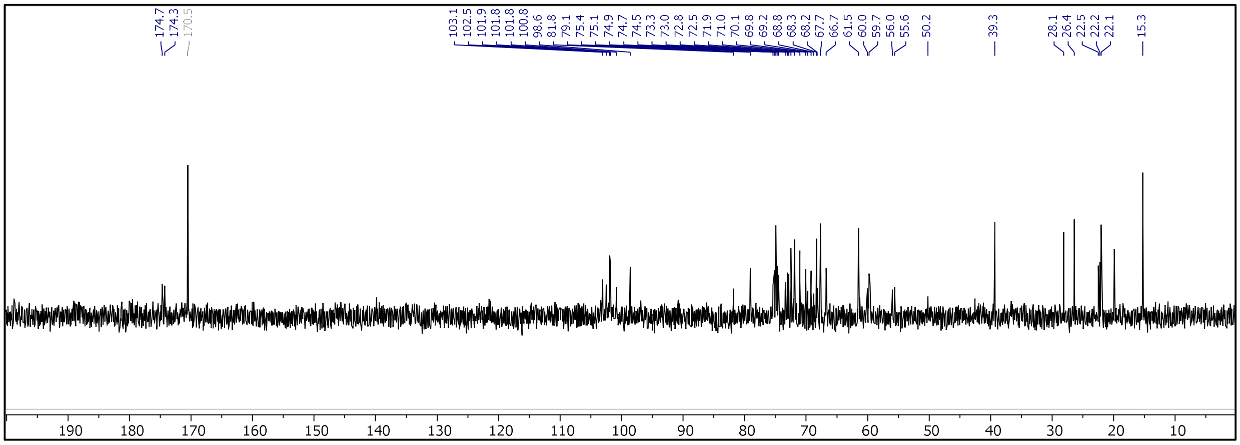

^{1}H-^{13}C HSQC NMR of **DFLNnH 41**:

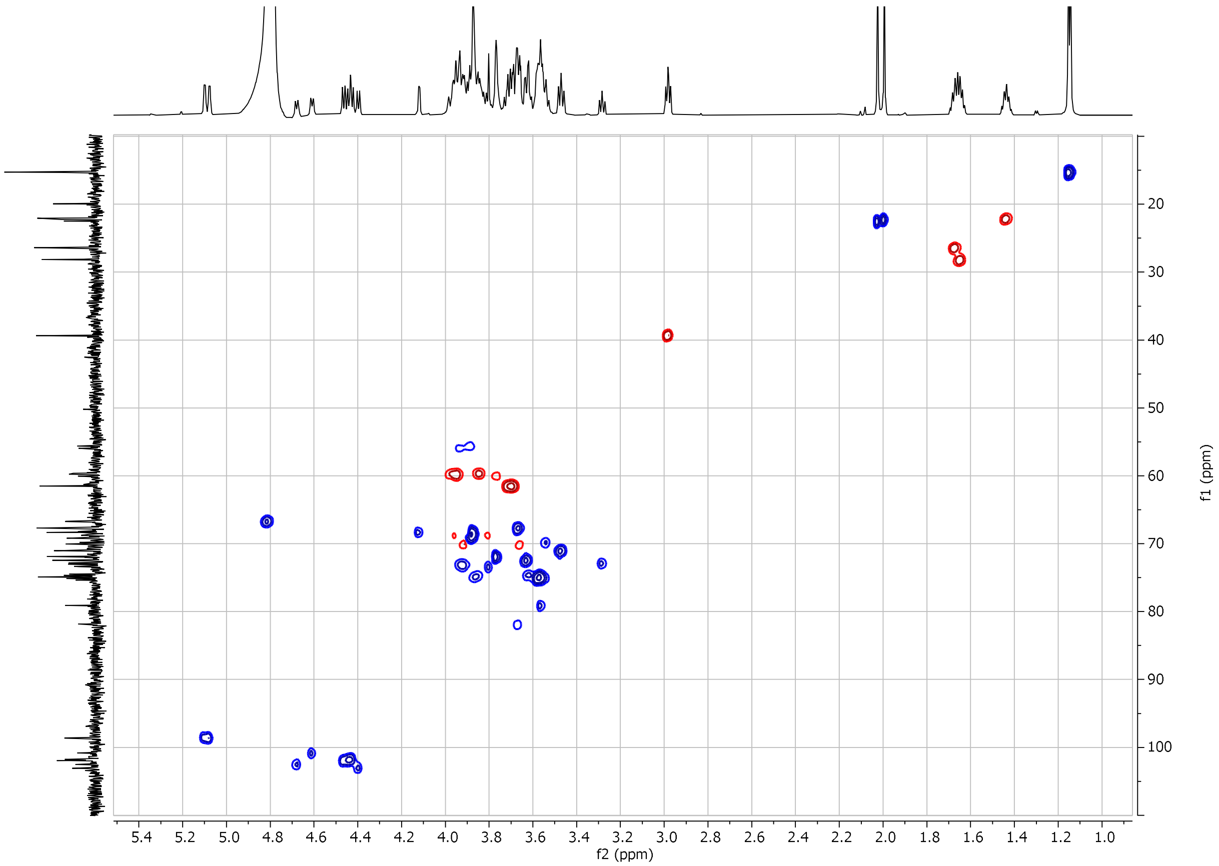

6.11 *iso*-Lacto-*N*-decaose ***i*LND 42**

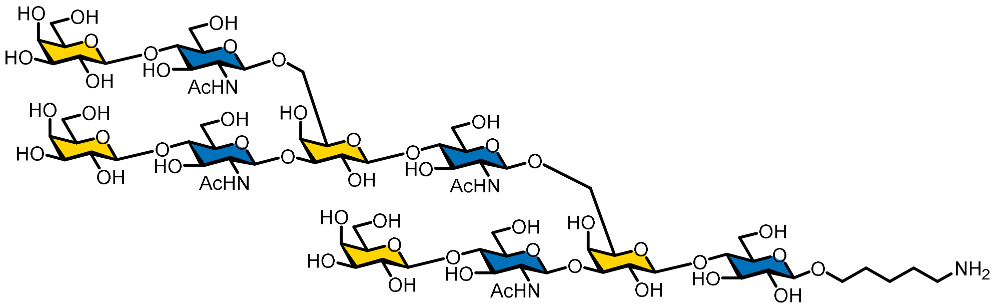

Deprotection procedure was shown as **General Deprotection Procedure** (Section: 4.4 Post-synthesizer Manipulation). Compound ***i*LND 42** (1.5 mg, 0.8 μmol, 40%) was obtained as a white solid by purification using preparative RP-HPLC (**Method H**) and lyophilizer.

(A) Analytical RP-HPLC of crude ***i*LND 42** (**Method E**, ELSD trace, t_R = 30.7 min)

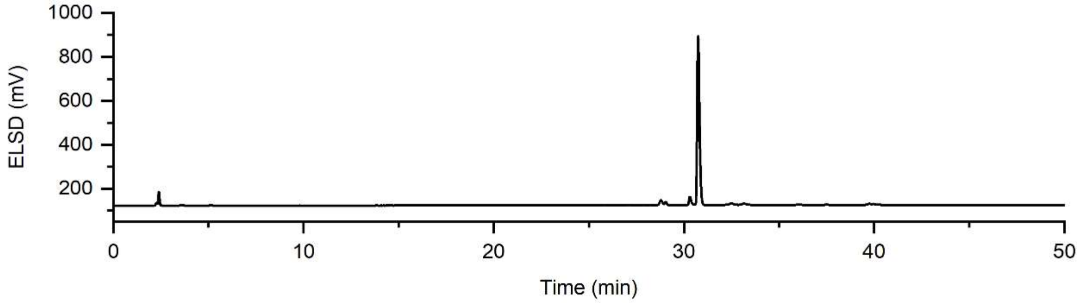

(B) Analytical RP-HPLC of pure ***i*LND 42** (**Method E**, ELSD trace, t_R = 31.2 min)

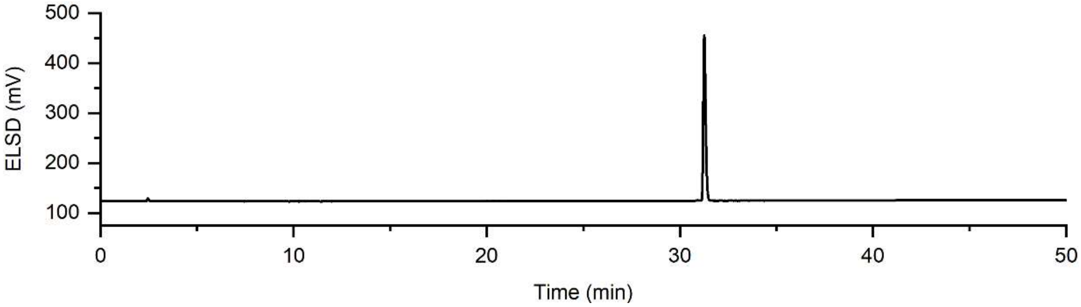

^{1}H NMR (600 MHz, D_2O) δ 4.71 (d, *J* = 8.3 Hz, 1H, **$H_1β$**), 4.71 (d, *J* = 8.5 Hz, 1H, **$H_1β$**), 4.64 (d, *J* = 8.2 Hz, 1H, **$H_1β$**), 4.62 (d, *J* = 7.9 Hz, 1H, **$H_1β$**), 4.50 (d, *J* = 8.0 Hz, 1H, **$H_1β$**), 4.49 (d, *J* = 7.8 Hz, 1H, **$H_1β$**), 4.49 (d, *J* = 7.8 Hz, 1H, **$H_1β$**), 4.48 (d, *J* = 7.7 Hz, 1H, **$H_1β$**), 4.46 (d, *J* = 7.8 Hz, 1H, **$H_1β$**), 4.44 (d, *J* = 7.9 Hz, 1H, **$H_1β$**), 4.16 (d, *J* = 3.2 Hz, 1H), 4.07 – 3.91 (m, 12H), 3.91 – 3.64 (m, 35H), 3.65 – 3.51 (m, 12H), 3.32 (dd, *J* = 8.9, 8.3 Hz, 1H), 3.02 (d, *J* = 7.5 Hz, 2H), 2.07 (s, 6H), 2.04 (s, 6H), 1.82 – 1.59 (m, 4H), 1.47 (p, *J* = 7.7 Hz, 2H).

^{13}C NMR (151 MHz, D_2O) δ 174.9, 174.5, 174.4, 170.9 (formic acid), 102.99 (**C_1**), 102.98 (**C_1**), 102.9 (**C_1**), 102.73 (**C_1**), 102.70 (**C_1**), 102.0 (**C_1**), 100.95 (**C_1**), 100.9 (**C_1**), 81.8, 78.9, 78.3, 78.1, 75.3, 74.7, 74.6, 74.54, 74.48, 73.7, 73.5, 72.8, 72.5, 72.3, 72.2, 71.0, 70.1, 69.8, 68.7, 68.5, 68.4, 61.0, 60.00, 59.97, 59.8, 55.2, 55.0, 54.9, 39.3, 28.1, 26.4, 22.4, 22.2, 22.1.

HRMS (QToF): Calcd for $C_{73}H_{126}N_5O_{51}$ $[M + H]^+$ 1888.7414; found 1888.7499.

^{1}H NMR (600 MHz, D_2O) of ***i*LND 42**:

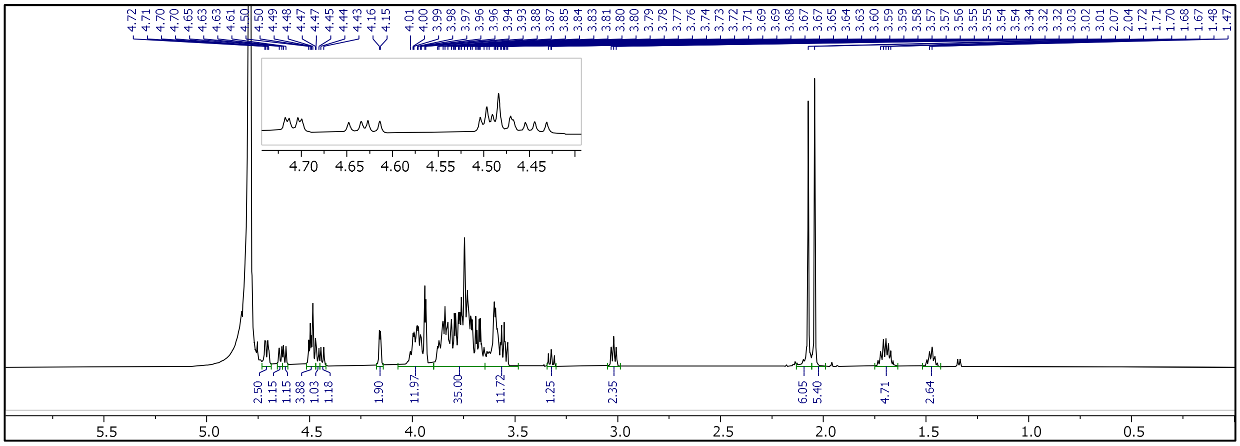

^{13}C NMR (151 MHz, D_2O) of ***i*LND 42**:

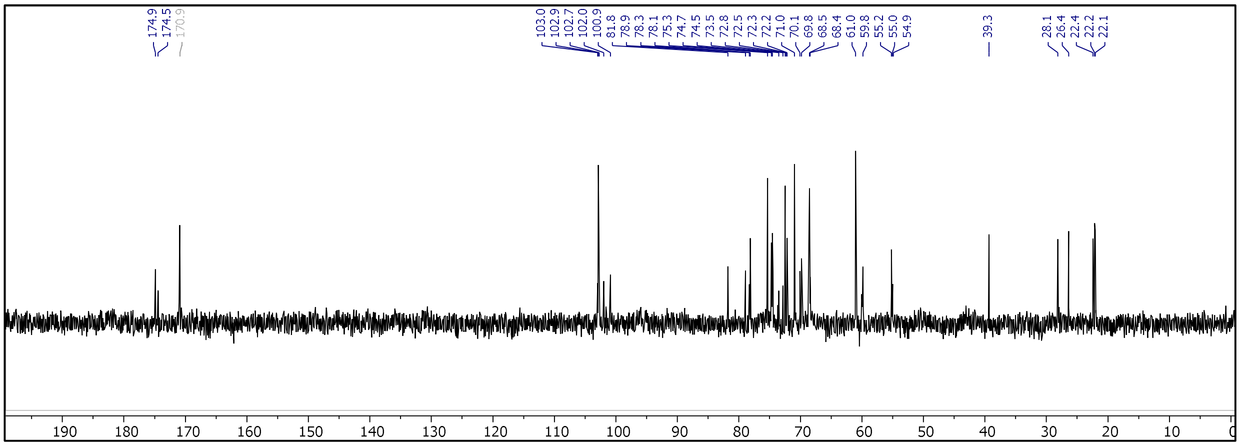

^{1}H-^{13}C HSQC NMR of ***i*LND 42**:

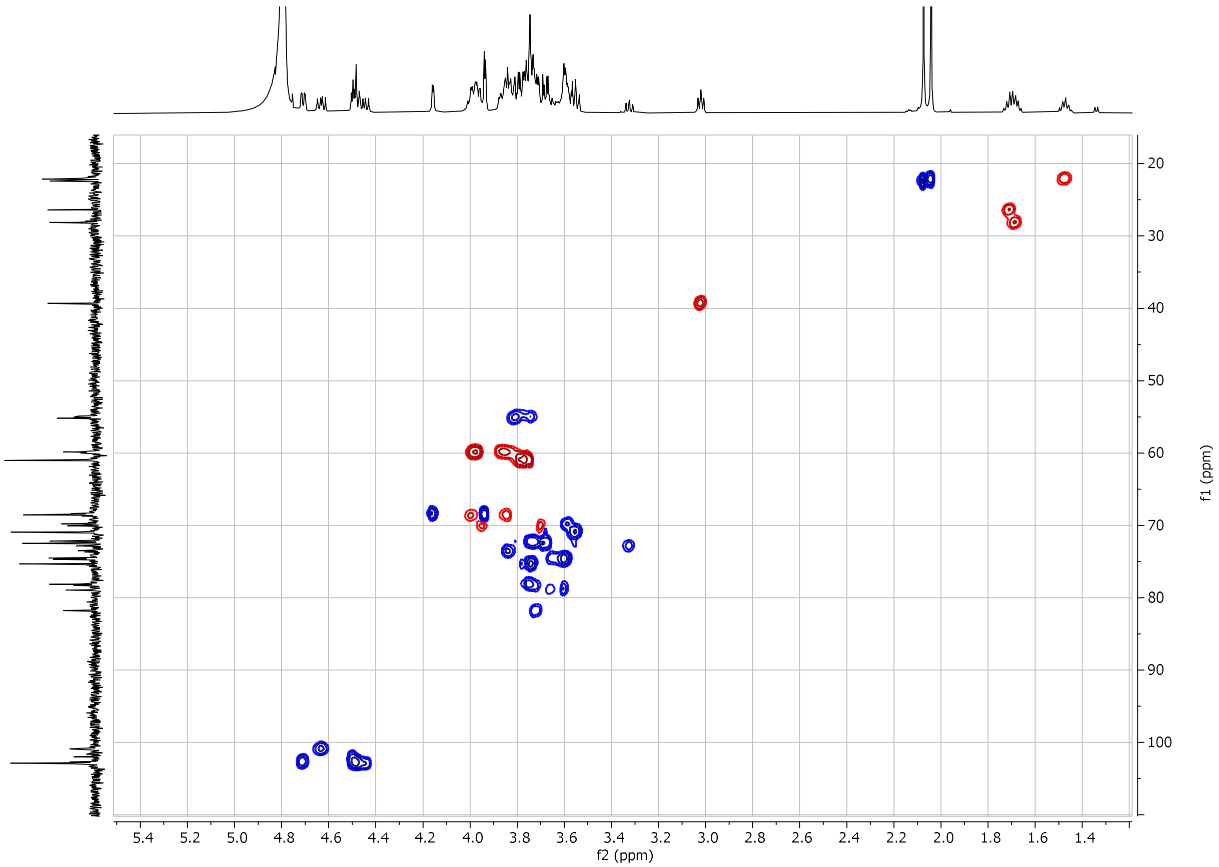

6.12 Fucosyllacto-*N*-neohexaose II **FLNnH II 43**

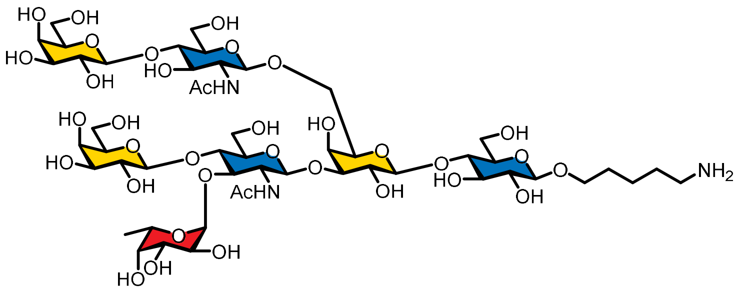

Deprotection procedure was shown as **General Deprotection Procedure** (Section: 4.4 Post-synthesizer Manipulation). Compound **FLNnH II 43** (1.3 mg, 1.0 µmol, 45%) was obtained as a white solid by purification using preparative RP-HPLC (**Method I**) and lyophilizer.

(A) Analytical RP-HPLC of crude **FLNnH II 43** (**Method F**, ELSD trace, t_R = 25.9 min)

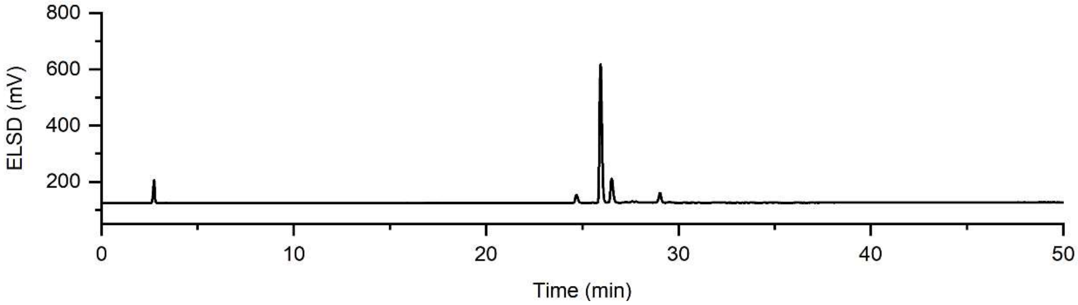

(B) Analytical RP-HPLC of pure **FLNnH II 43** (**Method F**, ELSD trace, t_R = 25.8 min)

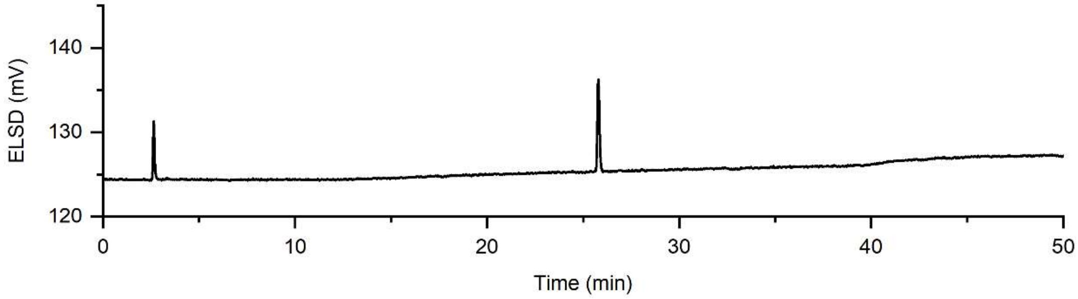

^{1}H NMR (700 MHz, D$_2$O) δ 5.15 (d, *J* = 3.8 Hz, 1H, **H$_1$α**), 4.86 (p, *J* = 6.3 Hz, 1H), 4.72 (d, *J* = 8.3 Hz, 1H, **H$_1$β**), 4.66 (d, *J* = 8.1 Hz, 1H, **H$_1$β**), 4.52 (d, *J* = 8.1 Hz, 1H, **H$_1$β**), 4.50 (d, *J* = 7.8 Hz, 1H, **H$_1$β**), 4.49 (d, *J* = 7.7 Hz, 1H, **H$_1$β**), 4.45 (d, *J* = 7.9 Hz, 1H, **H$_1$β**), 4.18 (d, *J* = 2.9 Hz, 1H), 4.07 – 3.84 (m, 15H), 3.85 – 3.48 (m, 24H), 3.34 (t, *J* = 8.7 Hz, 1H), 3.04 (t, *J* = 7.5 Hz, 2H), 2.09 (s, 3H), 2.05 (s, 3H), 1.80 – 1.66 (m, 4H),1.49 (p, *J* = 7.7 Hz, 2H), 1.20 (d, *J* = 6.6 Hz, 3H).

^{13}C NMR (151 MHz, D_2O) δ 174.7, 174.5, 103.0 (**C_1**), 102.9 (**C_1**), 102.5 (**C_1**), 102.0 (**C_1**), 101.8 (**C_1**), 101.0 (**C_1**), 98.6 (**C_1**), 81.9, 79.0, 78.4, 75.4, 75.1, 74.9, 74.8, 74.7, 74.5, 73.5, 73.1, 72.9, 72.5, 71.9, 71.1, 71.0, 70.1, 69.8, 69.2, 68.8, 68.6, 68.4, 67.7, 66.7, 61.5, 61.0, 60.1, 59.7, 56.0, 55.0, 39.4, 28.2, 26.4, 22.4, 22.3, 22.1, 15.3.

HRMS (QToF): Calcd for $C_{51}H_{90}N_3O_{35}SNa$ $[M + H]^+$ 1304.5350; found 1304.5411.

^{1}H NMR (700 MHz, D_2O) of **FLNnH II (43)**

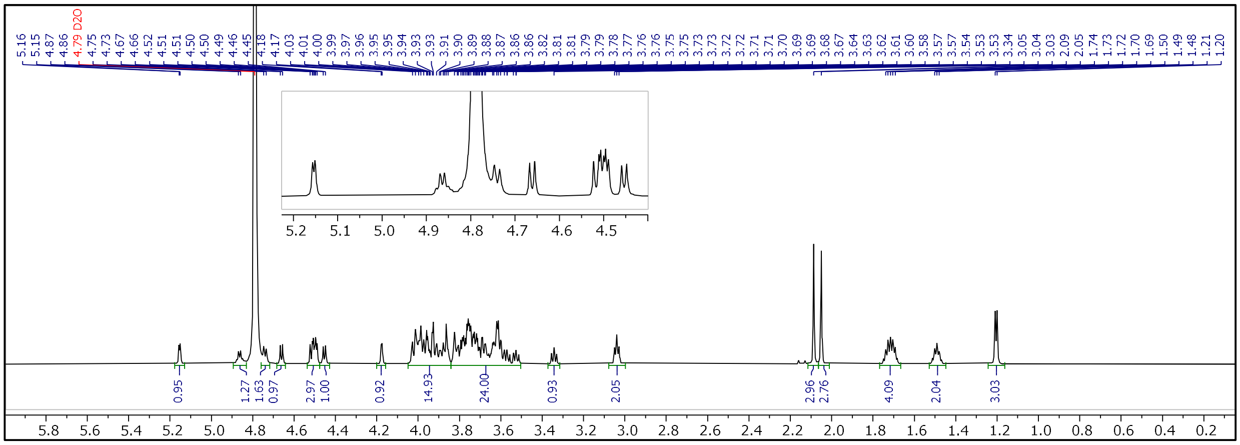

^{13}C NMR (151 MHz, D_2O) of **FLNnH II 43**:

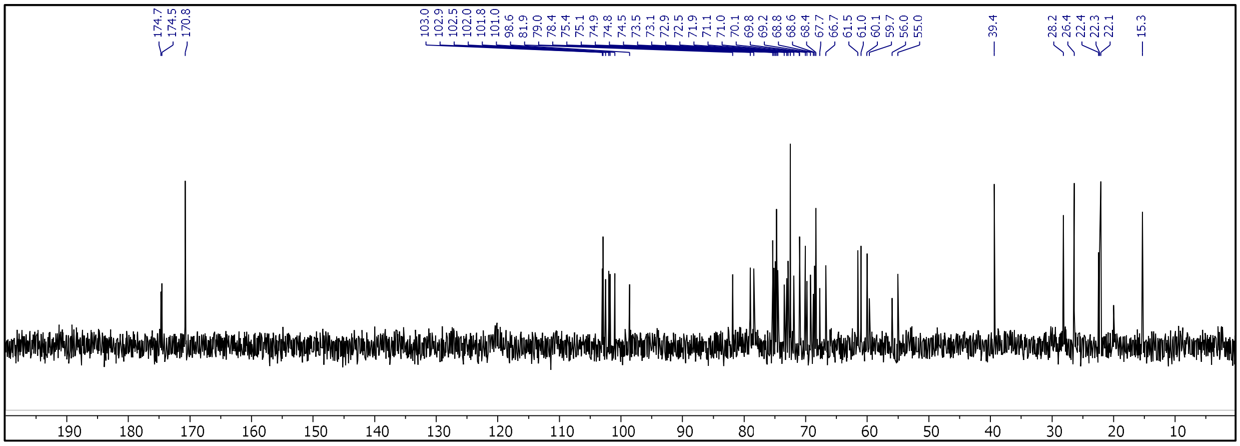

^{1}H-^{13}C HSQC NMR of **FLNnH II 43**:

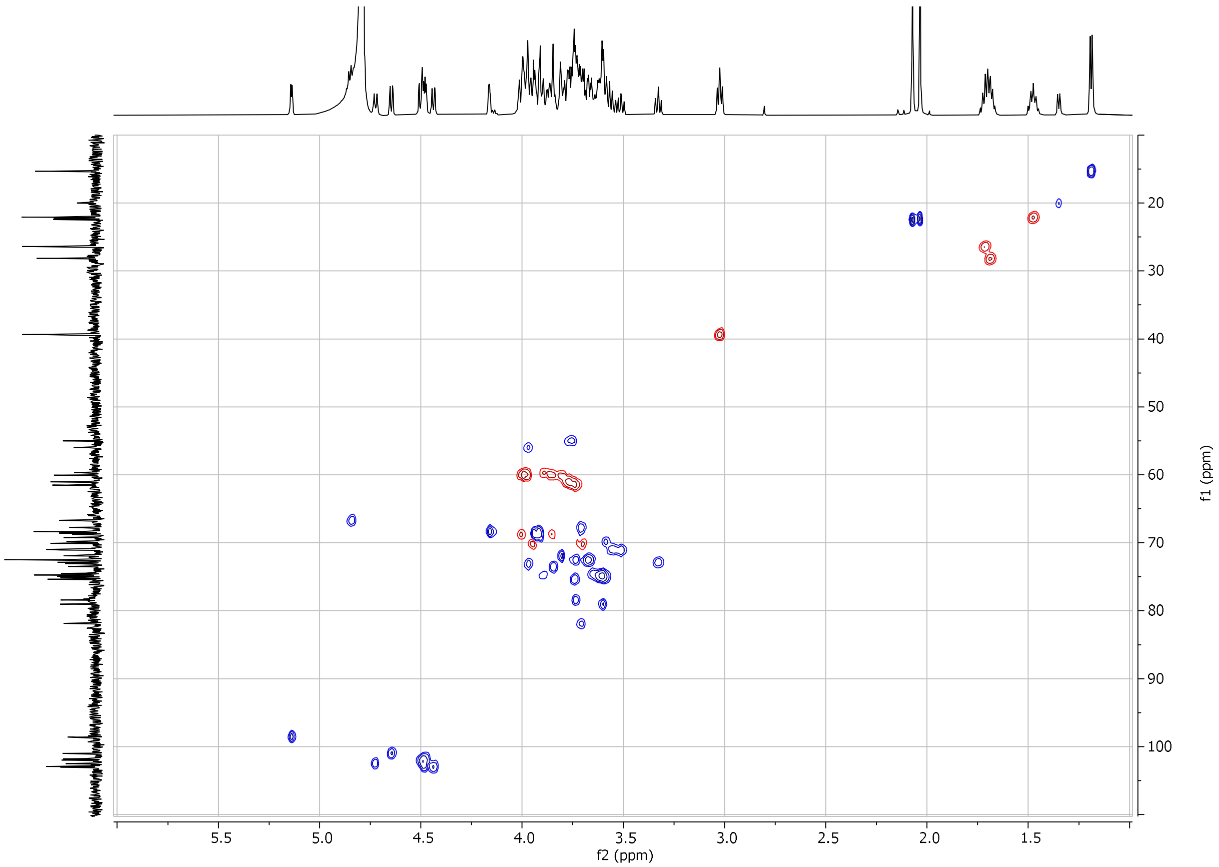

6.13 Lacto-*N*-difuco-hexoase II **LNDFH II 44**

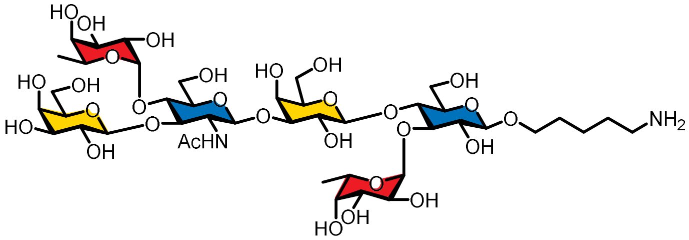

Deprotection procedure was shown as **General Deprotection Procedure** (Section: 4.4 Post-synthesizer Manipulation). Compound **LNDFH II (44)** (0.97 mg, 0.9 µmol, 40%) was obtained as a white solid by purification using preparative RP-HPLC (**Method J**) and lyophilizer.

(A) Analytical RP-HPLC of crude **LNDFH II 44** (**Method G**, ELSD trace, t_R = 23.9 min)

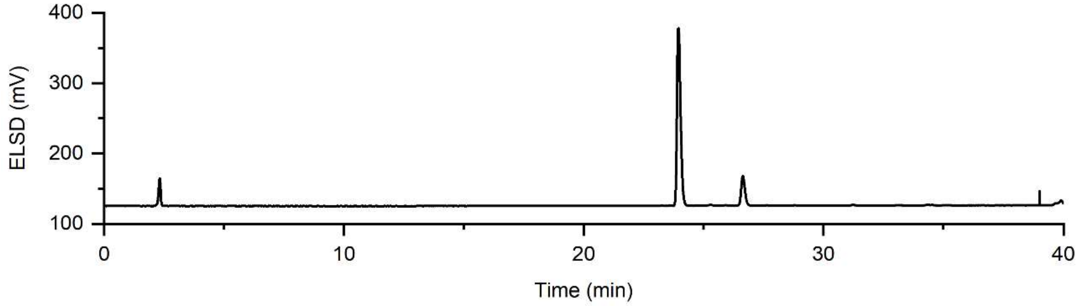

(B) Analytical RP-HPLC of pure **LNDFH II 44** (**Method G**, ELSD trace, t_R = 25.0 min)

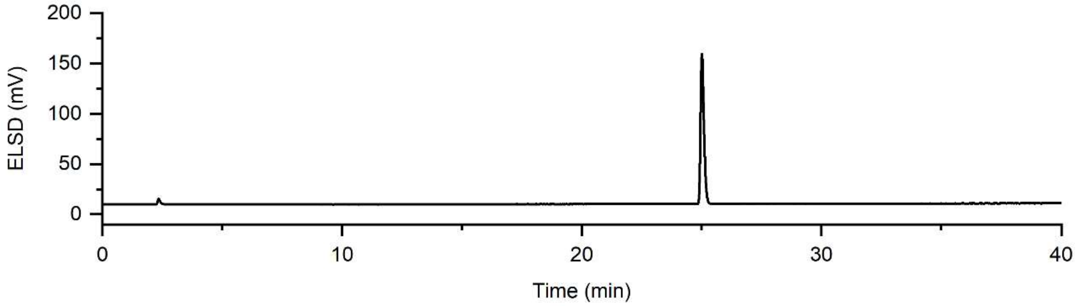

^{1}H NMR (600 MHz, D_2O) δ 5.44 (d, *J* = 4.0 Hz, 1H, **$H_1\alpha$**), 5.04 (d, *J* = 3.9 Hz, 1H, **$H_1\alpha$**), 4.92 – 4.87 (m, 1H), 4.70 (d, *J* = 8.4 Hz, 1H, **$H_1\beta$**), 4.52 (d, *J* = 7.7 Hz, 1H, **$H_1\beta$**), 4.48 (d, *J* = 8.1 Hz, 1H, **$H_1\beta$**), 4.42 (d, *J* = 7.8 Hz, 1H, **$H_1\beta$**), 4.11 (d, *J* = 3.3 Hz, 1H), 4.08 (d, *J* = 9.7 Hz, 1H), 4.02 – 3.92 (m, 5H), 3.92 – 3.66 (m, 17H), 3.63 (dd, *J* = 9.8, 3.4 Hz, 1H), 3.60 – 3.56 (m, 3H), 3.55 (dt, *J* = 9.7, 3.1 Hz, 1H), 3.52 – 3.48 (m, 3H), 3.02 (t, *J* = 7.5 Hz, 2H), 2.04 (s, 3H), 1.82 – 1.63 (m, 4H), 1.47 (p, *J* = 7.6 Hz, 2H), 1.19 (d, *J* = 6.6 Hz, 3H), 1.17 (d, *J* = 6.7 Hz, 3H).

^{13}C NMR (151 MHz, D_2O) δ 174.7, 170.7, 102.8 (**C_1**), 102.6 (**C_1**), 102.1 (**C_1**), 101.7 (**C_1**), 98.5 (**C_1**), 98.0 (**C_1**), 81.6, 77.0, 75.9, 75.3, 75.2, 74.8, 74.5, 72.4, 72.3, 72.1, 71.9, 70.6, 70.5, 70.1, 69.2, 68.3, 68.2, 68.0, 67.8, 66.8, 66.5, 61.6, 61.5, 59.8, 59.6, 55.9, 39.3, 28.1, 26.4, 22.3, 22.1, 15.3, 15.2.

HRMS (QToF): Calcd for $C_{43}H_{77}N_2O_{29}$ a $[M + H]^+$ 1085.4612; found 1085.4658.

^{1}H NMR (600 MHz, D_2O) of **LNDFH II 44**:

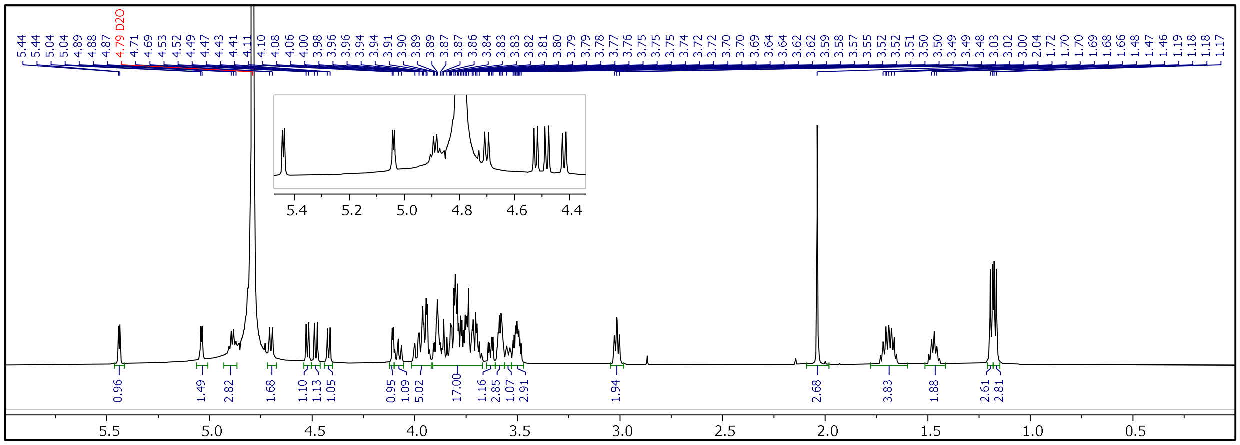

^{13}C NMR (151 MHz, D_2O) of **LNDFH II 44**:

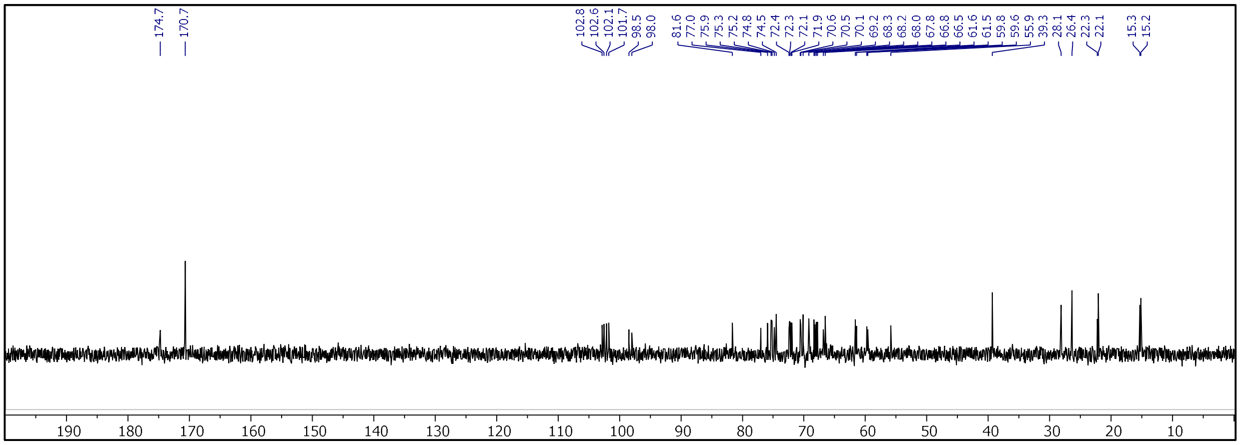

^1H-^{13}C HSQC NMR of **LNDFH II 44**:

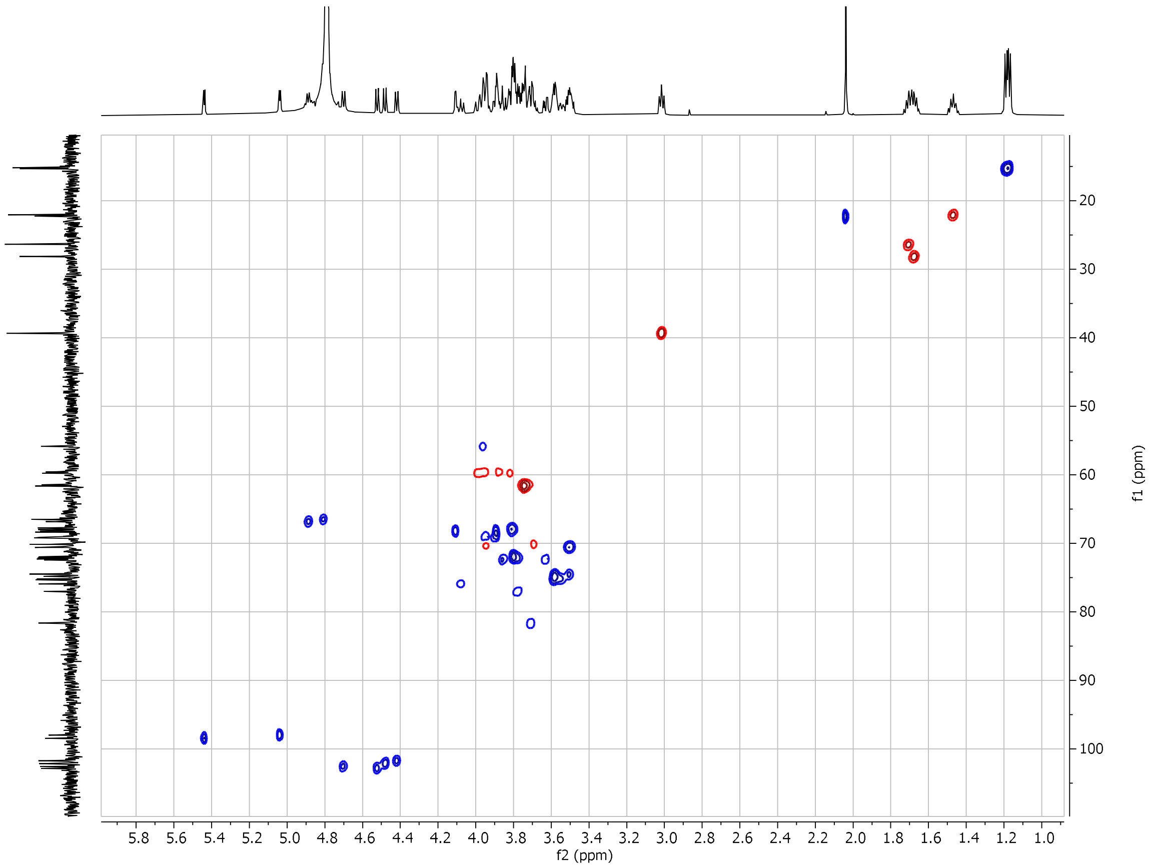

6.14 Lacto-*N*-fucopentaose I **LNFP I 45**

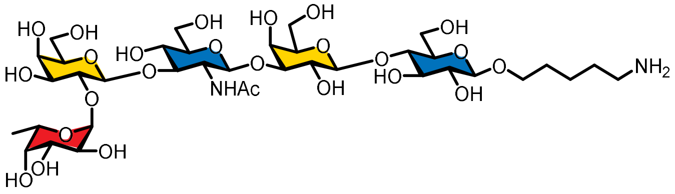

Deprotection procedure was shown as **General Deprotection Procedure** (Section: 4.4 Post-synthesizer Manipulation). Compound **LNFP I 45** (1.0 mg, 1.1 μmol, 42%) was obtained as a white solid by purification using preparative RP-HPLC (**Method J**) and lyophilizer.

(A) Analytical RP-HPLC of the crude **LNFP I 45** (**Method G**, ELSD trace, t_R = 30.8 min)

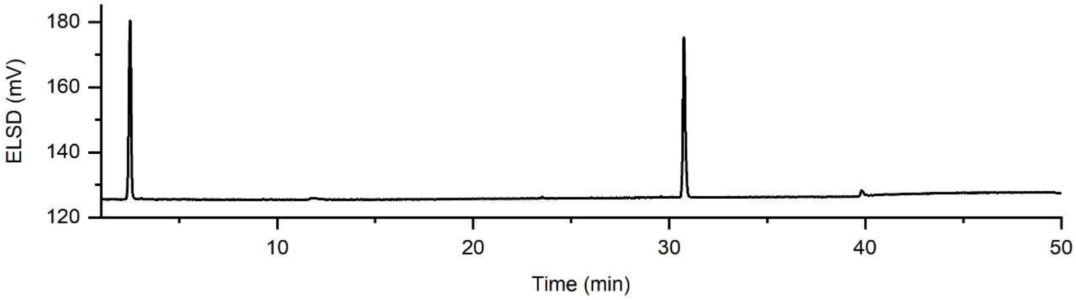

(B) Analytical RP-HPLC of pure **LNFP I 45** (**Method G**, ELSD trace, t_R = 30.2 min)

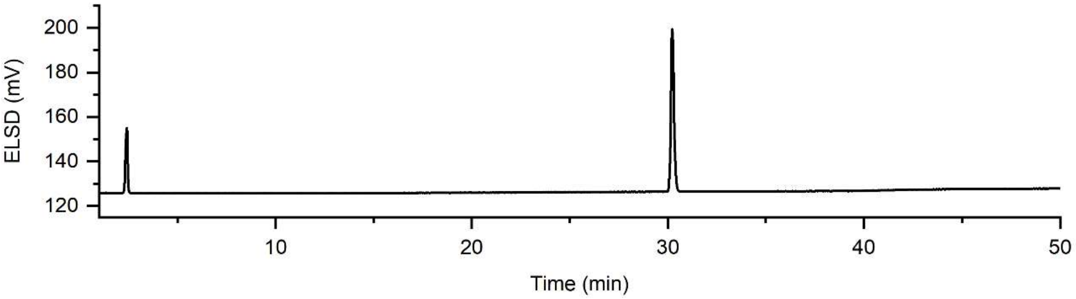

^{1}H NMR (700 MHz, D_2O) δ 5.21 (d, *J* = 4.1 Hz, 1H, **$H_1α$**), 4.66 (d, *J* = 7.7 Hz, 1H, **$H_1β$**), 4.64 (d, *J* = 8.5 Hz, 1H, **$H_1β$**), 4.50 (d, *J* = 8.0 Hz, 1H, **$H_1β$**), 4.44 (d, *J* = 7.9 Hz, 1H, **$H_1β$**), 4.31 (q, *J* = 6.7 Hz, 1H), 4.16 (d, *J* = 3.2 Hz, 1H), 4.04 – 3.89 (m, 5H), 3.87 – 3.55 (m, 20H), 3.54 (d, *J* = 8.6 Hz, 1H), 3.52 – 3.49 (m, 1H), 3.32 (t, *J* = 8.6 Hz, 1H), 3.02 (t, *J* = 7.5 Hz, 2H), 2.07 (s, 3H), 1.82 – 1.60 (m, 4H), 1.48 (p, *J* = 7.5 Hz, 2H), 1.25 (d, *J* = 6.7 Hz, 3H).

^{13}C NMR (176 MHz, D_2O) δ 174.2, 170.5, 103.2 (**C_1**), 103.0 (**C_1**), 102.0 (**C_1**), 100.3 (**C_1**), 99.5 (**C_1**), 81.6, 78.3, 77.2, 76.7, 75.3, 75.1, 74.8, 74.4, 73.5, 72.8, 71.9, 70.2, 70.1, 69.4, 69.1, 68.6, 68.5, 68.1, 66.5, 61.2, 61.0, 60.4, 60.1, 55.0, 39.4, 28.1, 26.4, 22.1, 22.1, 15.3.

HRMS (QToF): Calcd for $C_{37}H_{67}N_2O_{25}$ a $[M + H]^+$ 939.4033; found 939.4070.

^{1}H NMR (700 MHz, D_2O) of **LNFP I (45)**:

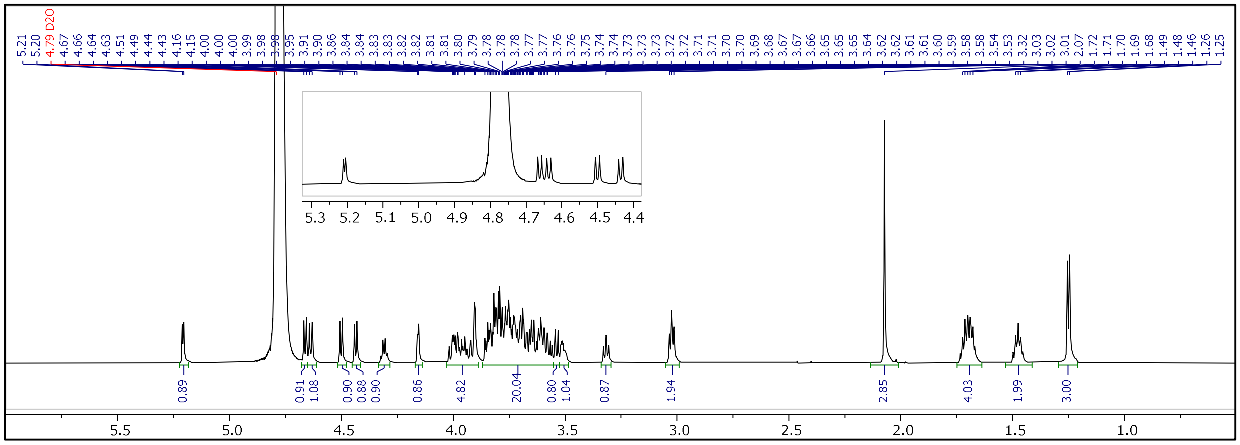

^{13}C NMR (176 MHz, D_2O) of **LNFP I 45**:

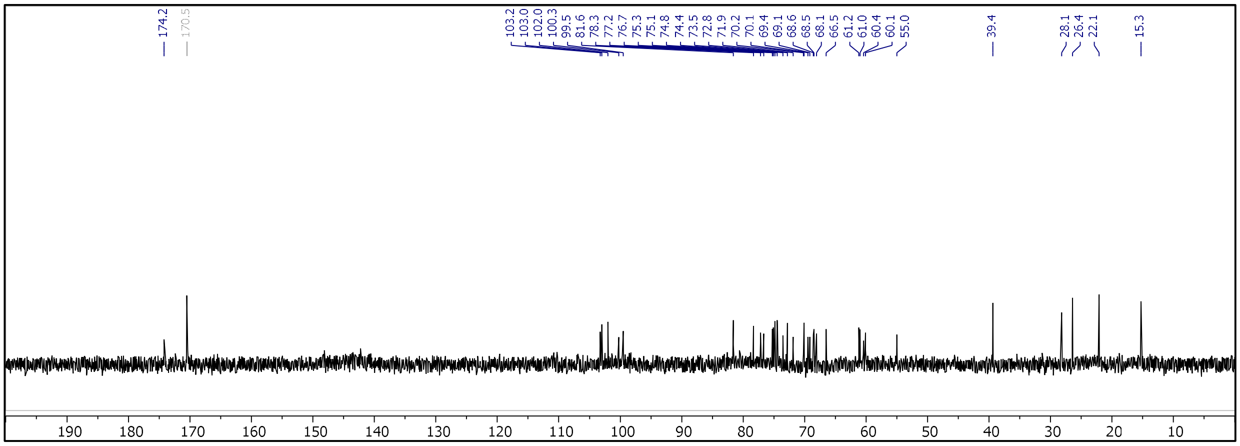

^{1}H-^{13}C HSQC NMR of **LNFP I 45**:

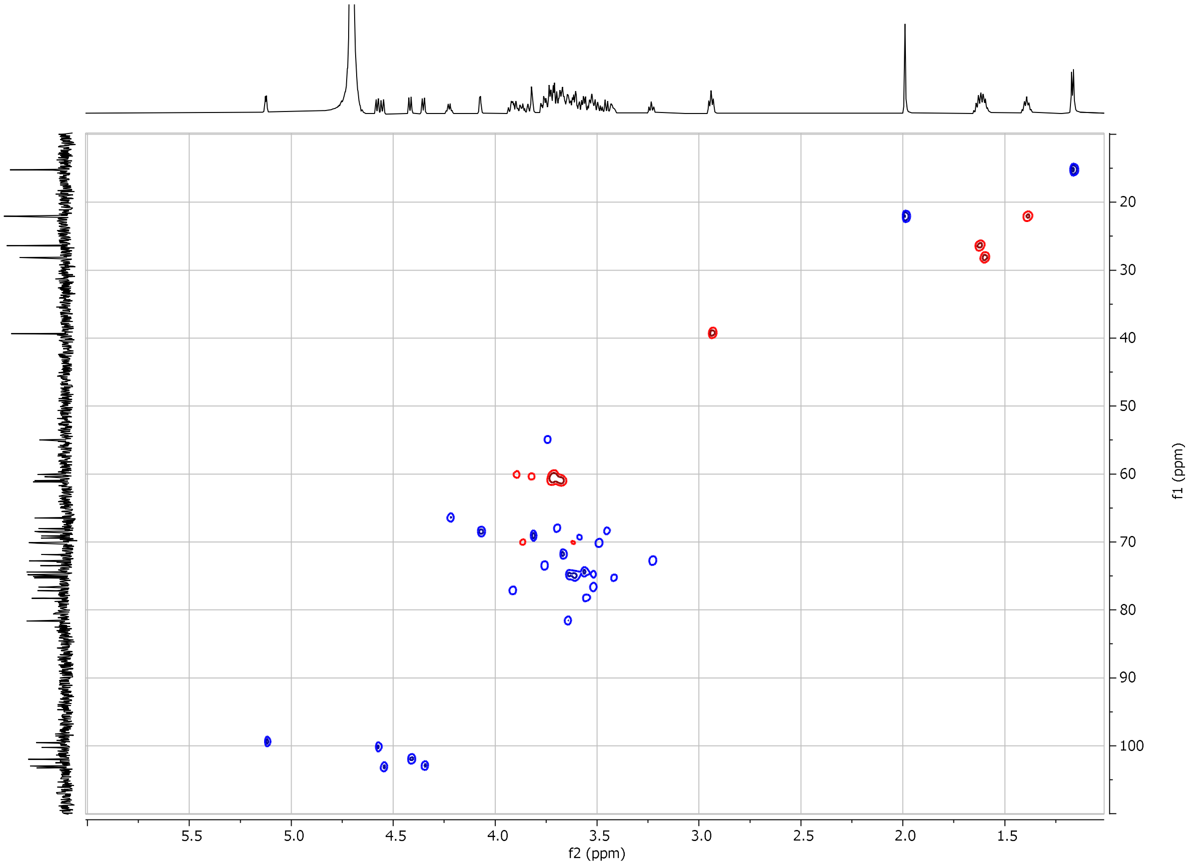

6.15 Lacto-*N*-difuco-hexoase I **LNDFH I 46**

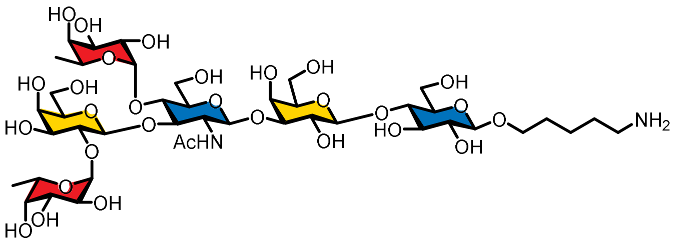

Deprotection procedure was shown as **General Deprotection Procedure** (Section: 4.4 Post-synthesizer Manipulation). Compound **LNDFH I 46** (0.8 mg, 0.7 μmol, 35%) was obtained as a white solid by purification using preparative RP-HPLC (**Method J**) and lyophilizer.

(A) Analytical RP-HPLC of the crude **LNDFH I 46** (**Method G**, ELSD trace, t_R = 26.1 min)

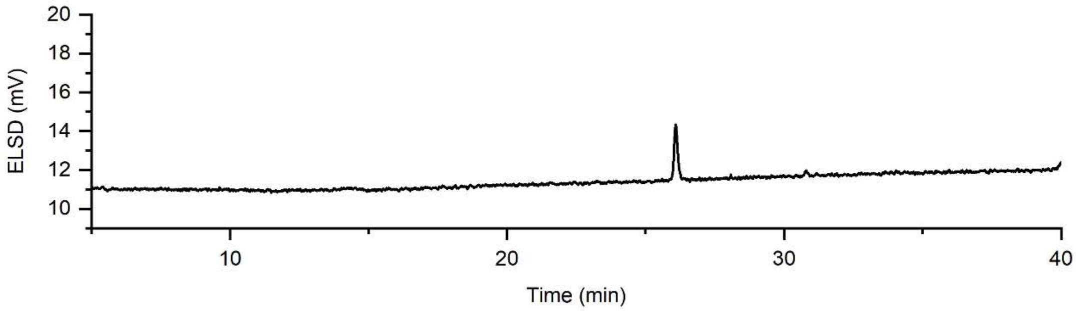

(B) Analytical RP-HPLC of pure **LNDFH I 46** (**Method G**, ELSD trace, t_R = 27.1 min)

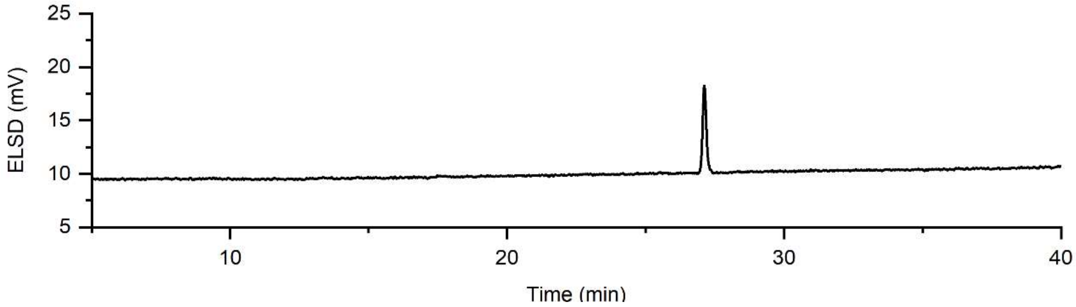

^{1}H NMR (700 MHz, D_2O) δ 5.18 (d, *J* = 4.1 Hz, 1H, **$H_1α$**), 5.05 (d, *J* = 3.9 Hz, 1H, **$H_1α$**), 4.92 – 4.87 (m, 1H), 4.68 (d, *J* = 7.8 Hz, 1H, **$H_1β$**), 4.62 (d, *J* = 8.5 Hz, 1H, **$H_1β$**), 4.51 (d, *J* = 8.0 Hz, 1H, **$H_1β$**), 4.44 (d, *J* = 7.9 Hz, 1H, **$H_1β$**), 4.37 (q, *J* = 6.4 Hz, 1H), 4.22 – 4.13 (m, 2H), 4.05 – 3.93 (m, 4H), 3.92 – 3.69 (m, 18H), 3.69 – 3.52 (m, 7H), 3.32 (t, *J* = 8.5 Hz, 1H), 3.03 (t, *J* = 7.5 Hz, 2H), 2.09 (s, 3H), 1.79 – 1.64 (m, 4H), 1.48 (p, *J* = 7.8 Hz, 2H), 1.30 (d, *J* = 6.7 Hz, 3H), 1.28 (d, *J* = 6.6 Hz, 3H).

^{13}C NMR (151 MHz, D_2O) δ 174.2, 171.1, 103.2 (**C_1**), 103.0 (**C_1**), 102.0 (**C_1**), 100.6 (**C_1**), 99.6 (**C_1**), 97.8 (**C_1**), 81.6, 78.3, 76.5, 75.2, 74.8, 74.8, 74.4, 72.8, 72.0, 70.1, 70.1, 69.5, 69.1, 68.7, 68.6, 68.5, 68.3, 67.8, 67.0, 66.2, 61.6, 61.0, 60.1, 59.5, 55.8, 39.4, 28.2, 26.5, 22.1, 15.4, 15.3.

HRMS (QToF): Calcd for $C_{43}H_{77}N_2O_{29}$ a $[M + H]^+$ 1085.4612; found 1085.4626.

^{1}H NMR (700 MHz, D_2O) of **LNDFH I 46**:

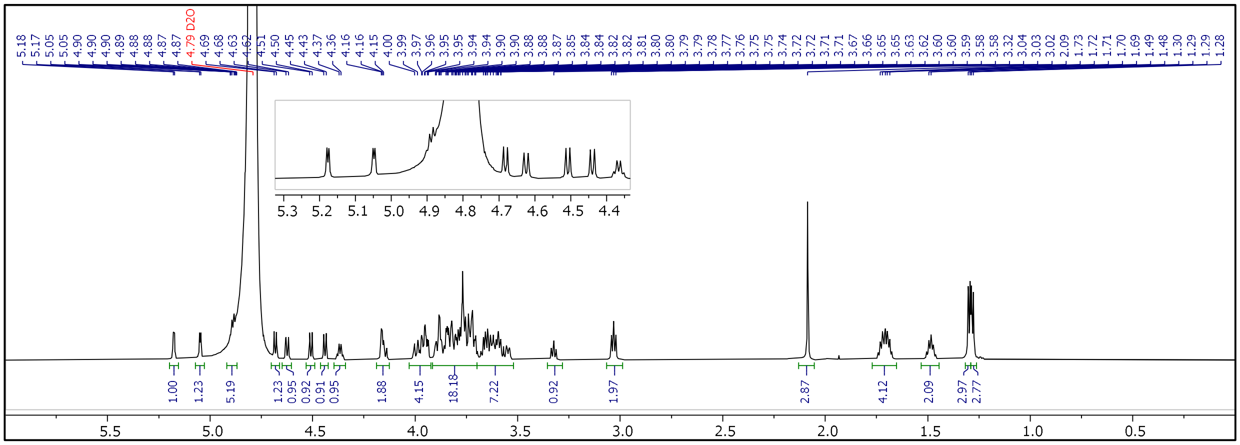

^{1}H-^{13}C HSQC NMR of **LNDFH I 46**:

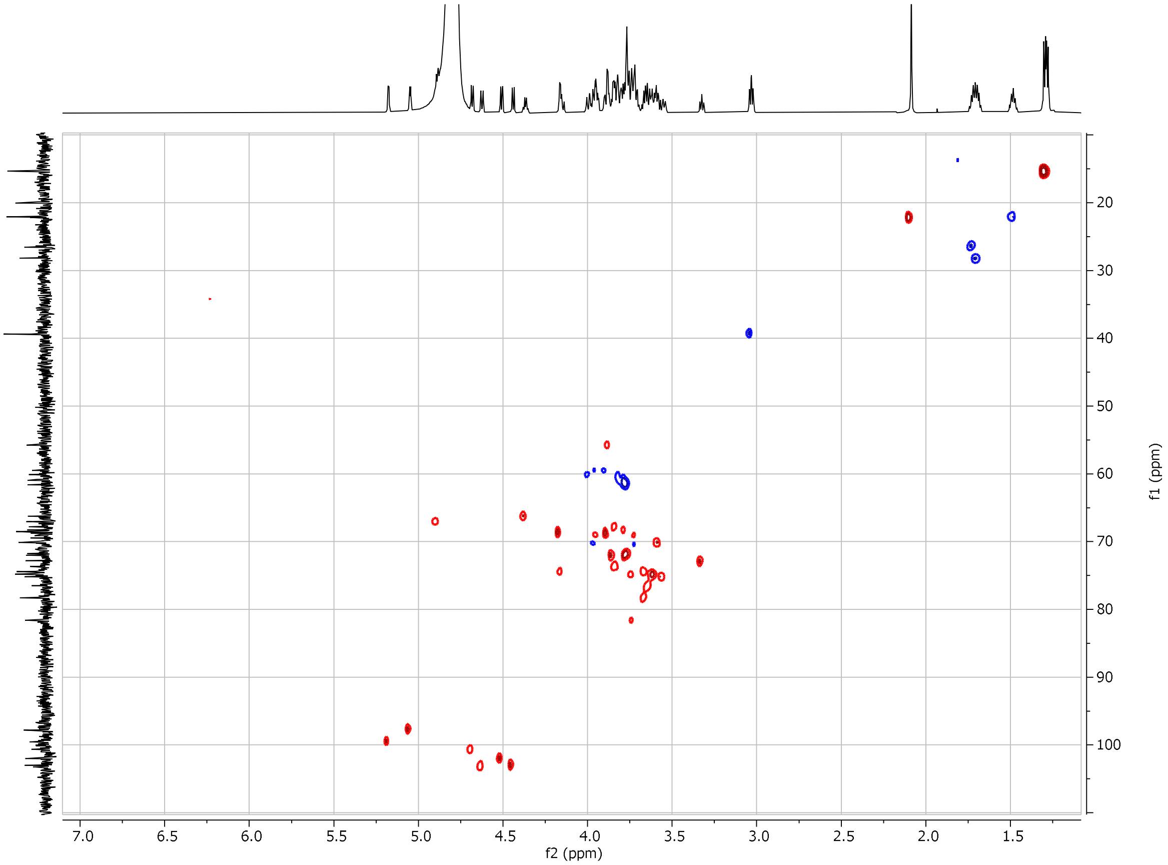

7. References

1. Grann Hansen, S. & Skrydstrup, T. Studies Directed to the Synthesis of Oligochitosans – Preparation of Building Blocks and Their Evaluation in Glycosylation Studies. *Eur. J. Org. Chem.* **2007**, 3392-3401 (2007).
2. Le Mai Hoang, K. *et al.* Traceless Photolabile Linker Expedites the Chemical Synthesis of Complex Oligosaccharides by Automated Glycan Assembly. *J. Am. Chem. Soc.* **141**, 9079-9086 (2019).
3. Pfeiffer, C. T. *et al.* Utilization of the *p*-nitrobenzyloxycarbonyl (pNZ) amine protecting group and pentafluorophenyl (Pfp) esters for the solid phase synthesis of spiroligomers. *Tetrahedron Lett.* **59**, 2884-2888 (2018).
4. Isidro-Llobet, A., Guasch-Camell, J., Álvarez, M. & Albericio, F. *p*-Nitrobenzyloxycarbonyl (pNZ) as a Temporary N^{α}-Protecting Group in Orthogonal Solid-Phase Peptide Synthesis – Avoiding Diketopiperazine and Aspartimide Formation. *Eur. J. Org. Chem.* **2005**, 3031-3039 (2005).
5. Jang, M., Lim, T., Park, B. Y. & Han, M. S. Metal-Free, Rapid, and Highly Chemoselective Reduction of Aromatic Nitro Compounds at Room Temperature. *J. Org. Chem.* **87**, 910-919 (2022).
6. Inamura, S. *et al.* Synthesis of peptidoglycan fragments and evaluation of their biological activity. *Org. Biomol. Chem.* **4**, 232-242 (2006).
7. Chang, C. W. *et al.* Automated Quantification of Hydroxyl Reactivities: Prediction of Glycosylation Reactions. *Angew. Chem. Int. Ed.* **60**, 12413-12423 (2021).
